# Supplementary material for: Cohesin positions the epigenetic reader Phf2 within the genome
Source: EMBO J. 2025 Jan 2;44(3):736–66. doi: 10.1038/s44318-024-00348-2 (PMC11790891; doi:10.1038/s44318-024-00348-2)
Supplement: Supplementary file 2 — Appendix [file 44318_2024_348_MOESM2_ESM.pdf]

Appendix for:

Cohesin positions the epigenetic reader Phf2 within the genome

Wen Tang†, Lorenzo Costantino†, Roman Stocsits, Gordana Wutz, Rene Ladurner, Otto Hudecz, Karl Mechtler, Jan-Michael Peters*

Research Institute of Molecular Pathology (IMP), Vienna Biocenter (VBC), Campus-Vienna-Biocenter 1, 1030 Vienna, Austria

† These authors contributed equally to this work.

*To whom correspondence should be addressed: Tel: +43 1797303000; E-mail: *peters@imp.ac.at*

Table of contents:

Appendix Figures

Appendix Figure S1: Identification of Phf2 as a cohesin-interacting protein. 2

Appendix Figure S2: Phf2 residues 660-819 are essential for cohesin binding and vermicelli localization. 4

Appendix Figure S3: Phf2 co-localizes with H3K4me3 and cohesin genome-wide........................ 6

Appendix Figure S4: Phf2 does not influence cohesin chromatin association. 7

Appendix Figure S5: Phf2 depletion limits the length of heterochromatic B compartments.......... 9

Appendix Figure S6: Phf2 depletion negatively affects a subset of TAD boundaries characterized by low levels of CTCF. 11

Appendix Figure S1

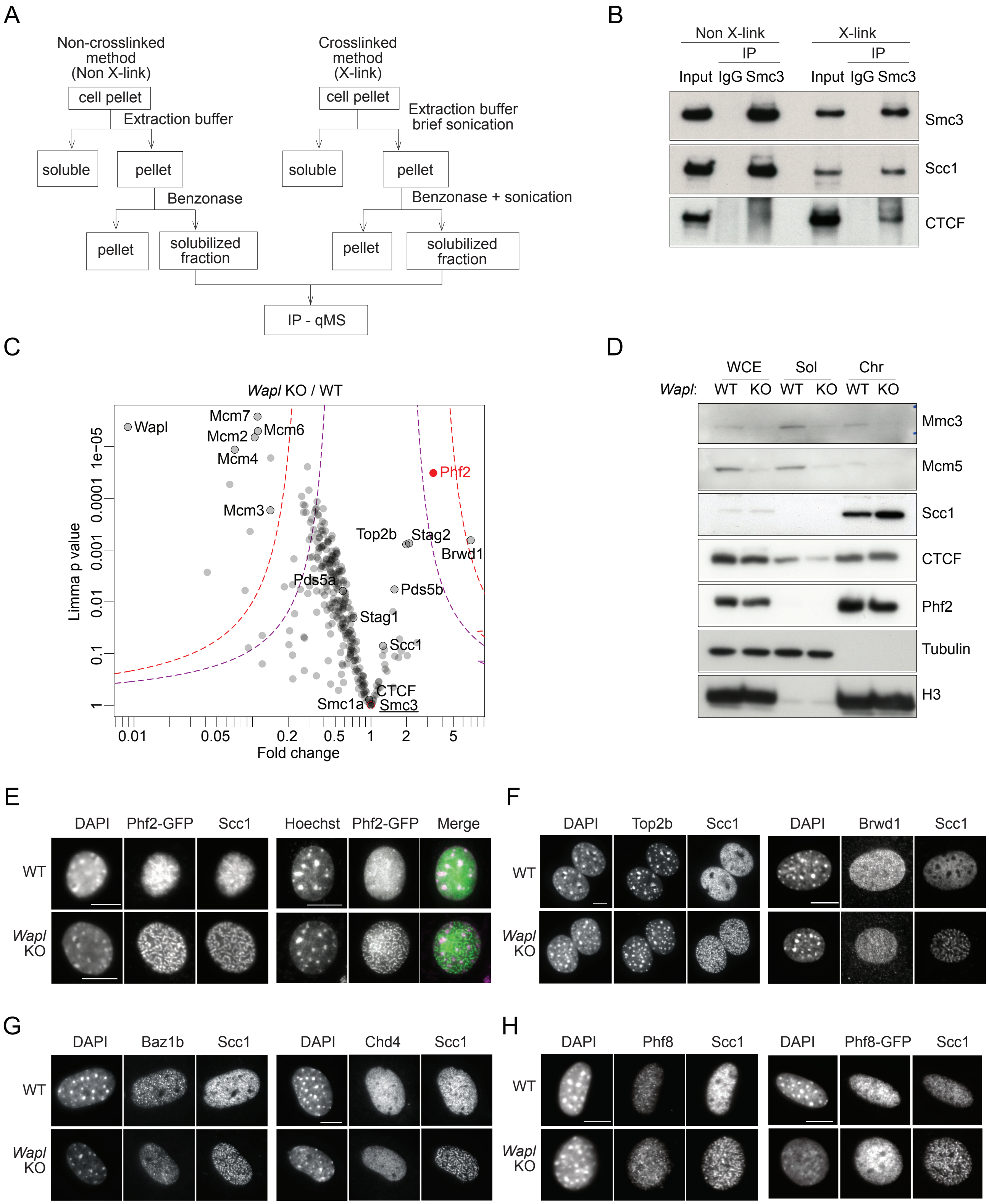

Appendix Figure S1: Identification of Phf2 as a cohesin-interacting protein.

A. Schematic representation of protocols for non-crosslinked method (Non X-link) and crosslinked method (X-link) with immunoprecipitation followed by quantitative mass-spectrometry (IP-qMS).

B. Immunoblot analysis of immunoprecipitated samples described in A. Immunoprecipitations were performed using anti-Smc3 antibody and proteins were detected using the indicated antibodies.

C. Volcano plot of proteins identified by Smc3 ChIP-qMS, normalized by bait protein (Smc3). The plot shows enriched proteins in the *Wapl* KO sample on the right and depleted proteins on the left. Biological replicates n=3. Statistical significance of differentially expressed proteins was determined using limma. Proteins above the purple and red dotted lines represent significance thresholds of $p < 0.05$ and $p < 0.01$, respectively. The top enriched protein, Phf2, is highlighted in red.

D. Immunoblot analysis of whole-cell extracts (WCE), soluble (Sol), and chromatin (Chr) fractions from WT and *Wapl* KO MEFs using indicated antibodies.

E. Fluorescence microscopy for Phf2 and Scc1 in WT and Wapl KO MEFs. Left panel: representative immunofluorescence images of immortalized WT or *Wapl* KO MEFs expressing Phf2-GFP stained with DAPI and antibodies to GFP and Scc1. Right panel: representative live-cell images of immortalized WT or *Wapl* KO MEFs expressing Phf2-GFP, DNA was stained with Hoechst. Scale bar, 10µm. Please note that a portion of this immunofluorescence image was used in Fig.1C.

F. Fluorescence microscopy for Top2b, Brwd1, and Scc1 in WT and *Wapl* KO. Representative immunofluorescence images of WT or *Wapl* KO MEFs stained with DAPI and antibodies to Top2b (left panel) or Brwd1 (right panel) and Scc1. Scale bar, 10µm.

G. Fluorescence microscopy for Baz1b, Chd4, and Scc1 in WT and *Wapl* KO. Representative immunofluorescence images of WT or *Wapl* KO MEFs stained with DAPI and antibodies to Baz1b (left panel) or Chd4 (right panel) and Scc1. Scale bar, 10µm.

H. Fluorescence microscopy for Phf8 and Scc1 in WT and *Wapl*. Left: Representative immunofluorescence images of WT or *Wapl* KO MEFs stained with DAPI and antibodies to Phf8 and Scc1. Right: Representative immunofluorescence images of immortalized WT or *Wapl* KO MEFs expressing Phf8-GFP stained with DAPI and antibodies to GFP and Scc1. Scale bar, 10µm.

Appendix Figure S2

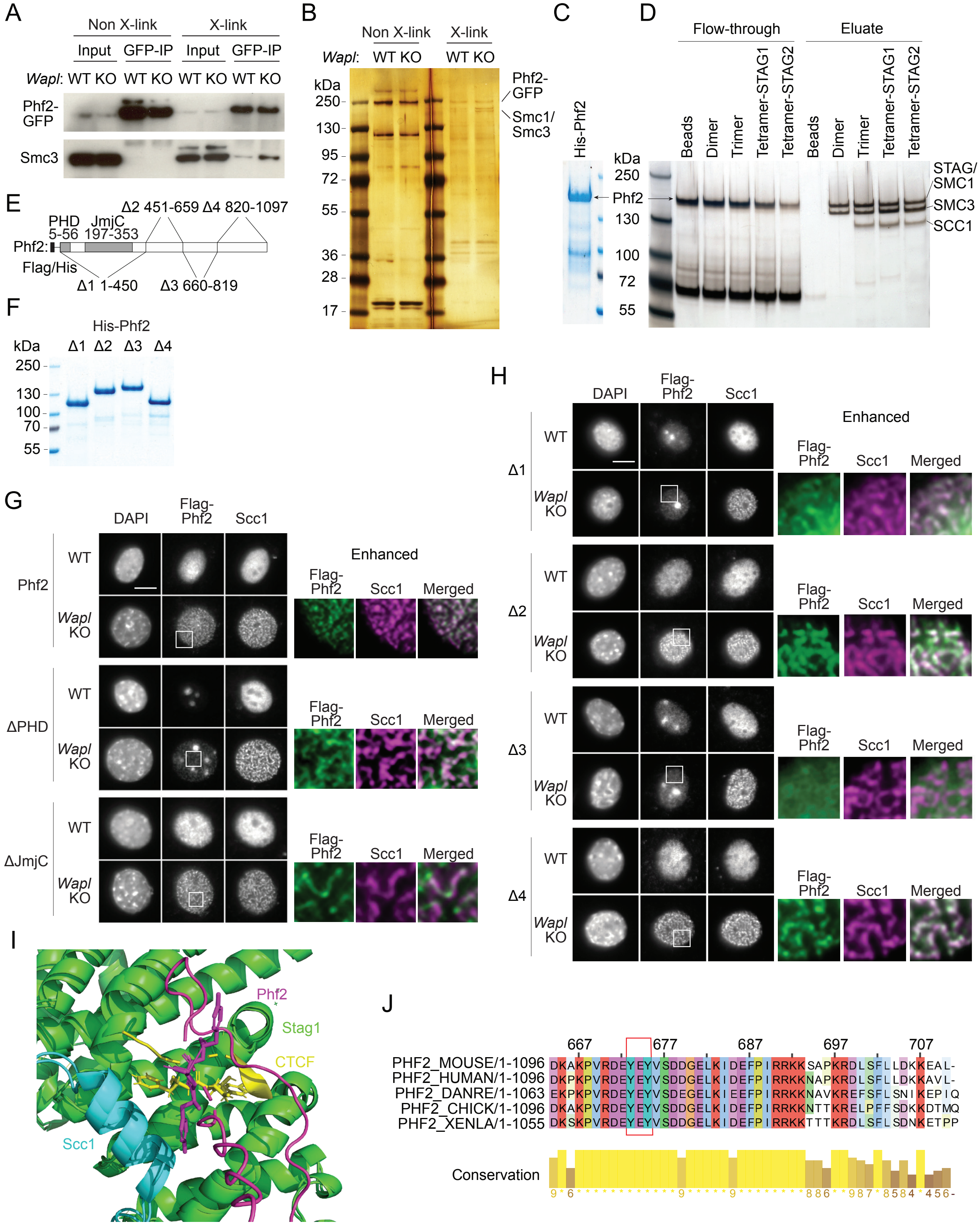

Appendix Figure S2: Phf2 residues 660-819 are essential for cohesin binding and vermicelli localization.

A. Immunoblot analysis of MEFs samples immunoprecipitated with Phf2-GFP. Immortalized WT and *Wapl* KO MEFs expressing Phf2-GFP were prepared using non X-link or X-link protocols and precipitated using an anti-GFP antibody. Proteins were detected using the indicated antibodies. Please note that a portion of this blot was used in the middle panel of Fig.1D.

B. Silver staining of immunoprecipitated samples described in A. Please note that a portion of this silver staining was used at the bottom panel of Fig.1D.

C. Coomassie blue stained SDS-PAGE gel of purified His-Phf2, indicated with a black arrow.

D. Silver staining for the *in vitro* binding assay of Phf2 with cohesin complex. Flag-beads binding human cohesin complex (Dimer, Trimer, or Tetramer) were mixed with purified Phf2 described in C.

E. Schematic representations of full-length Phf2 and its deletion mutants. PHD, plant homeodomain; JmjC, Jumonji C domain. Δ1: 1-450aa; Δ2: 451-659aa; Δ3: 660-819aa; Δ4: 820-1096aa. The Flag-tags or His-tags were fused to the N-terminus.

F. Coomassie blue stained SDS-PAGE gel of purified His-Phf2 deletion mutants.

G. Fluorescence microscopy of Phf2 mutants and Scc1 in WT and *Wapl* KO MEFs. Representative images of immortalized WT or *Wapl* KO MEFs expressing Flag-Phf2 full length, ΔPHD or ΔJmjc stained with DAPI and antibodies to Flag and Scc1. On the right-hand side, the boxed regions have been enlarged, showing Flag-Phf2 (green), Scc1 (magenta), and merged images. Scale bar, 10μm.

H. Fluorescence microscopy of Phf2 mutants and Scc1 in WT and *Wapl* KO MEFs. Representative images of immortalized WT or Wapl KO MEFs expressing Flag-Phf2 deletion mutants Δ1, Δ2, Δ3, or Δ4 stained with DAPI and antibodies to Flag and Scc1. Scale bar, 10μm. On the right-hand side, the boxed regions have been enlarged, showing Flag-Phf2 (green), Scc1 (magenta), and merged images. Scale bar, 10μm.

I. Structural model for the Stag1/Scc1 CES pocket bound to CTCF and Phf2. The crystal structure for Stag1 (green), Scc1 (light blue), and CTCF (yellow) was aligned with an aFold2 model for the interaction between Stag1/Scc1 and Phf2 (purple).

J. Protein sequence alignment for the predicted Phf2 fragment that binds the Stag1/Scc1 CES pocket from different model organisms. The conserved YEY motif is boxed in red.

Appendix Figure S3

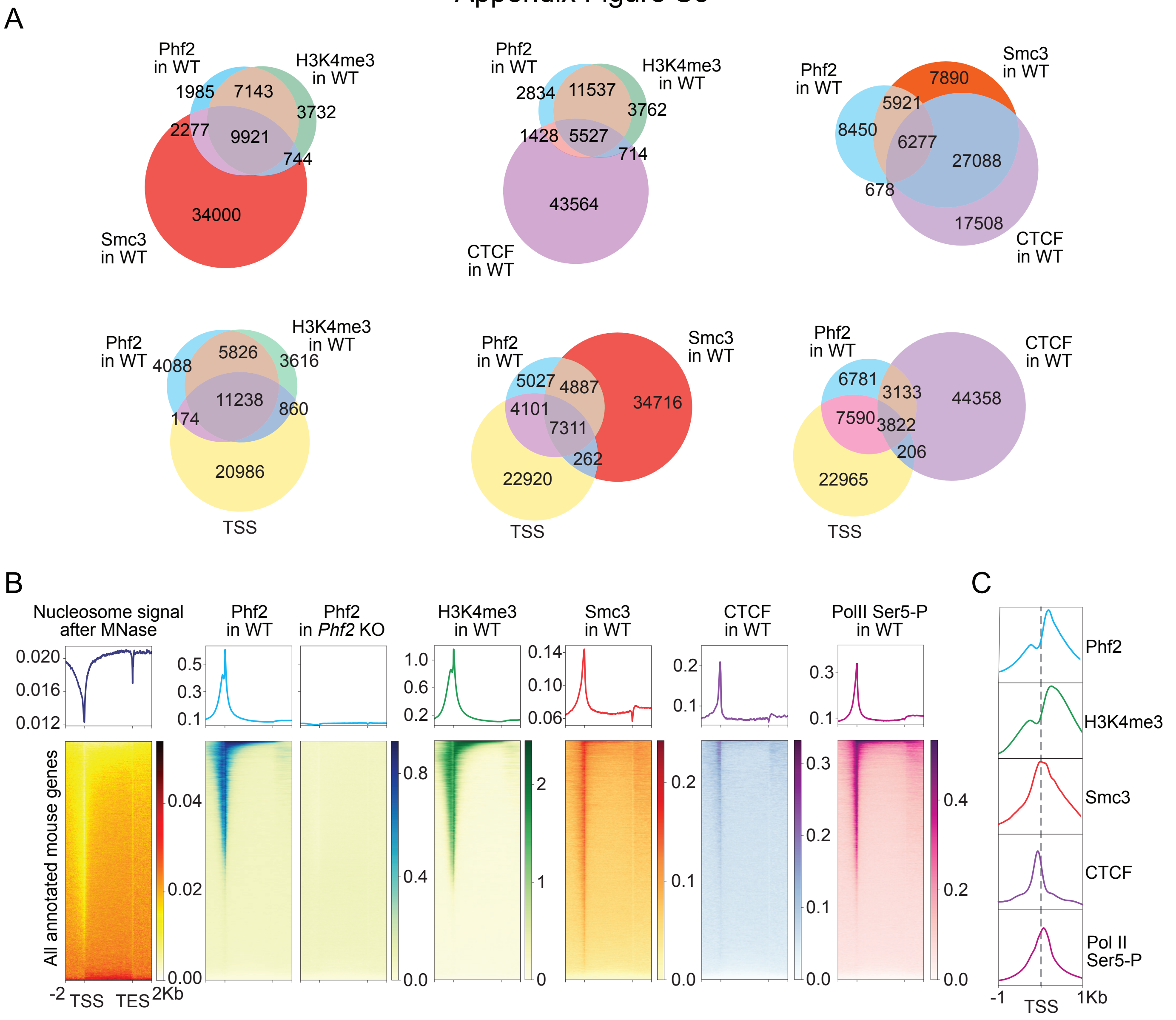

Appendix Figure S3: Phf2 co-localizes with H3K4me3 and cohesin genome-wide.

A. Venn diagram showing the overlap between ChIP-seq peaks for Phf2, H3K4me3, Smc3, CTCF, and transcription start sites (TSS).

B. Pile-up heat maps and summary plots of ChIP-seq signals for nucleosome occupancy, binding of Phf2 (in WT and *Phf2* KO), H3K4me3, Smc3, CTCF, and RNA polymerase II phosphorylated at Ser5 (PolII Ser5-P) at all annotated mouse genes from transcription start sites (TSS) to transcription end sited (TES) (genes were stretched to equal size).

C. Summary plots of ChIP-seq of Phf2, H3K4me3, Smc3, CTCF, and PolII Ser5-P at annotated mouse genes transcription start sites (TSS).

Appendix Figure S4

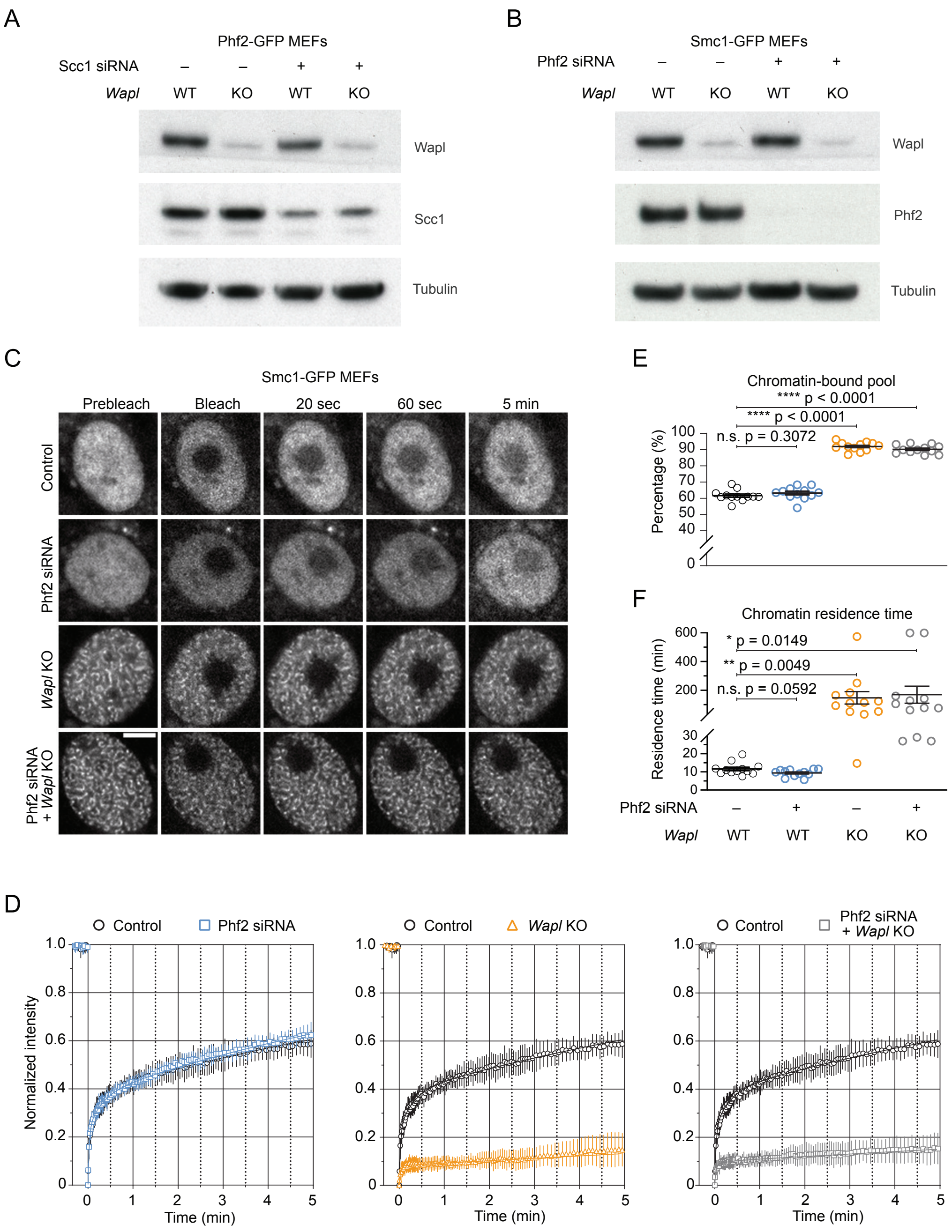

Appendix Figure S4: Phf2 does not influence cohesin chromatin association.

A. Immunoblot analysis of whole-cell extracts from control, *Wapl* KO, Scc1 depleted by siRNA, and Scc1 depleted in combination with Wapl KO MEFs were analyzed using the indicated antibodies.
B. Immunoblot analysis of whole-cell extracts from control, *Wapl* KO, Phf2 depleted by siRNA, and Phf2 depleted in combination with Wapl KO MEFs were analyzed using the indicated antibodies.
C. Microscopy images of MEFs expressing Smc1-GFP were analyzed by fluorescence recovery after photobleaching (FRAP) of an area with 2µm diameter. Phf2 was depleted by siRNA transfection in either control or *Wapl* KO MEFs. Scale bar, 2µm.
D. Quantification of Smc1-GFP fluorescence intensity in the bleached area over time, shown for 12 cells per condition as described in C. Number of cells used n=12. Error bars indicate the Standard Error of the Mean.
E. Quantification of the chromatin bound fraction of Smc1-GFP calculated from curves in D. Statistical analyses were performed with unpaired t test and the *P* values are indicated in the figure. Error bars indicate the Standard Error of the Mean.

F. Quantification of the residence time on chromatin of Smc1-GFP calculated from curves in D. Statistical analyses were performed with unpaired t test and the *P* values are indicated in the figure. Error bars indicate the Standard Error of the Mean.

Appendix Figure S5

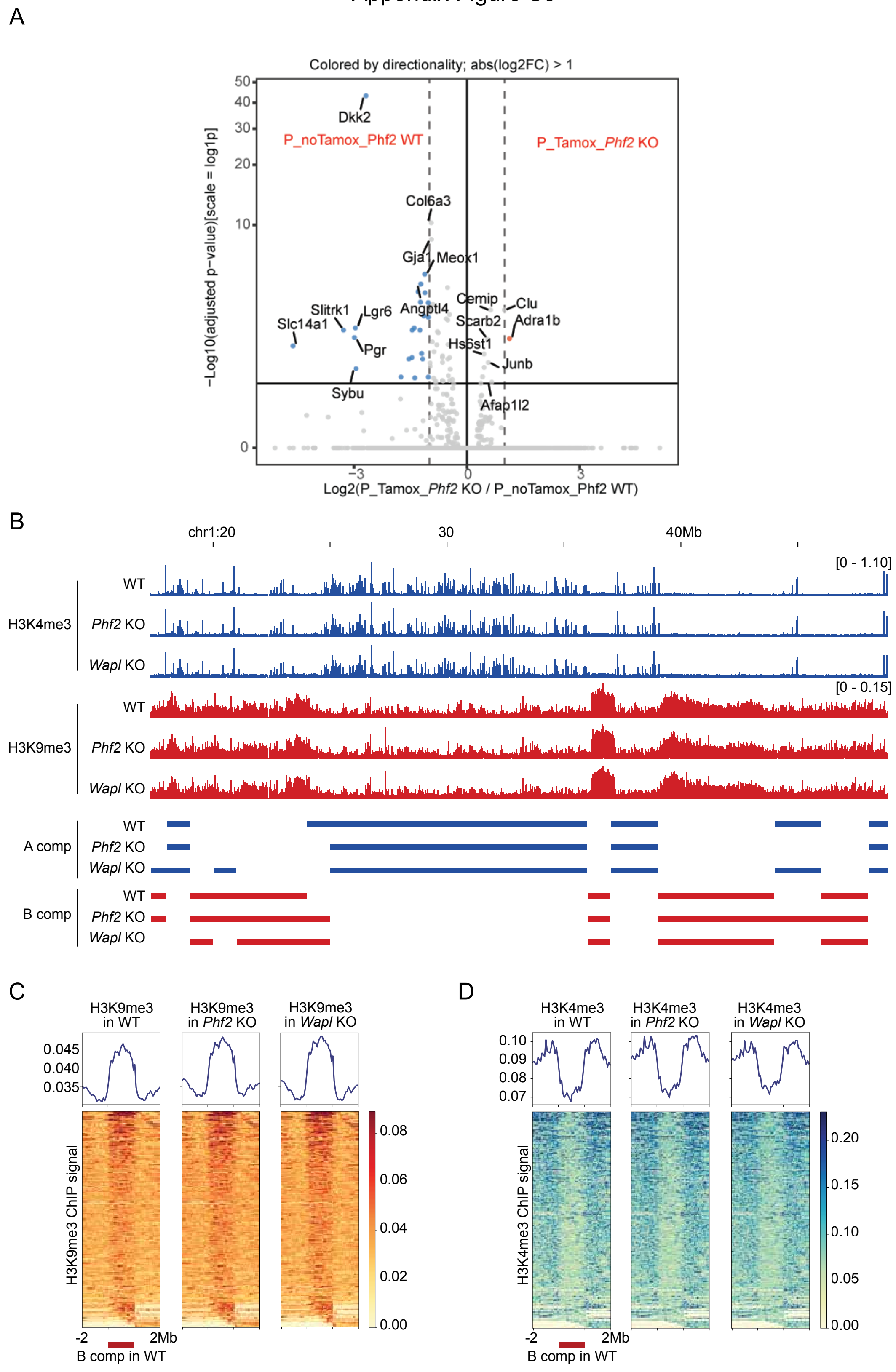

Appendix Figure S5: Phf2 depletion limits the length of heterochromatic B compartments.

A. Volcano plot depicting the differential expression of genes from WT (left) and *Phf2* KO MEFs (right) as measured by RNA-seq. Y-axis denotes − log10 P values while X-axis shows log2 fold change values. Adjusted P-value was calculated using DESeq2 tool. Biological replicates n=2.

B. Binding of H3K4me3 (in WT, *Phf2* KO, and *Wapl* KO), H3K9me3 (in WT, Phf2 KO, and *Wapl* KO) at representative locus determined by ChIP-seq (top). A and B compartments identified in WT, *Phf2* KO, and *Wapl* KO were depicted with blue and red bars respectively.

C. Pile-up heat maps and summary plots of ChIP-seq signals obtained for H3K9me3 (WT, *Phf2* KO, and *Wapl* KO) at B compartments (all B compartments were stretched to the same size).

D. Pile-up heat maps and summary plots of ChIP-seq signals obtained for H3K4me3 (WT, *Phf2* KO, and Wapl KO) at B compartments (all B compartments were stretched to the same size).

Appendix Figure S6

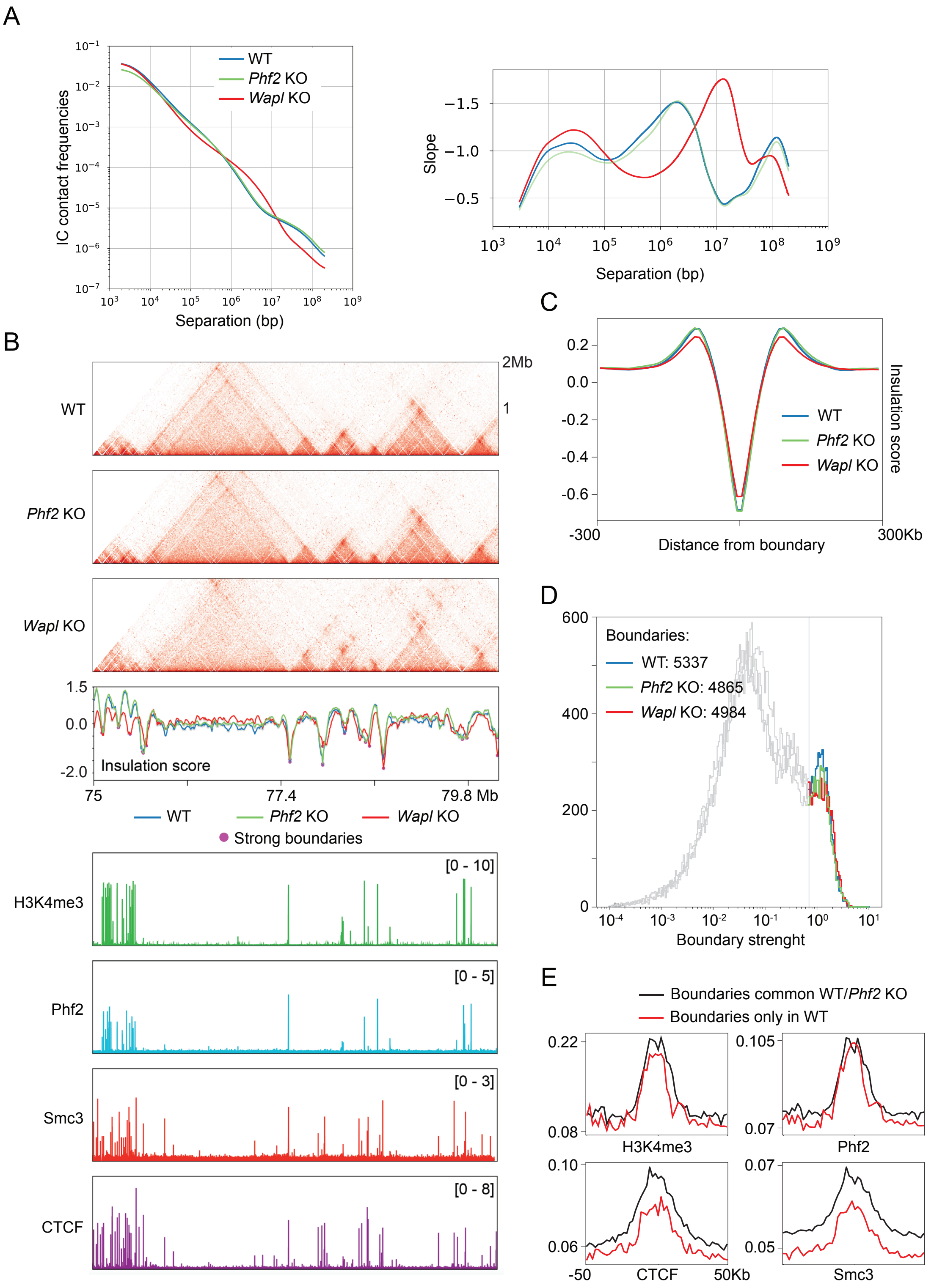

Appendix Figure S6: Phf2 depletion negatively affects a subset of TAD boundaries characterized by low levels of CTCF.

A. Intra-chromosomal contact frequency distribution as a function of genomic distance in WT, *Phf2* KO, and *Wapl* KO MEFs.

B. Topologically associating domain (TAD) boundaries from Hi-C experiments. Contact matrix (top), insulation score (middle) from Hi-C experiments performed in WT, *Phf2* KO, and *Wapl* KO MEFs. Strong boundaries were defined by strength values bigger than 0.7 (magenta circles). Binding of H3K4me3, Phf2, Smc3, and CTCF in WT MEFs was depicted with ChIP-seq profile (bottom).

C. Summary plots for the total insulation score at TAD boundaries identified in WT was shown for WT, *Phf2* KO, and *Wapl* KO MEFs.

D. Column plot indicating the number of TAD boundaries in WT, *Phf2* KO, and *Wapl* KO. Only strong boundaries as defined in B were counted and colored.

E. Summary plots of ChIP-seq signals obtained for H3K4me3, Phf2, Smc3, and CTCF at TAD boundaries. Strong TAD boundaries identified in WT were divided into two groups, the ones that are also found in *Phf2* KO (Boundaries common WT/*Phf2* KO), and the ones that are not found in *Phf2* KO (Boundaries only in WT).
