# Supplementary material for: Cohesin positions the epigenetic reader Phf2 within the genome
Source: EMBO J. 2025 Jan 2;44(3):736–66. doi: 10.1038/s44318-024-00348-2 (PMC11790891; doi:10.1038/s44318-024-00348-2)
Supplement: Supplementary file 5 — Source data Fig. 1 [file 44318_2024_348_MOESM5_ESM.zip › Figure 1/1A/PRIDE.rtf]

Reviewer access details
Log in to the PRIDE website using the following:
·	Project accession: PXD057926
·	Token: rxSzUc41hkJD
Alternatively, reviewers can use this login information:
·	Username: reviewer_pxd057926@ebi.ac.uk
·	Password: PobweepguhfD
