# Supplementary figures and images for: Cohesin positions the epigenetic reader Phf2 within the genome (part 1 of 3)
Source: EMBO J. 2025 Jan 2;44(3):736–66. doi: 10.1038/s44318-024-00348-2 (PMC11790891; doi:10.1038/s44318-024-00348-2)

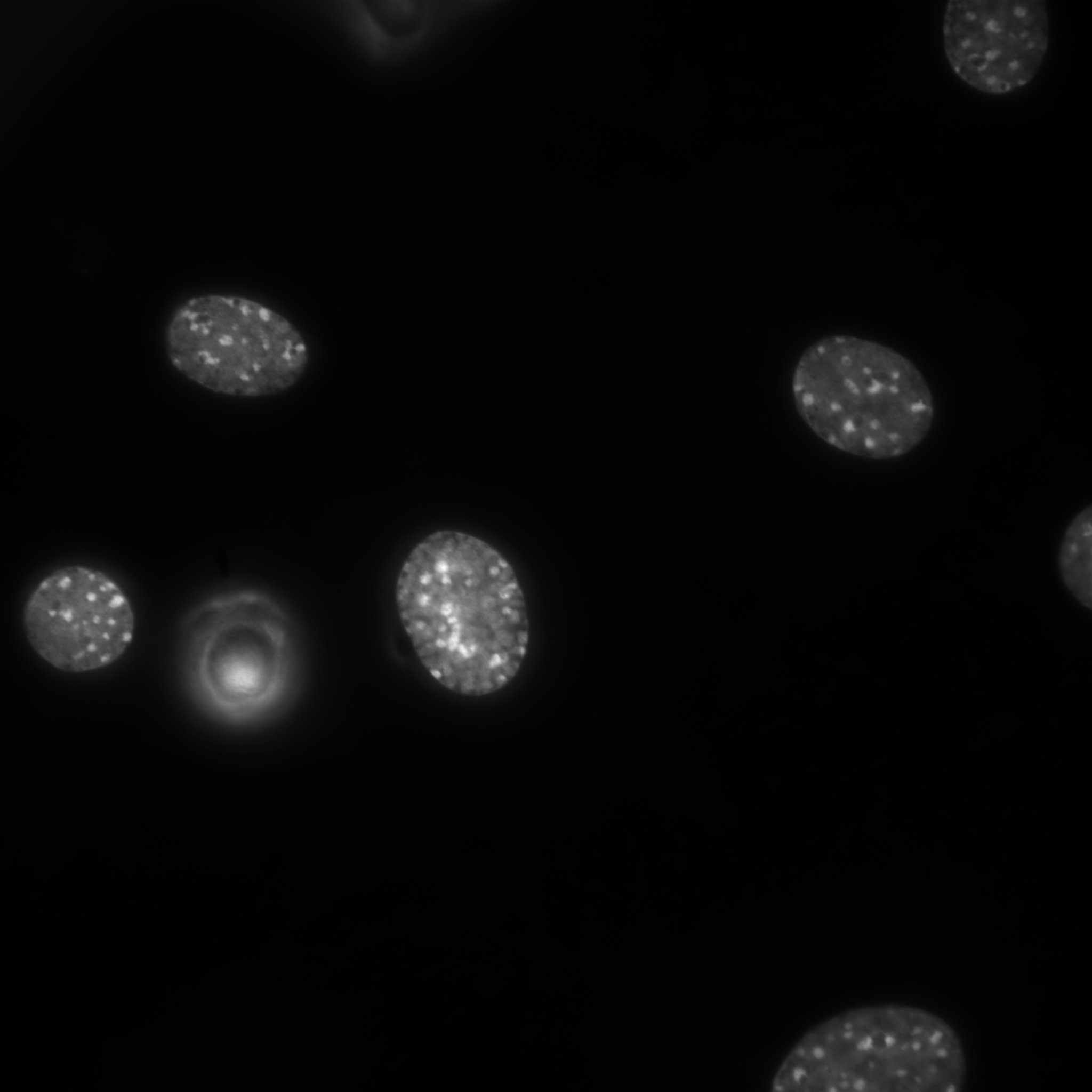

Supplement: Supplementary file 5 — Source data Fig. 1 [file 44318_2024_348_MOESM5_ESM.zip › Figure 1/1B/Immunofluorescence DAPI in Wapl KO.jpg]

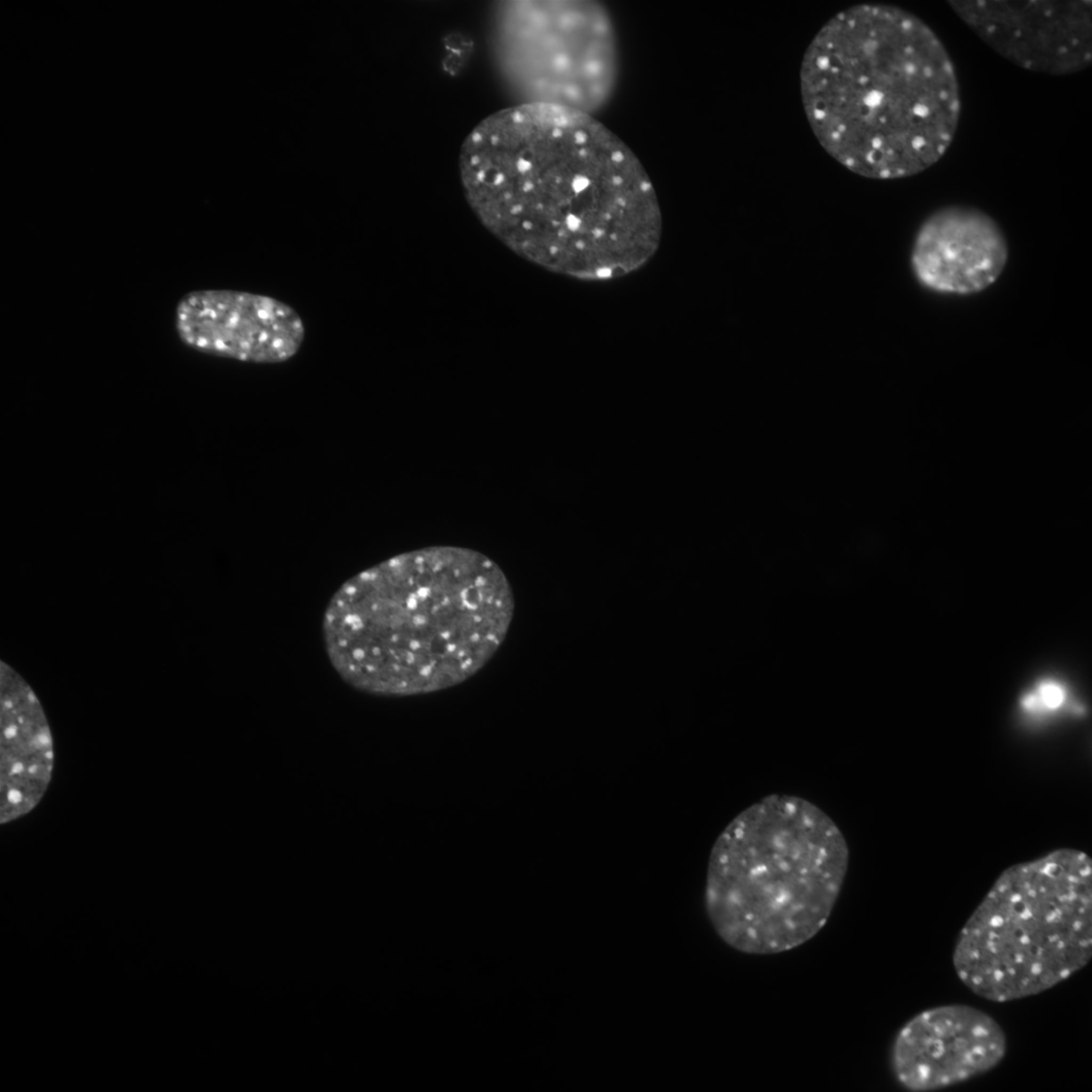

Supplement: Supplementary file 5 — Source data Fig. 1 [file 44318_2024_348_MOESM5_ESM.zip › Figure 1/1B/Immunofluorescence DAPI in WT.jpg]

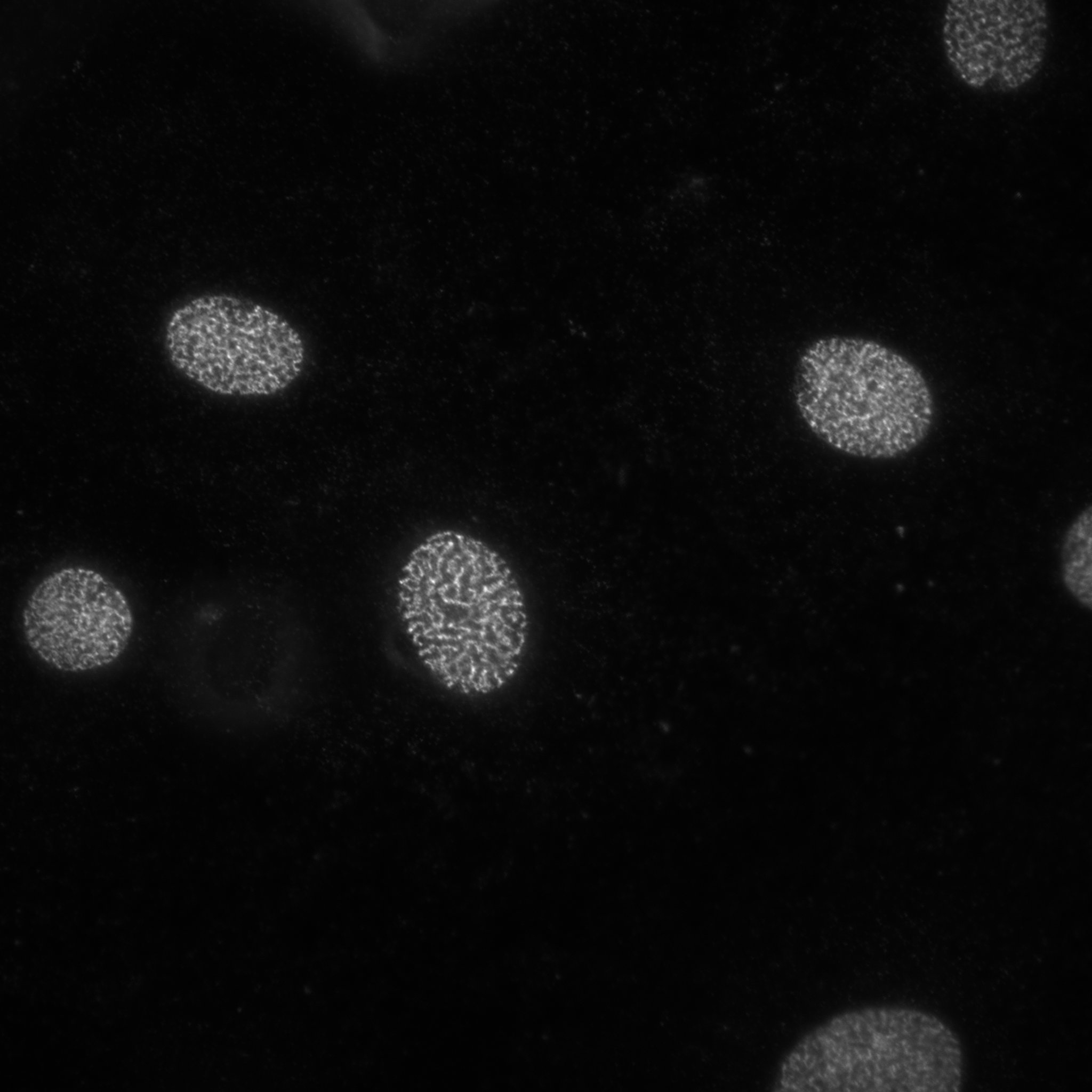

Supplement: Supplementary file 5 — Source data Fig. 1 [file 44318_2024_348_MOESM5_ESM.zip › Figure 1/1B/Immunofluorescence Phf2 in Wapl KO.jpg]

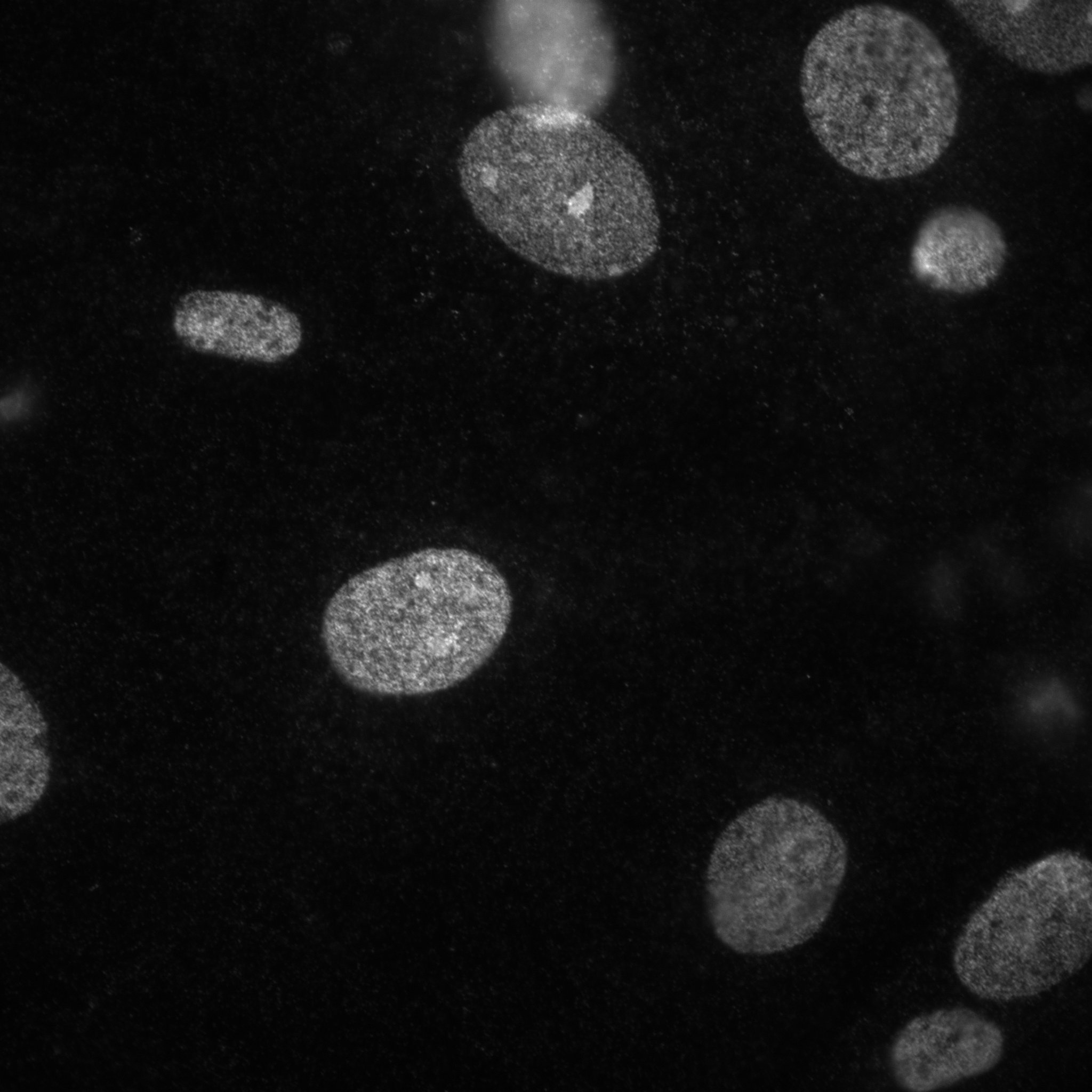

Supplement: Supplementary file 5 — Source data Fig. 1 [file 44318_2024_348_MOESM5_ESM.zip › Figure 1/1B/Immunofluorescence Phf2 in WT.jpg]

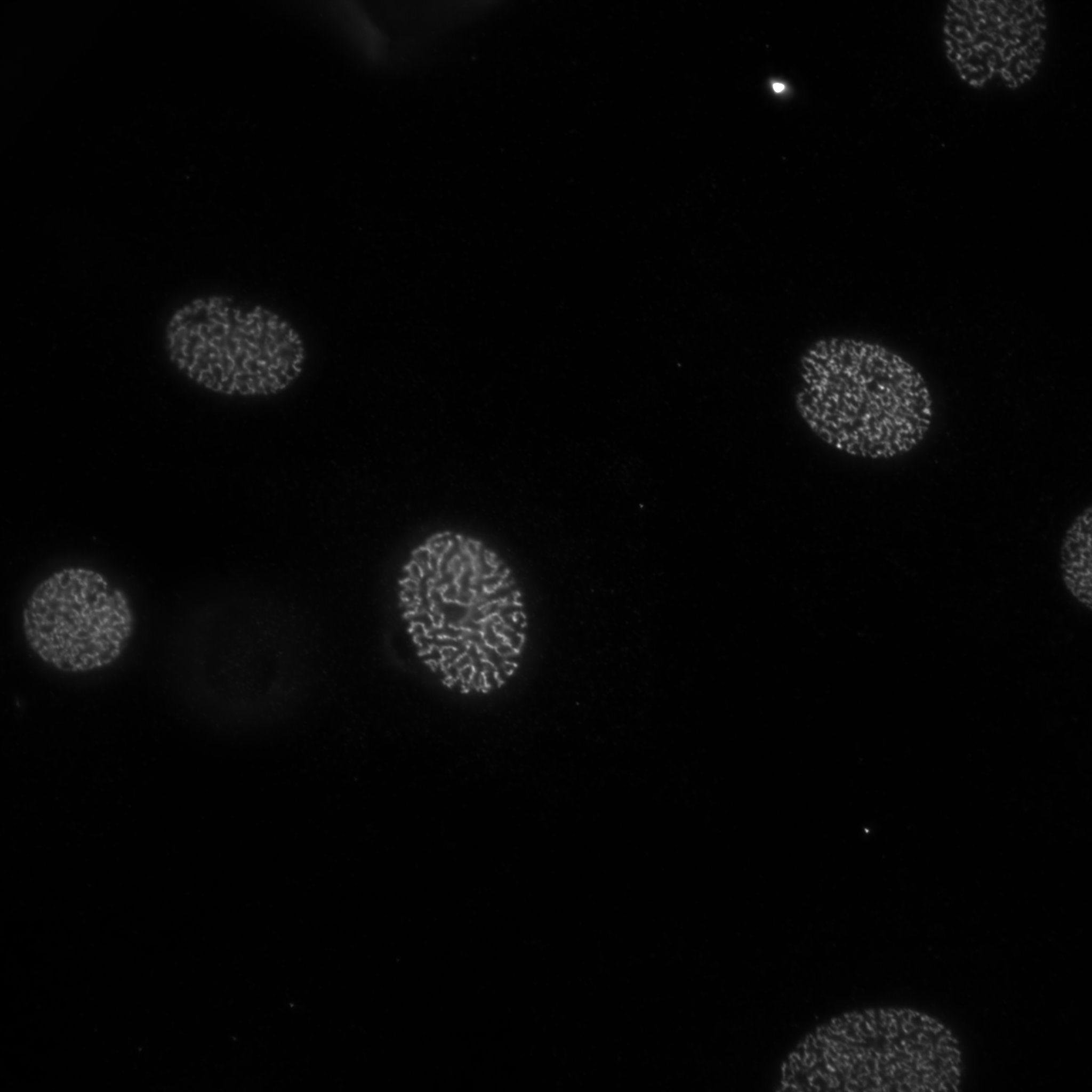

Supplement: Supplementary file 5 — Source data Fig. 1 [file 44318_2024_348_MOESM5_ESM.zip › Figure 1/1B/Immunofluorescence Scc1 in Wapl KO.jpg]

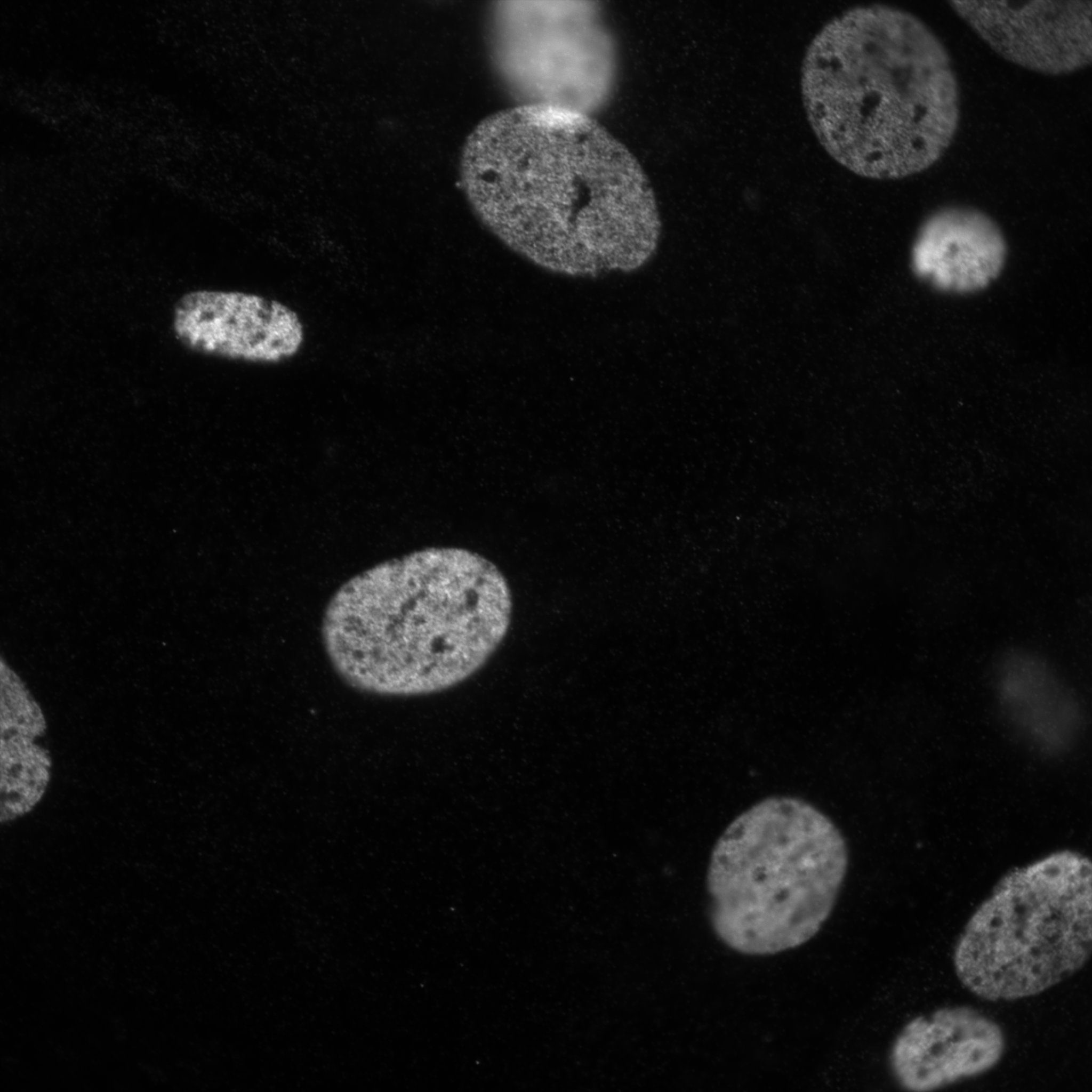

Supplement: Supplementary file 5 — Source data Fig. 1 [file 44318_2024_348_MOESM5_ESM.zip › Figure 1/1B/Immunofluorescence Scc1 in WT.jpg]

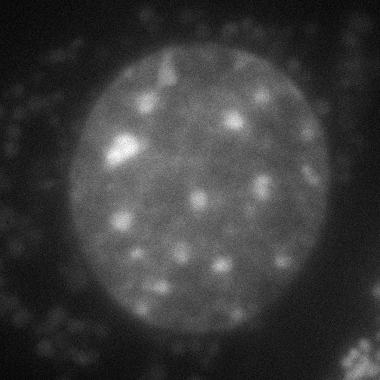

Supplement: Supplementary file 5 — Source data Fig. 1 [file 44318_2024_348_MOESM5_ESM.zip › Figure 1/1C/live cell imaging Phf2-GFP in Wapl KO.tif]

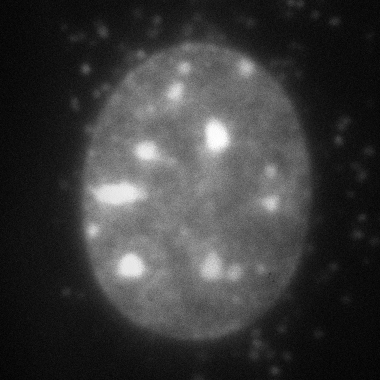

Supplement: Supplementary file 5 — Source data Fig. 1 [file 44318_2024_348_MOESM5_ESM.zip › Figure 1/1C/live cell imaging Phf2-GFP in WT.tif]

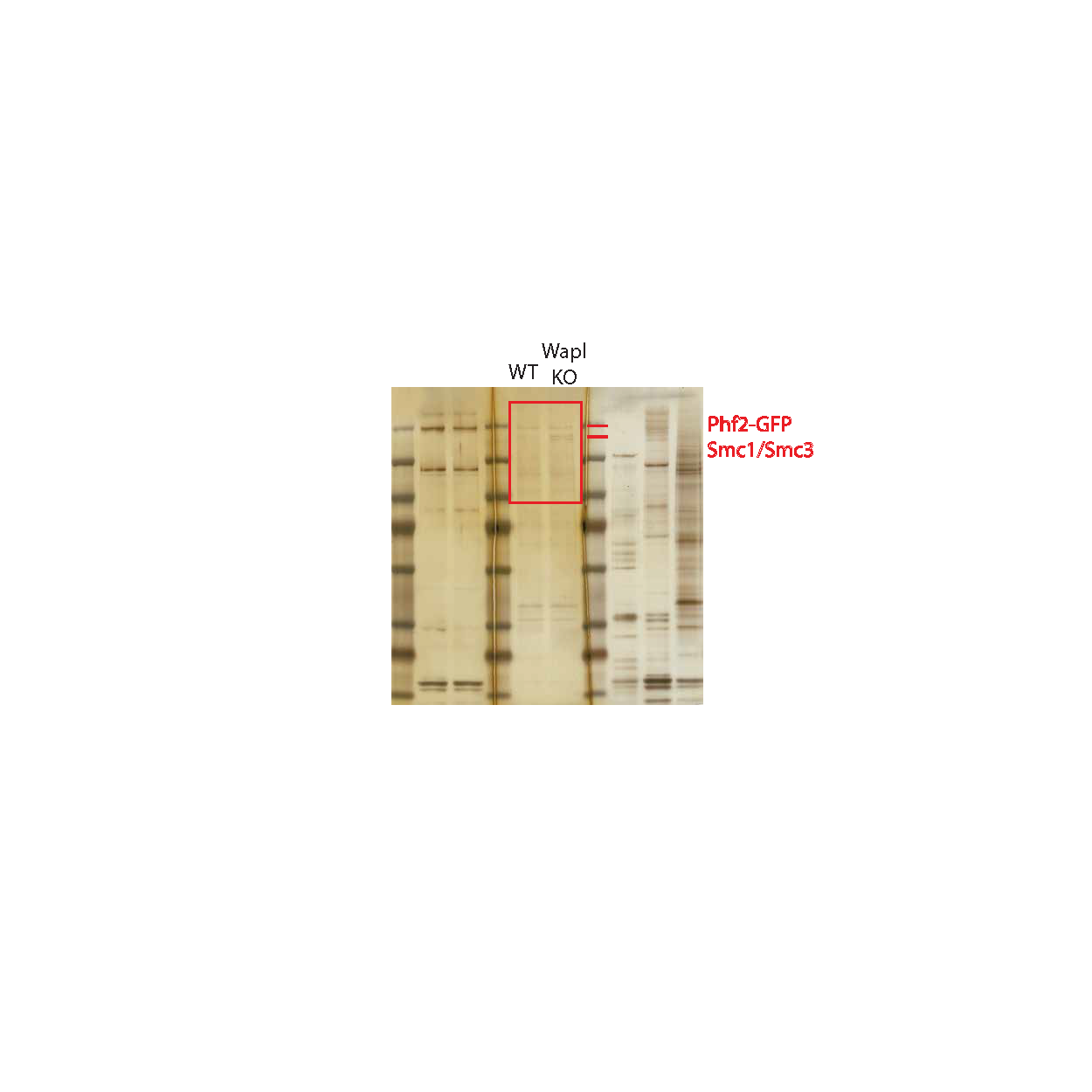

Supplement: Supplementary file 5 — Source data Fig. 1 [file 44318_2024_348_MOESM5_ESM.zip › Figure 1/1D/silver gel Fig 1D-bottom panel.tiff]

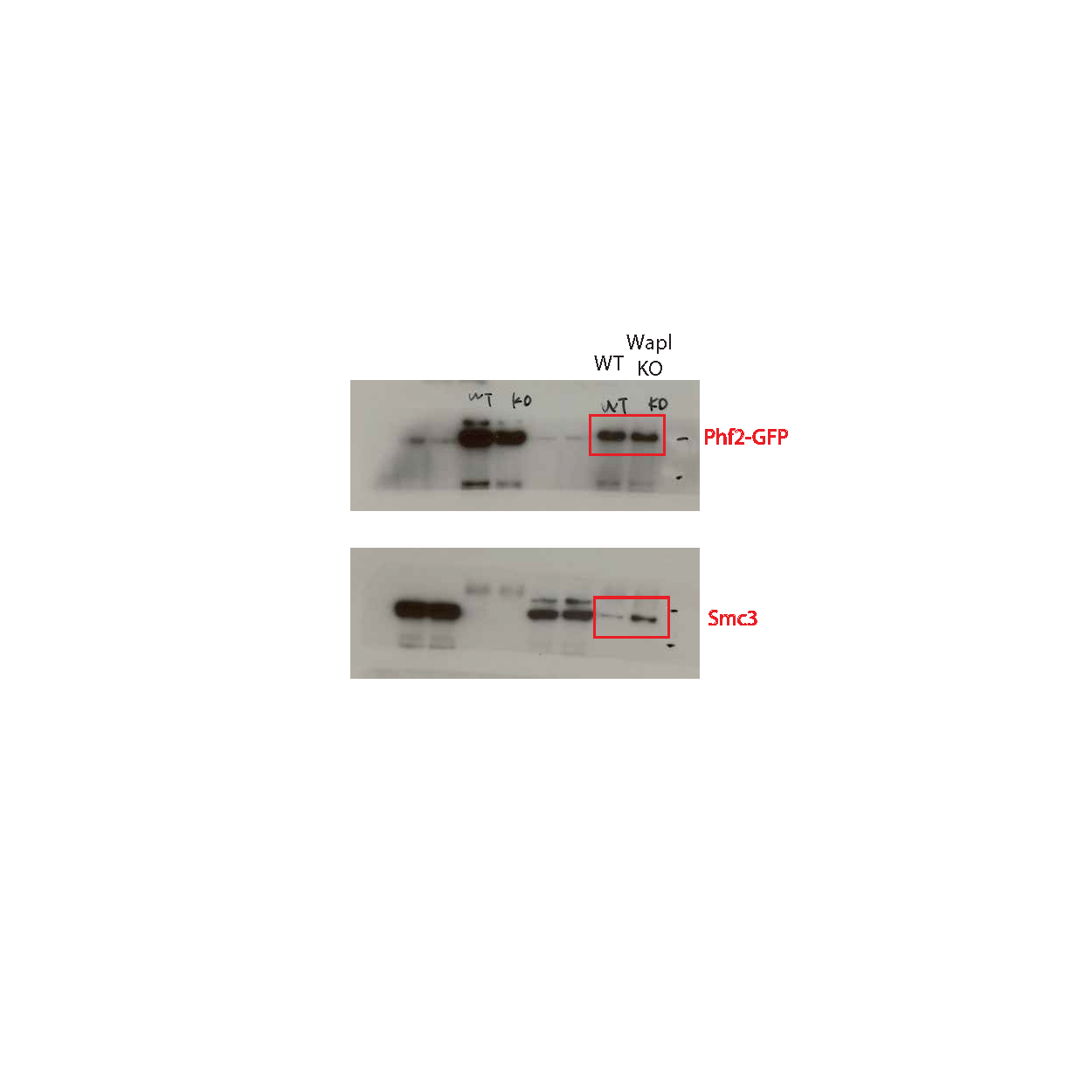

Supplement: Supplementary file 5 — Source data Fig. 1 [file 44318_2024_348_MOESM5_ESM.zip › Figure 1/1D/western Fig 1D-middle panel.tiff]

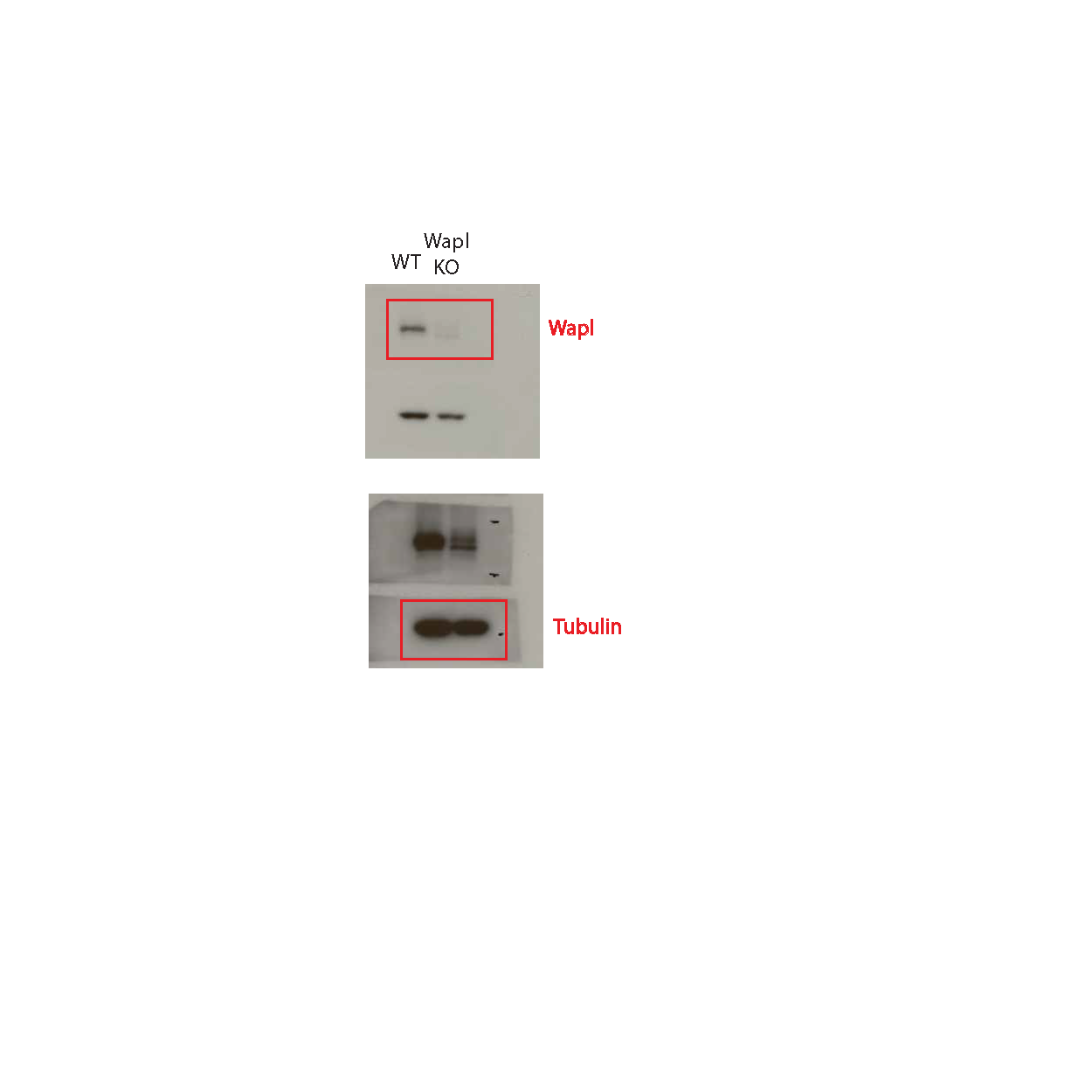

Supplement: Supplementary file 5 — Source data Fig. 1 [file 44318_2024_348_MOESM5_ESM.zip › Figure 1/1D/western Fig 1D-top panel.tiff]

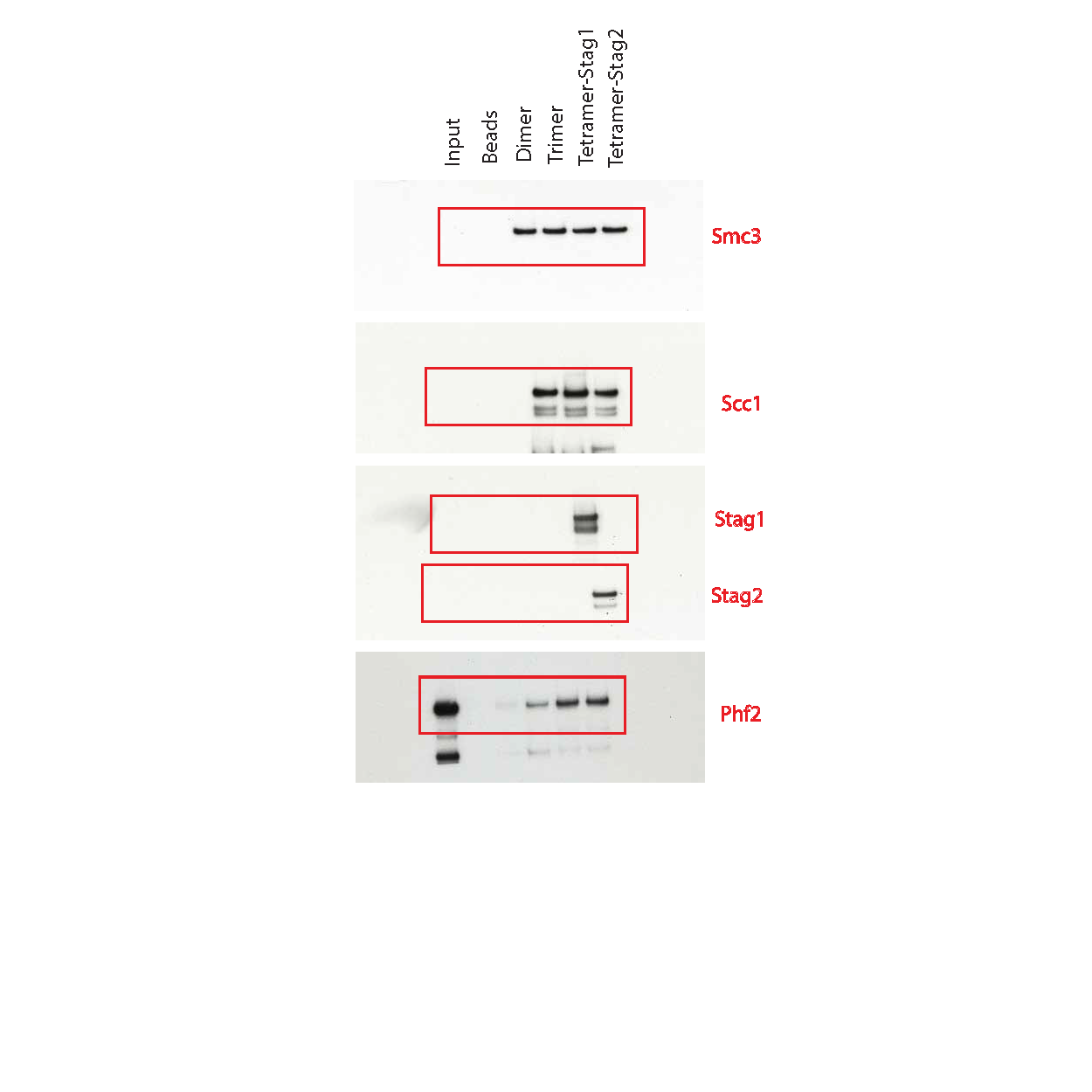

Supplement: Supplementary file 5 — Source data Fig. 1 [file 44318_2024_348_MOESM5_ESM.zip › Figure 1/1E/western Fig 1E.tiff]

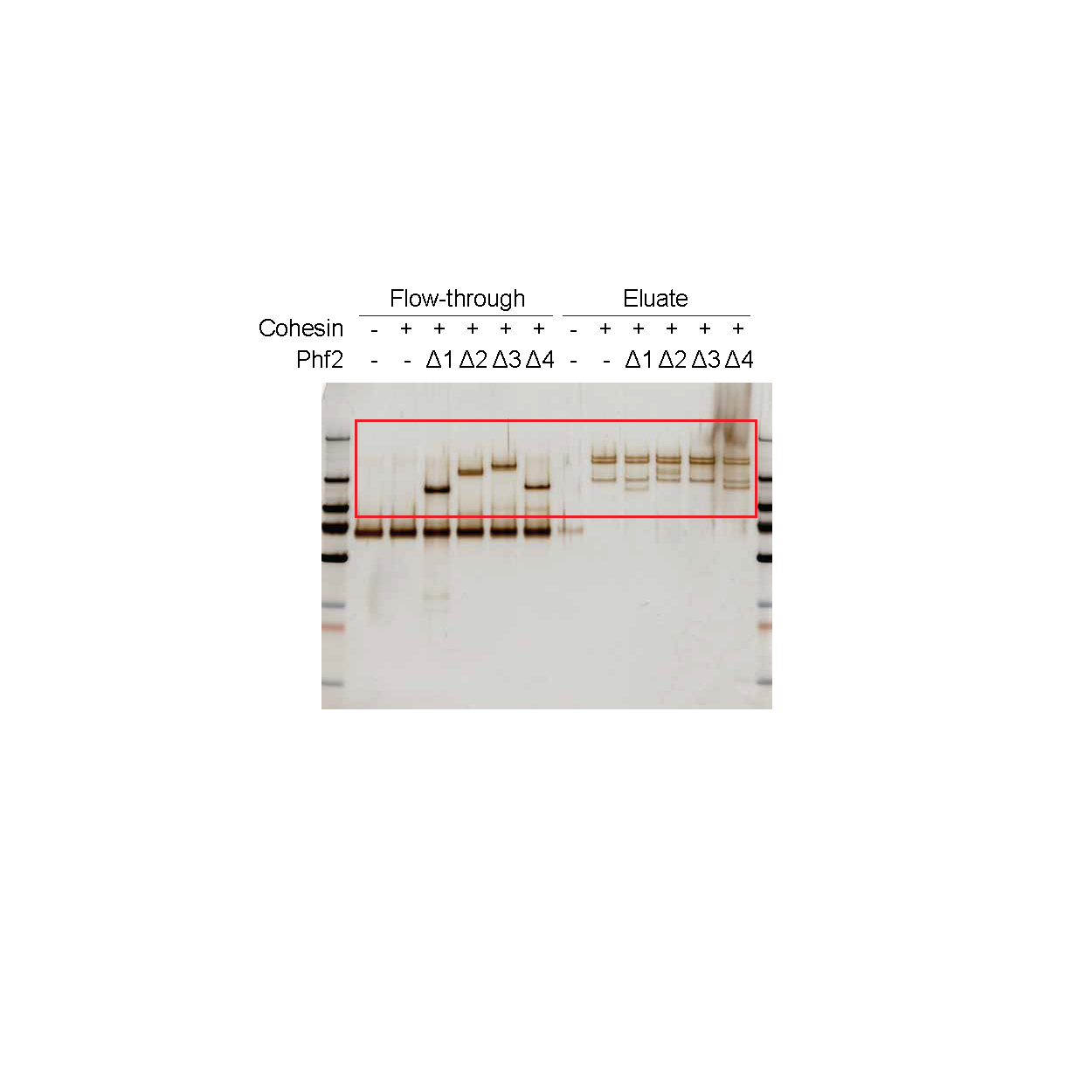

Supplement: Supplementary file 5 — Source data Fig. 1 [file 44318_2024_348_MOESM5_ESM.zip › Figure 1/1G/silver gel Fig 1G.tiff]

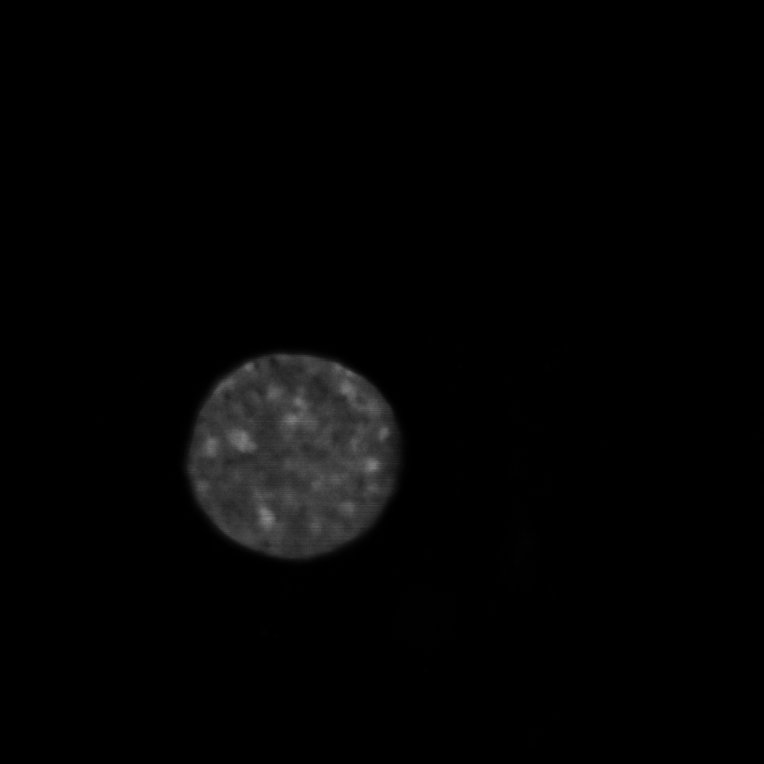

Supplement: Supplementary file 5 — Source data Fig. 1 [file 44318_2024_348_MOESM5_ESM.zip › Figure 1/1I/Immunofluorescence DAPI- Del 673-711 in Wapl KO.jpg]

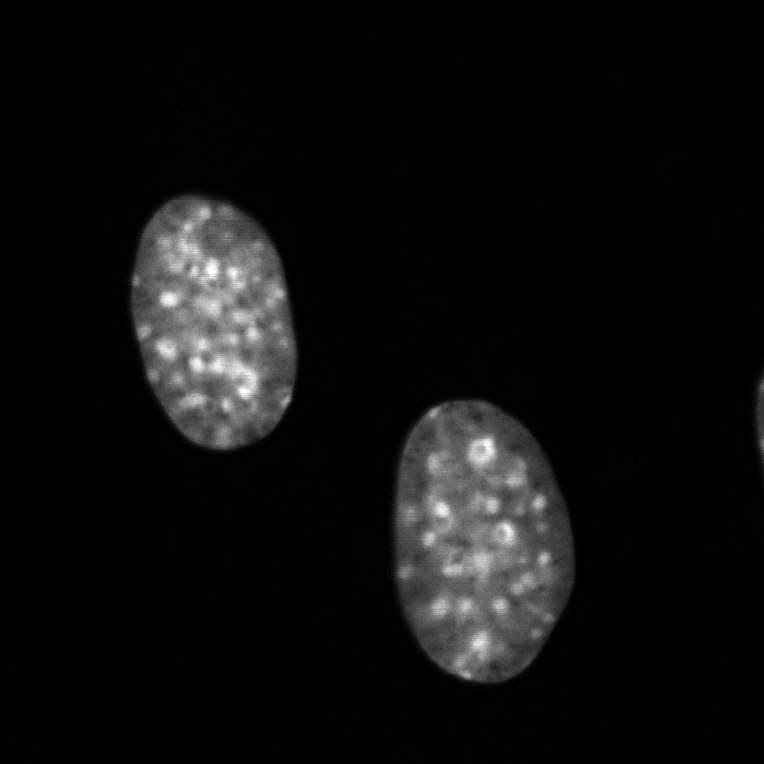

Supplement: Supplementary file 5 — Source data Fig. 1 [file 44318_2024_348_MOESM5_ESM.zip › Figure 1/1I/Immunofluorescence DAPI-Del 673-711 in WT.jpg]

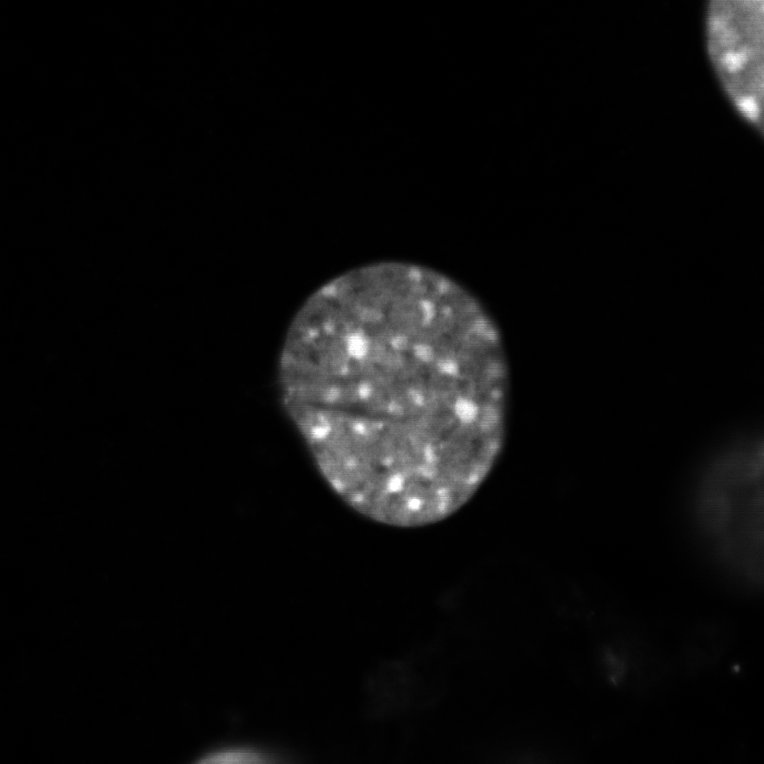

Supplement: Supplementary file 5 — Source data Fig. 1 [file 44318_2024_348_MOESM5_ESM.zip › Figure 1/1I/Immunofluorescence DAPI-wildtype Phf2 in Wapl KO.jpg]

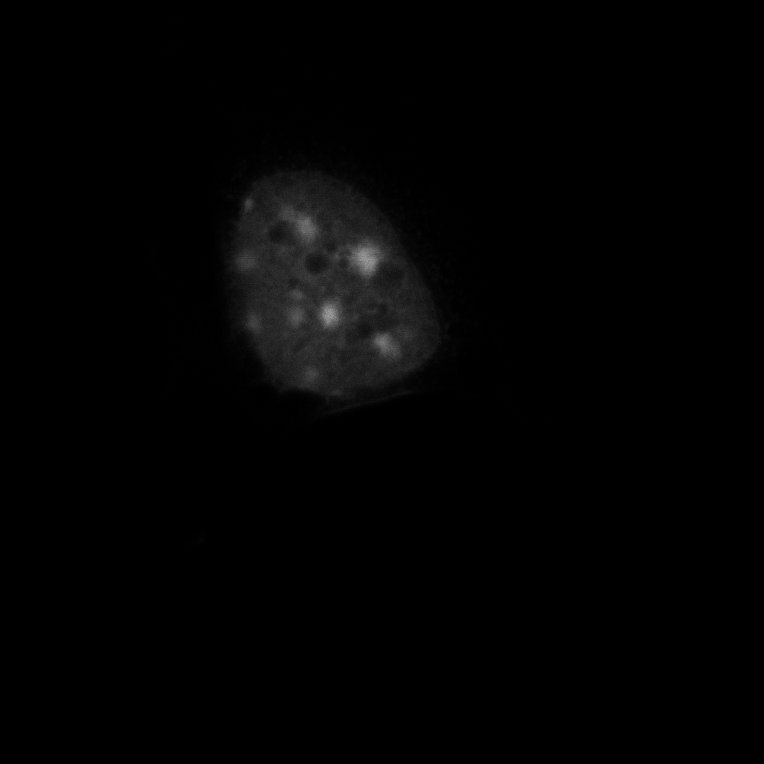

Supplement: Supplementary file 5 — Source data Fig. 1 [file 44318_2024_348_MOESM5_ESM.zip › Figure 1/1I/Immunofluorescence DAPI-wildtype Phf2 in WT.jpg]

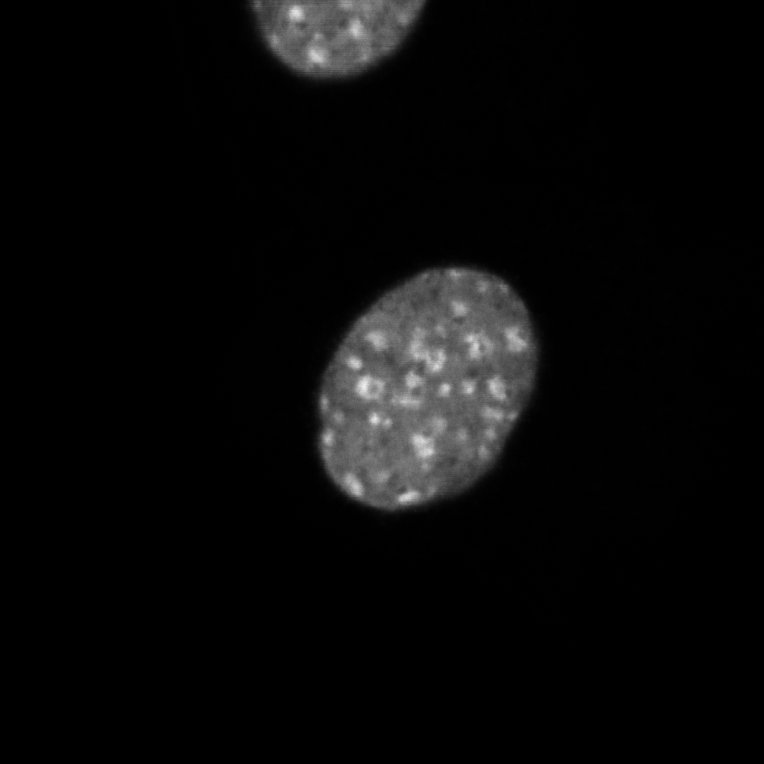

Supplement: Supplementary file 5 — Source data Fig. 1 [file 44318_2024_348_MOESM5_ESM.zip › Figure 1/1I/Immunofluorescence DAPI-Y673A Y675A in Wapl KO.jpg]

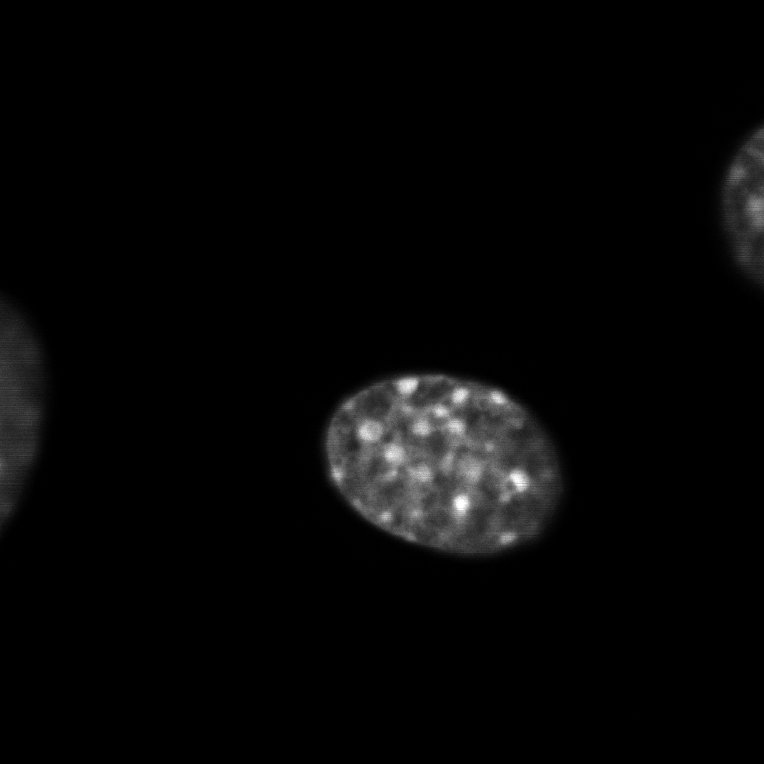

Supplement: Supplementary file 5 — Source data Fig. 1 [file 44318_2024_348_MOESM5_ESM.zip › Figure 1/1I/Immunofluorescence DAPI-Y673A Y675A in WT.jpg]

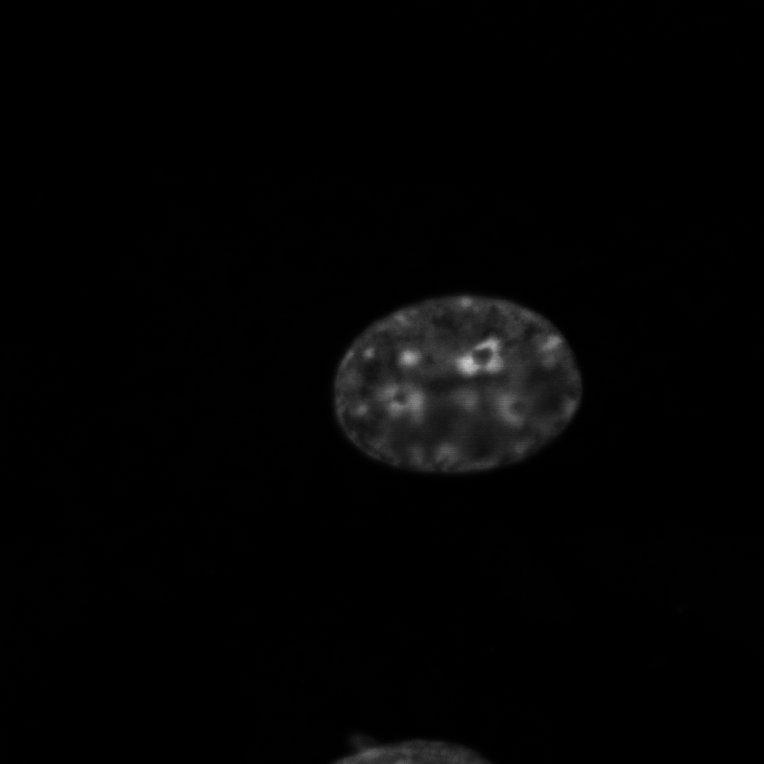

Supplement: Supplementary file 5 — Source data Fig. 1 [file 44318_2024_348_MOESM5_ESM.zip › Figure 1/1I/Immunofluorescence DAPI-Y869A Y871A in Wapl KO.jpg]

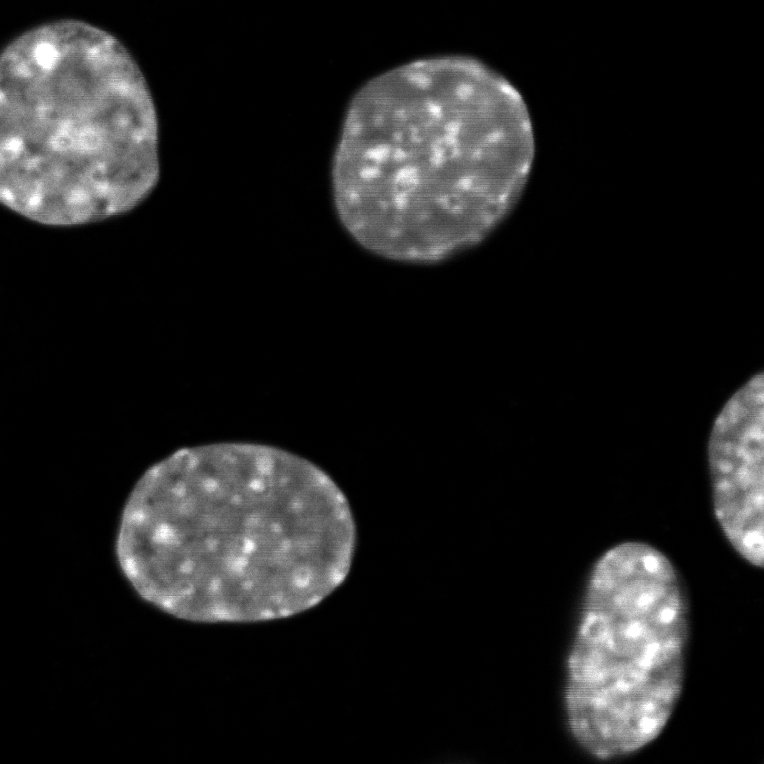

Supplement: Supplementary file 5 — Source data Fig. 1 [file 44318_2024_348_MOESM5_ESM.zip › Figure 1/1I/Immunofluorescence DAPI-Y869A Y871A in WT.jpg]

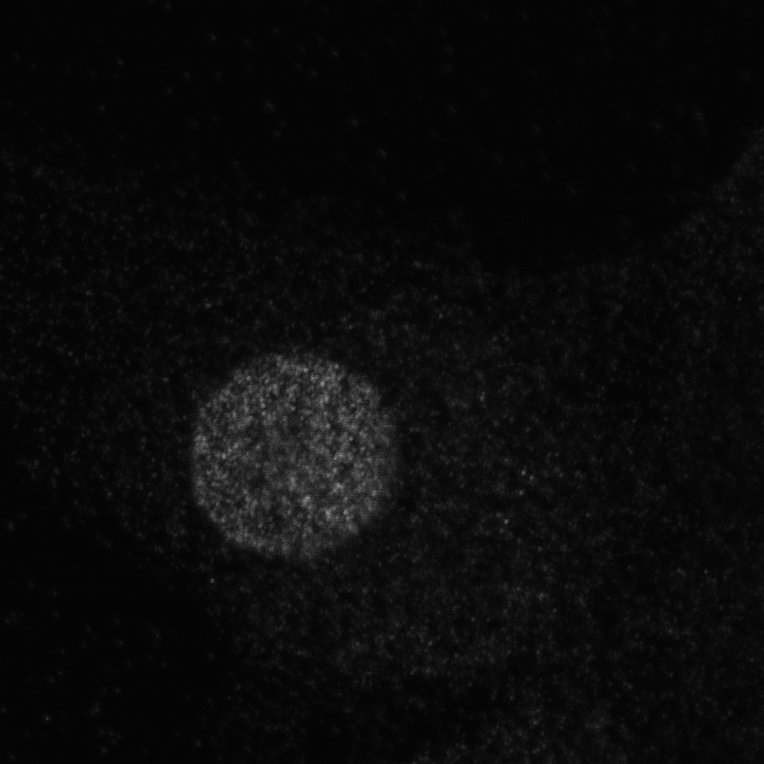

Supplement: Supplementary file 5 — Source data Fig. 1 [file 44318_2024_348_MOESM5_ESM.zip › Figure 1/1I/Immunofluorescence FLAG-Del 673-711 in Wapl KO.jpg]

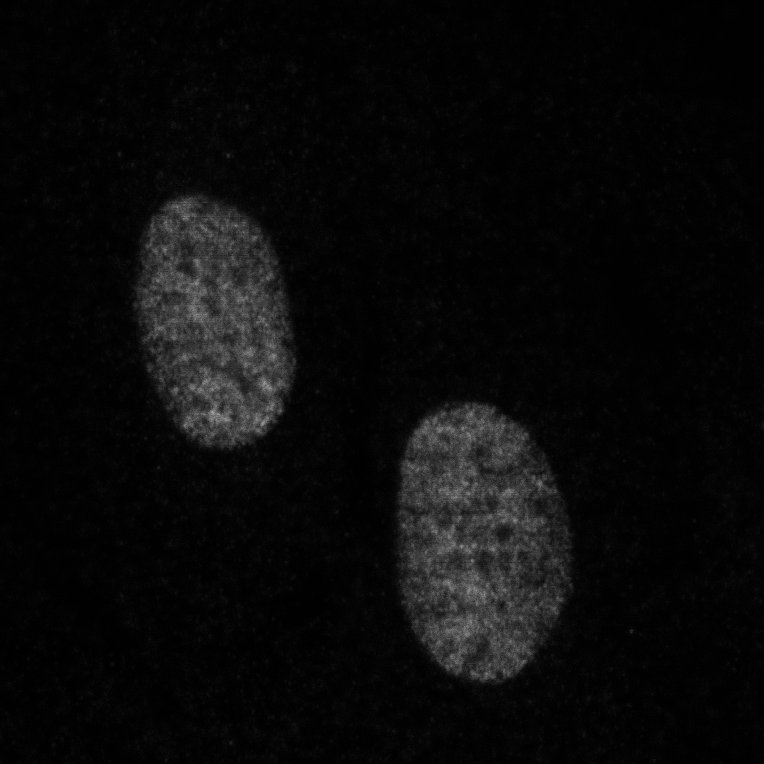

Supplement: Supplementary file 5 — Source data Fig. 1 [file 44318_2024_348_MOESM5_ESM.zip › Figure 1/1I/Immunofluorescence FLAG-Del 673-711 in WT.jpg]

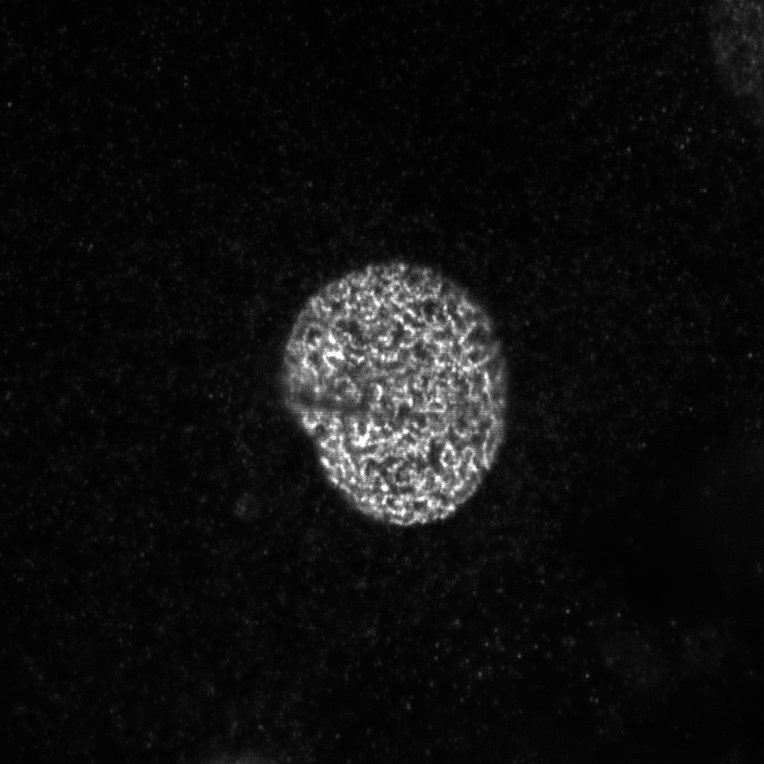

Supplement: Supplementary file 5 — Source data Fig. 1 [file 44318_2024_348_MOESM5_ESM.zip › Figure 1/1I/Immunofluorescence FLAG-wildtype Phf2 in Wapl KO.jpg]

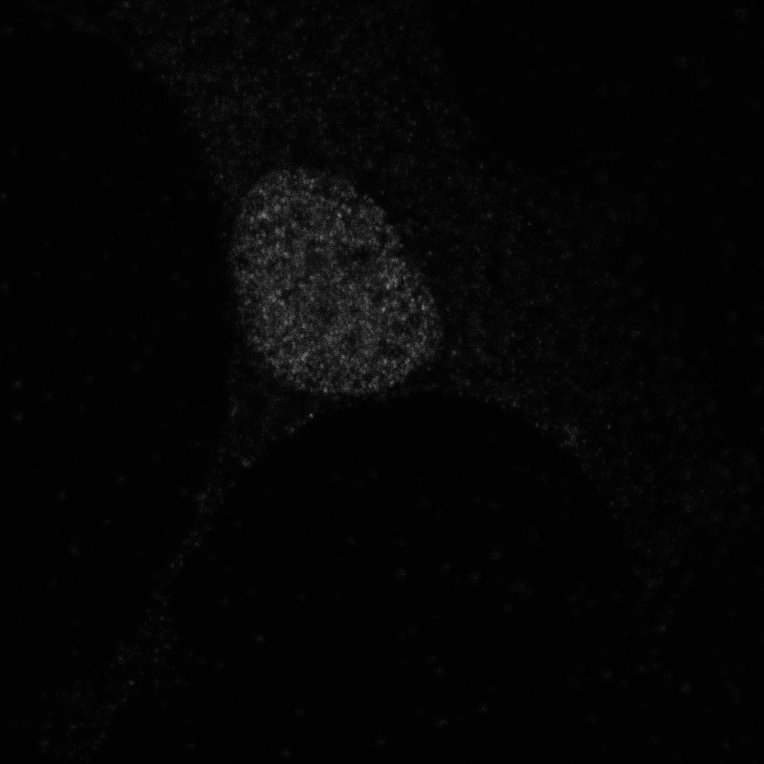

Supplement: Supplementary file 5 — Source data Fig. 1 [file 44318_2024_348_MOESM5_ESM.zip › Figure 1/1I/Immunofluorescence FLAG-wildtype Phf2 in WT.jpg]

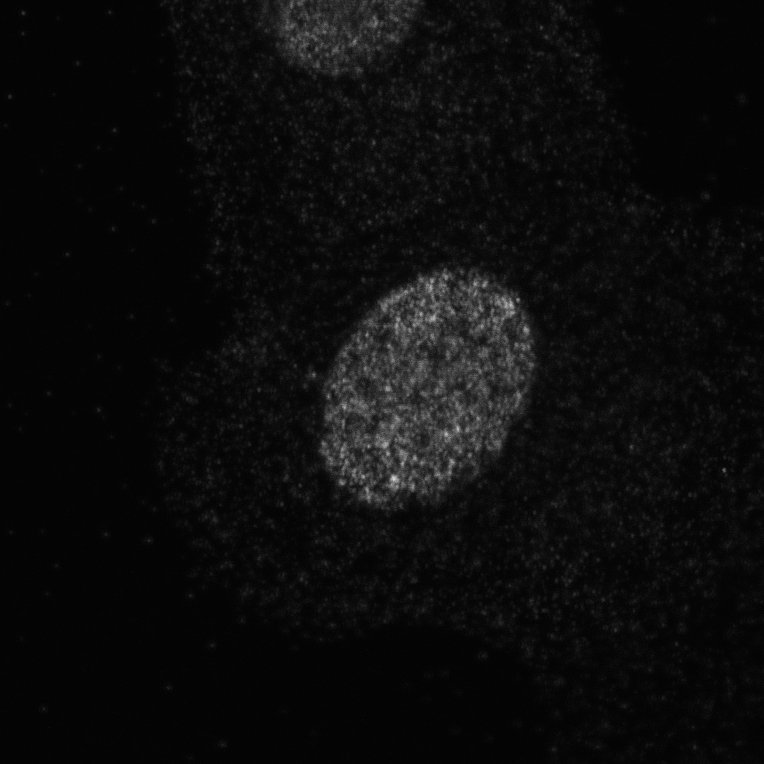

Supplement: Supplementary file 5 — Source data Fig. 1 [file 44318_2024_348_MOESM5_ESM.zip › Figure 1/1I/Immunofluorescence FLAG-Y673A Y675A in Wapl KO.jpg]

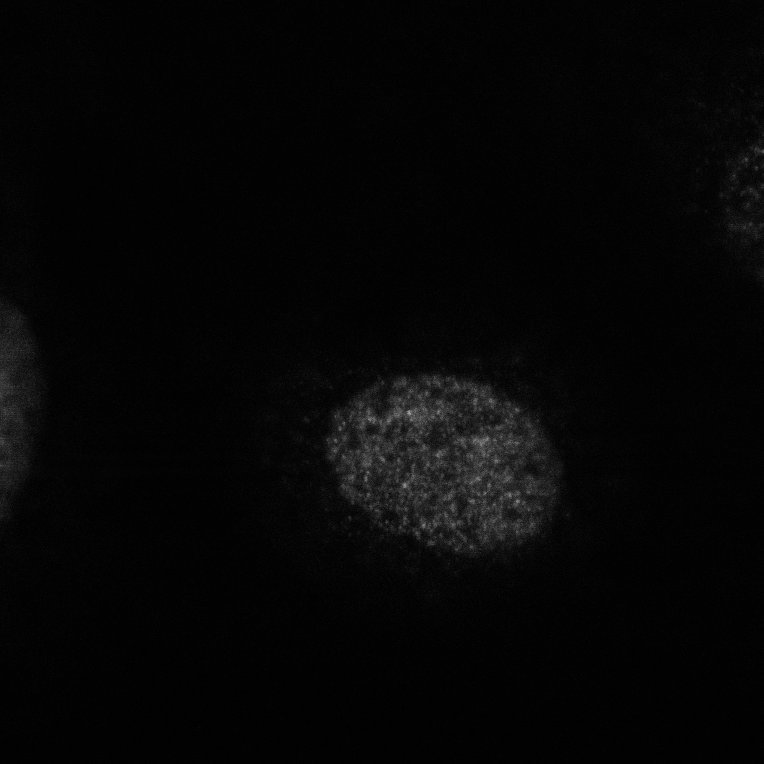

Supplement: Supplementary file 5 — Source data Fig. 1 [file 44318_2024_348_MOESM5_ESM.zip › Figure 1/1I/Immunofluorescence FLAG-Y673A Y675A in WT.jpg]

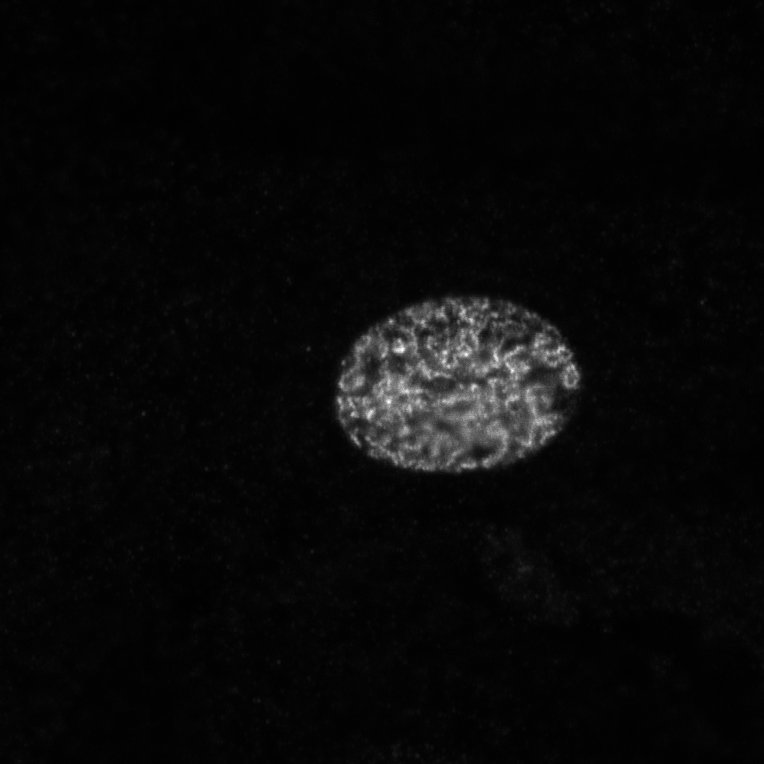

Supplement: Supplementary file 5 — Source data Fig. 1 [file 44318_2024_348_MOESM5_ESM.zip › Figure 1/1I/Immunofluorescence FLAG-Y869A Y871A in Wapl KO.jpg]

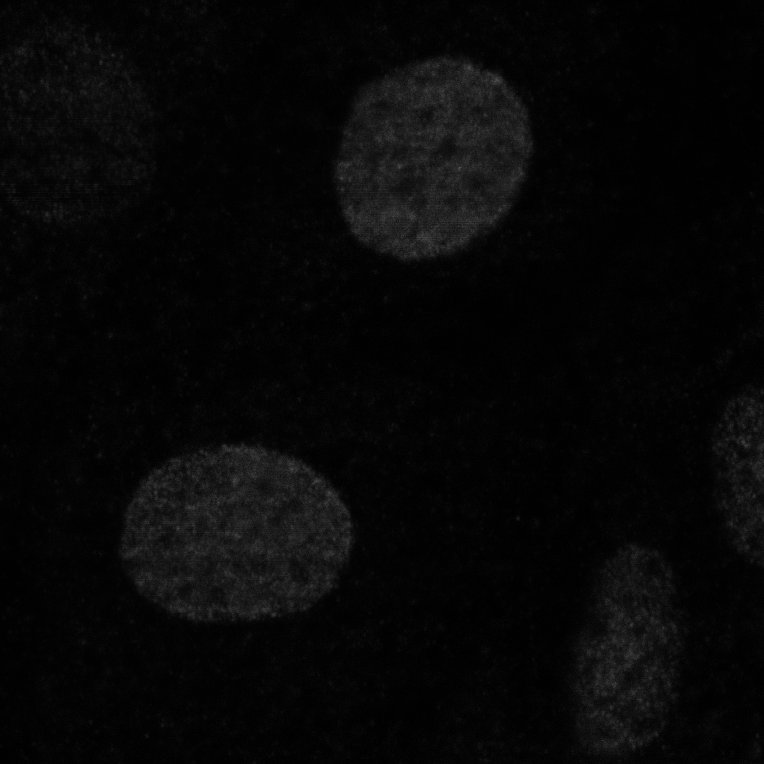

Supplement: Supplementary file 5 — Source data Fig. 1 [file 44318_2024_348_MOESM5_ESM.zip › Figure 1/1I/Immunofluorescence FLAG-Y869A Y871A in WT.jpg]

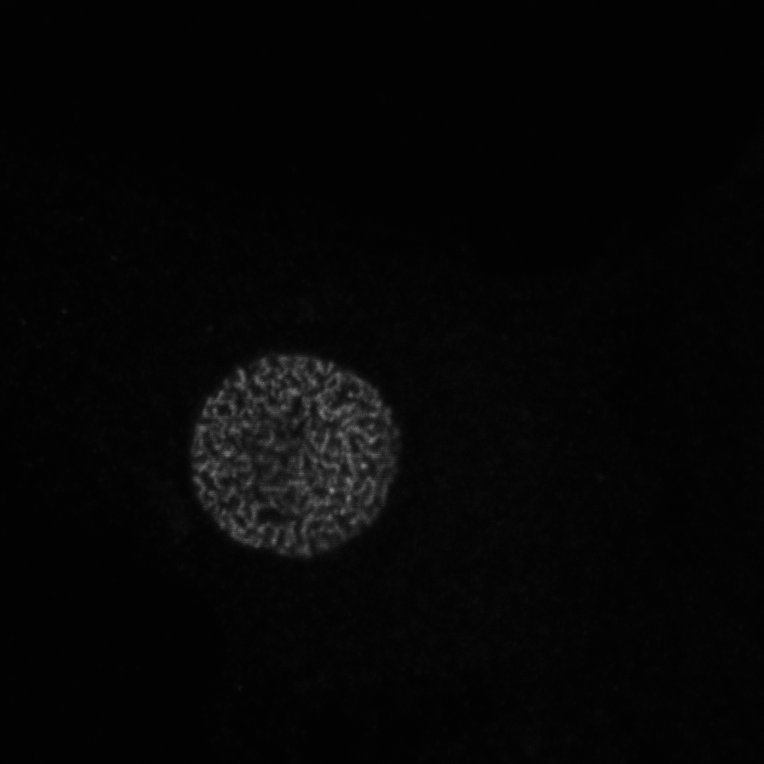

Supplement: Supplementary file 5 — Source data Fig. 1 [file 44318_2024_348_MOESM5_ESM.zip › Figure 1/1I/Immunofluorescence Smc3-Del 673-711 in Wapl KO.jpg]

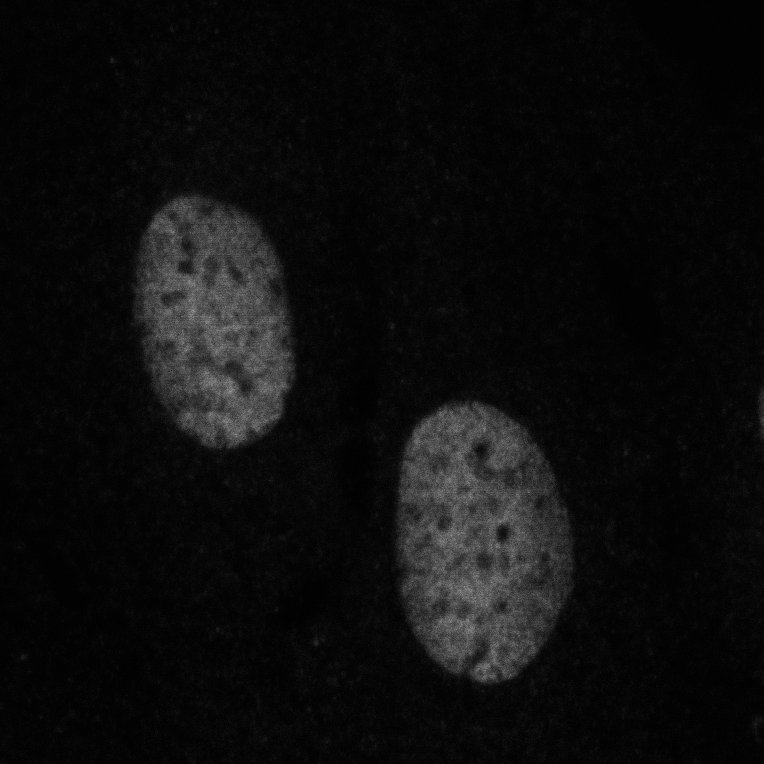

Supplement: Supplementary file 5 — Source data Fig. 1 [file 44318_2024_348_MOESM5_ESM.zip › Figure 1/1I/Immunofluorescence Smc3-Del 673-711 in WT.jpg]

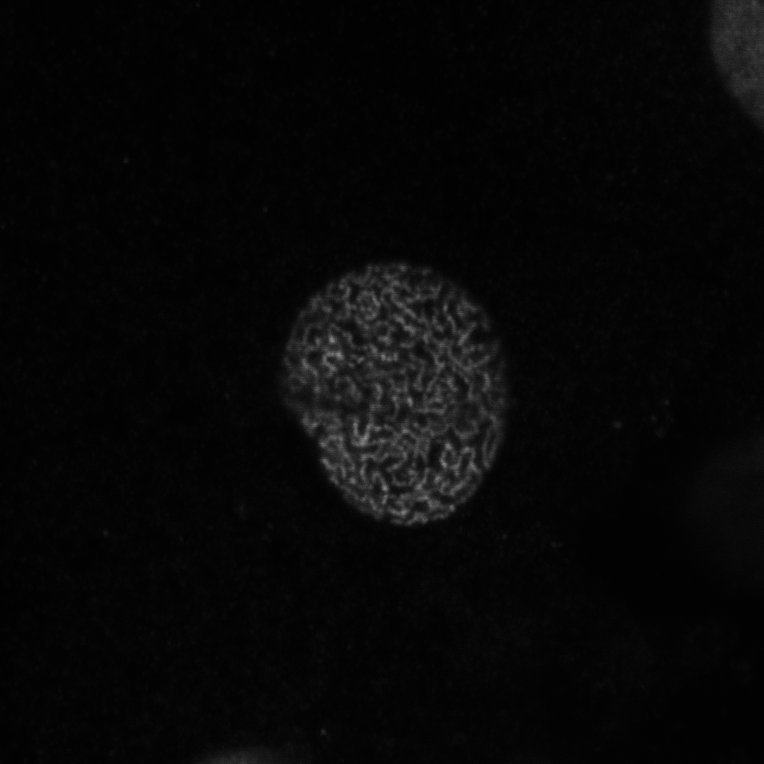

Supplement: Supplementary file 5 — Source data Fig. 1 [file 44318_2024_348_MOESM5_ESM.zip › Figure 1/1I/Immunofluorescence Smc3-wildtype Phf2 in Wapl KO.jpg]

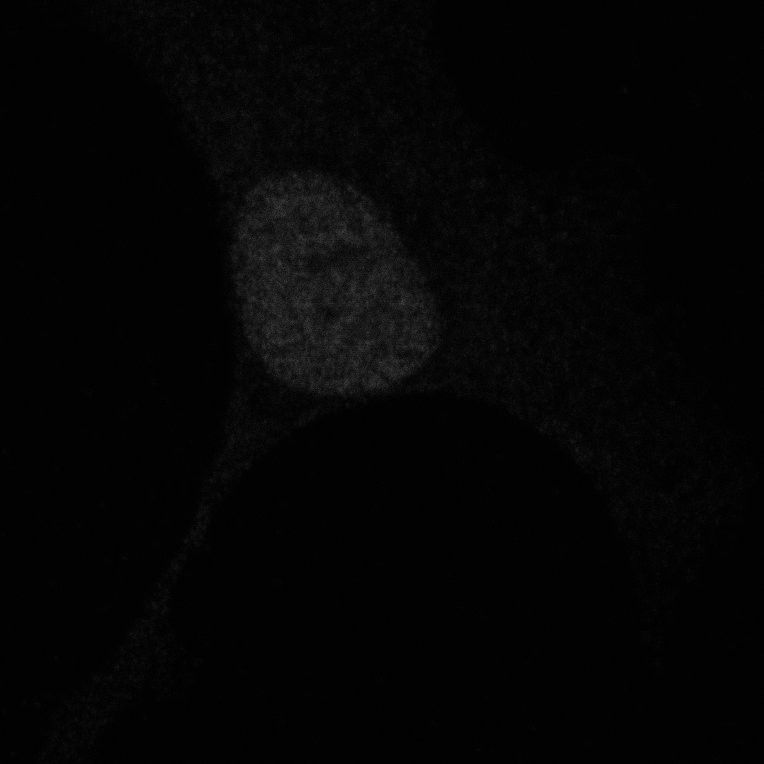

Supplement: Supplementary file 5 — Source data Fig. 1 [file 44318_2024_348_MOESM5_ESM.zip › Figure 1/1I/Immunofluorescence Smc3-wildtype Phf2 in WT.jpg]

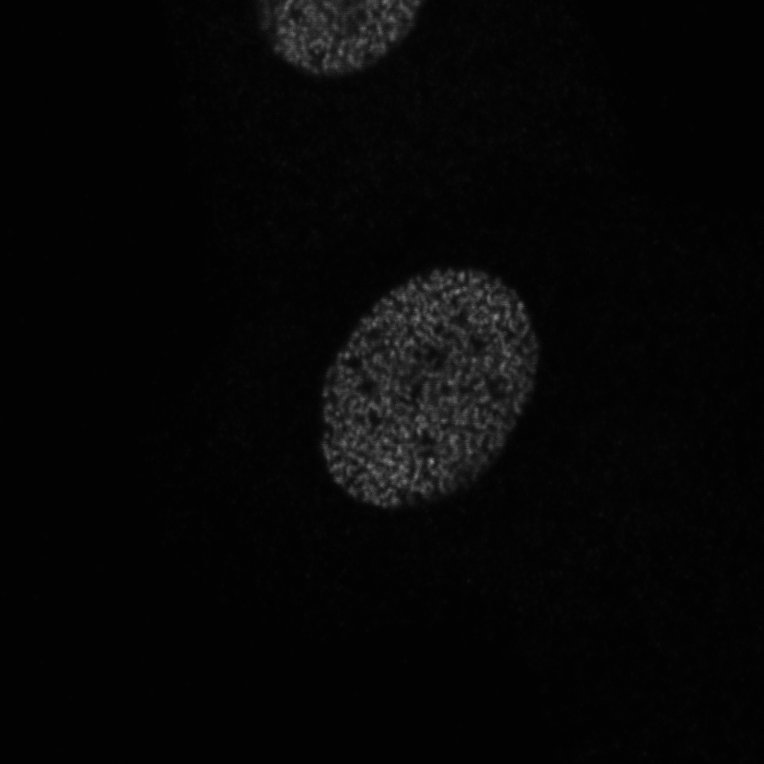

Supplement: Supplementary file 5 — Source data Fig. 1 [file 44318_2024_348_MOESM5_ESM.zip › Figure 1/1I/Immunofluorescence Smc3-Y673A Y675A in Wapl KO.jpg]

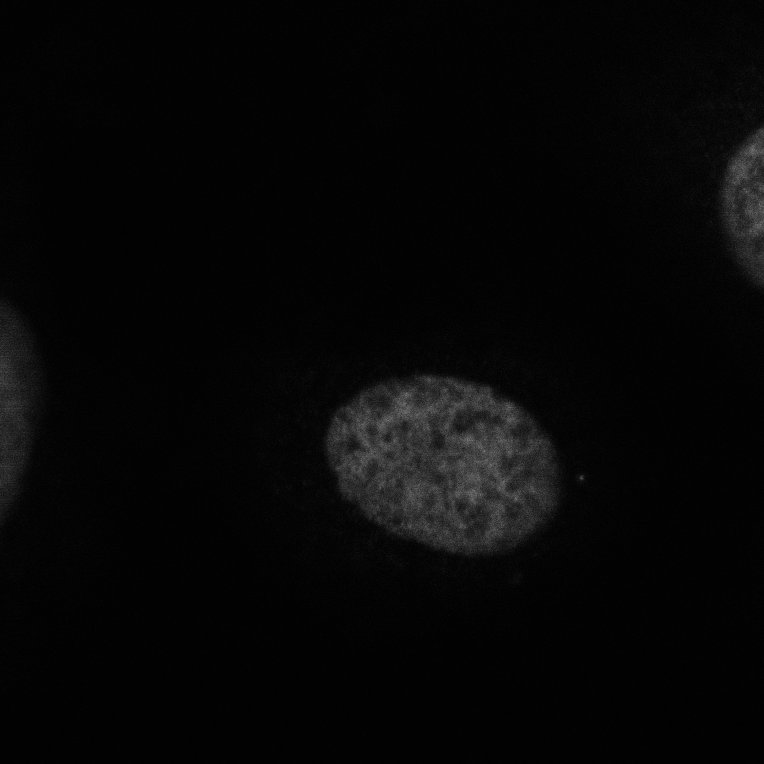

Supplement: Supplementary file 5 — Source data Fig. 1 [file 44318_2024_348_MOESM5_ESM.zip › Figure 1/1I/Immunofluorescence Smc3-Y673A Y675A in WT.jpg]

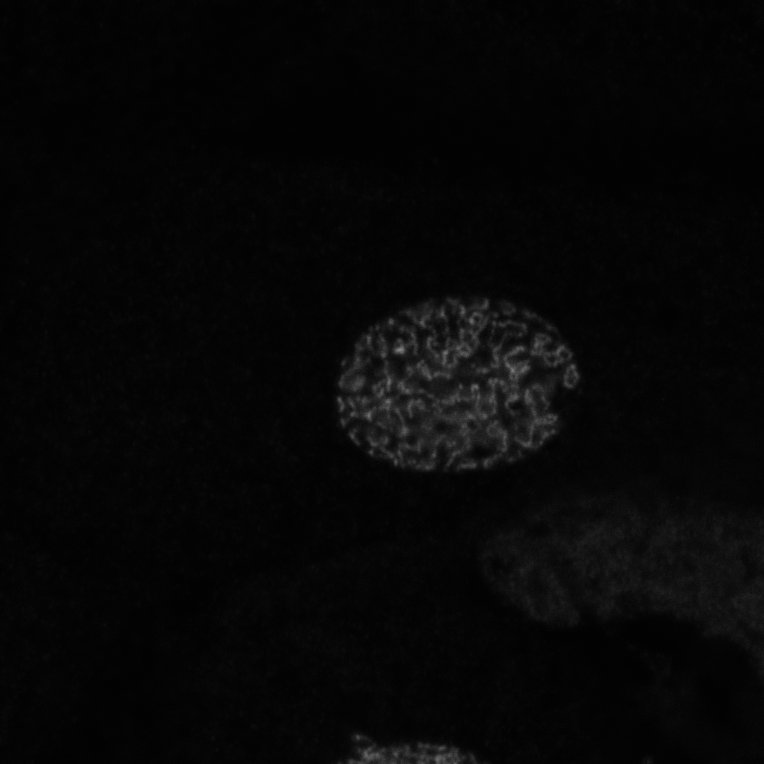

Supplement: Supplementary file 5 — Source data Fig. 1 [file 44318_2024_348_MOESM5_ESM.zip › Figure 1/1I/Immunofluorescence Smc3-Y869A Y871A in Wapl KO.jpg]

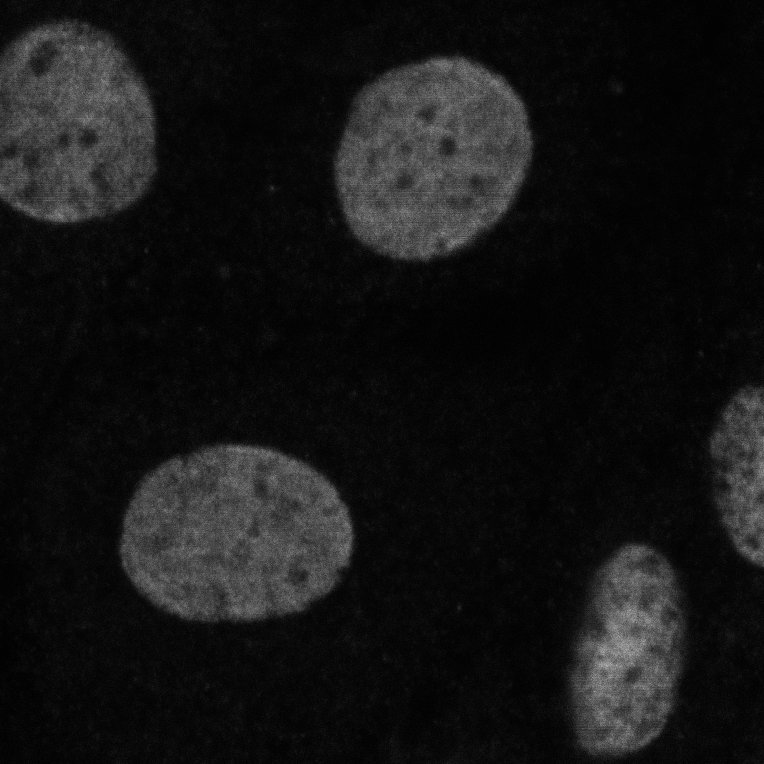

Supplement: Supplementary file 5 — Source data Fig. 1 [file 44318_2024_348_MOESM5_ESM.zip › Figure 1/1I/Immunofluorescence Smc3-Y869A Y871A in WT.jpg]

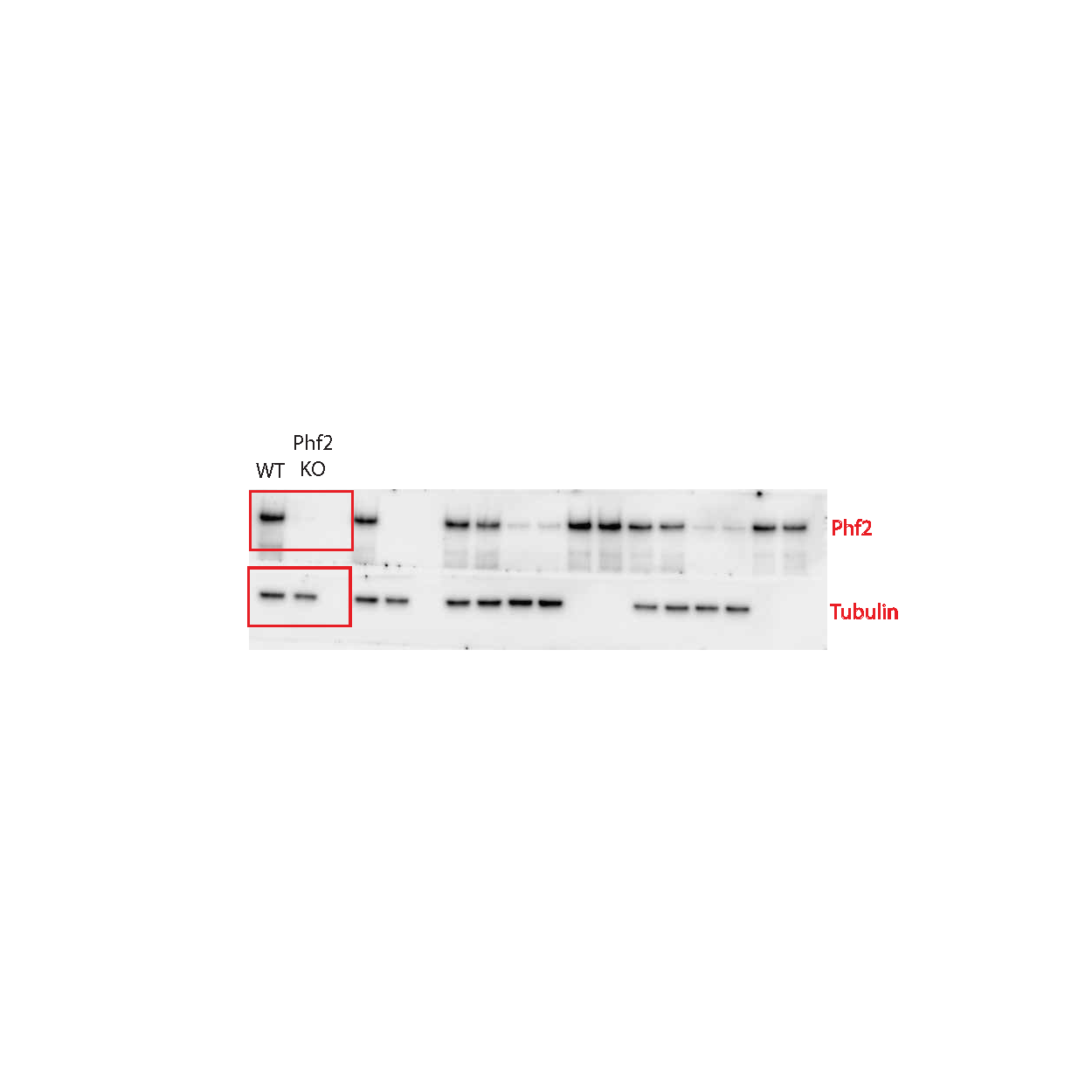

Supplement: Supplementary file 6 — Source data Fig. 2 [file 44318_2024_348_MOESM6_ESM.zip › Figure 2/2D/western Fig 2D.tiff]

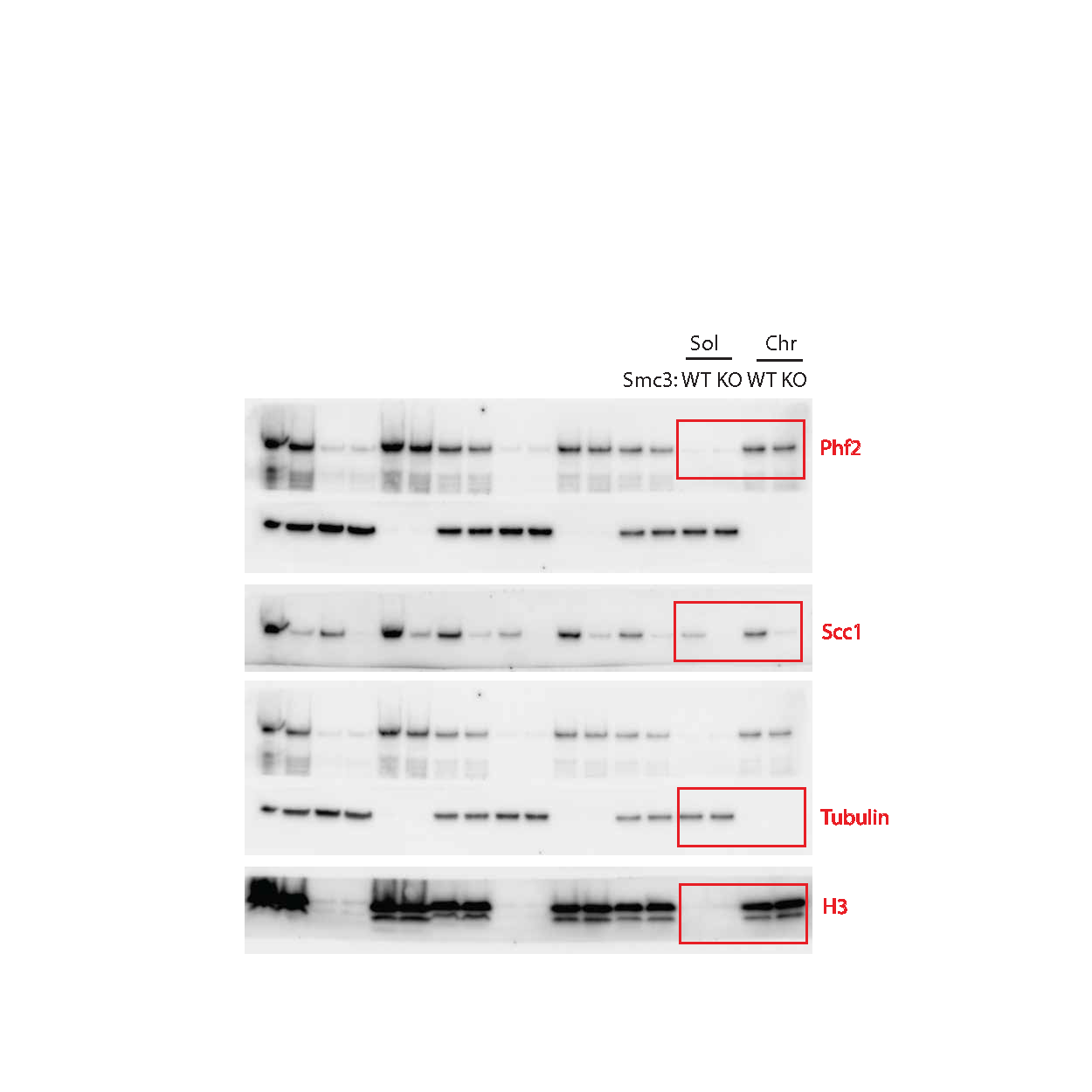

Supplement: Supplementary file 7 — Source data Fig. 3 [file 44318_2024_348_MOESM7_ESM.zip › Figure 3/3A/western Fig 3A.tiff]

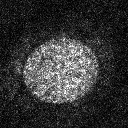

Supplement: Supplementary file 8 — Source data Fig. 4 [file 44318_2024_348_MOESM8_ESM.zip › Figure 4/4A/live cell imaging Phf2-GFP in Wapl KO.lsm]

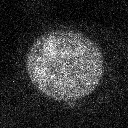

Supplement: Supplementary file 8 — Source data Fig. 4 [file 44318_2024_348_MOESM8_ESM.zip › Figure 4/4A/live cell imaging Phf2-GFP in Scc1 siRNA + Wapl KO.lsm]

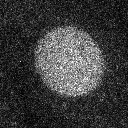

Supplement: Supplementary file 8 — Source data Fig. 4 [file 44318_2024_348_MOESM8_ESM.zip › Figure 4/4A/live cell imaging Phf2-GFP in control.lsm]

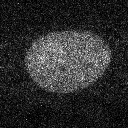

Supplement: Supplementary file 8 — Source data Fig. 4 [file 44318_2024_348_MOESM8_ESM.zip › Figure 4/4A/live cell imaging Phf2-GFP in Scc1 siRNA.lsm]

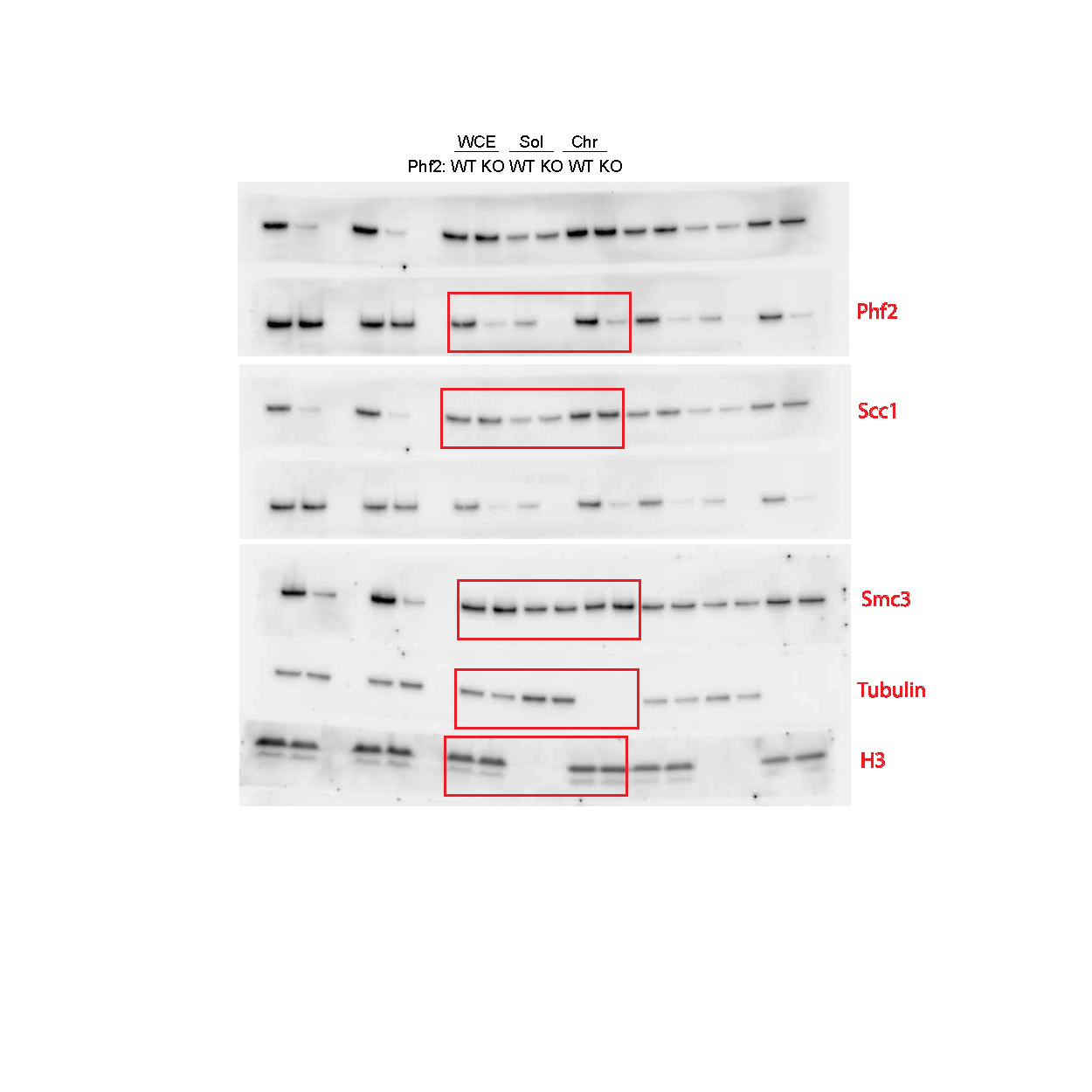

Supplement: Supplementary file 12 — Source data Fig. 8 [file 44318_2024_348_MOESM12_ESM.zip › Figure 8/8D/western Fig 8D.tiff]

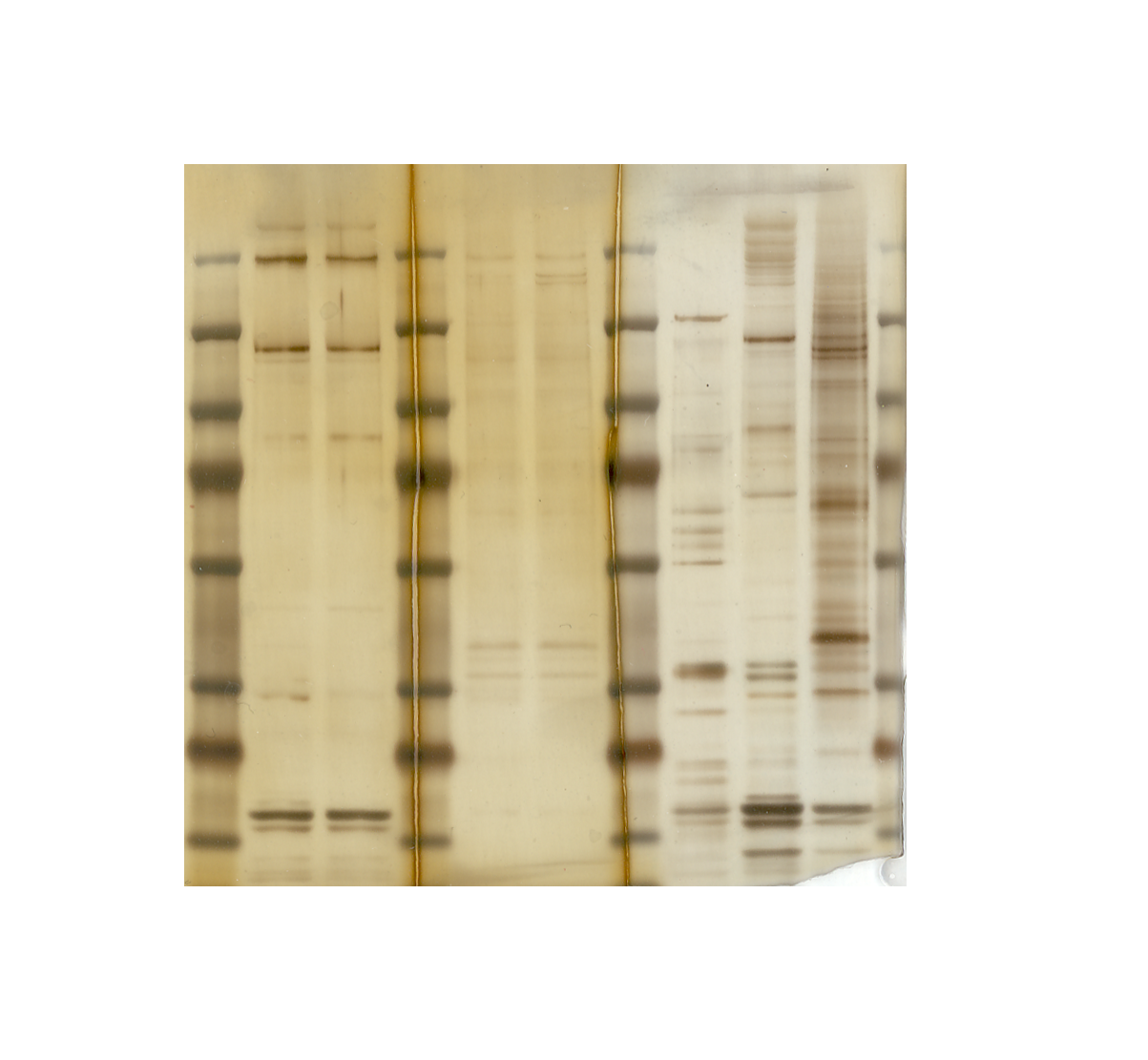

Supplement: Supplementary file 13 — Figures EV and Appendix Source Data [file 44318_2024_348_MOESM13_ESM.zip › SD figure EV and Appendix/Appendix Figure 2B.tif]

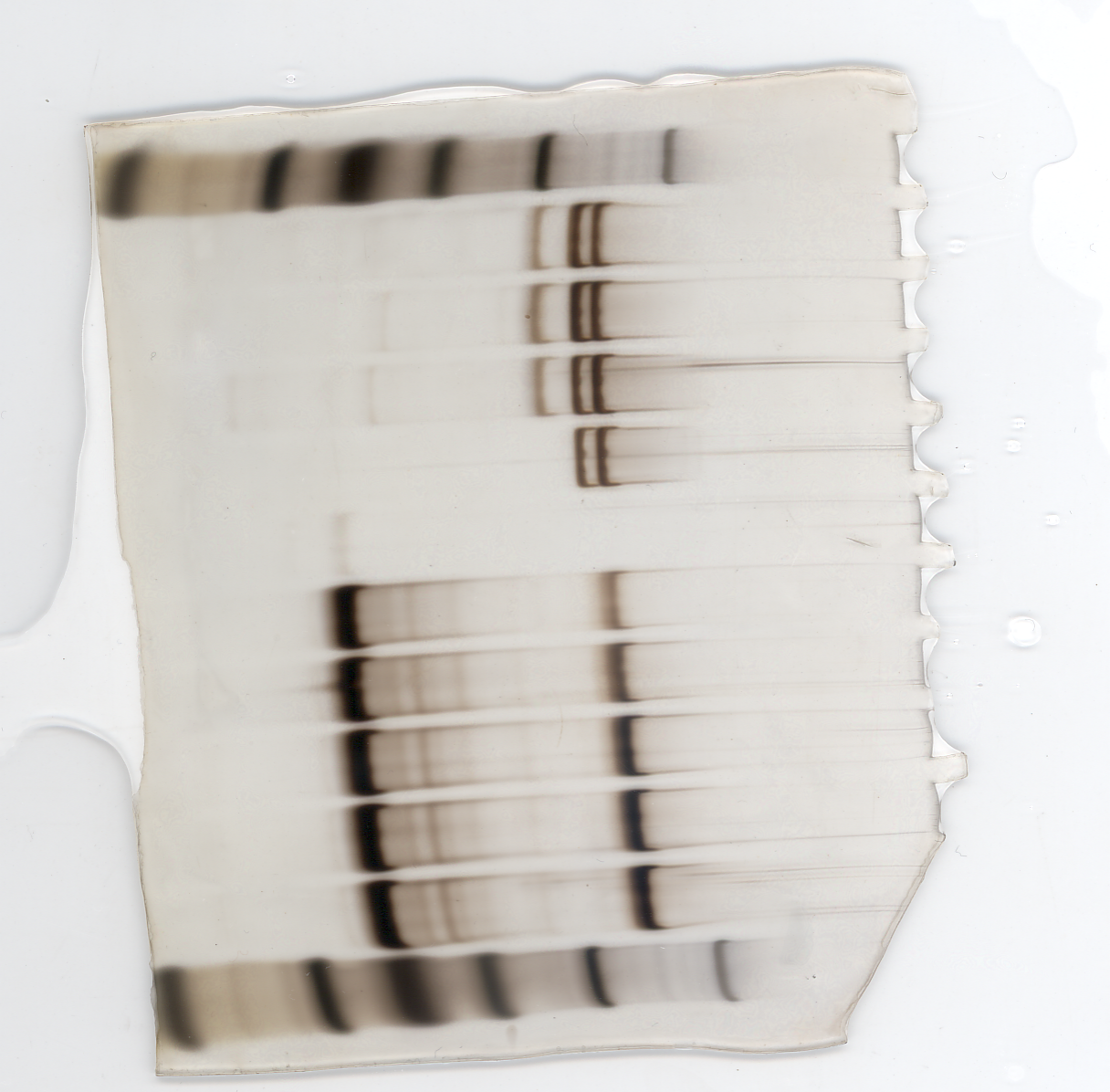

Supplement: Supplementary file 13 — Figures EV and Appendix Source Data [file 44318_2024_348_MOESM13_ESM.zip › SD figure EV and Appendix/Appendix Figure 2D.tif]

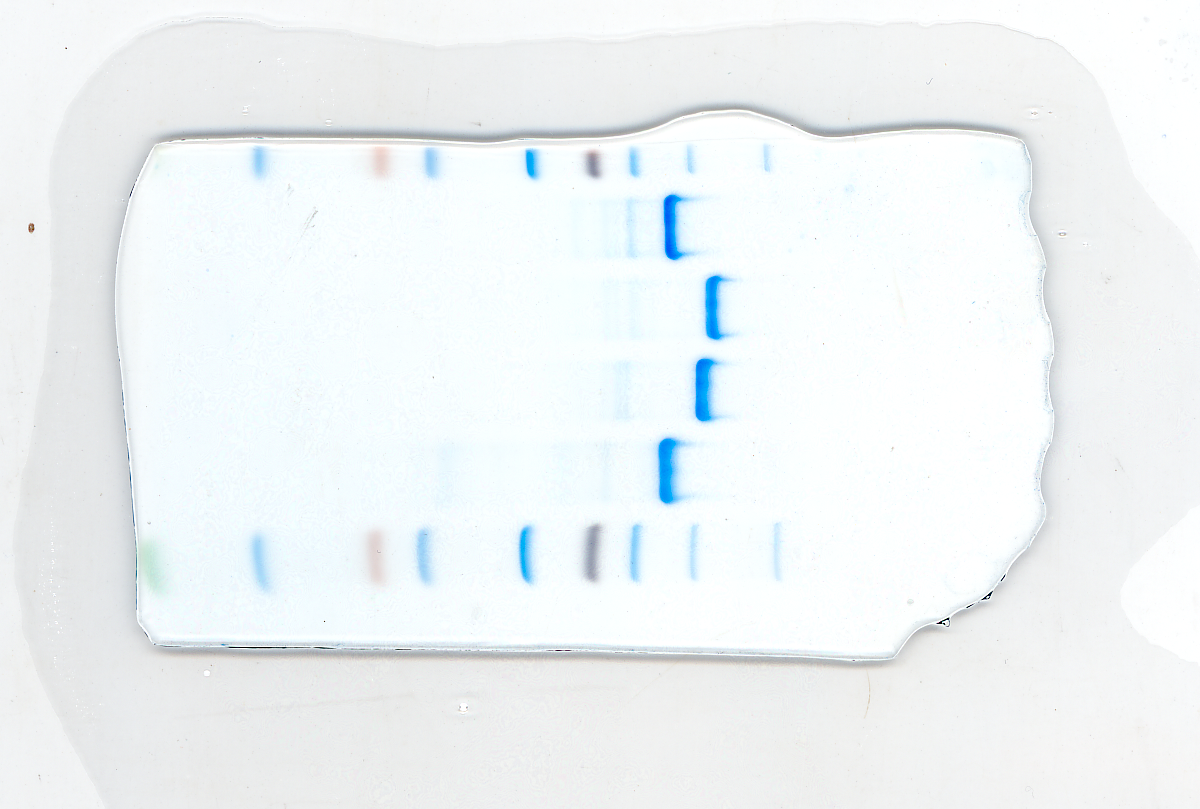

Supplement: Supplementary file 13 — Figures EV and Appendix Source Data [file 44318_2024_348_MOESM13_ESM.zip › SD figure EV and Appendix/Appendix Figure 2F.tif]

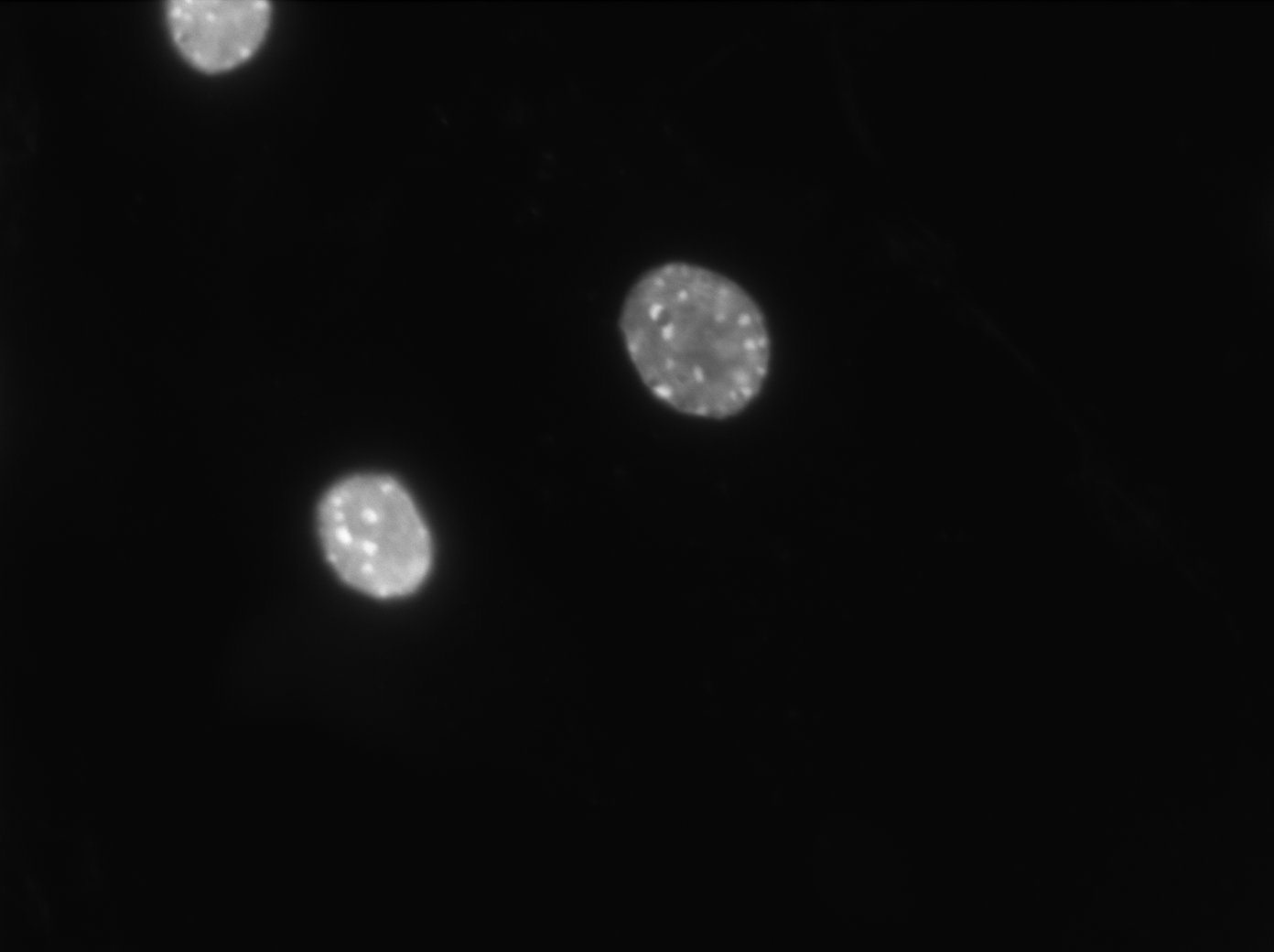

Supplement: Supplementary file 13 — Figures EV and Appendix Source Data [file 44318_2024_348_MOESM13_ESM.zip › SD figure EV and Appendix/Appendix Figure 1E/Acquired-7.jpg]

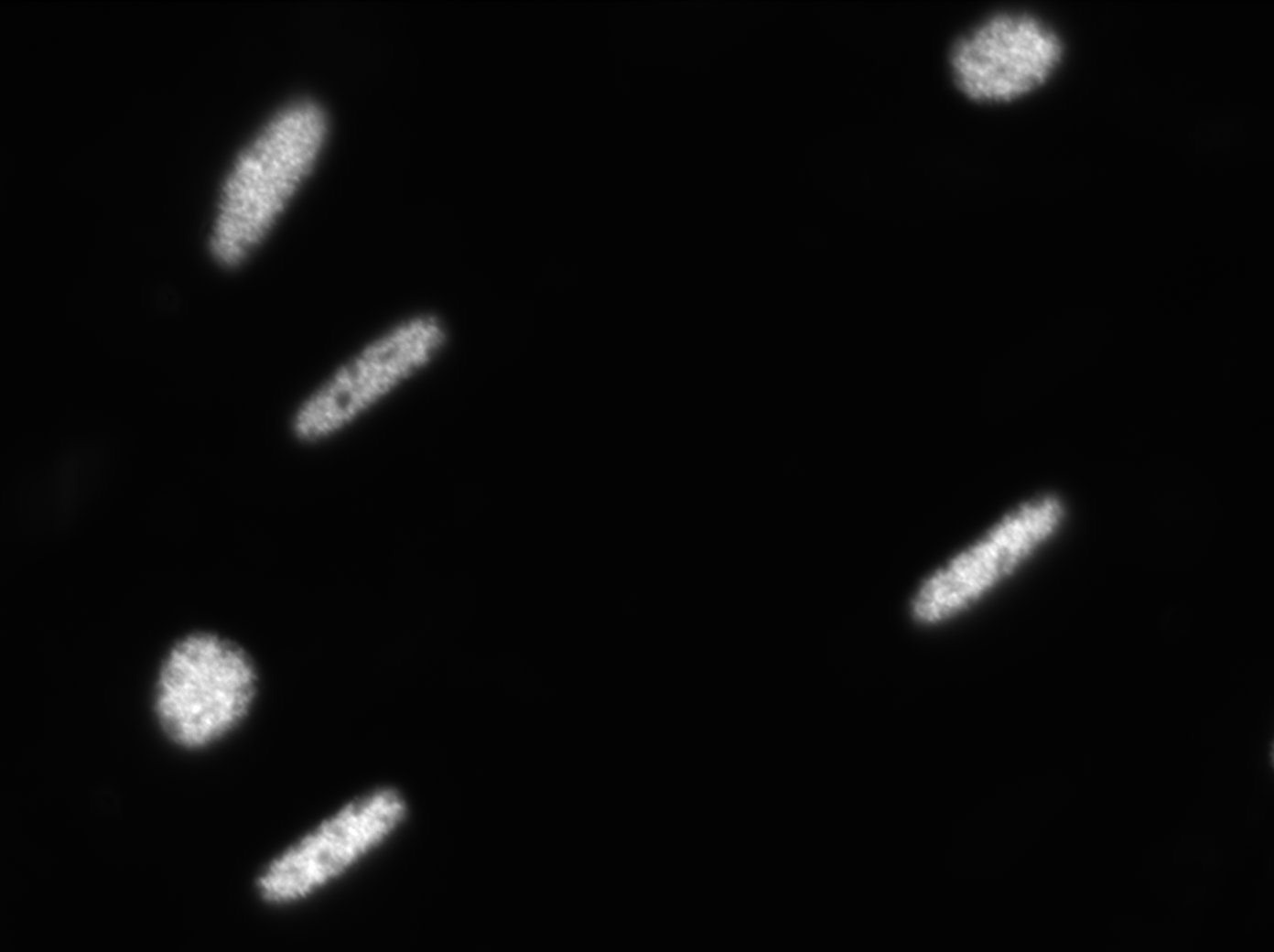

Supplement: Supplementary file 13 — Figures EV and Appendix Source Data [file 44318_2024_348_MOESM13_ESM.zip › SD figure EV and Appendix/Appendix Figure 1E/Acquired-6.jpg]

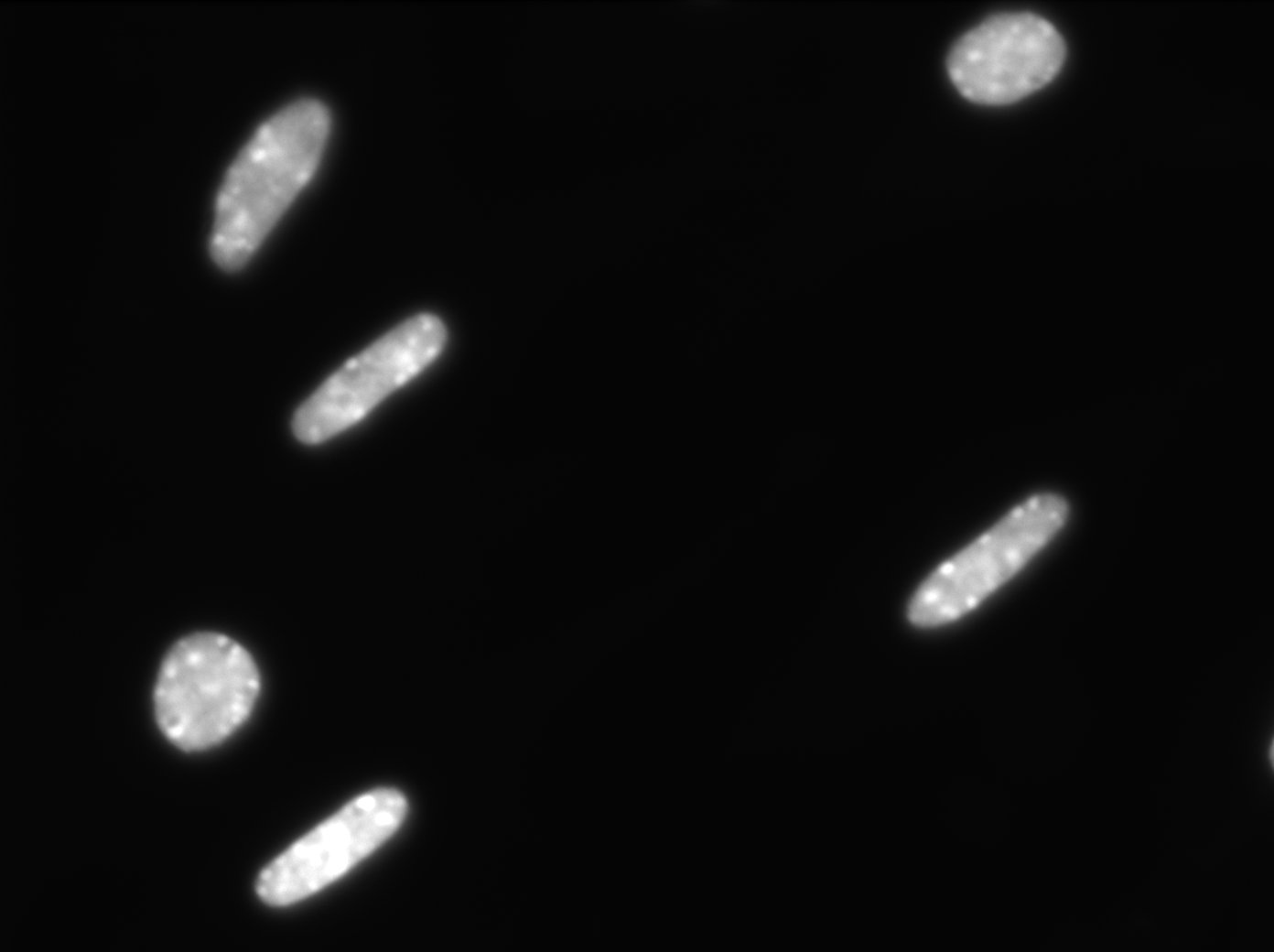

Supplement: Supplementary file 13 — Figures EV and Appendix Source Data [file 44318_2024_348_MOESM13_ESM.zip › SD figure EV and Appendix/Appendix Figure 1E/Acquired-4.jpg]

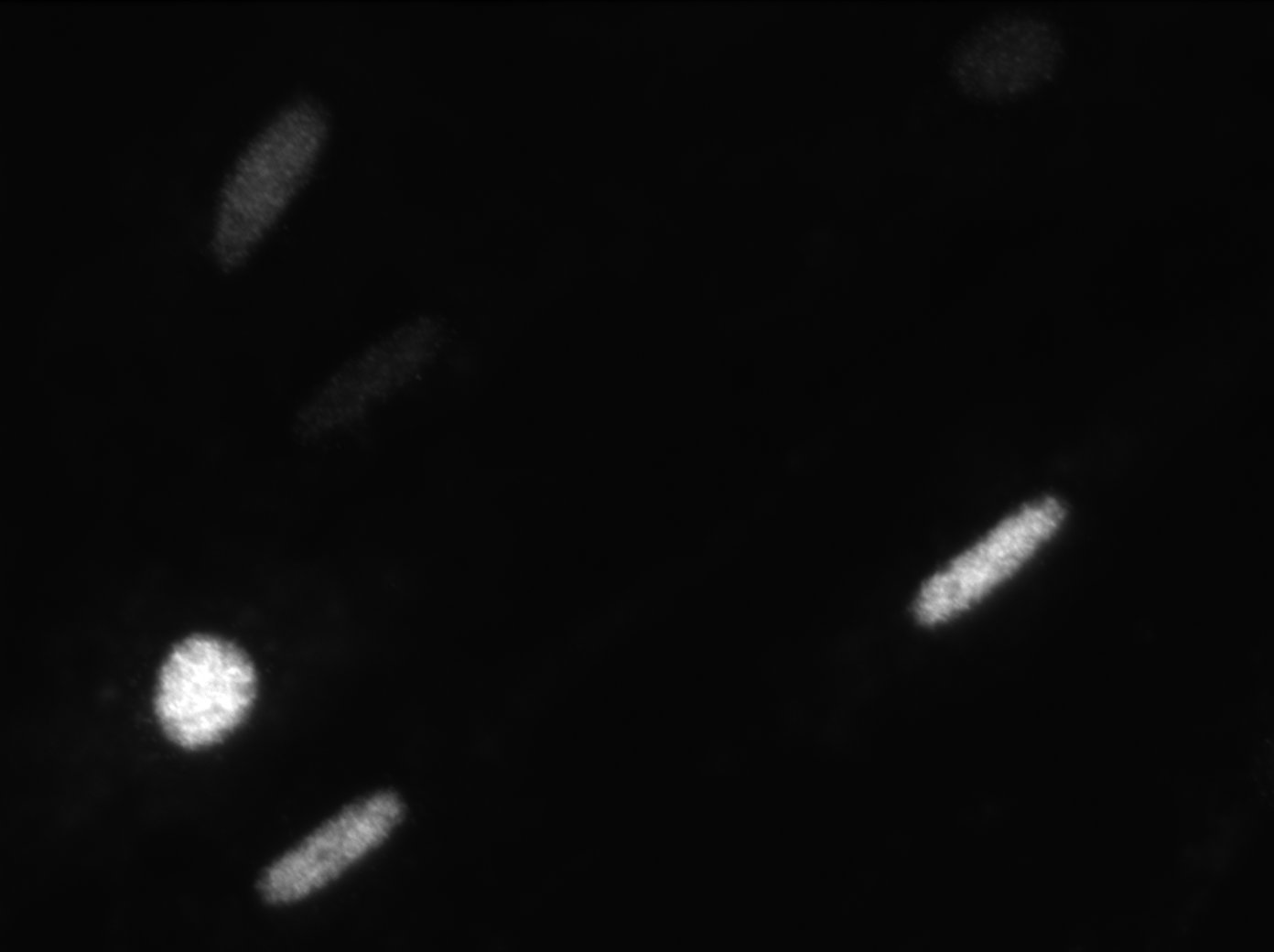

Supplement: Supplementary file 13 — Figures EV and Appendix Source Data [file 44318_2024_348_MOESM13_ESM.zip › SD figure EV and Appendix/Appendix Figure 1E/Acquired-5.jpg]

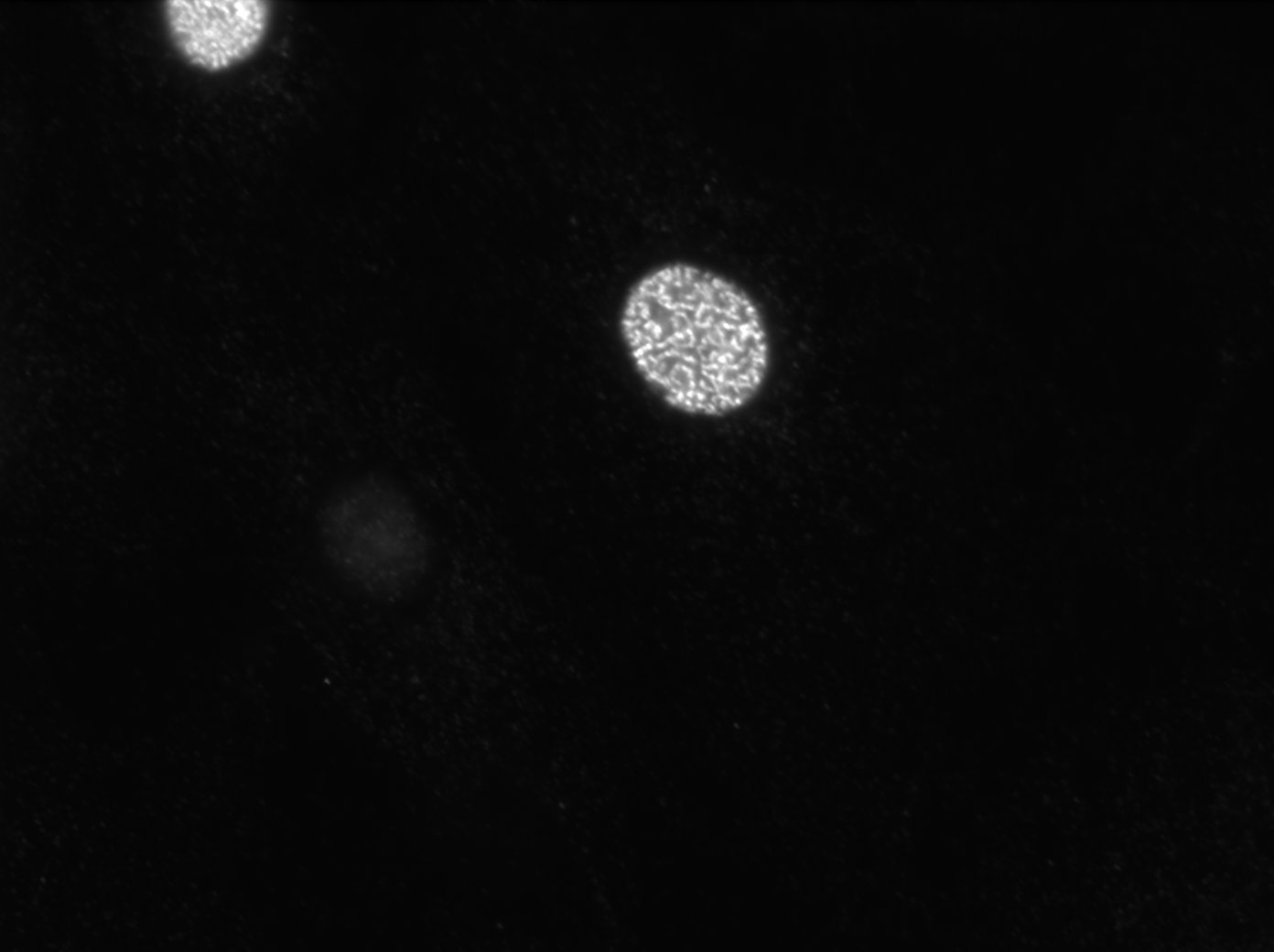

Supplement: Supplementary file 13 — Figures EV and Appendix Source Data [file 44318_2024_348_MOESM13_ESM.zip › SD figure EV and Appendix/Appendix Figure 1E/Acquired-8.jpg]

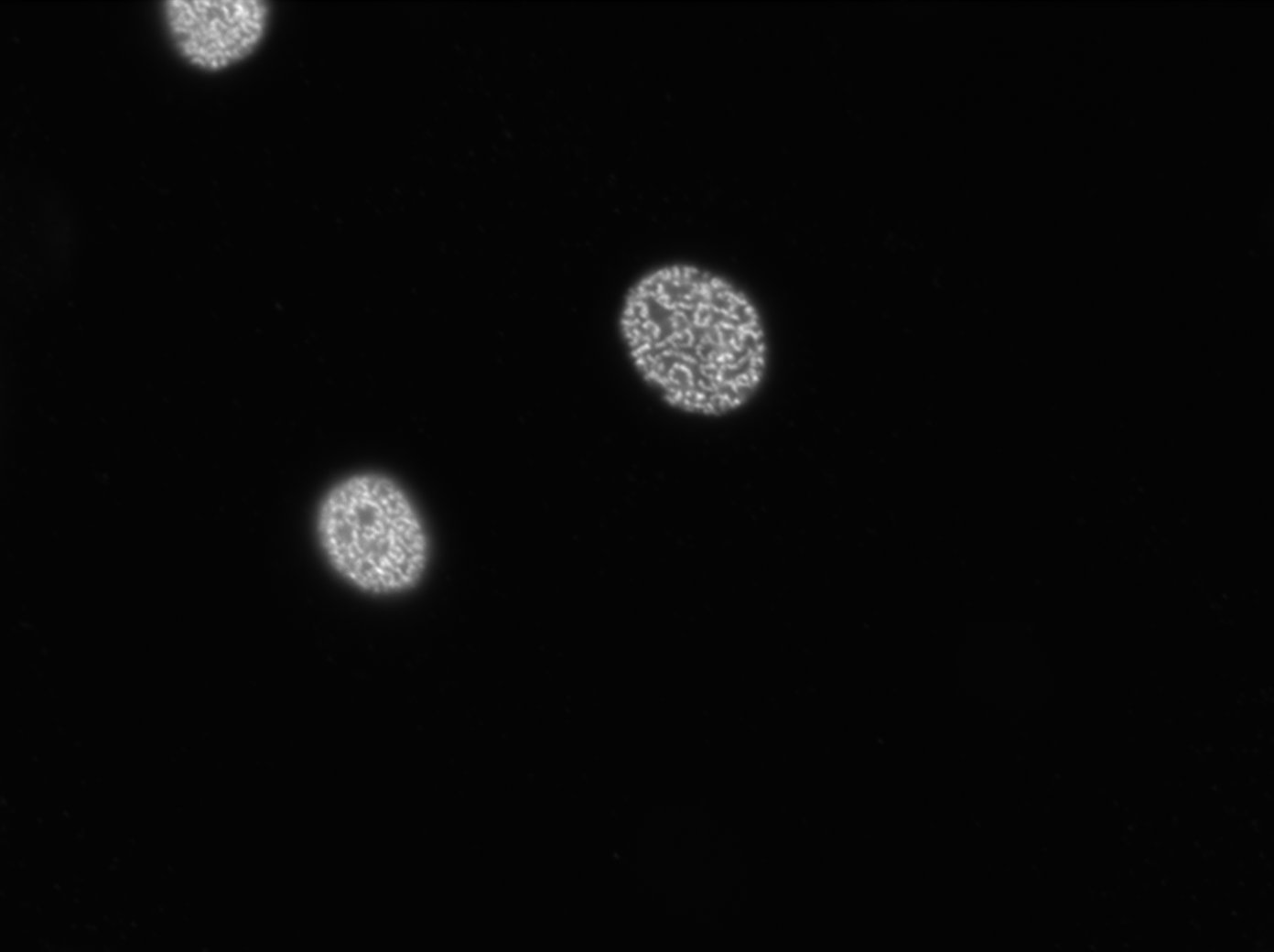

Supplement: Supplementary file 13 — Figures EV and Appendix Source Data [file 44318_2024_348_MOESM13_ESM.zip › SD figure EV and Appendix/Appendix Figure 1E/Acquired-9.jpg]

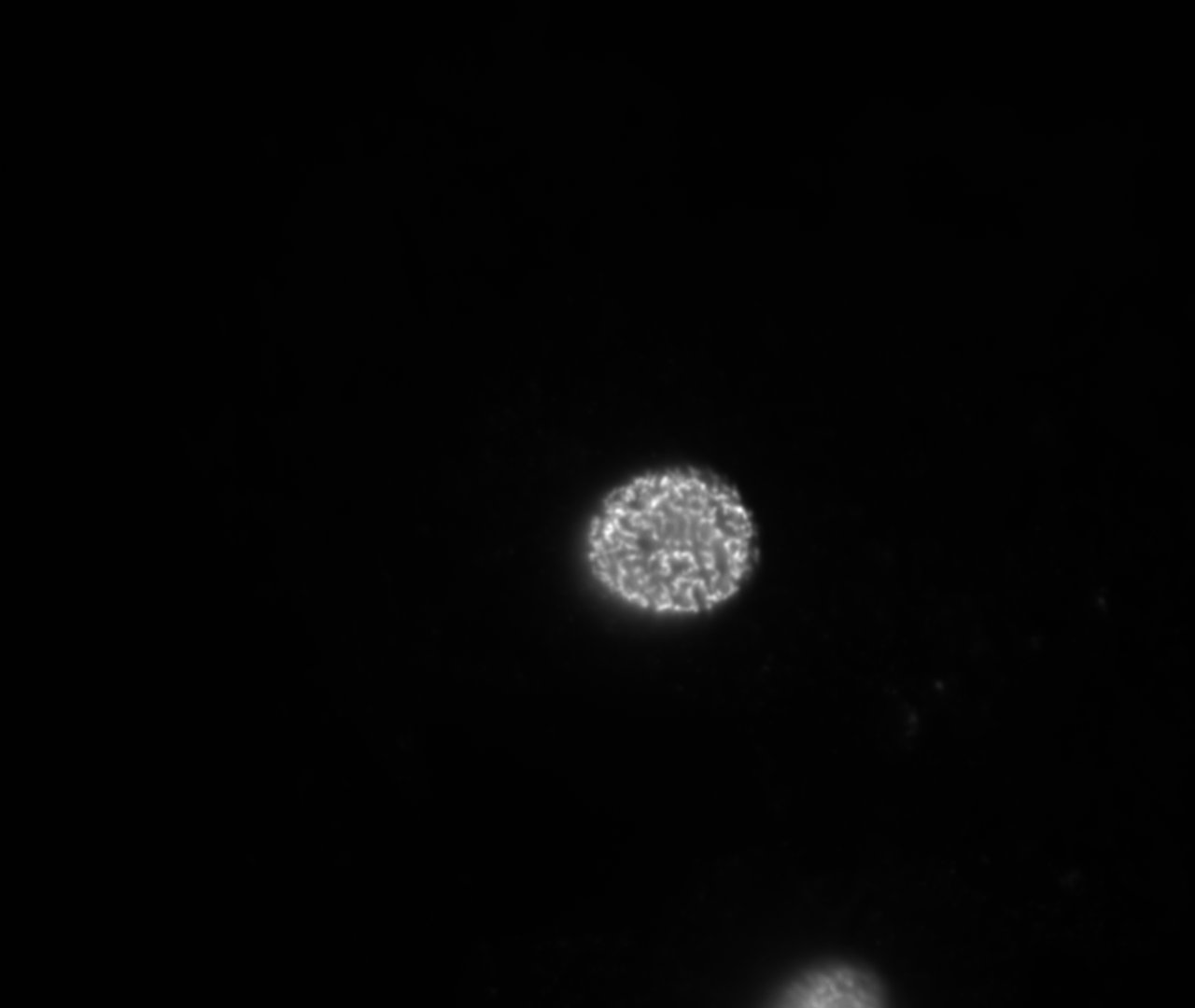

Supplement: Supplementary file 13 — Figures EV and Appendix Source Data [file 44318_2024_348_MOESM13_ESM.zip › SD figure EV and Appendix/Appendix Figure 2H/560-8.jpg]

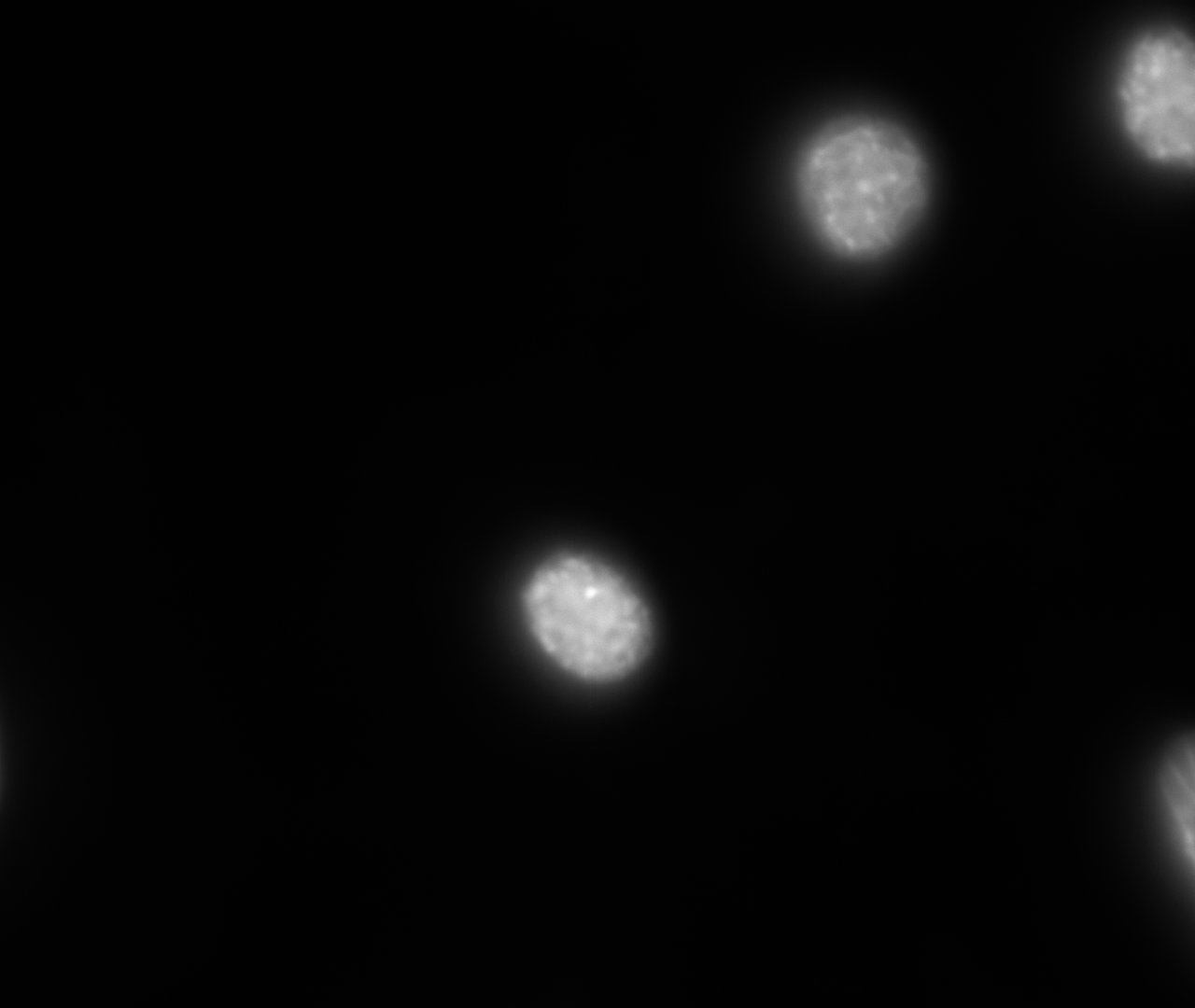

Supplement: Supplementary file 13 — Figures EV and Appendix Source Data [file 44318_2024_348_MOESM13_ESM.zip › SD figure EV and Appendix/Appendix Figure 2H/360.jpg]

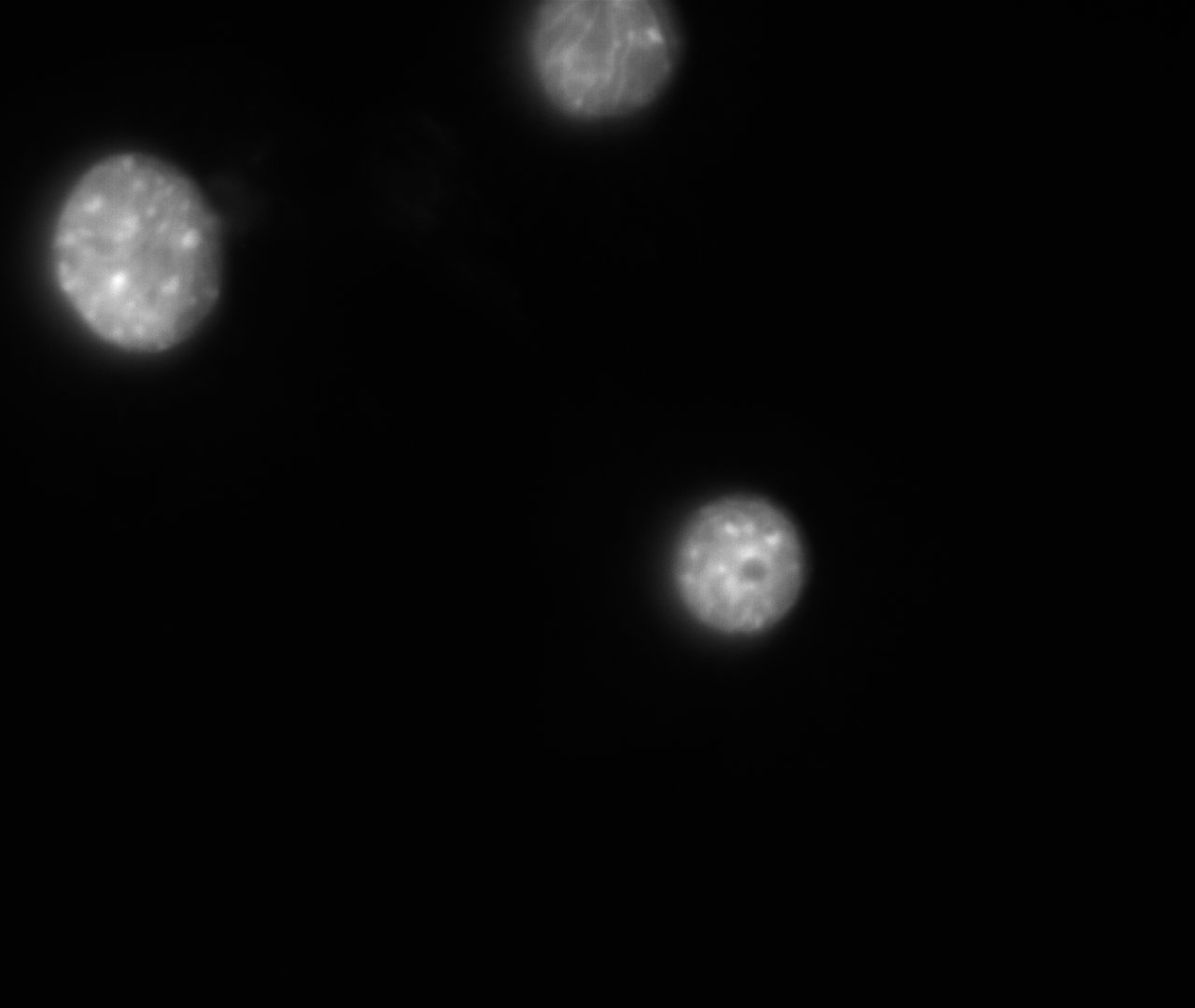

Supplement: Supplementary file 13 — Figures EV and Appendix Source Data [file 44318_2024_348_MOESM13_ESM.zip › SD figure EV and Appendix/Appendix Figure 2H/360-2.jpg]

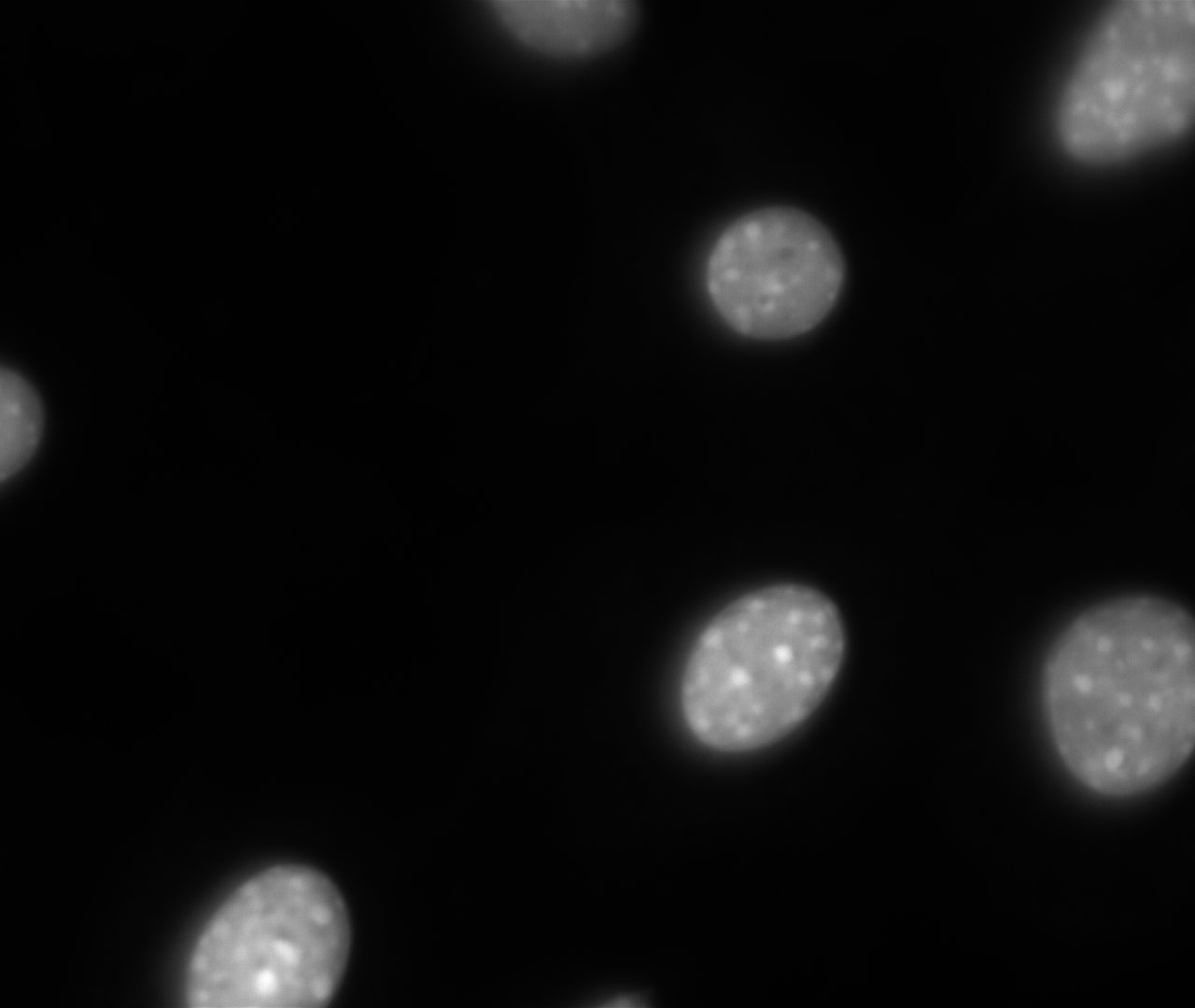

Supplement: Supplementary file 13 — Figures EV and Appendix Source Data [file 44318_2024_348_MOESM13_ESM.zip › SD figure EV and Appendix/Appendix Figure 2H/360-3.jpg]

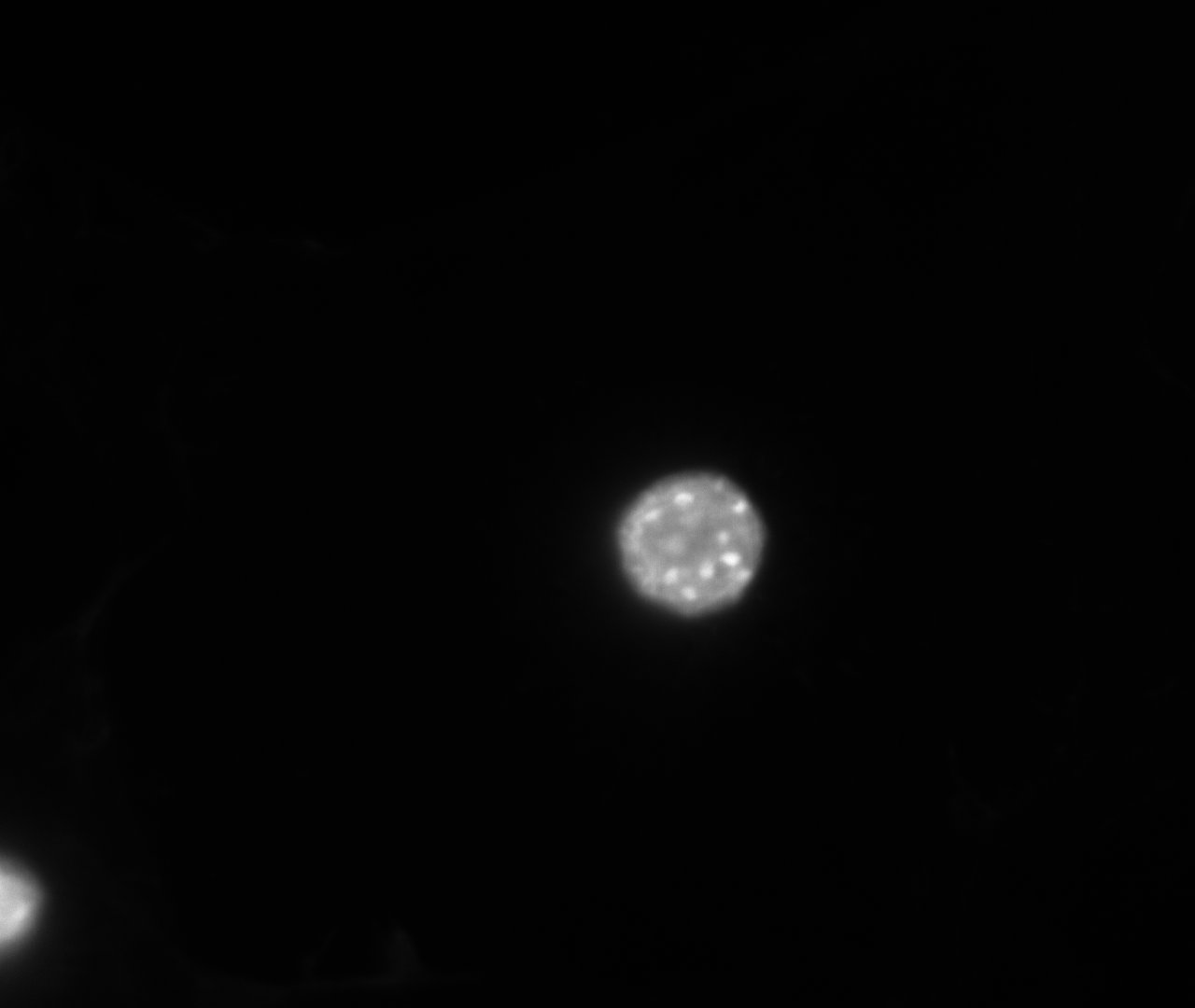

Supplement: Supplementary file 13 — Figures EV and Appendix Source Data [file 44318_2024_348_MOESM13_ESM.zip › SD figure EV and Appendix/Appendix Figure 2H/360-4.jpg]

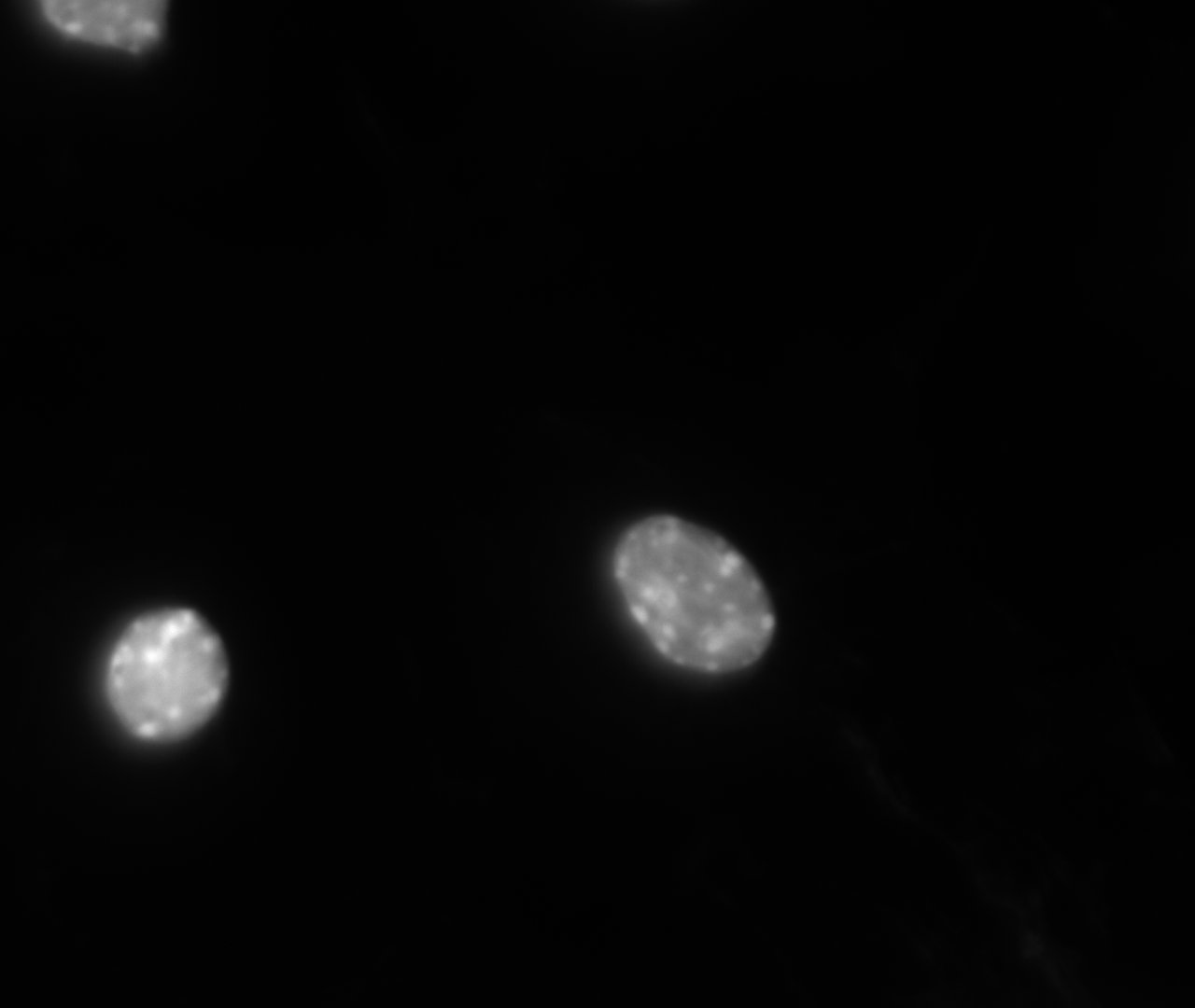

Supplement: Supplementary file 13 — Figures EV and Appendix Source Data [file 44318_2024_348_MOESM13_ESM.zip › SD figure EV and Appendix/Appendix Figure 2H/360-5.jpg]

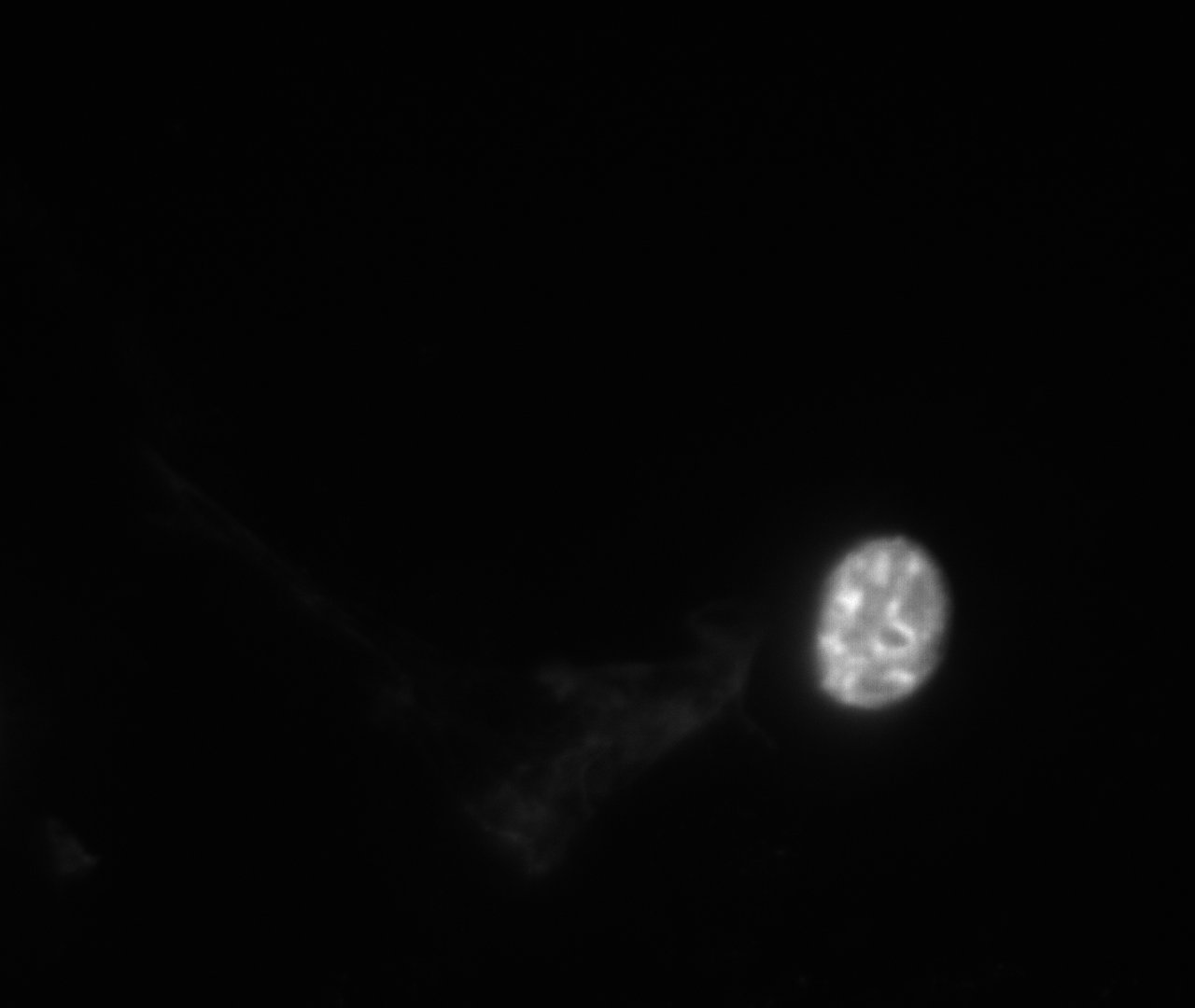

Supplement: Supplementary file 13 — Figures EV and Appendix Source Data [file 44318_2024_348_MOESM13_ESM.zip › SD figure EV and Appendix/Appendix Figure 2H/360-6.jpg]

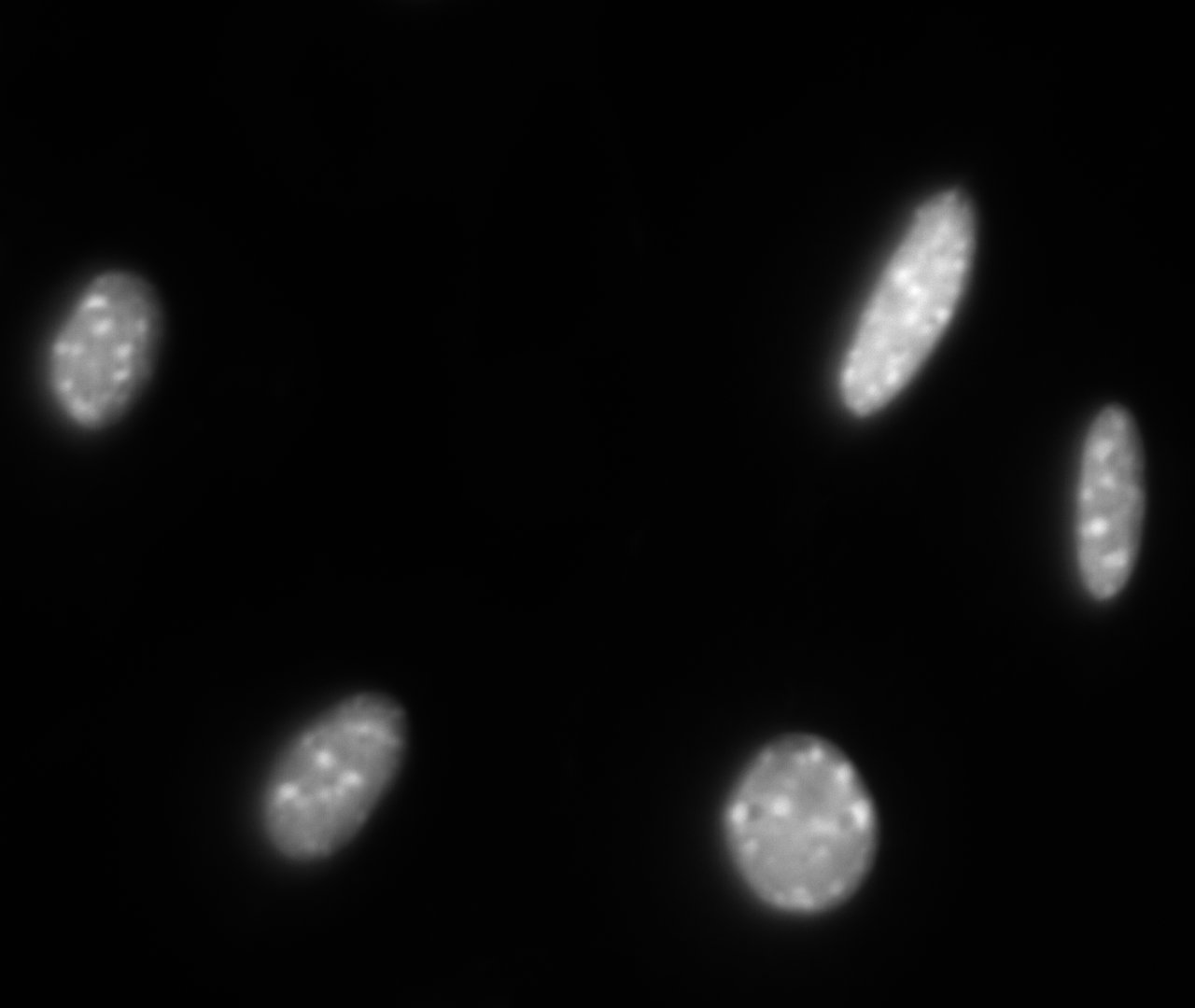

Supplement: Supplementary file 13 — Figures EV and Appendix Source Data [file 44318_2024_348_MOESM13_ESM.zip › SD figure EV and Appendix/Appendix Figure 2H/360-7.jpg]

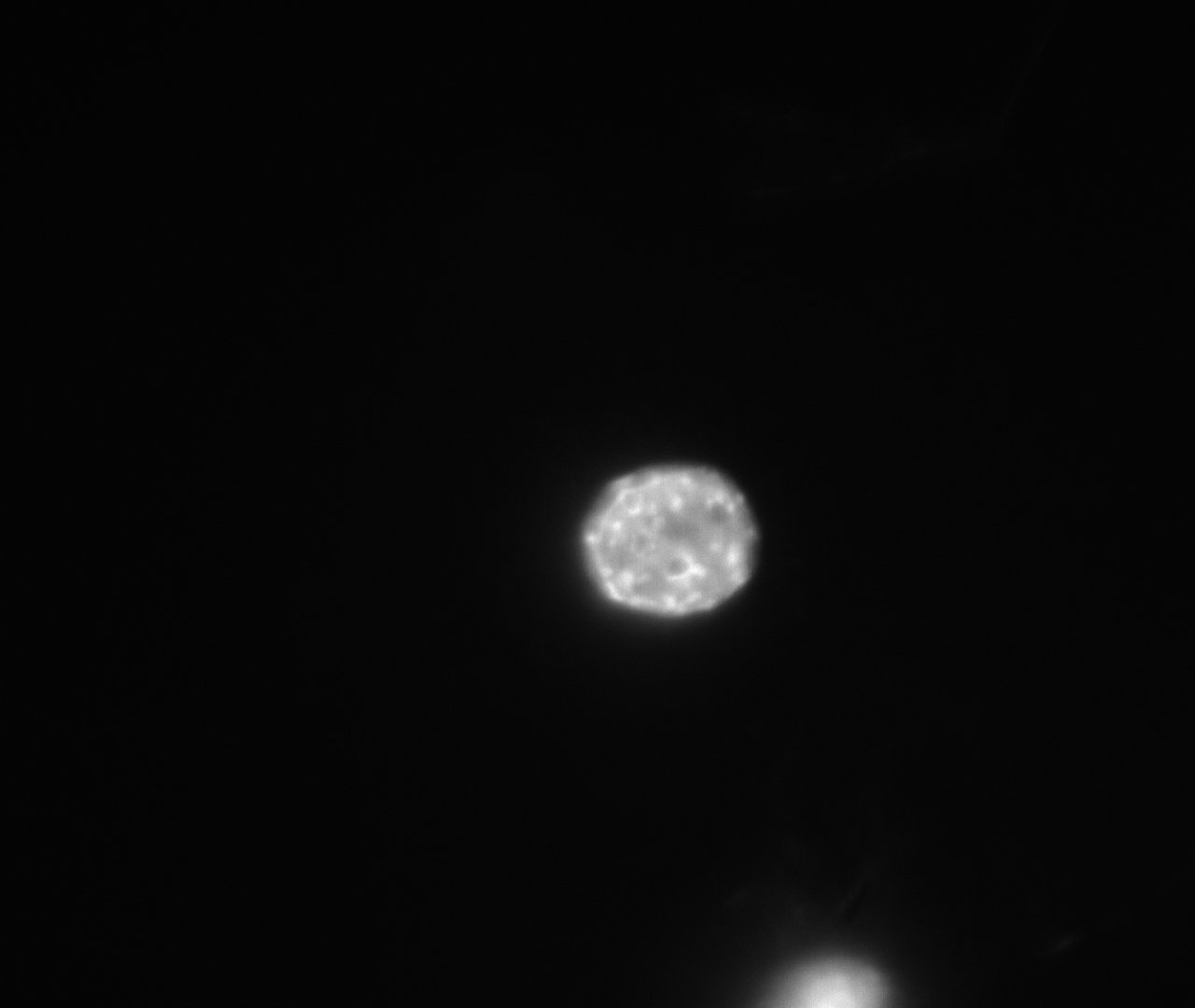

Supplement: Supplementary file 13 — Figures EV and Appendix Source Data [file 44318_2024_348_MOESM13_ESM.zip › SD figure EV and Appendix/Appendix Figure 2H/360-8.jpg]

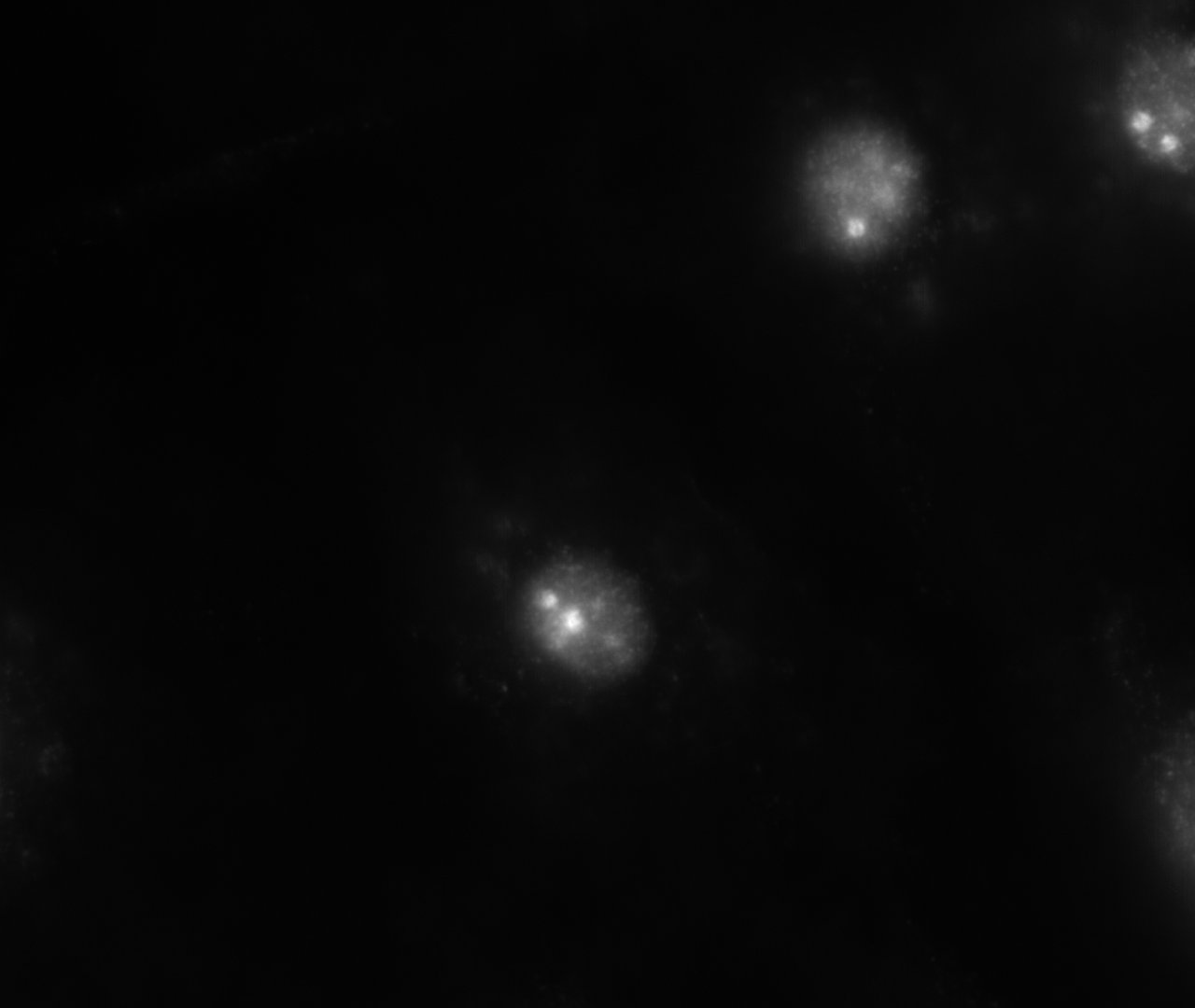

Supplement: Supplementary file 13 — Figures EV and Appendix Source Data [file 44318_2024_348_MOESM13_ESM.zip › SD figure EV and Appendix/Appendix Figure 2H/480.jpg]

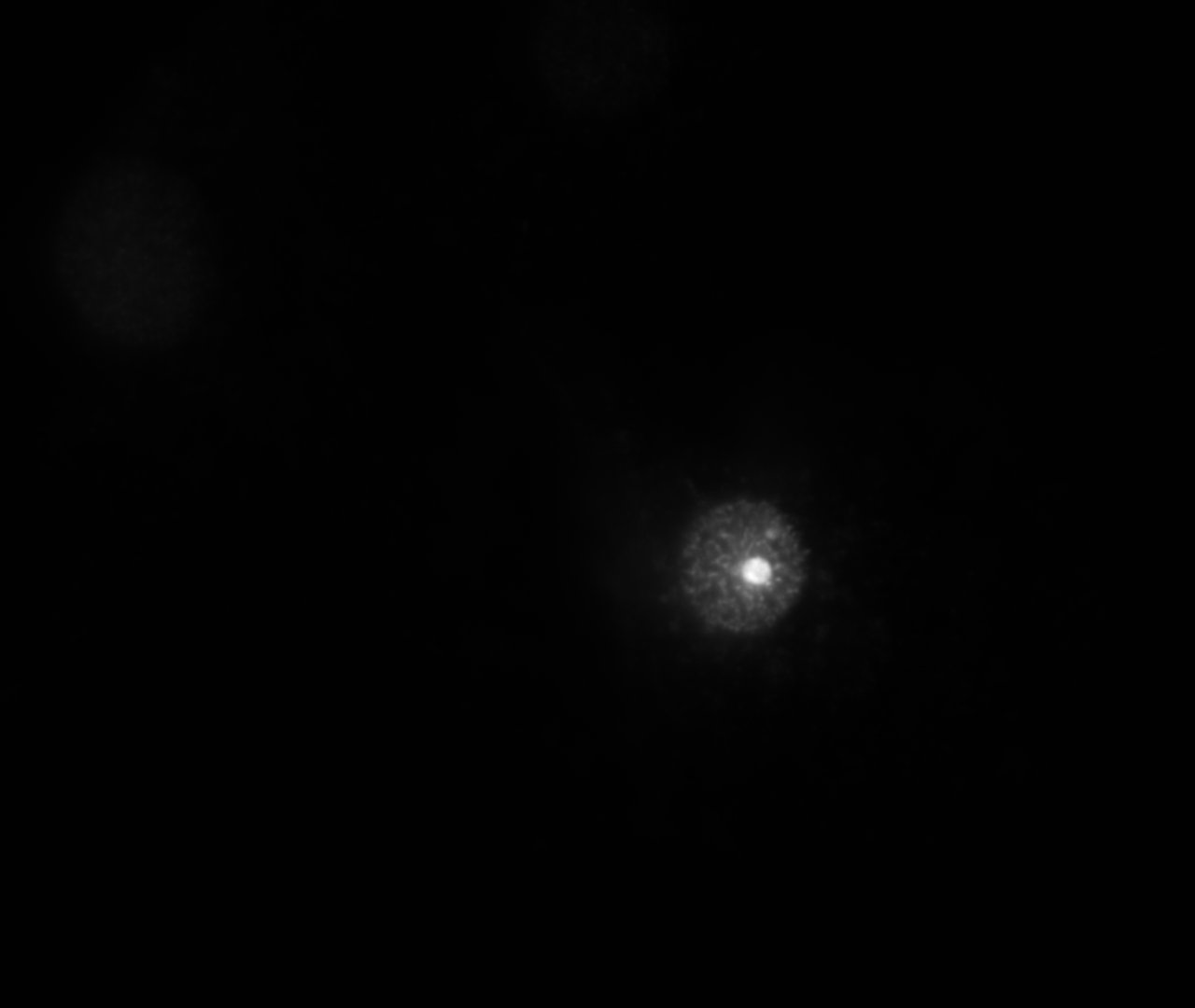

Supplement: Supplementary file 13 — Figures EV and Appendix Source Data [file 44318_2024_348_MOESM13_ESM.zip › SD figure EV and Appendix/Appendix Figure 2H/480-2.jpg]

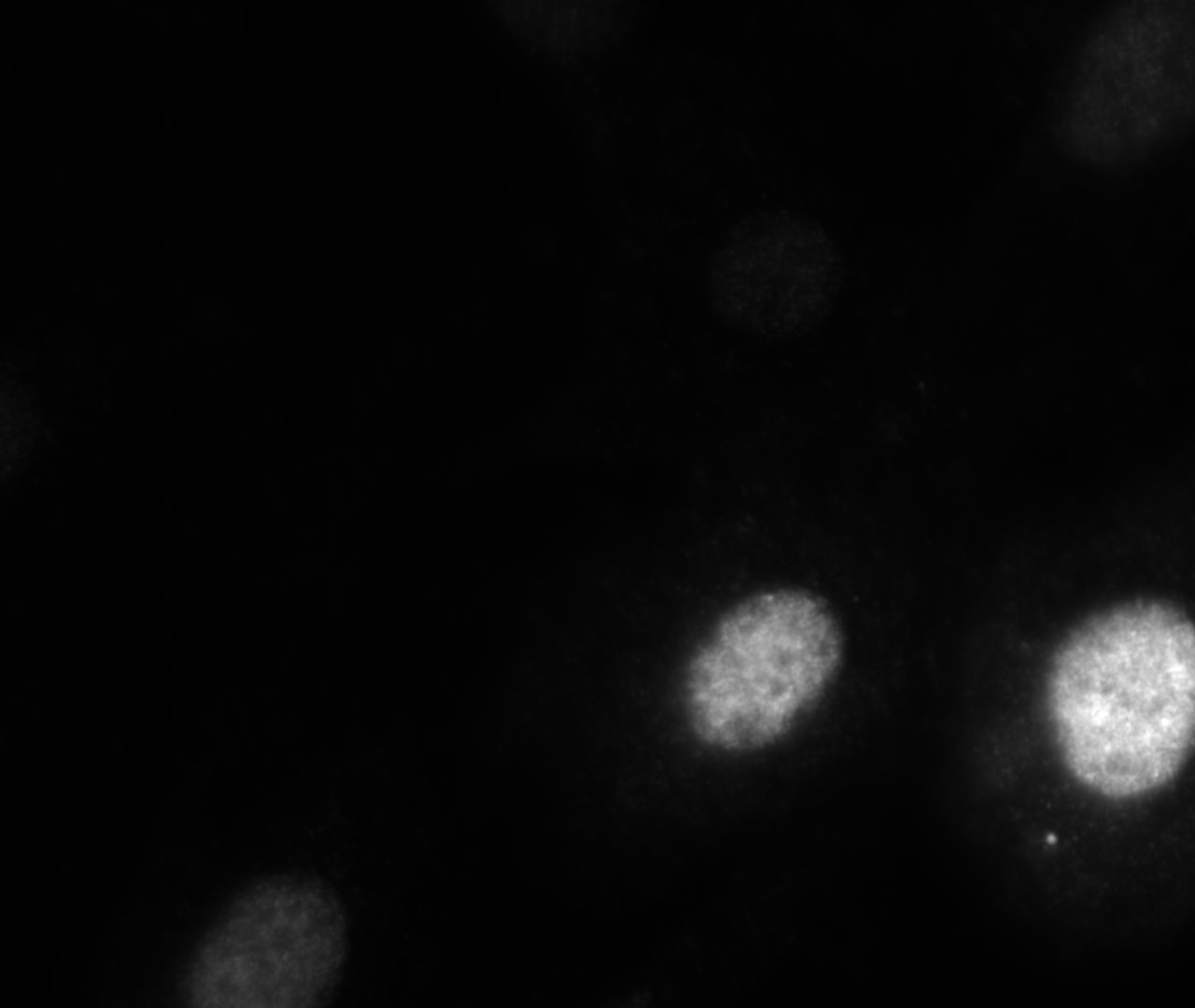

Supplement: Supplementary file 13 — Figures EV and Appendix Source Data [file 44318_2024_348_MOESM13_ESM.zip › SD figure EV and Appendix/Appendix Figure 2H/480-3.jpg]

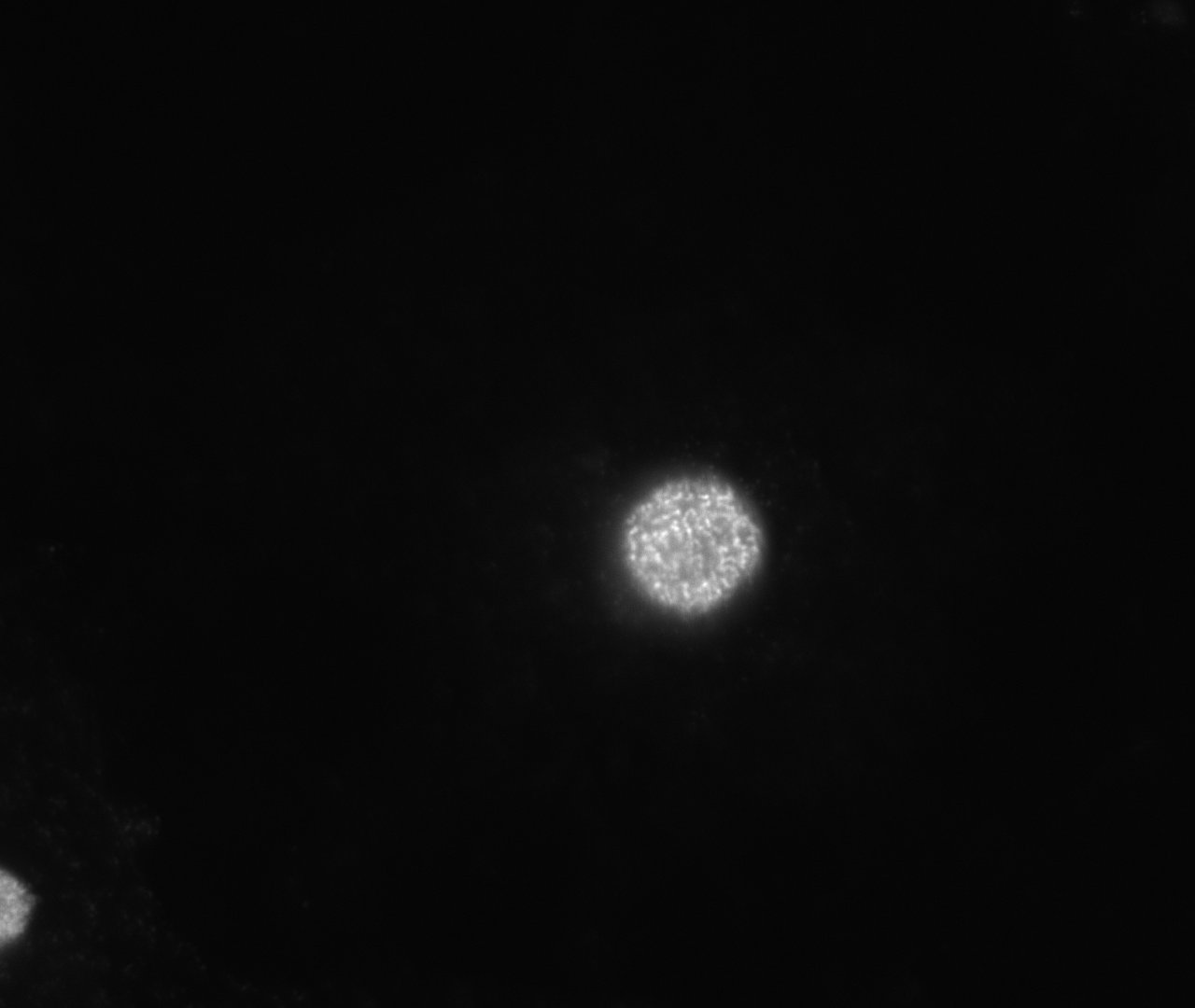

Supplement: Supplementary file 13 — Figures EV and Appendix Source Data [file 44318_2024_348_MOESM13_ESM.zip › SD figure EV and Appendix/Appendix Figure 2H/480-4.jpg]

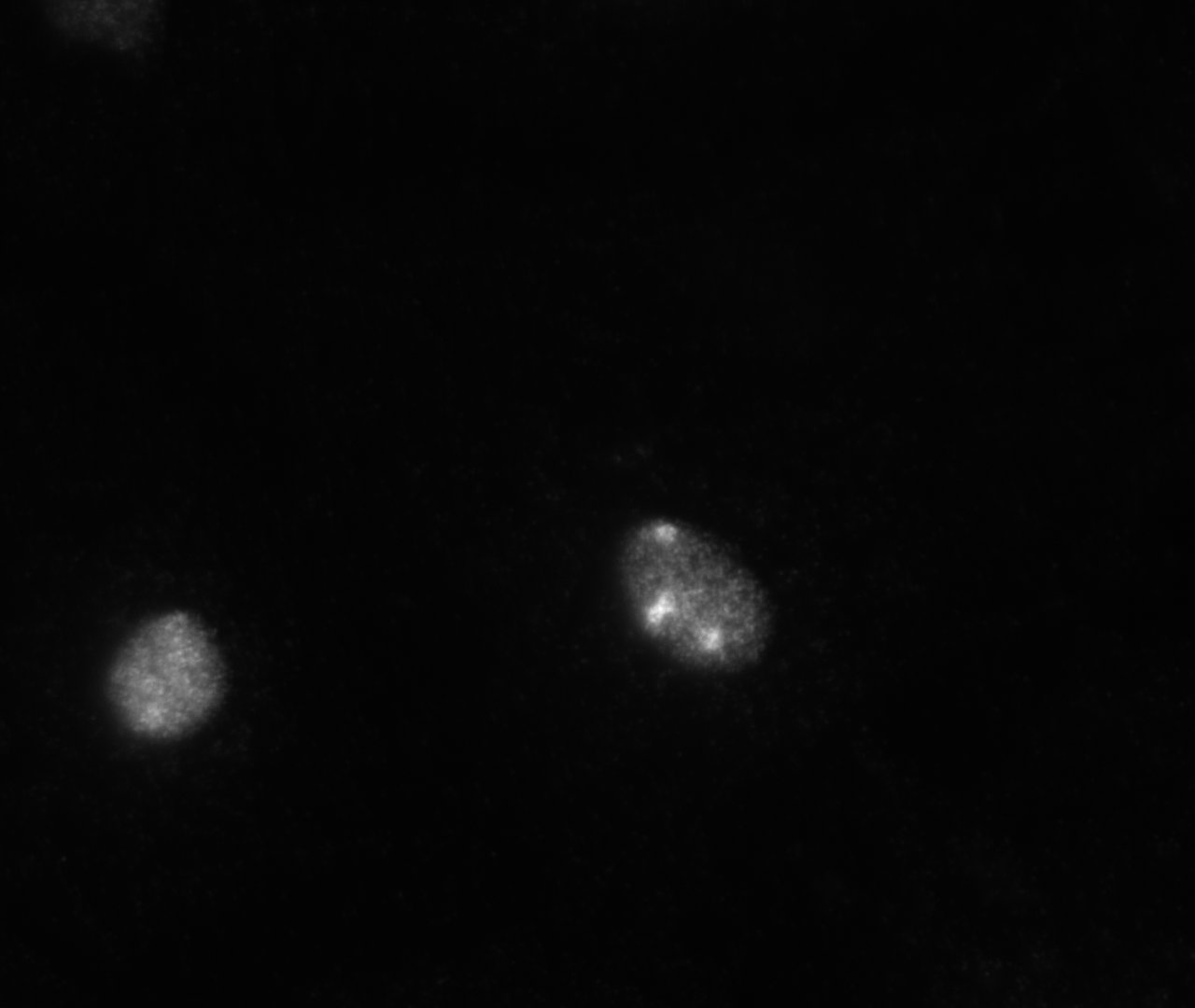

Supplement: Supplementary file 13 — Figures EV and Appendix Source Data [file 44318_2024_348_MOESM13_ESM.zip › SD figure EV and Appendix/Appendix Figure 2H/480-5.jpg]

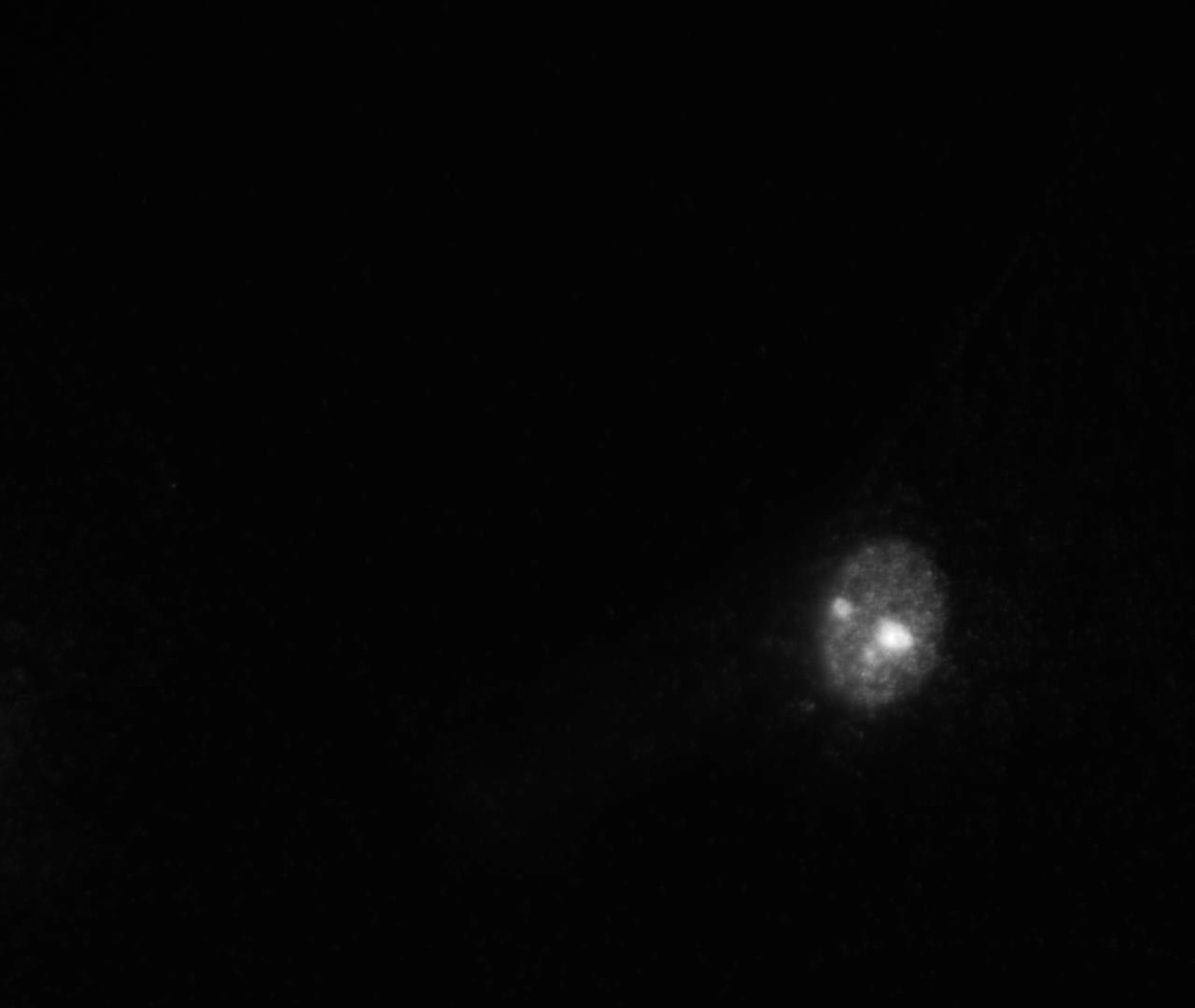

Supplement: Supplementary file 13 — Figures EV and Appendix Source Data [file 44318_2024_348_MOESM13_ESM.zip › SD figure EV and Appendix/Appendix Figure 2H/480-6.jpg]

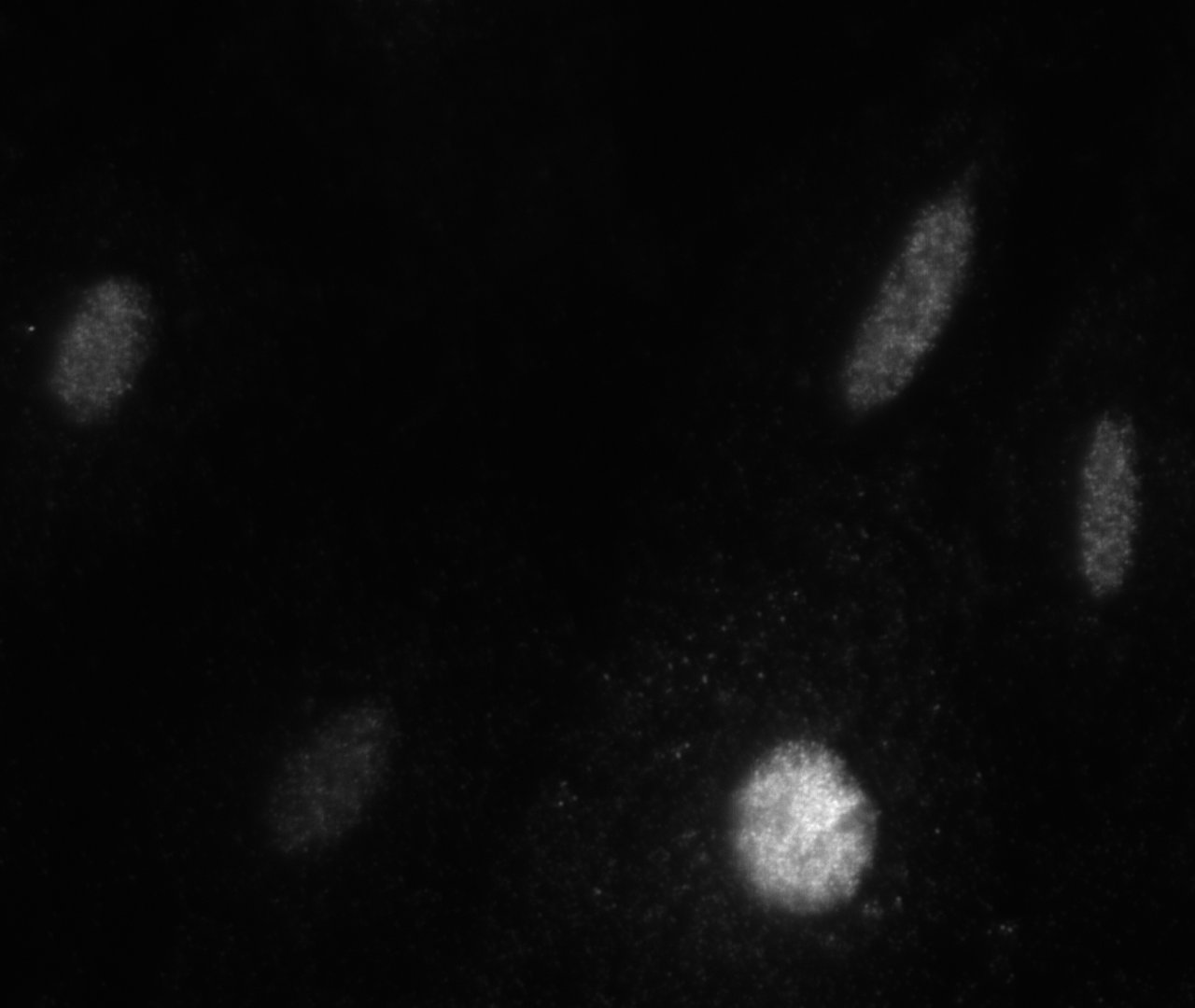

Supplement: Supplementary file 13 — Figures EV and Appendix Source Data [file 44318_2024_348_MOESM13_ESM.zip › SD figure EV and Appendix/Appendix Figure 2H/480-7.jpg]

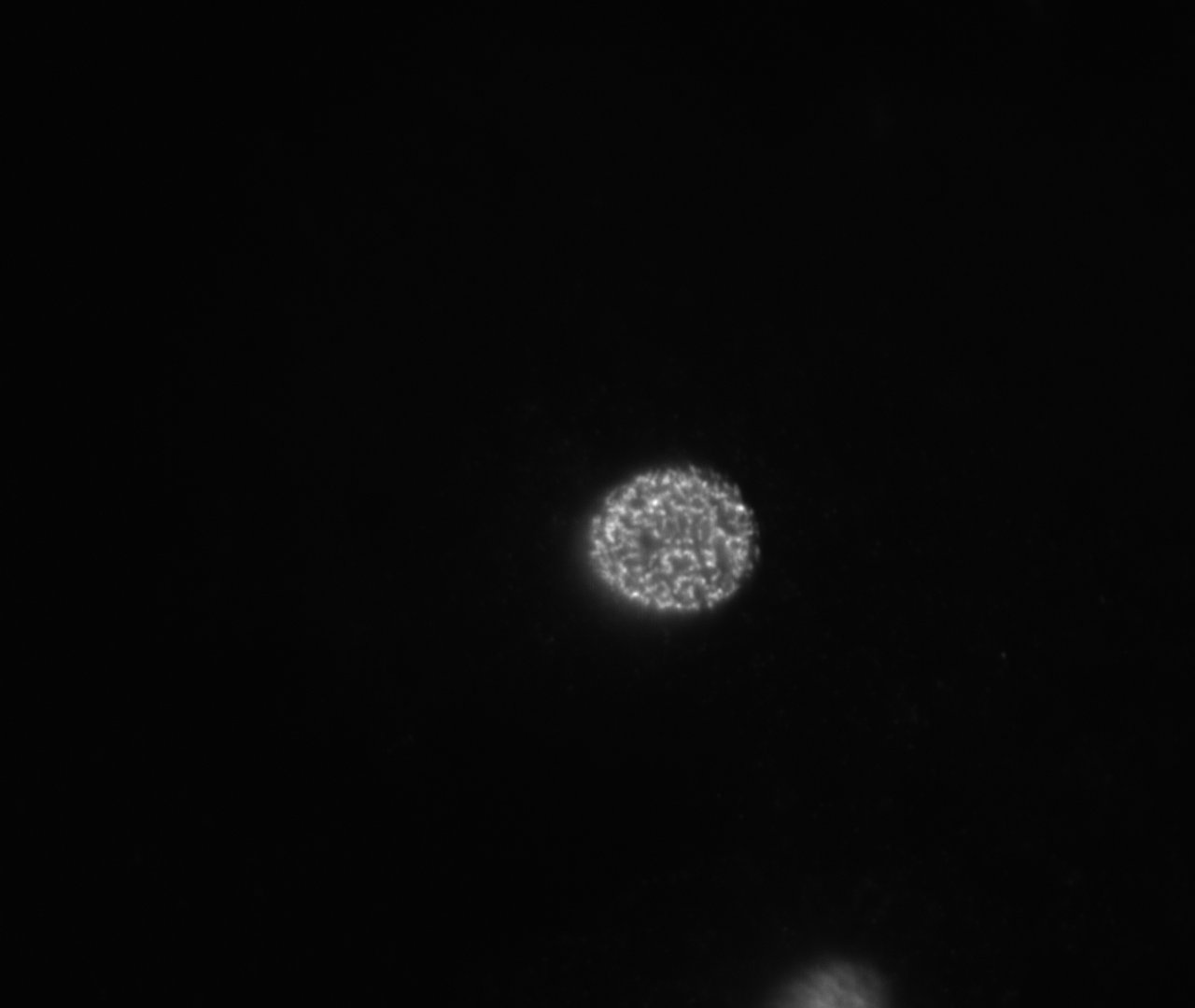

Supplement: Supplementary file 13 — Figures EV and Appendix Source Data [file 44318_2024_348_MOESM13_ESM.zip › SD figure EV and Appendix/Appendix Figure 2H/480-8.jpg]

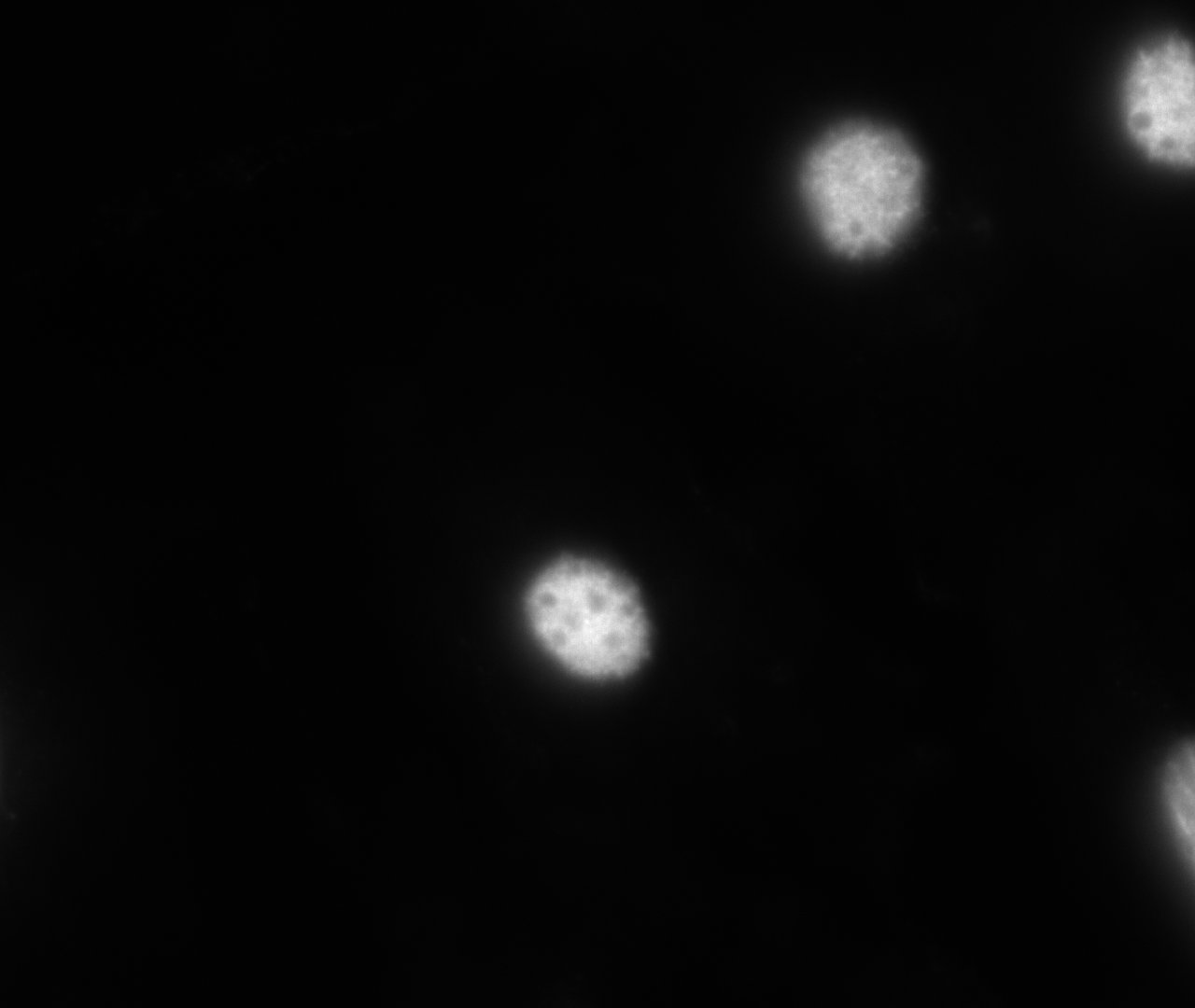

Supplement: Supplementary file 13 — Figures EV and Appendix Source Data [file 44318_2024_348_MOESM13_ESM.zip › SD figure EV and Appendix/Appendix Figure 2H/560.jpg]

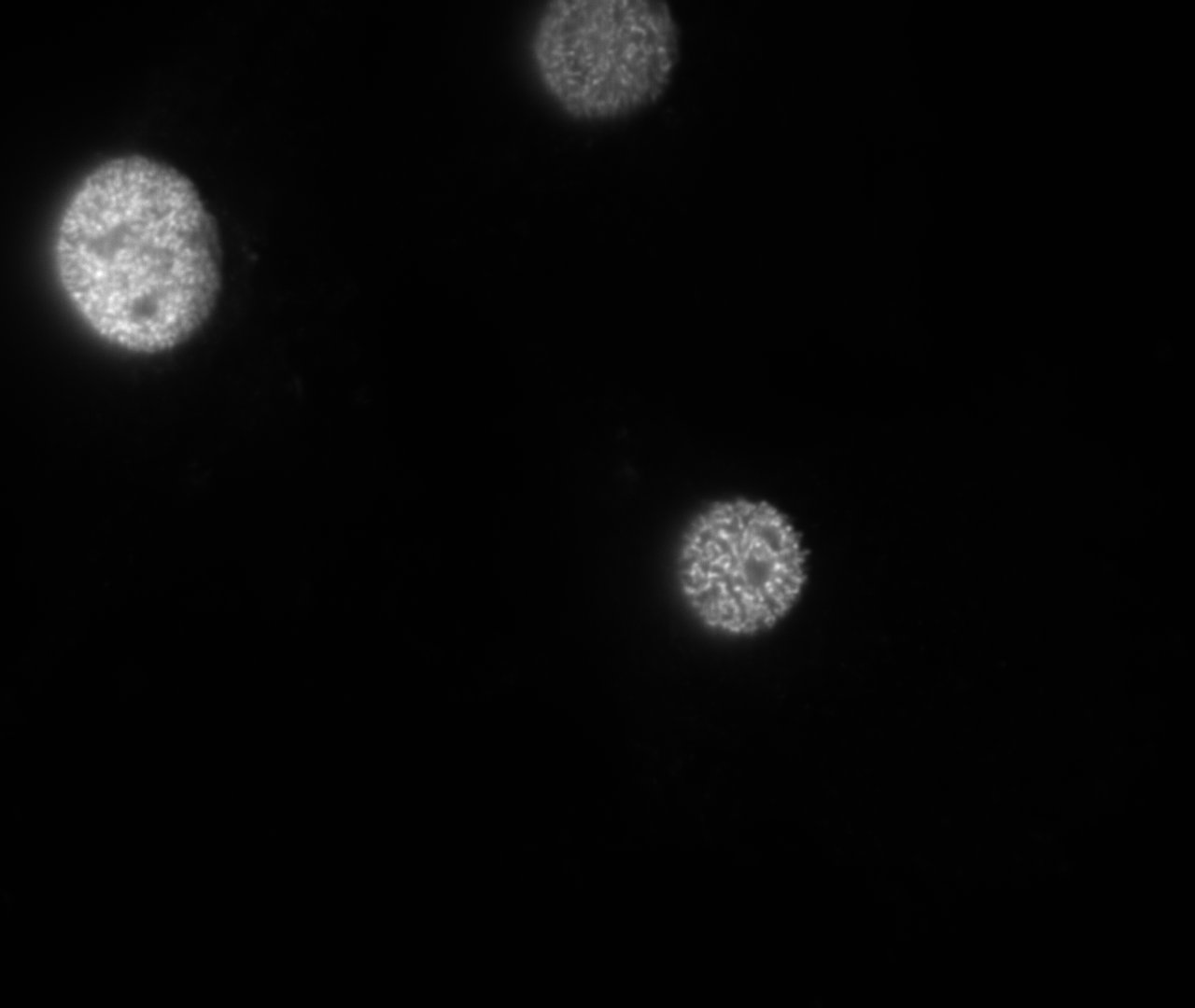

Supplement: Supplementary file 13 — Figures EV and Appendix Source Data [file 44318_2024_348_MOESM13_ESM.zip › SD figure EV and Appendix/Appendix Figure 2H/560-2.jpg]

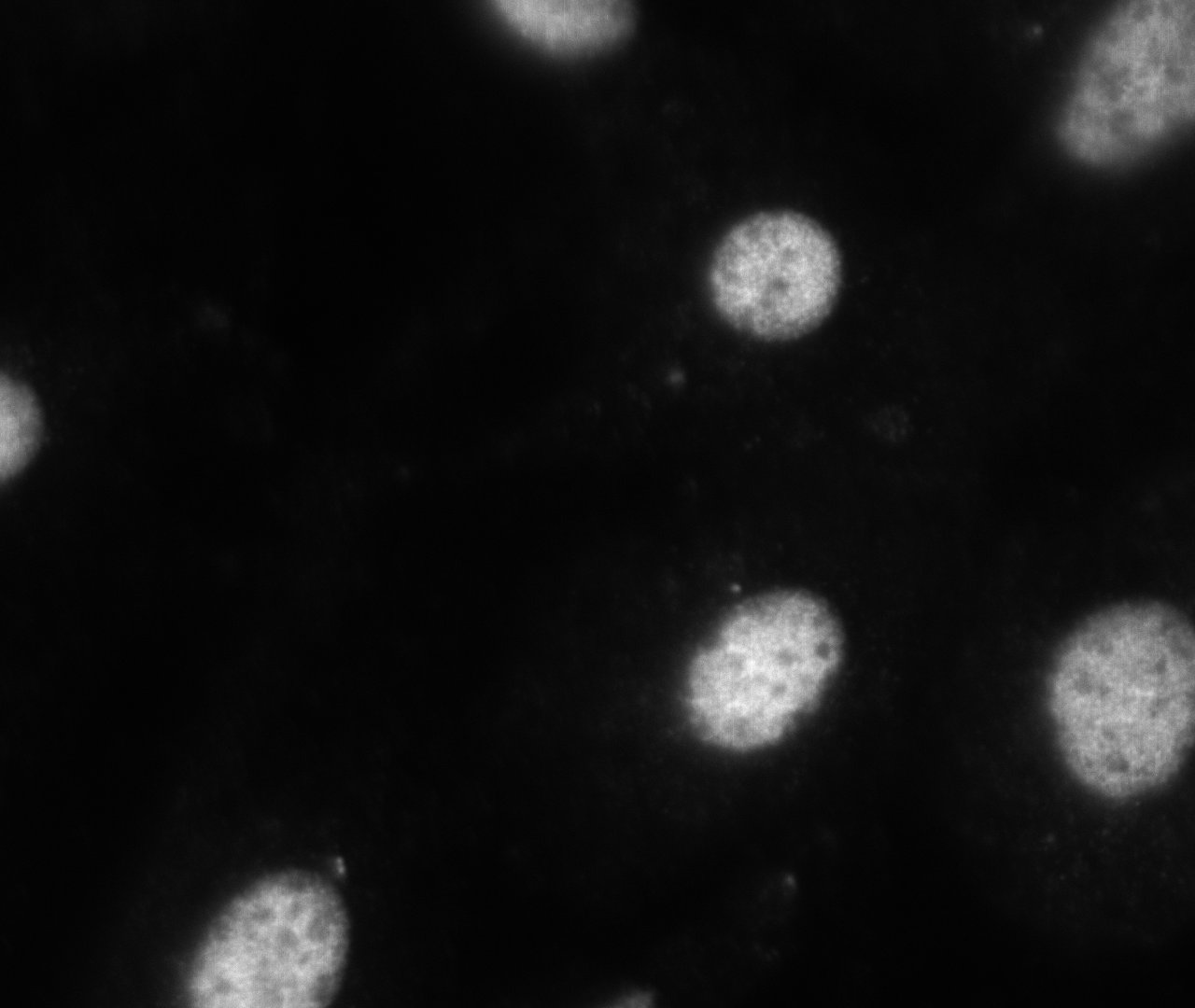

Supplement: Supplementary file 13 — Figures EV and Appendix Source Data [file 44318_2024_348_MOESM13_ESM.zip › SD figure EV and Appendix/Appendix Figure 2H/560-3.jpg]

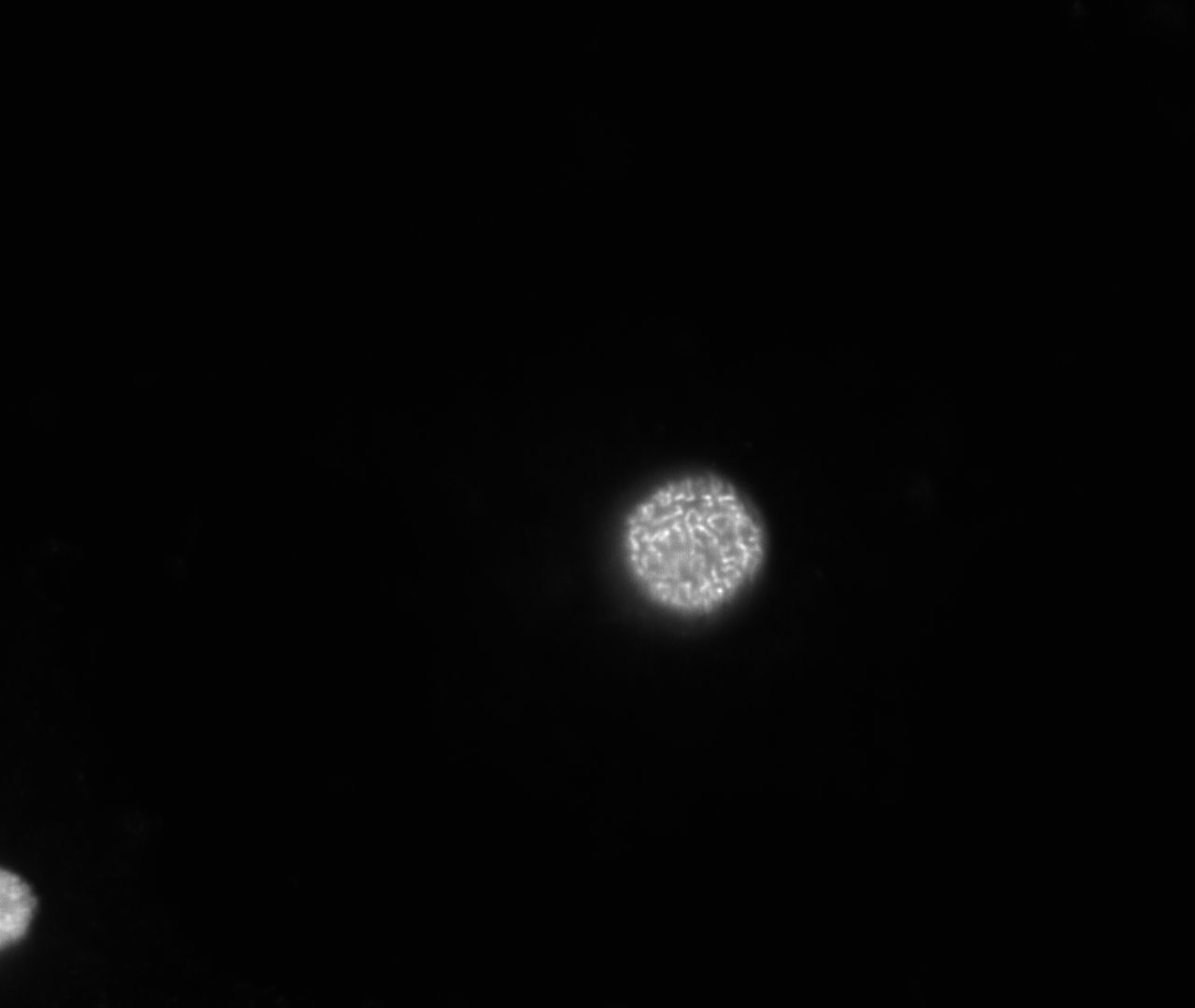

Supplement: Supplementary file 13 — Figures EV and Appendix Source Data [file 44318_2024_348_MOESM13_ESM.zip › SD figure EV and Appendix/Appendix Figure 2H/560-4.jpg]

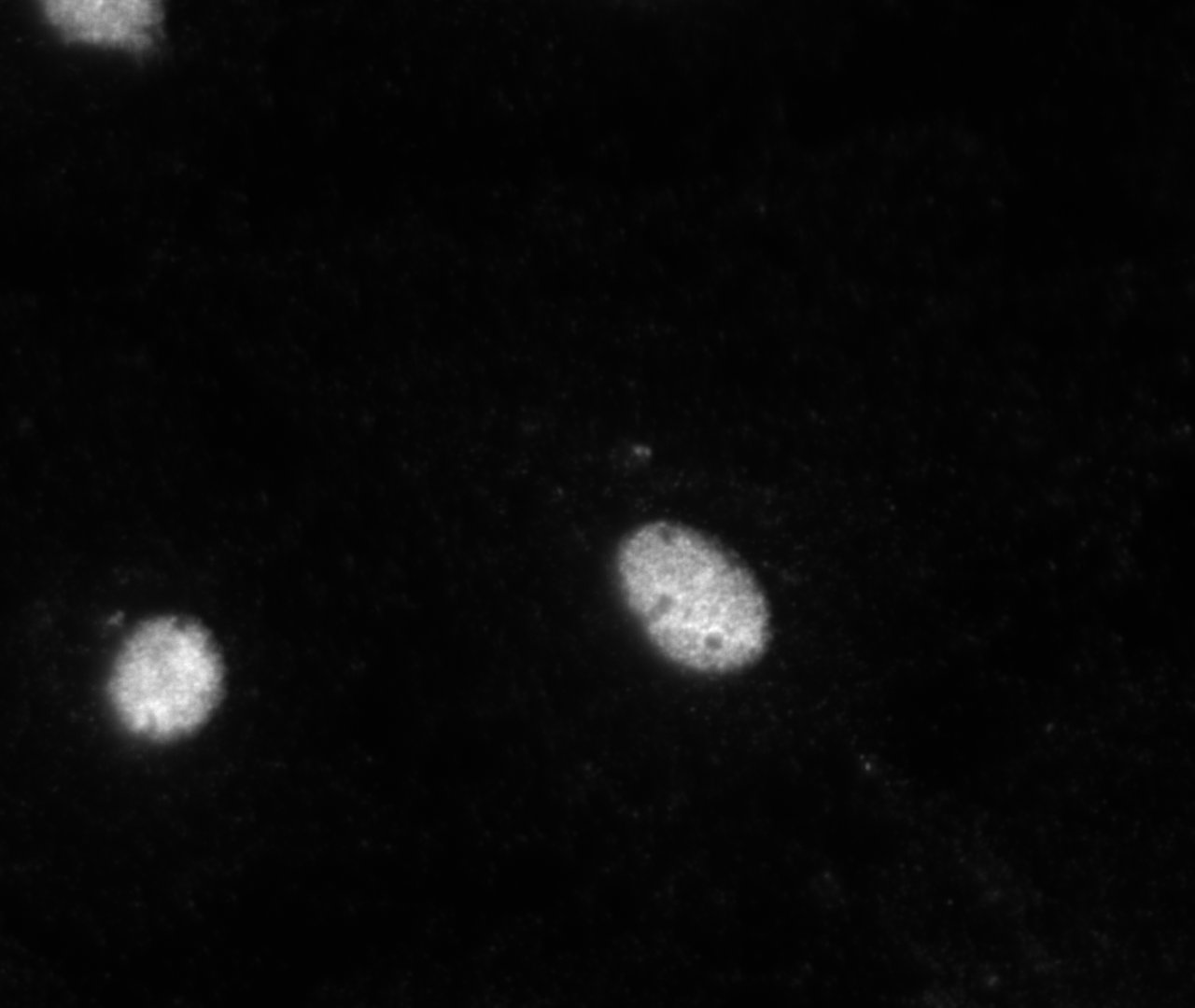

Supplement: Supplementary file 13 — Figures EV and Appendix Source Data [file 44318_2024_348_MOESM13_ESM.zip › SD figure EV and Appendix/Appendix Figure 2H/560-5.jpg]

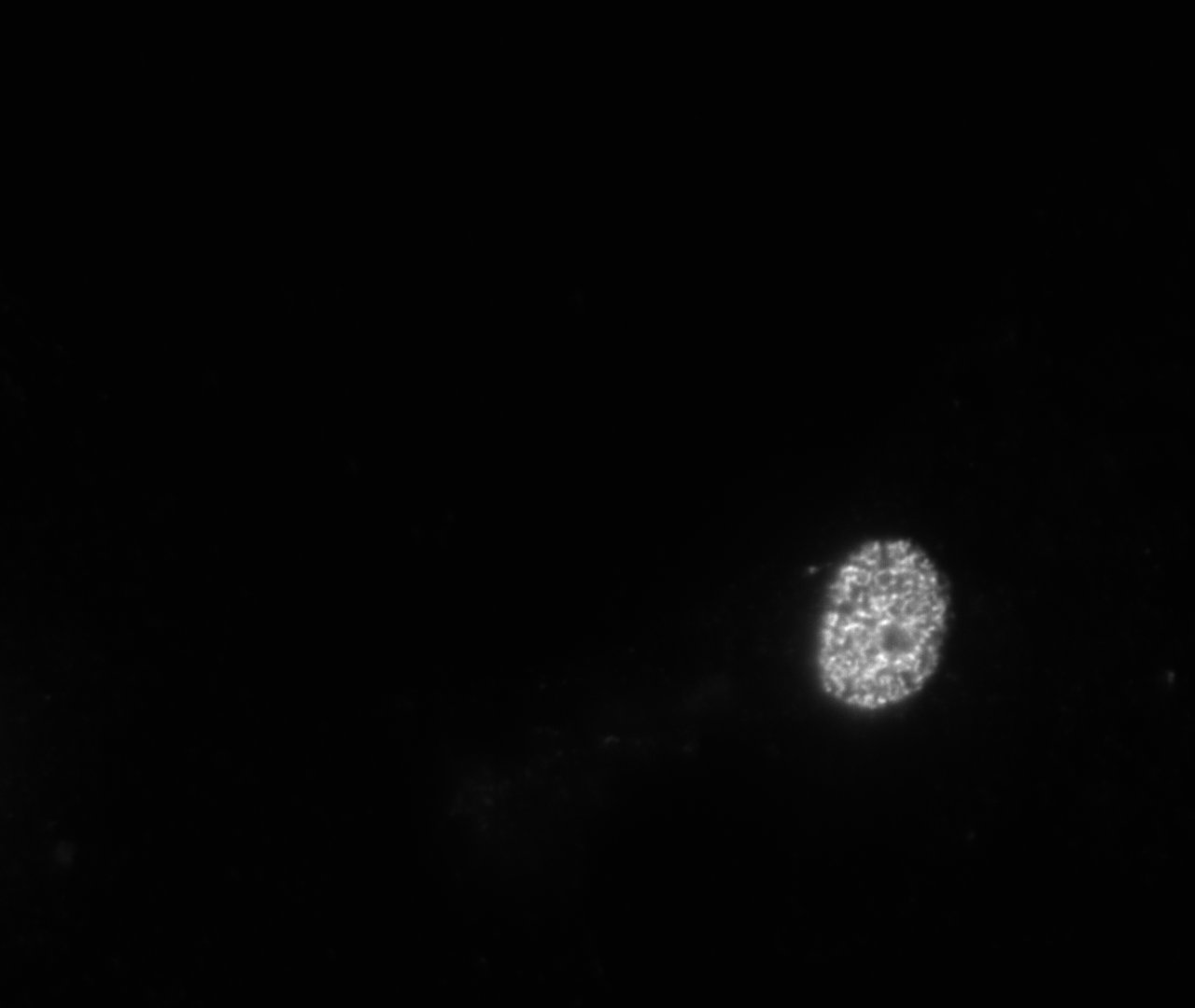

Supplement: Supplementary file 13 — Figures EV and Appendix Source Data [file 44318_2024_348_MOESM13_ESM.zip › SD figure EV and Appendix/Appendix Figure 2H/560-6.jpg]

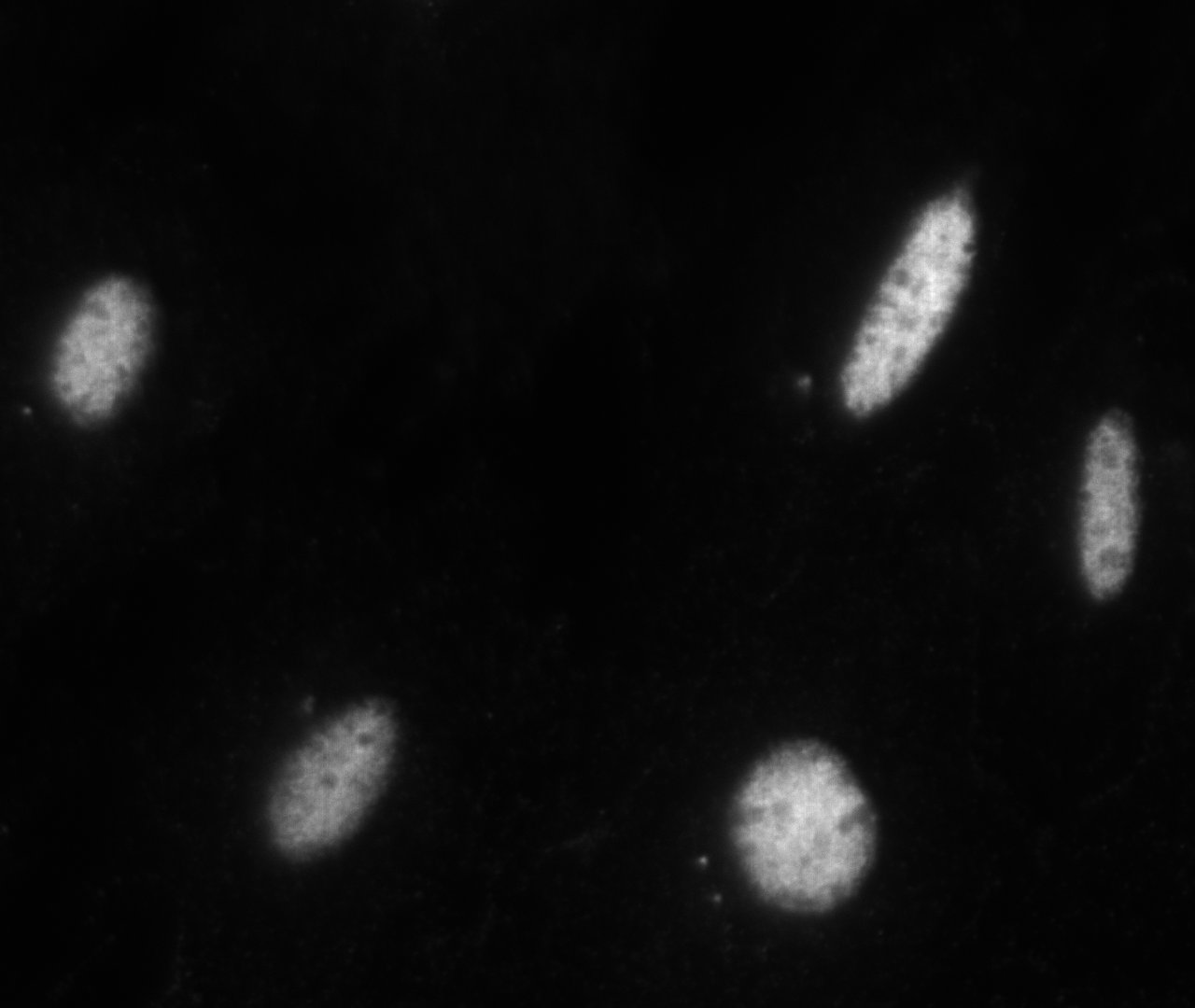

Supplement: Supplementary file 13 — Figures EV and Appendix Source Data [file 44318_2024_348_MOESM13_ESM.zip › SD figure EV and Appendix/Appendix Figure 2H/560-7.jpg]

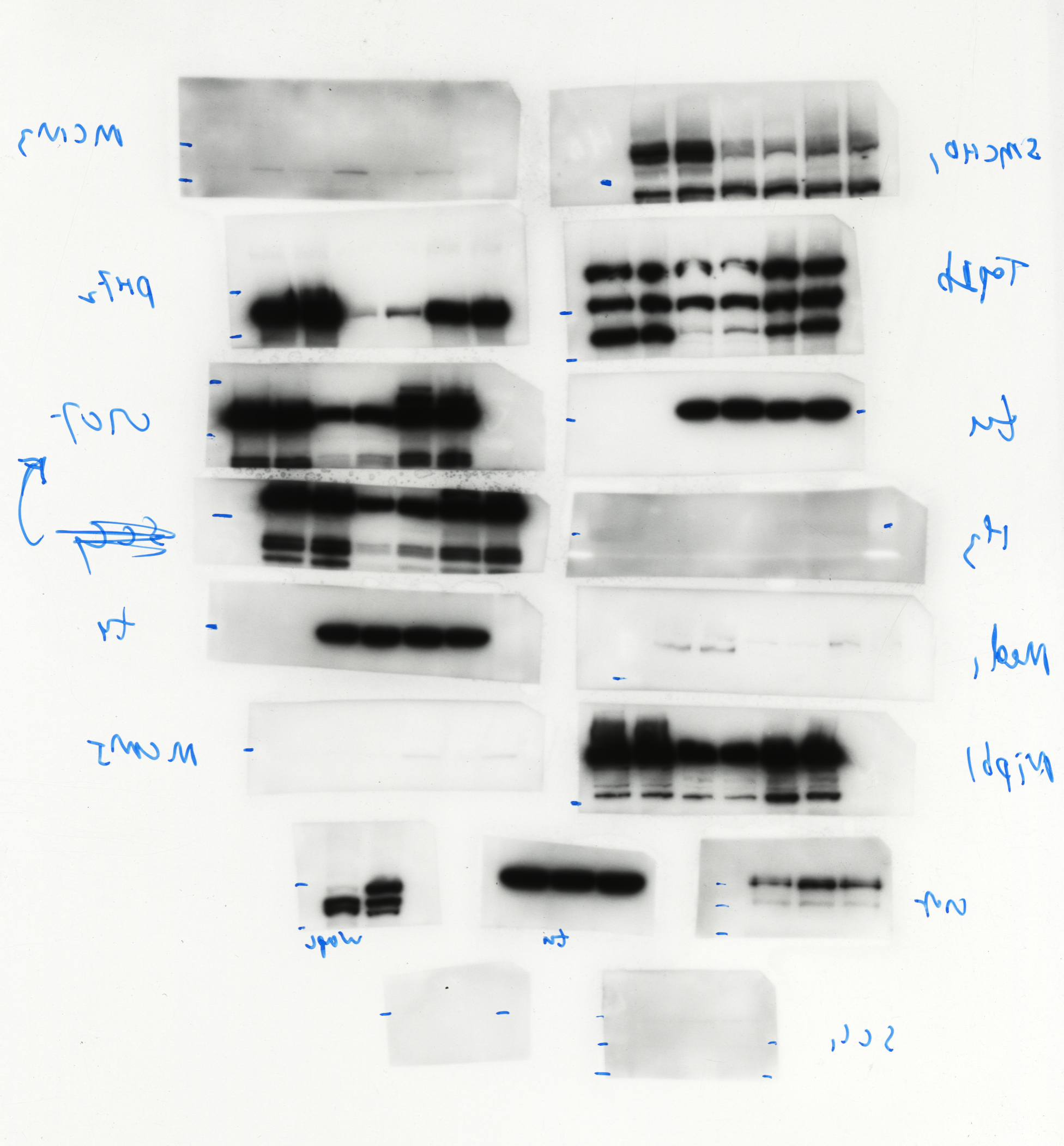

Supplement: Supplementary file 13 — Figures EV and Appendix Source Data [file 44318_2024_348_MOESM13_ESM.zip › SD figure EV and Appendix/Appendix Figure 1D/Untitled-4.tif]

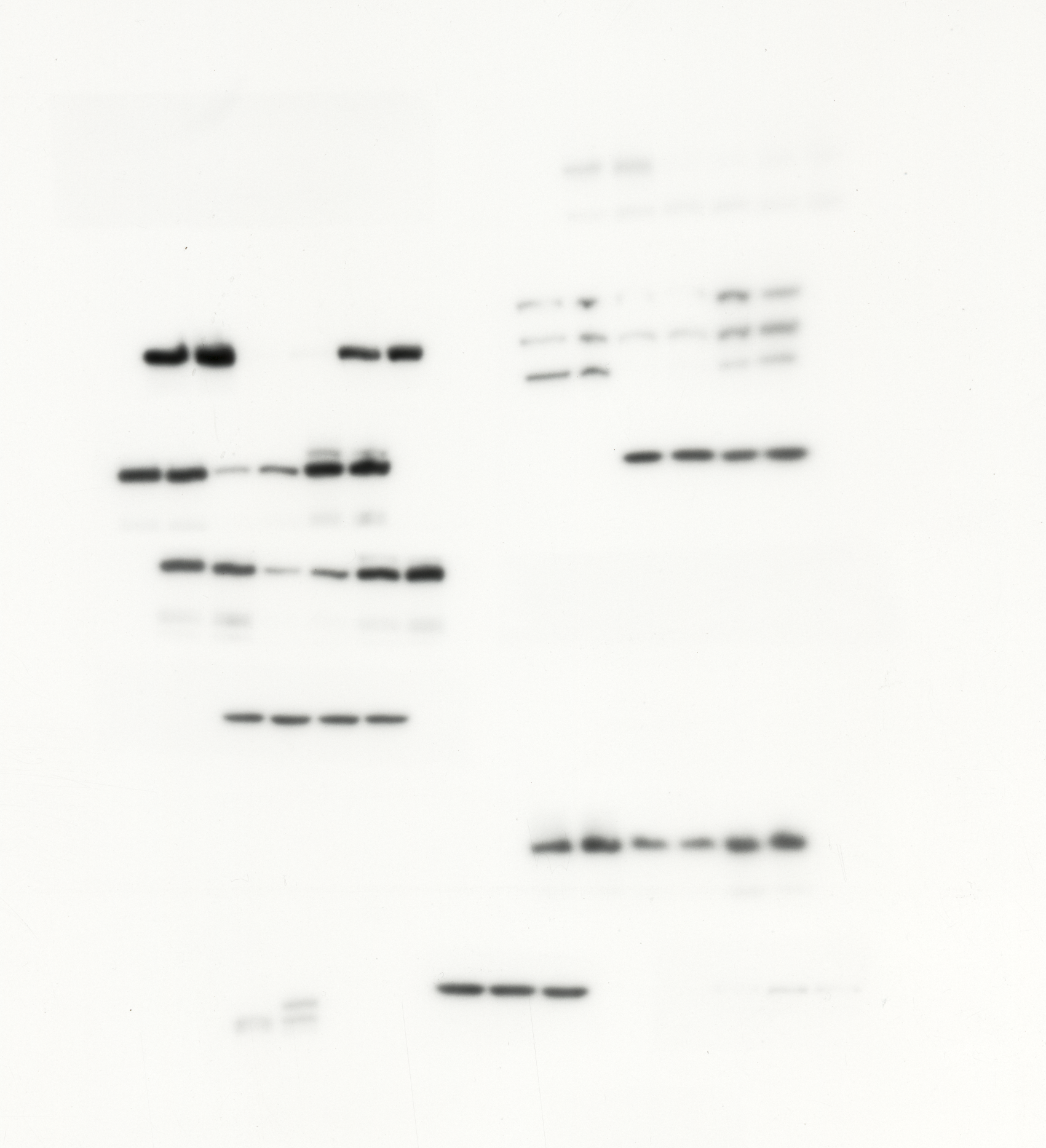

Supplement: Supplementary file 13 — Figures EV and Appendix Source Data [file 44318_2024_348_MOESM13_ESM.zip › SD figure EV and Appendix/Appendix Figure 1D/Untitled-5.tif]

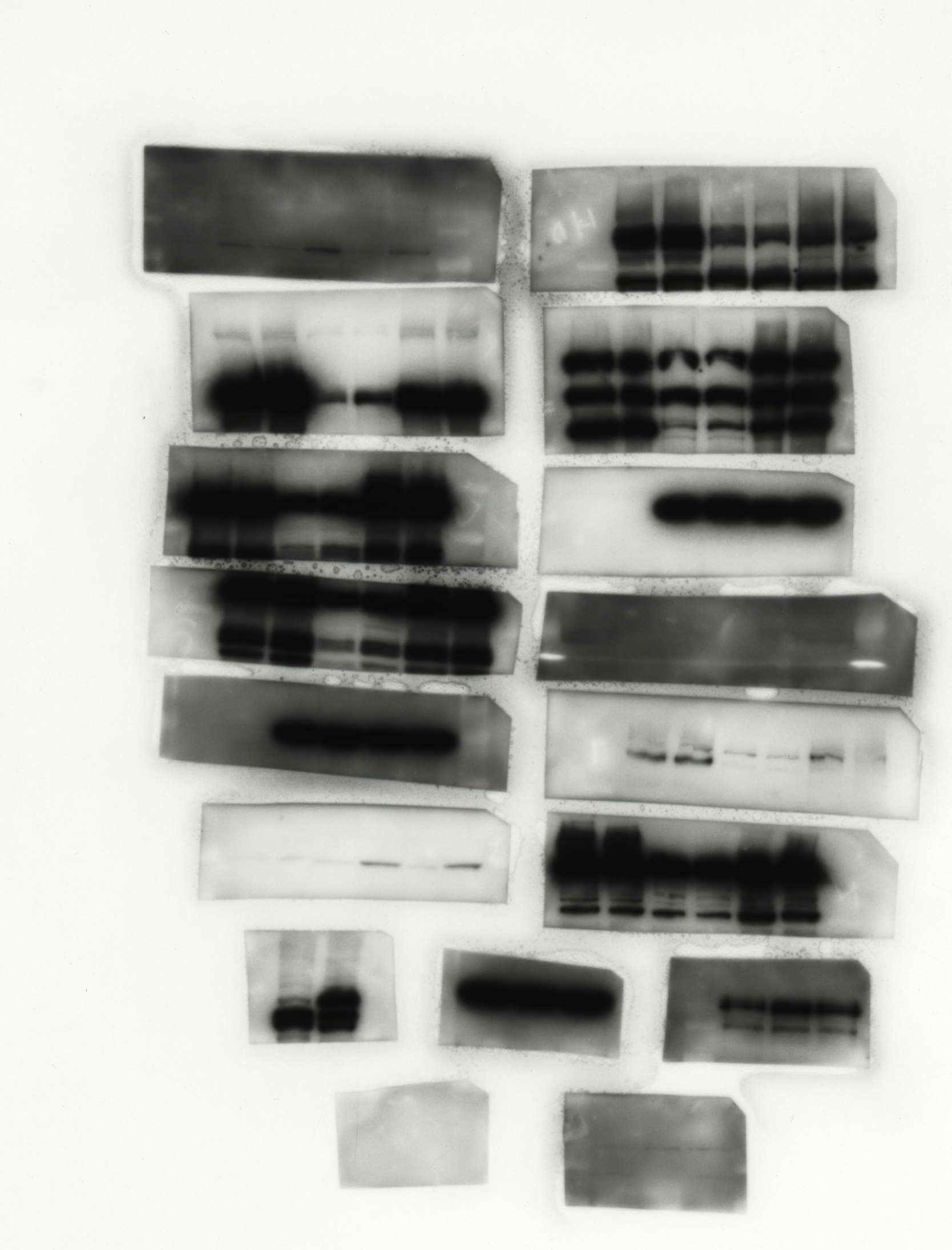

Supplement: Supplementary file 13 — Figures EV and Appendix Source Data [file 44318_2024_348_MOESM13_ESM.zip › SD figure EV and Appendix/Appendix Figure 1D/Untitled-6.tif]

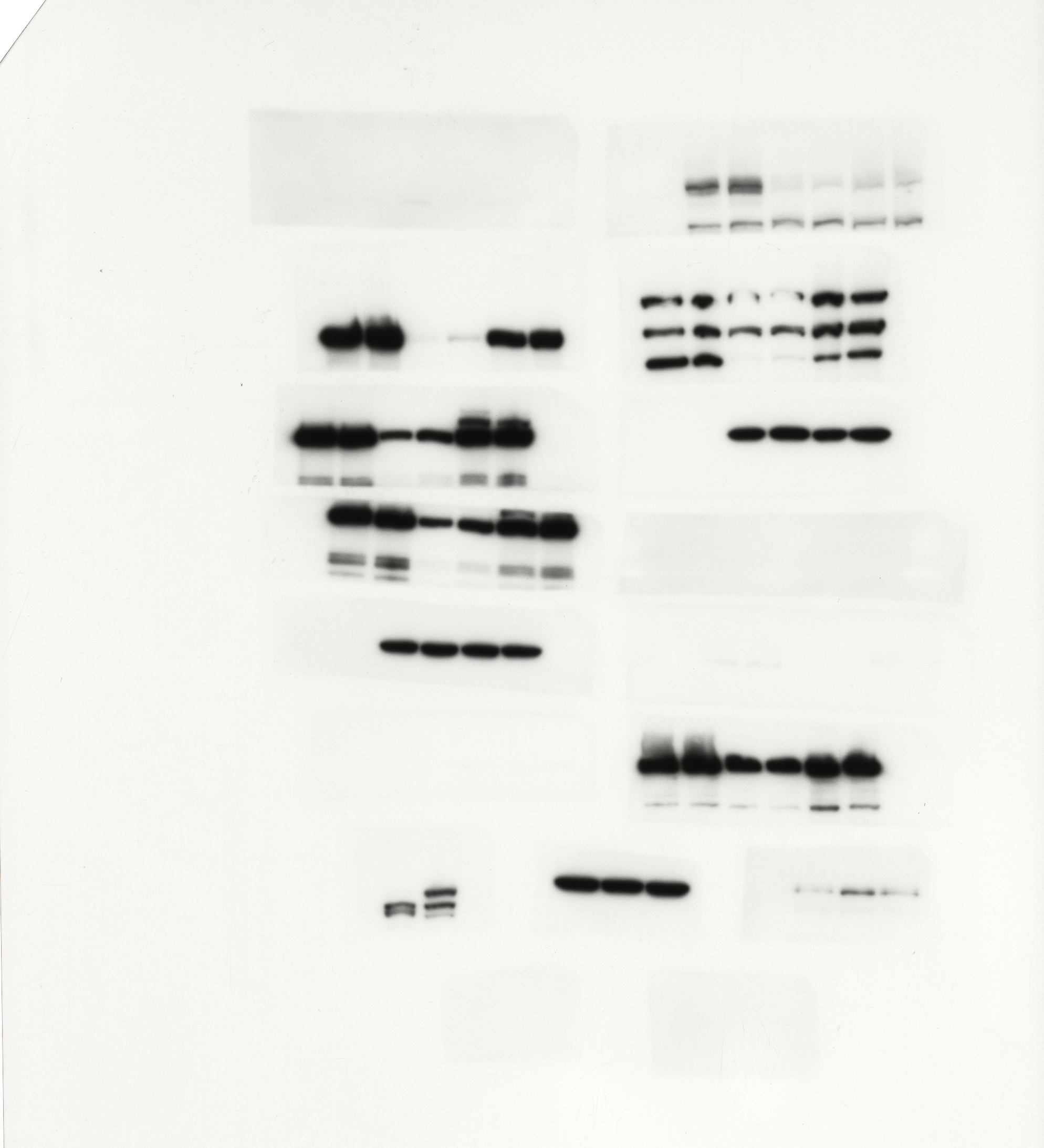

Supplement: Supplementary file 13 — Figures EV and Appendix Source Data [file 44318_2024_348_MOESM13_ESM.zip › SD figure EV and Appendix/Appendix Figure 1D/Untitled-7.tif]

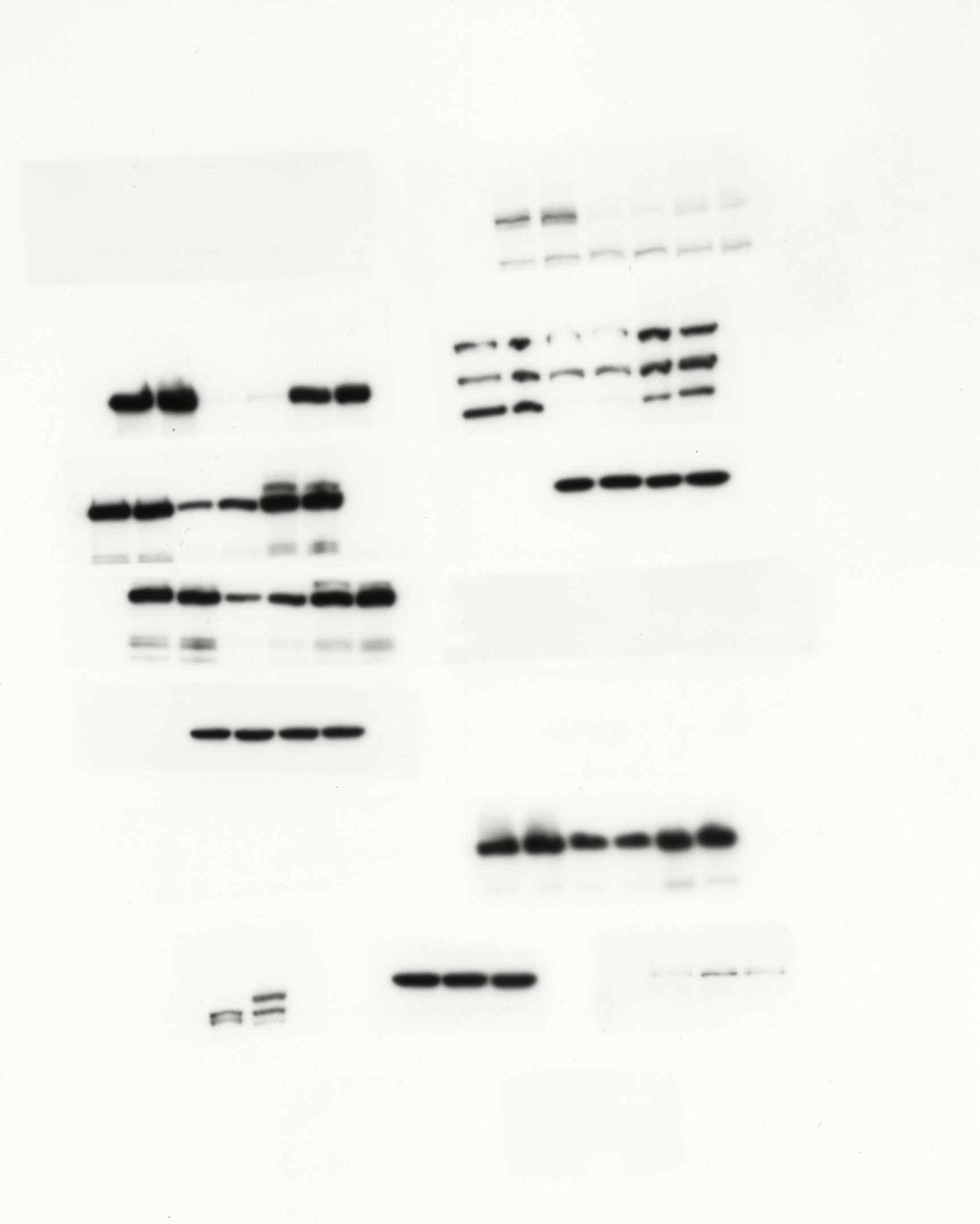

Supplement: Supplementary file 13 — Figures EV and Appendix Source Data [file 44318_2024_348_MOESM13_ESM.zip › SD figure EV and Appendix/Appendix Figure 1D/Untitled-8.tif]

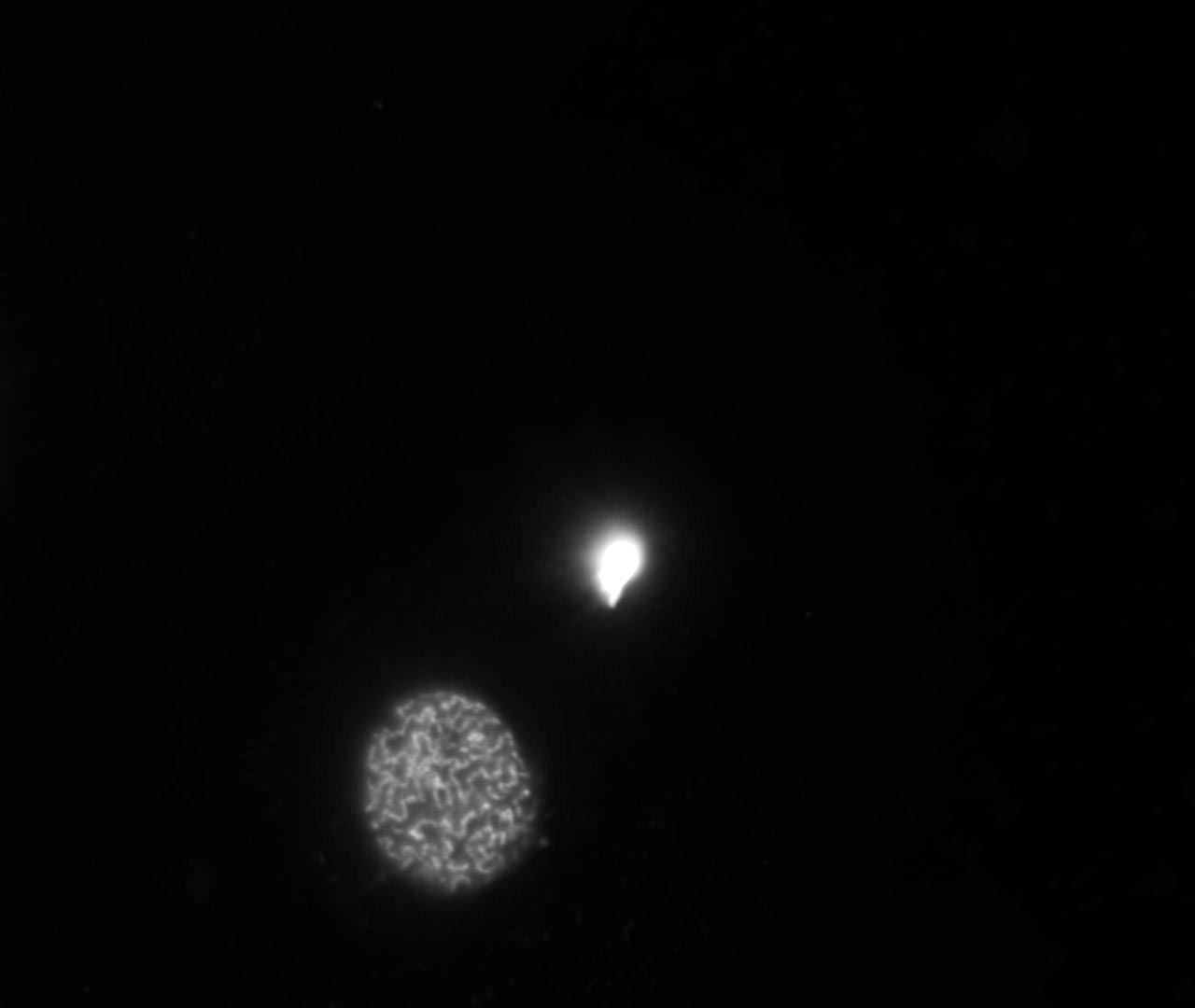

Supplement: Supplementary file 13 — Figures EV and Appendix Source Data [file 44318_2024_348_MOESM13_ESM.zip › SD figure EV and Appendix/Appendix Figure 2G/560-6.jpg]

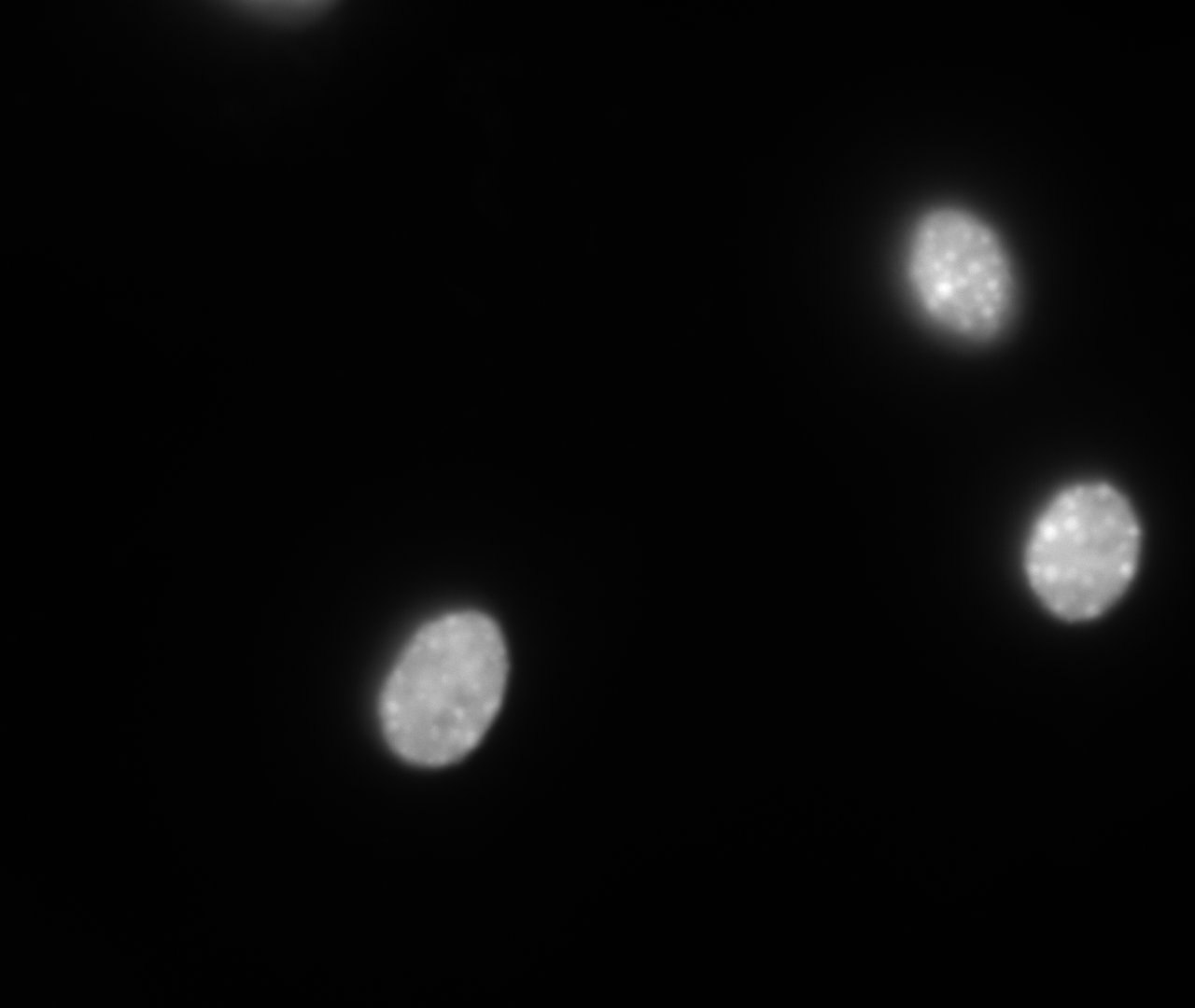

Supplement: Supplementary file 13 — Figures EV and Appendix Source Data [file 44318_2024_348_MOESM13_ESM.zip › SD figure EV and Appendix/Appendix Figure 2G/360.jpg]

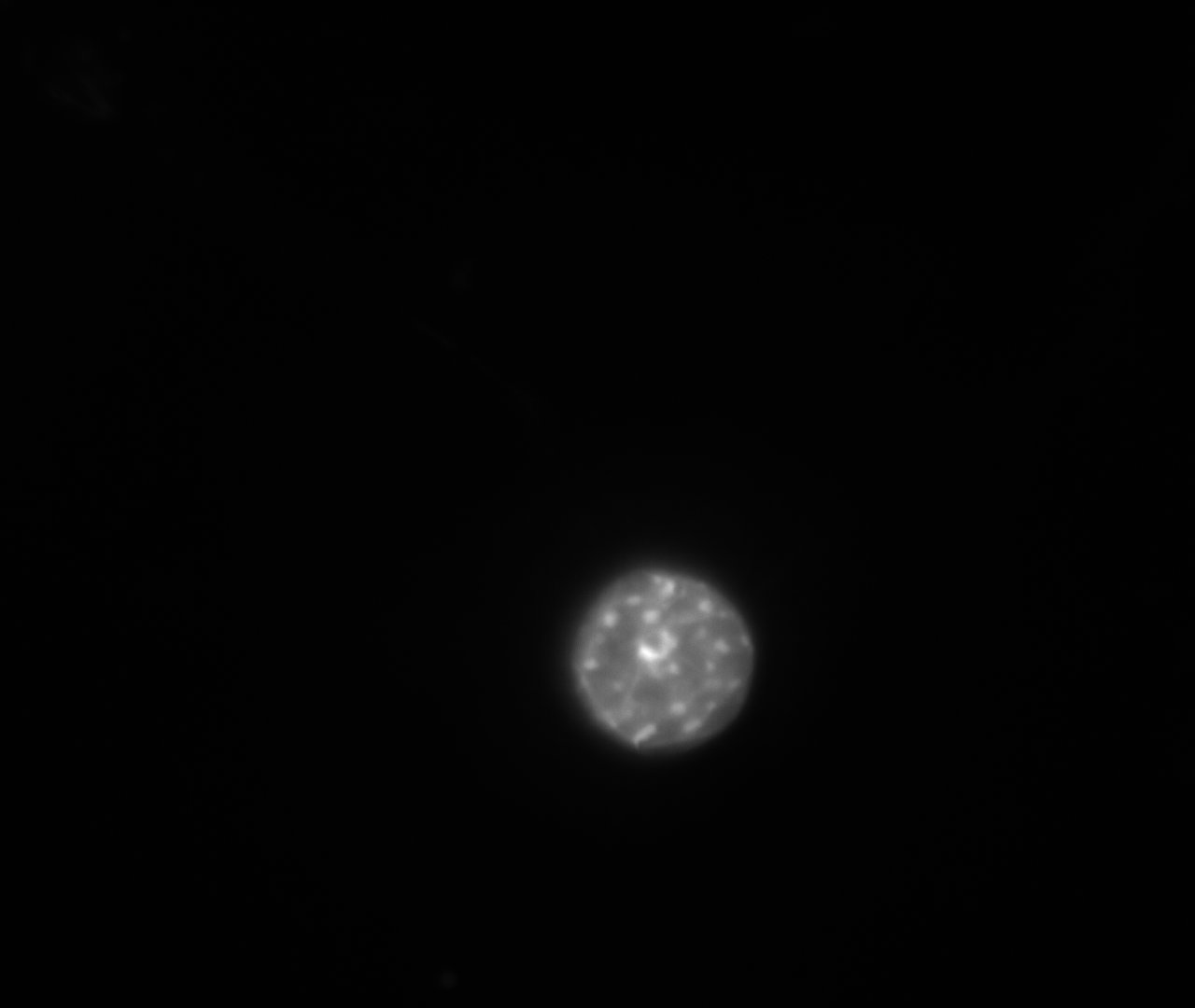

Supplement: Supplementary file 13 — Figures EV and Appendix Source Data [file 44318_2024_348_MOESM13_ESM.zip › SD figure EV and Appendix/Appendix Figure 2G/360-2.jpg]

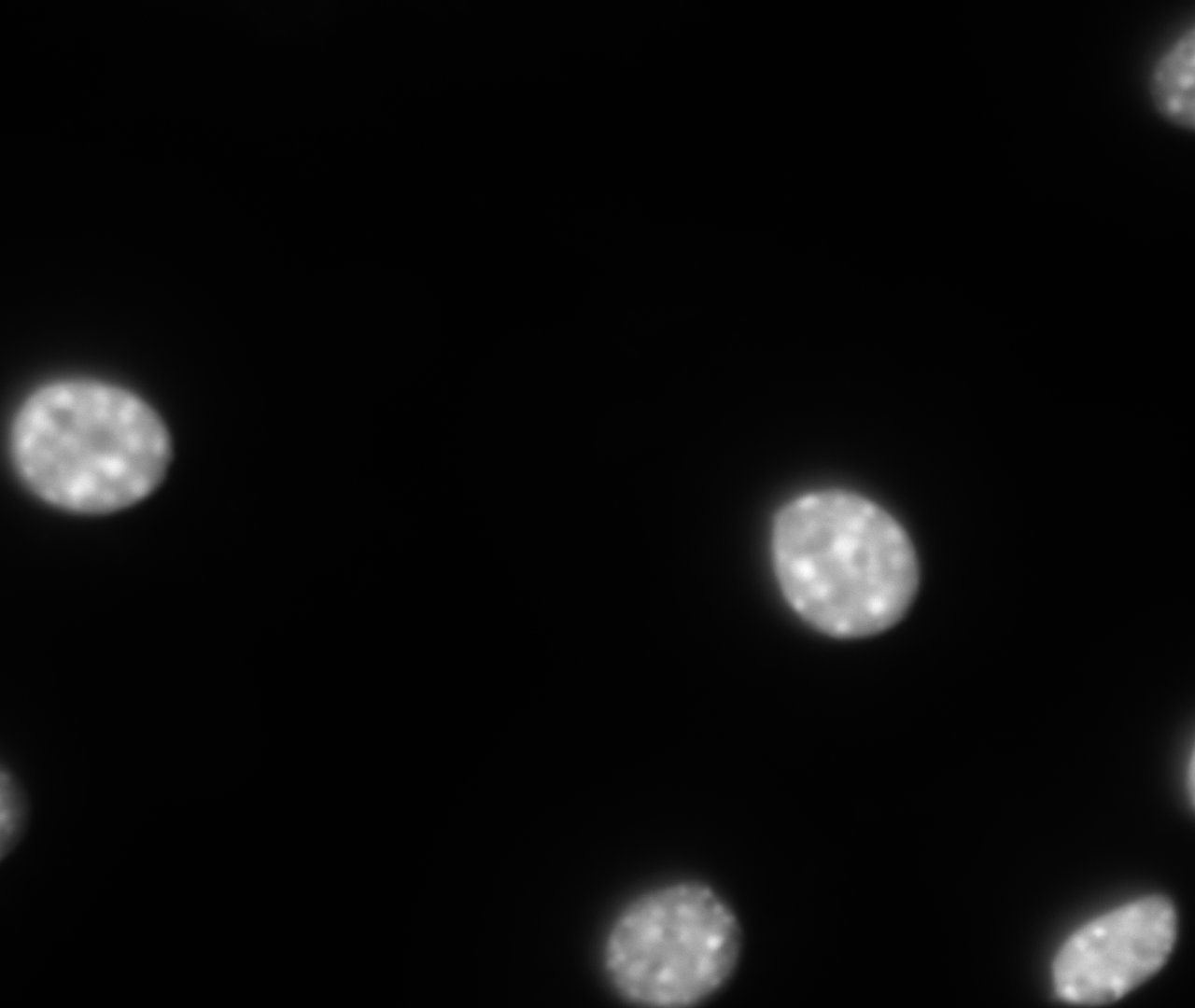

Supplement: Supplementary file 13 — Figures EV and Appendix Source Data [file 44318_2024_348_MOESM13_ESM.zip › SD figure EV and Appendix/Appendix Figure 2G/360-3.jpg]

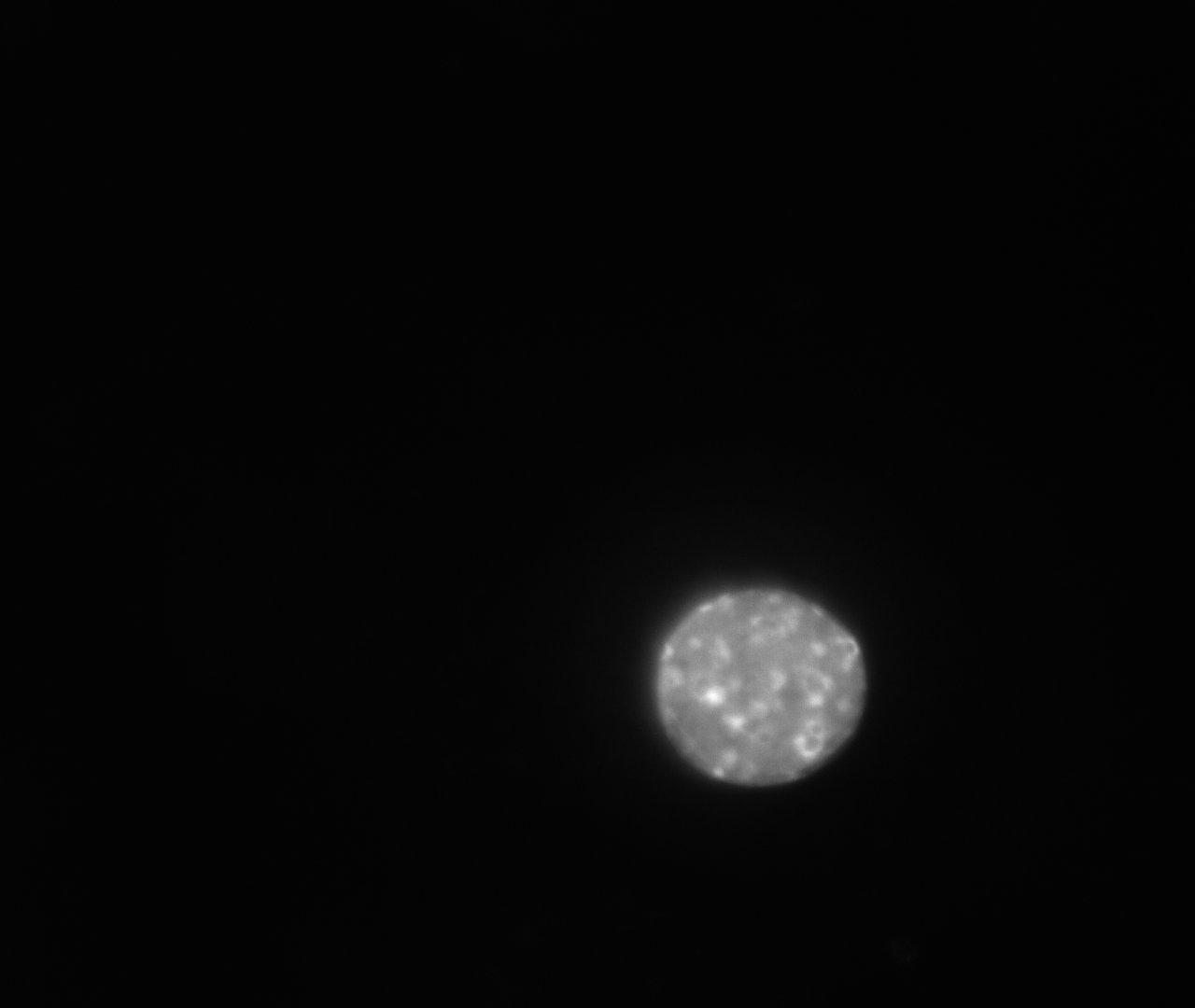

Supplement: Supplementary file 13 — Figures EV and Appendix Source Data [file 44318_2024_348_MOESM13_ESM.zip › SD figure EV and Appendix/Appendix Figure 2G/360-4.jpg]

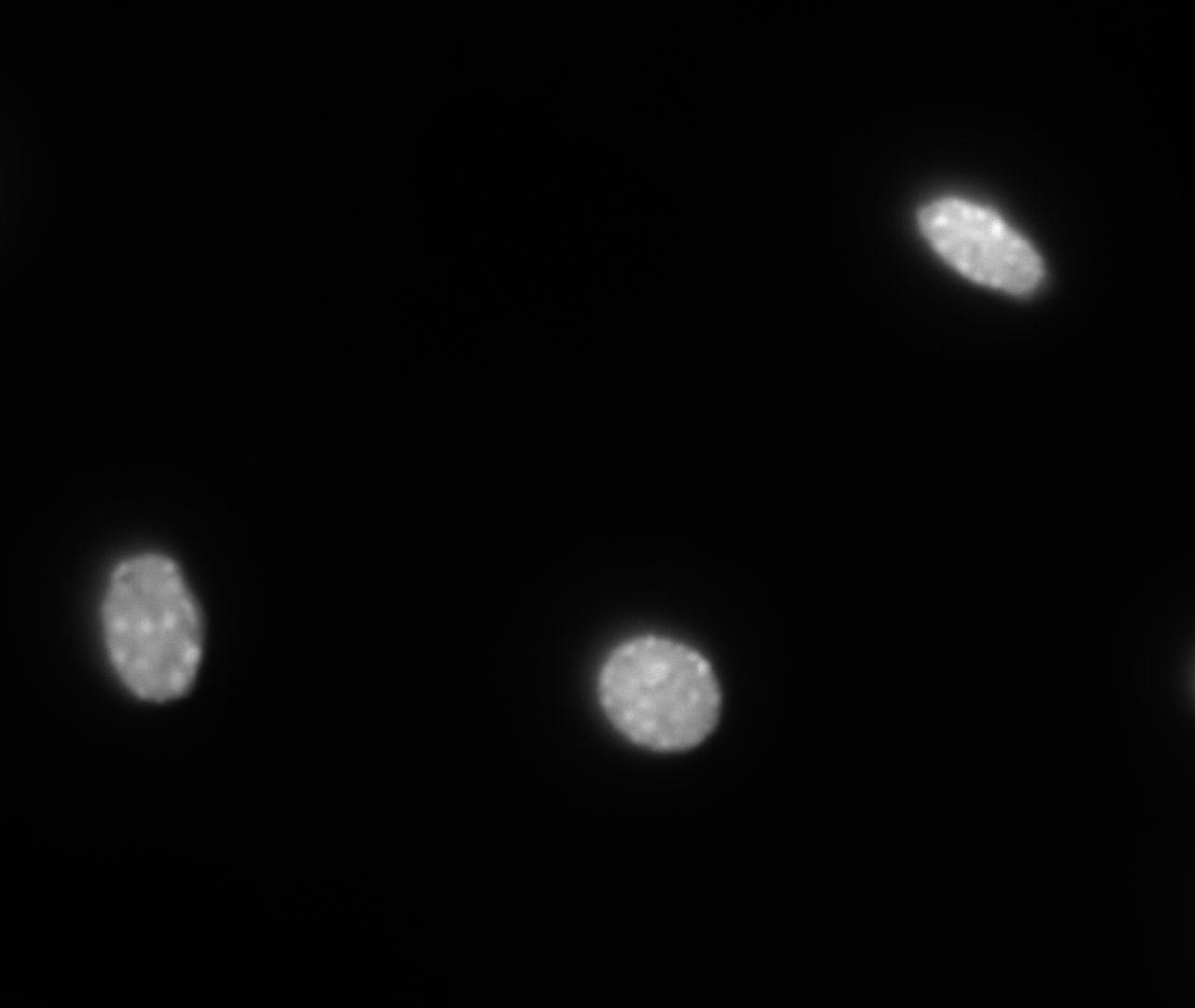

Supplement: Supplementary file 13 — Figures EV and Appendix Source Data [file 44318_2024_348_MOESM13_ESM.zip › SD figure EV and Appendix/Appendix Figure 2G/360-5.jpg]

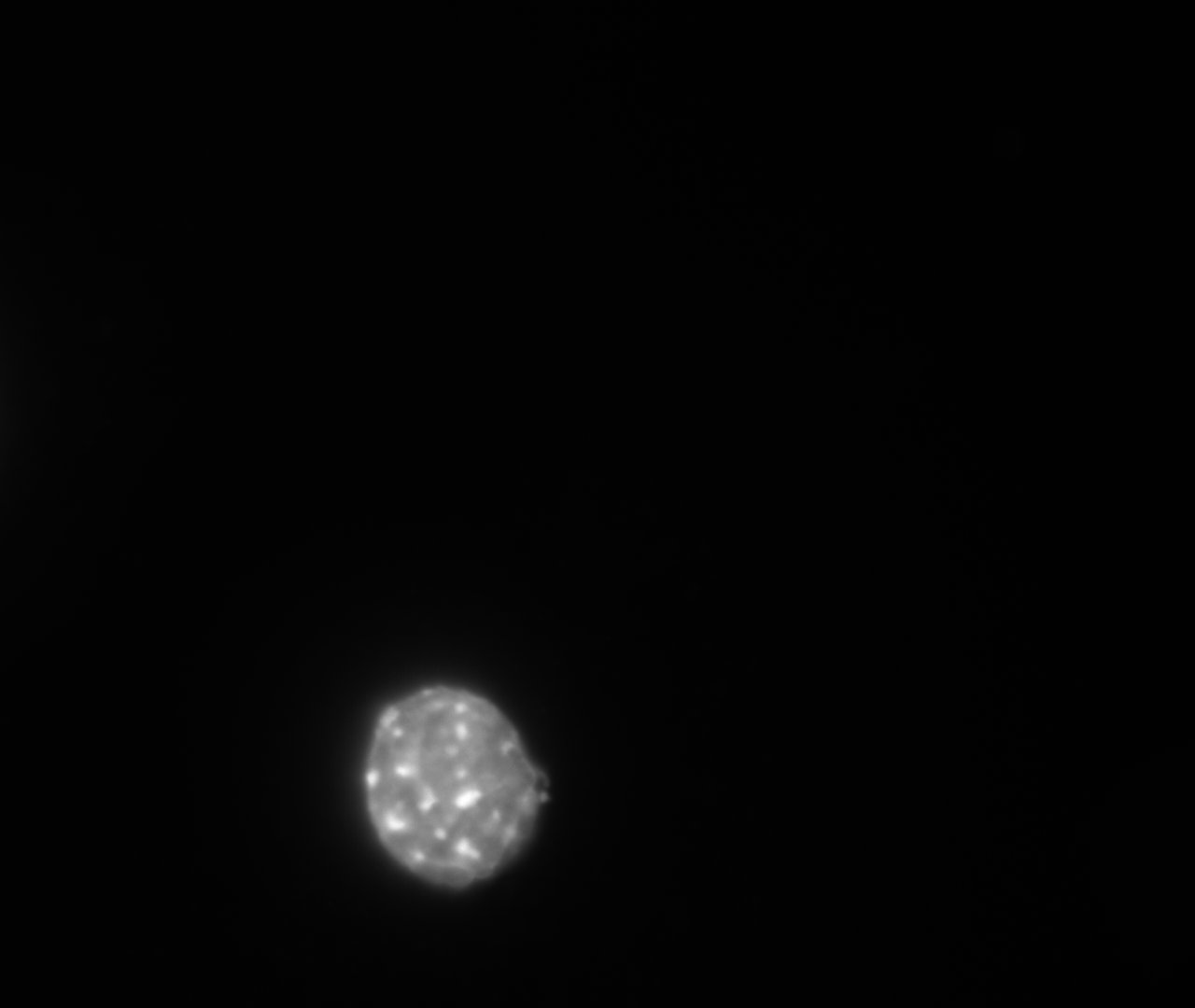

Supplement: Supplementary file 13 — Figures EV and Appendix Source Data [file 44318_2024_348_MOESM13_ESM.zip › SD figure EV and Appendix/Appendix Figure 2G/360-6.jpg]

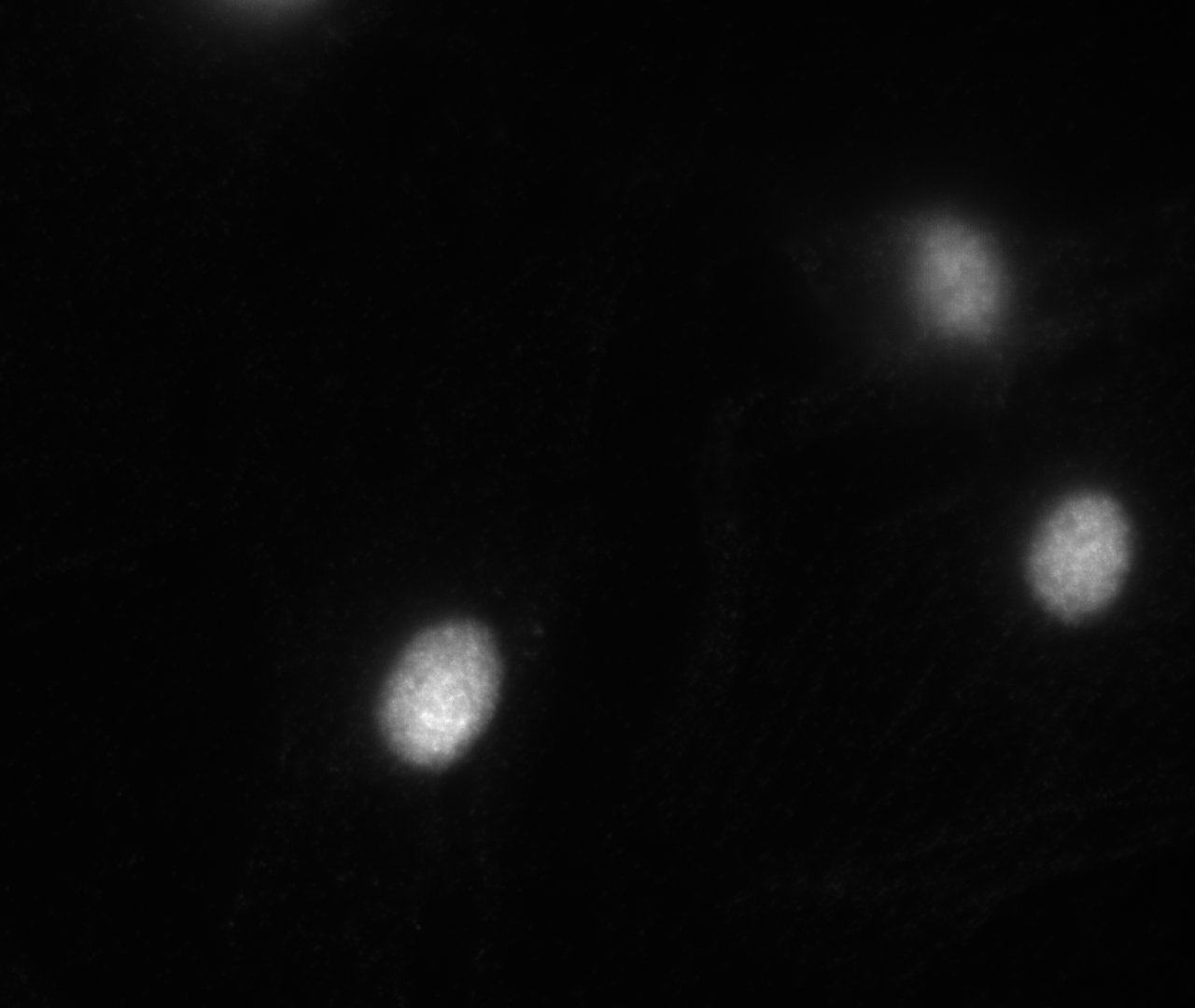

Supplement: Supplementary file 13 — Figures EV and Appendix Source Data [file 44318_2024_348_MOESM13_ESM.zip › SD figure EV and Appendix/Appendix Figure 2G/480.jpg]

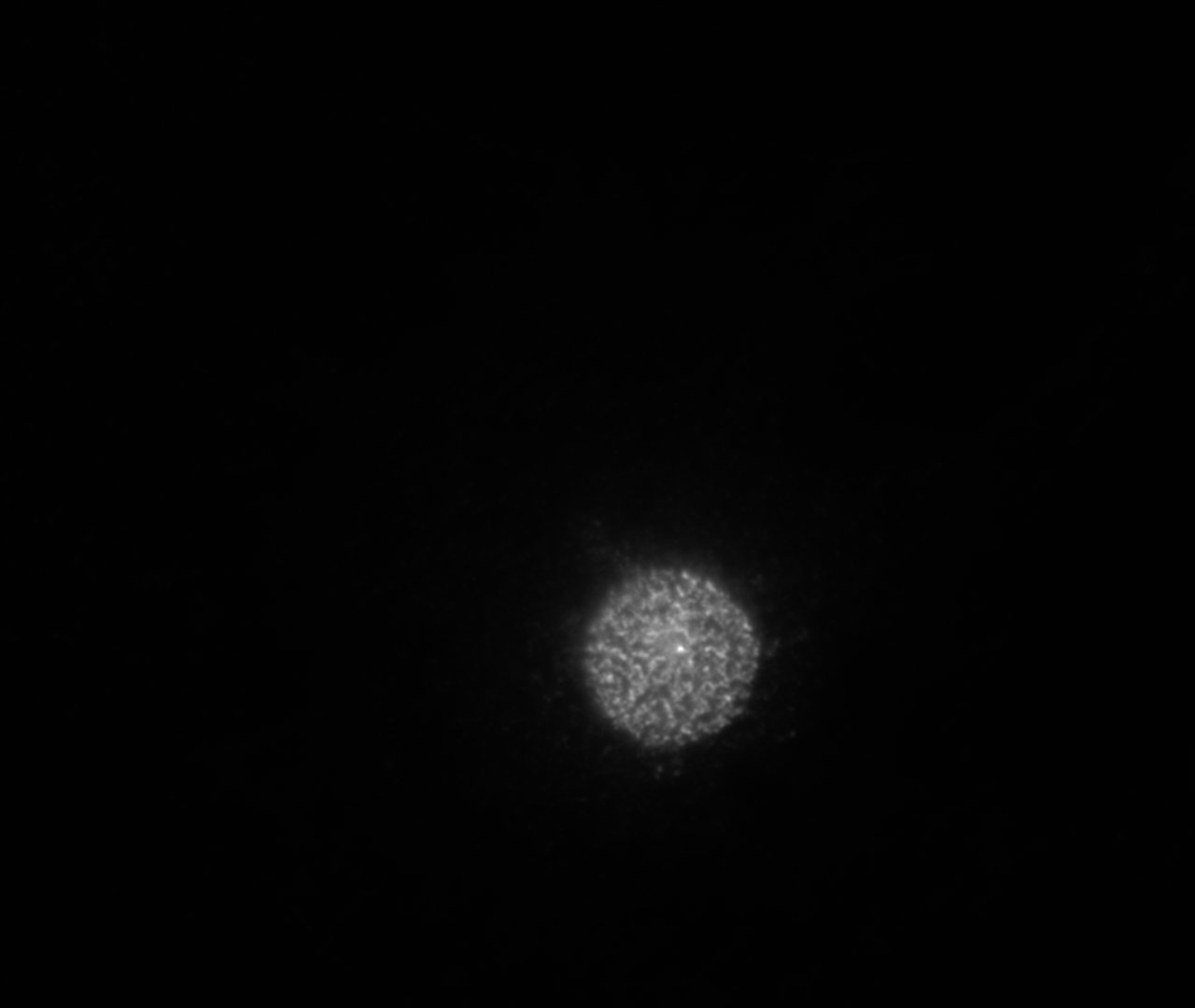

Supplement: Supplementary file 13 — Figures EV and Appendix Source Data [file 44318_2024_348_MOESM13_ESM.zip › SD figure EV and Appendix/Appendix Figure 2G/480-2.jpg]

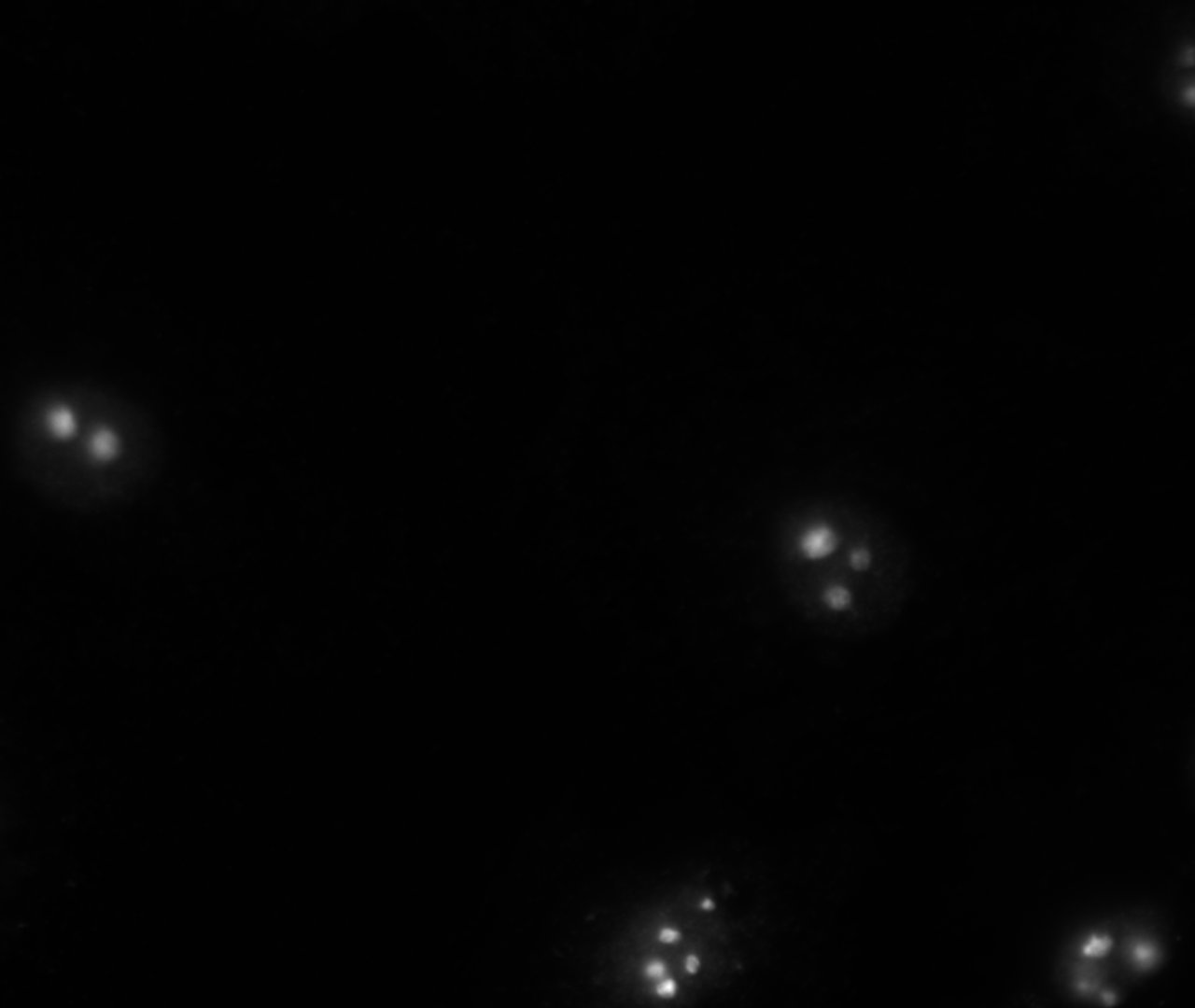

Supplement: Supplementary file 13 — Figures EV and Appendix Source Data [file 44318_2024_348_MOESM13_ESM.zip › SD figure EV and Appendix/Appendix Figure 2G/480-3.jpg]

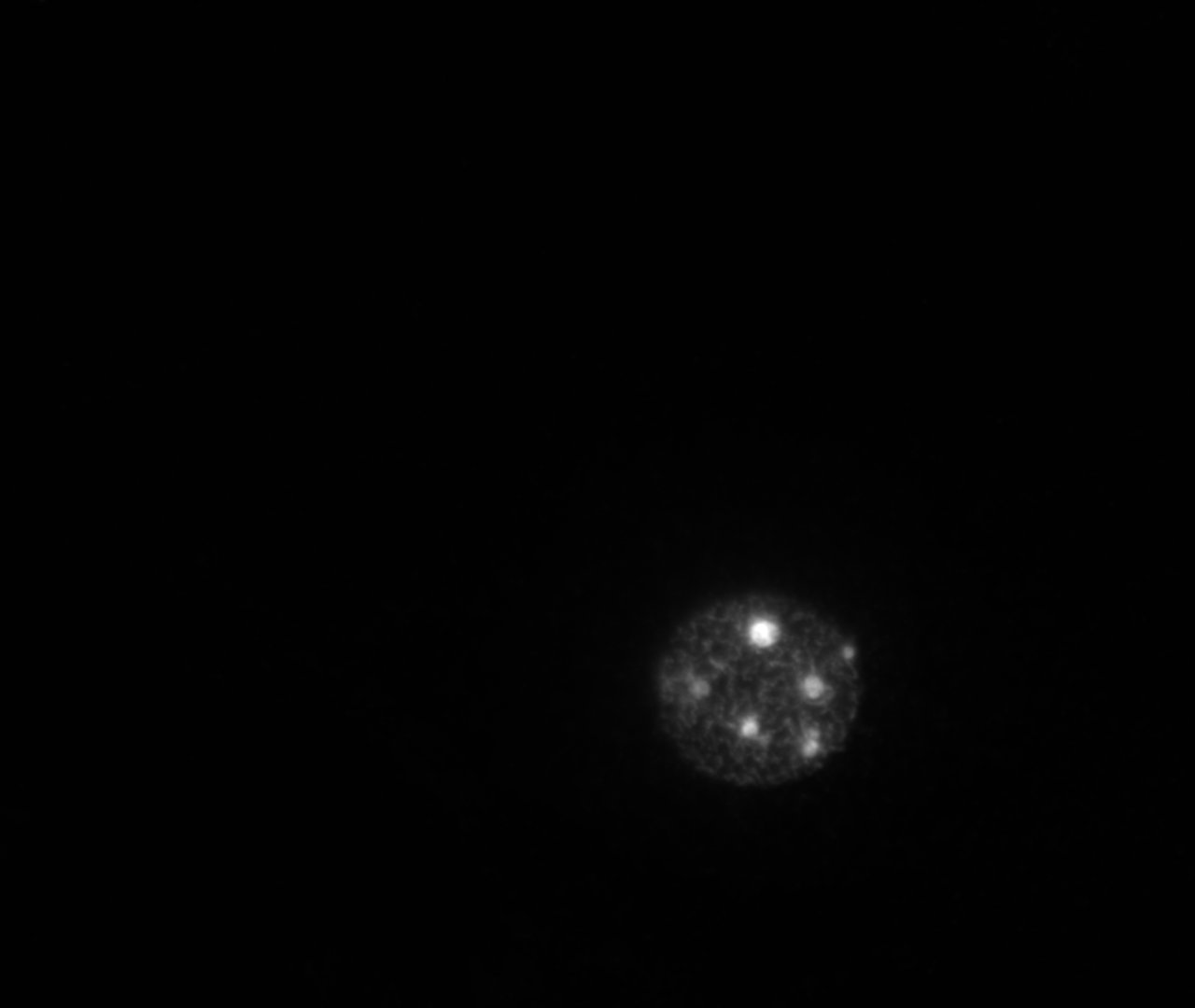

Supplement: Supplementary file 13 — Figures EV and Appendix Source Data [file 44318_2024_348_MOESM13_ESM.zip › SD figure EV and Appendix/Appendix Figure 2G/480-4.jpg]

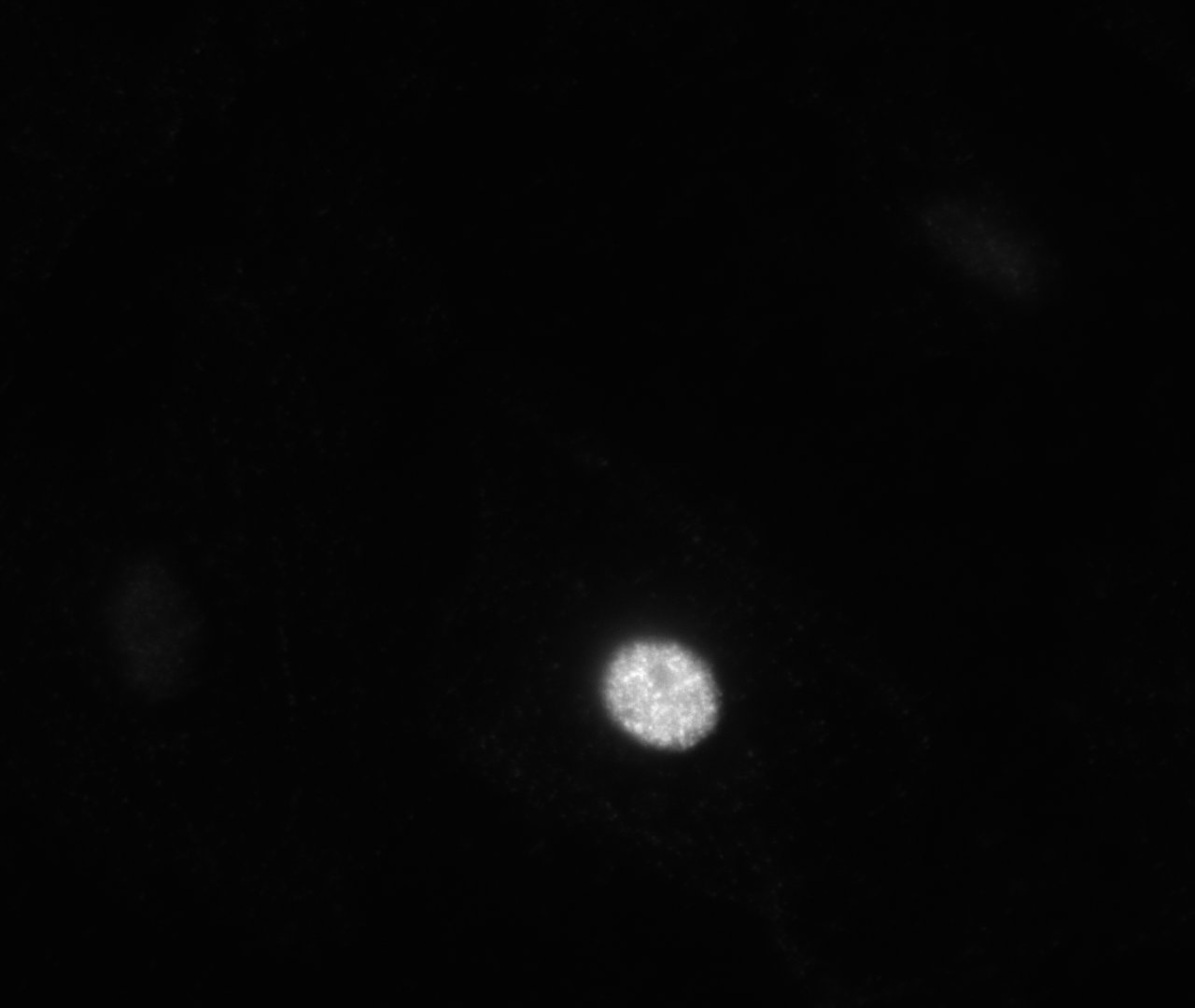

Supplement: Supplementary file 13 — Figures EV and Appendix Source Data [file 44318_2024_348_MOESM13_ESM.zip › SD figure EV and Appendix/Appendix Figure 2G/480-5.jpg]

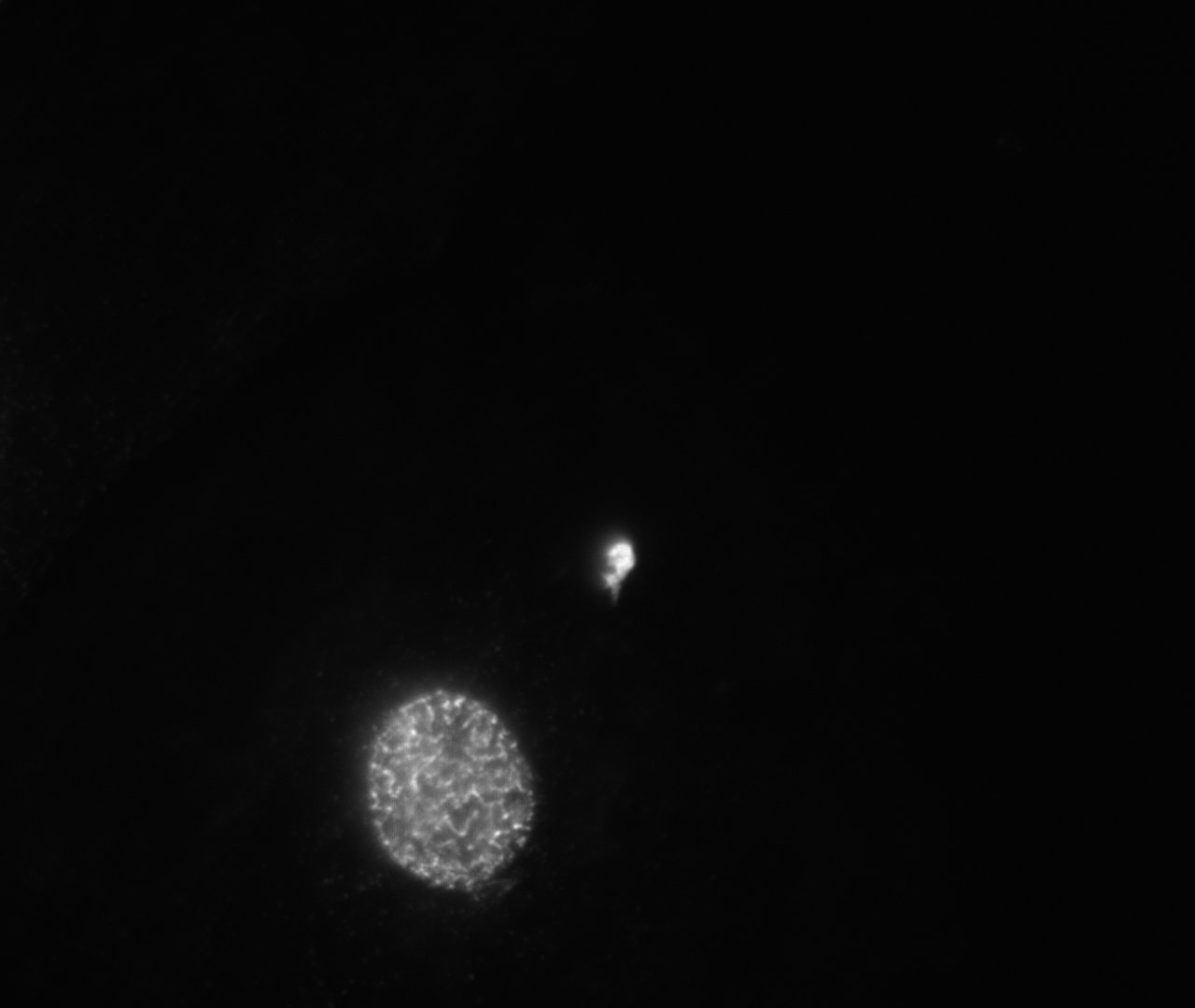

Supplement: Supplementary file 13 — Figures EV and Appendix Source Data [file 44318_2024_348_MOESM13_ESM.zip › SD figure EV and Appendix/Appendix Figure 2G/480-6.jpg]

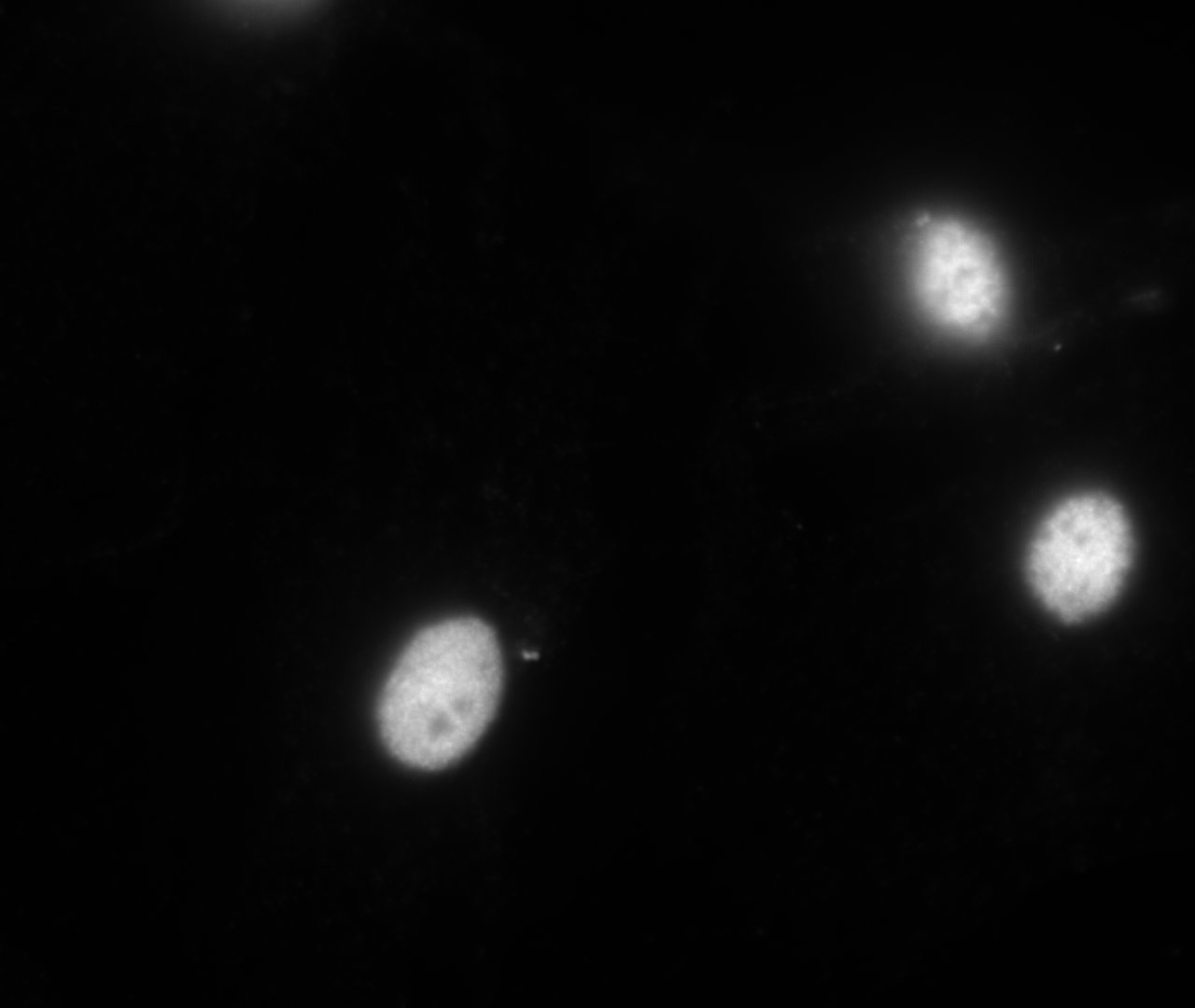

Supplement: Supplementary file 13 — Figures EV and Appendix Source Data [file 44318_2024_348_MOESM13_ESM.zip › SD figure EV and Appendix/Appendix Figure 2G/560.jpg]

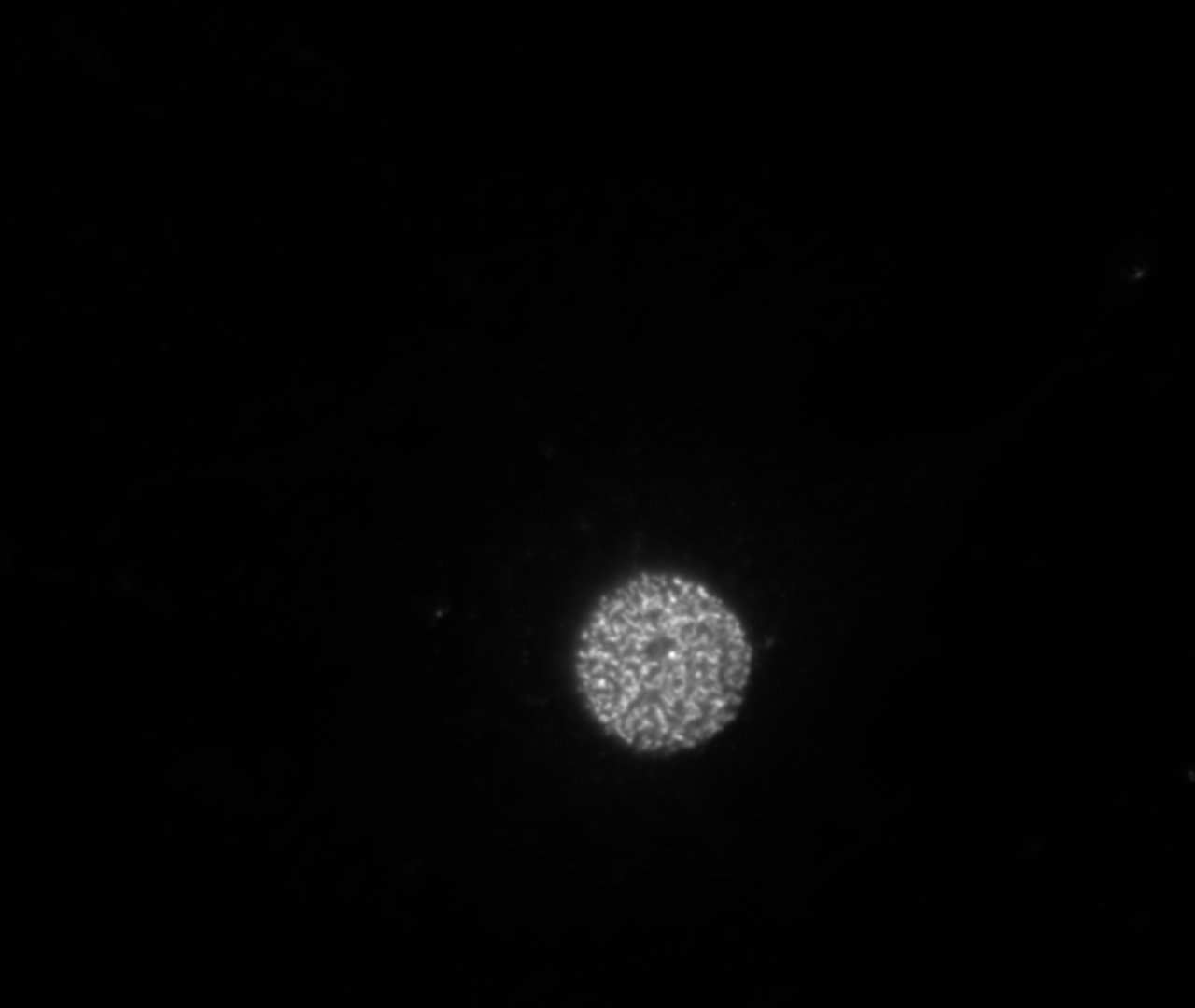

Supplement: Supplementary file 13 — Figures EV and Appendix Source Data [file 44318_2024_348_MOESM13_ESM.zip › SD figure EV and Appendix/Appendix Figure 2G/560-2.jpg]

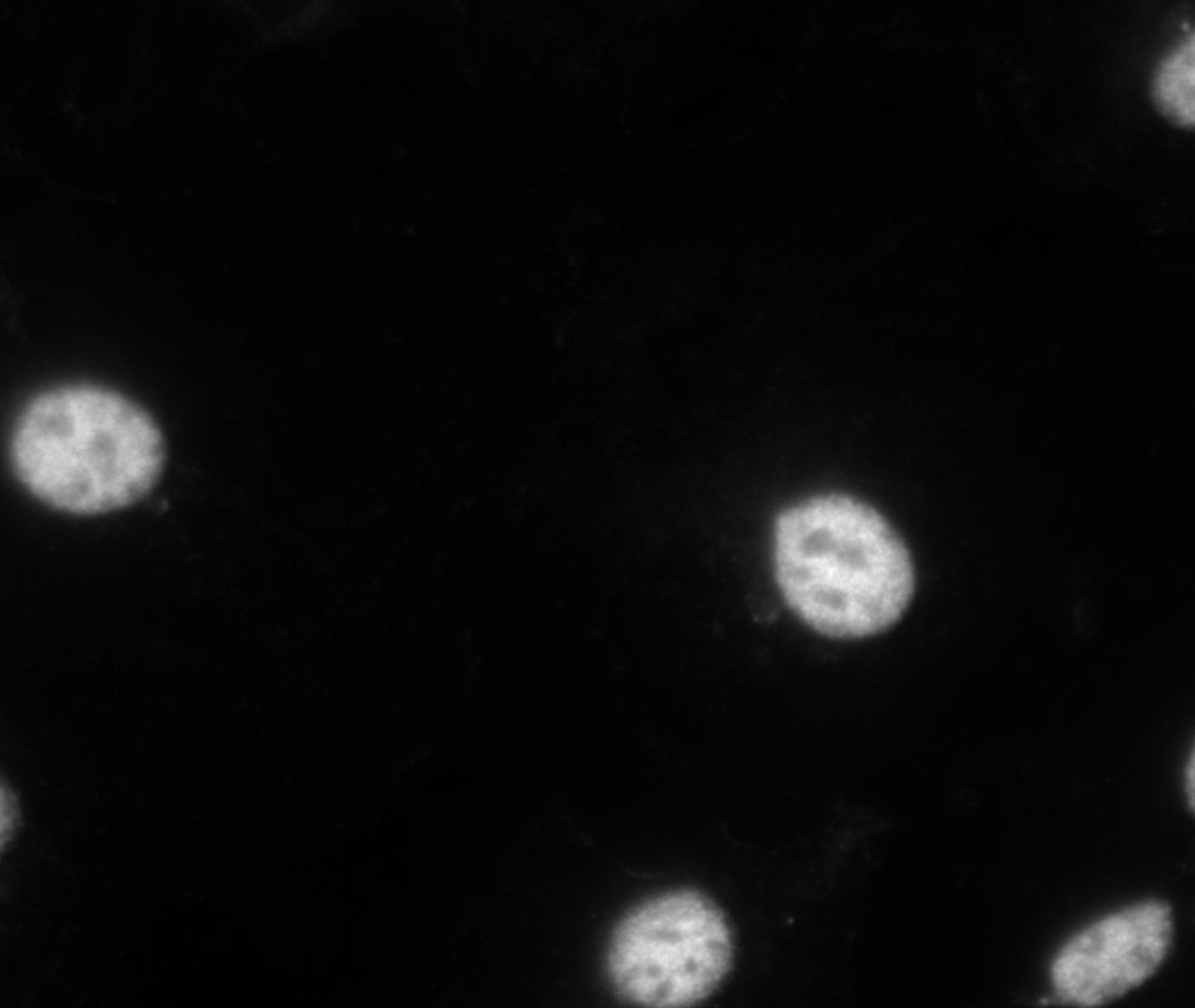

Supplement: Supplementary file 13 — Figures EV and Appendix Source Data [file 44318_2024_348_MOESM13_ESM.zip › SD figure EV and Appendix/Appendix Figure 2G/560-3.jpg]

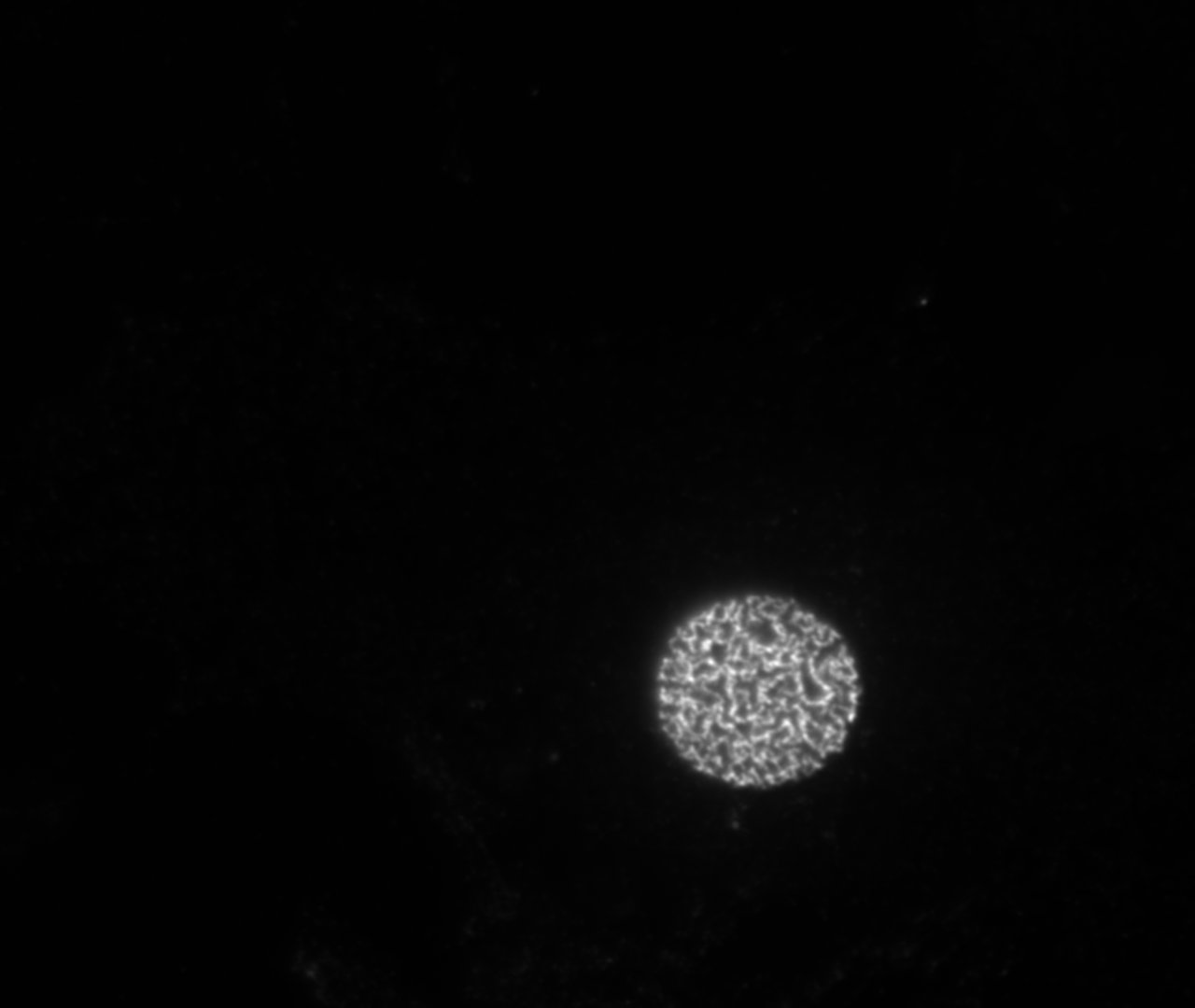

Supplement: Supplementary file 13 — Figures EV and Appendix Source Data [file 44318_2024_348_MOESM13_ESM.zip › SD figure EV and Appendix/Appendix Figure 2G/560-4.jpg]

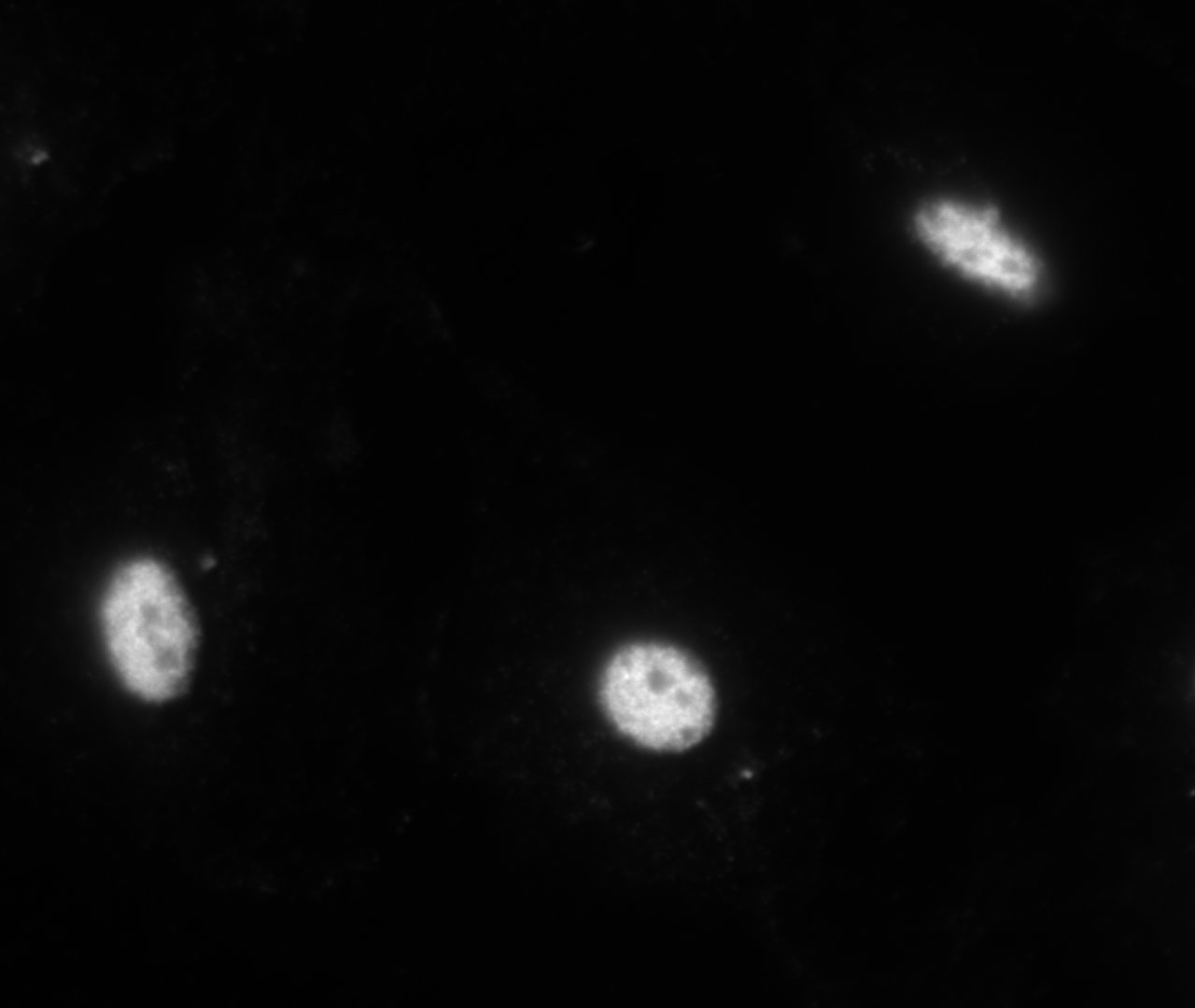

Supplement: Supplementary file 13 — Figures EV and Appendix Source Data [file 44318_2024_348_MOESM13_ESM.zip › SD figure EV and Appendix/Appendix Figure 2G/560-5.jpg]
