# Supplementary material for: Cohesin positions the epigenetic reader Phf2 within the genome
Source: EMBO J. 2025 Jan 2;44(3):736–66. doi: 10.1038/s44318-024-00348-2 (PMC11790891; doi:10.1038/s44318-024-00348-2)
Supplement: Supplementary file 6 — Source data Fig. 2 [file 44318_2024_348_MOESM6_ESM.zip › Figure 2/2A/GEO.rtf]

To review GEO accession GSE278142 for ChIP-Seq:
Go to https://www.ncbi.nlm.nih.gov/geo/query/acc.cgi?acc=GSE278142
Enter token ktcdukccrxcbrgl into the box
