# Supplementary material for: Cohesin positions the epigenetic reader Phf2 within the genome
Source: EMBO J. 2025 Jan 2;44(3):736–66. doi: 10.1038/s44318-024-00348-2 (PMC11790891; doi:10.1038/s44318-024-00348-2)
Supplement: Supplementary file 10 — Source data Fig. 6 [file 44318_2024_348_MOESM10_ESM.zip › Figure 6/6B/GEO.rtf]

To review GEO accession GSE278143 for Hi-C:
Go to https://www.ncbi.nlm.nih.gov/geo/query/acc.cgi?acc=GSE278143
Enter token stgxacaorburdsh into the box


	
