# Supplementary figures and images for: Cohesin positions the epigenetic reader Phf2 within the genome (part 3 of 3)
Source: EMBO J. 2025 Jan 2;44(3):736–66. doi: 10.1038/s44318-024-00348-2 (PMC11790891; doi:10.1038/s44318-024-00348-2)

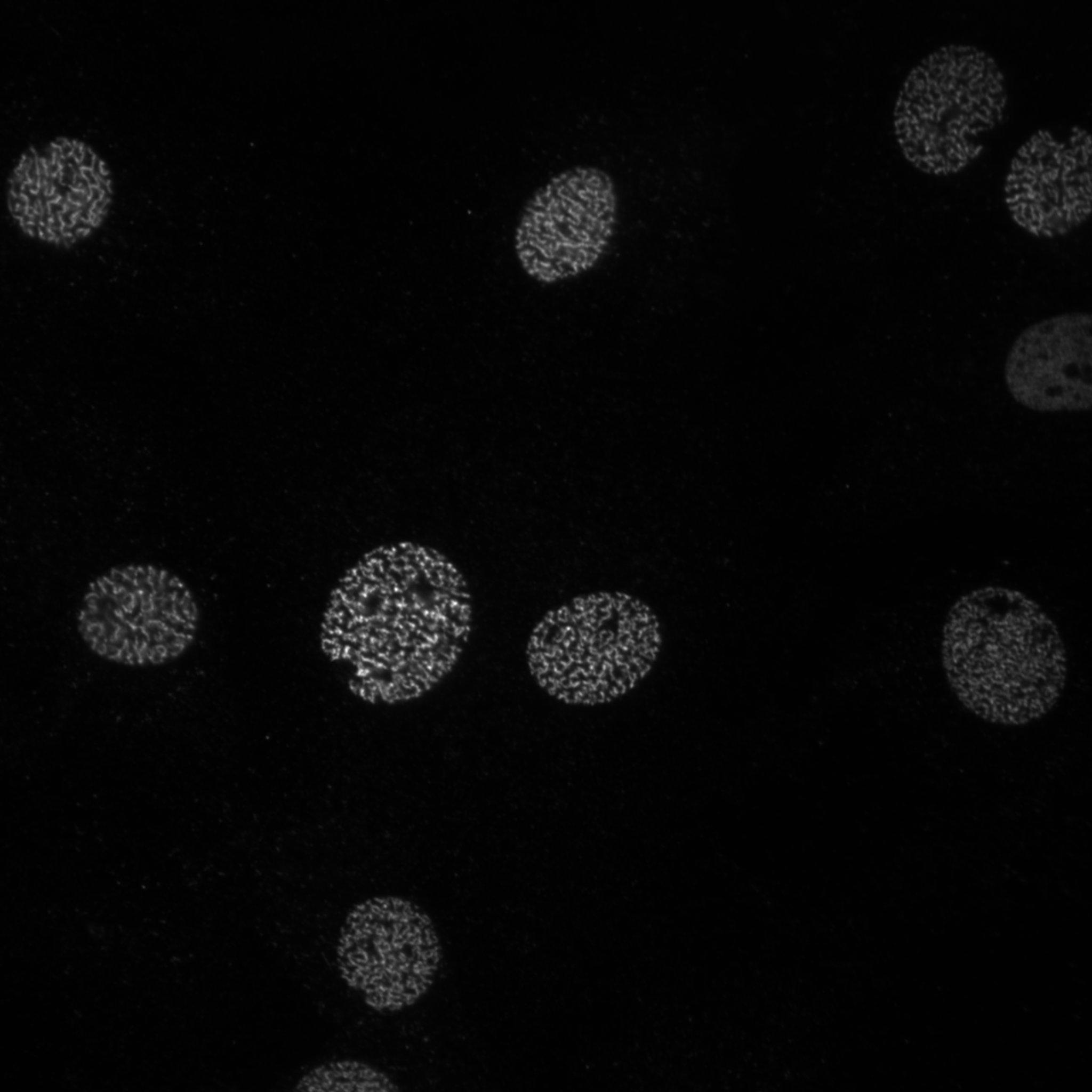

Supplement: Supplementary file 13 — Figures EV and Appendix Source Data [file 44318_2024_348_MOESM13_ESM.zip › SD figure EV and Appendix/Appendix Figure 1F/Brwd1/560-2.jpg]

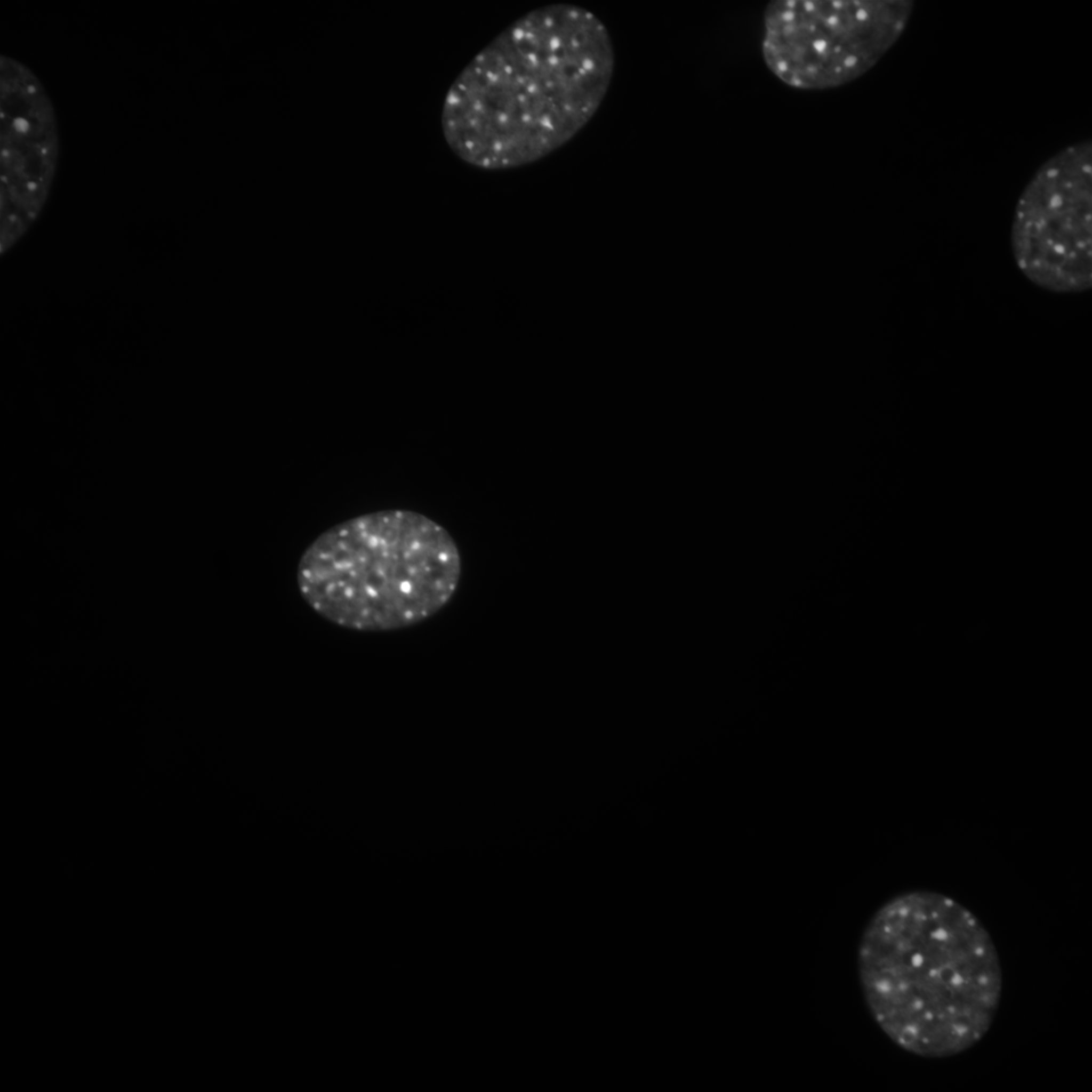

Supplement: Supplementary file 13 — Figures EV and Appendix Source Data [file 44318_2024_348_MOESM13_ESM.zip › SD figure EV and Appendix/Appendix Figure 1F/Brwd1/360.jpg]

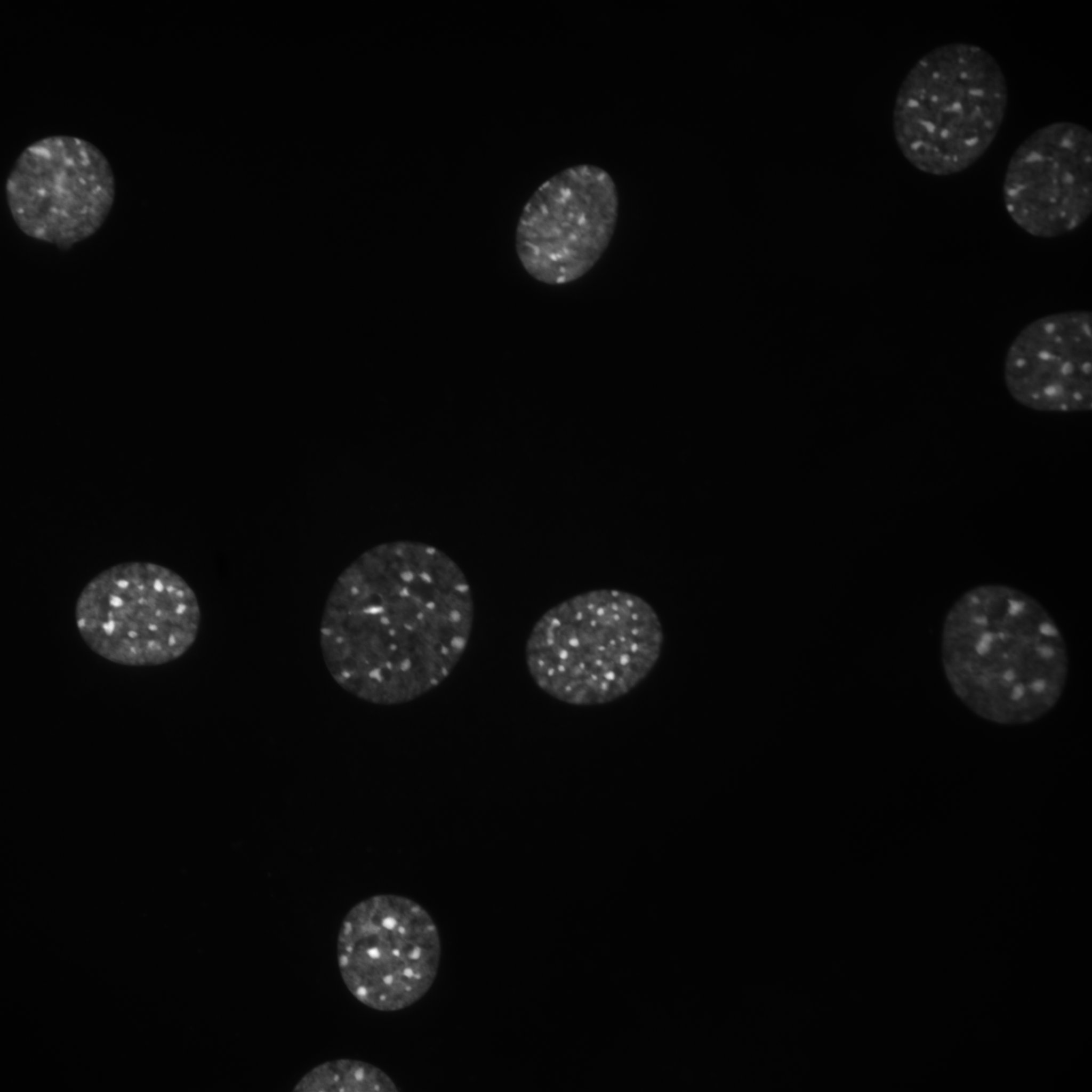

Supplement: Supplementary file 13 — Figures EV and Appendix Source Data [file 44318_2024_348_MOESM13_ESM.zip › SD figure EV and Appendix/Appendix Figure 1F/Brwd1/360-2.jpg]

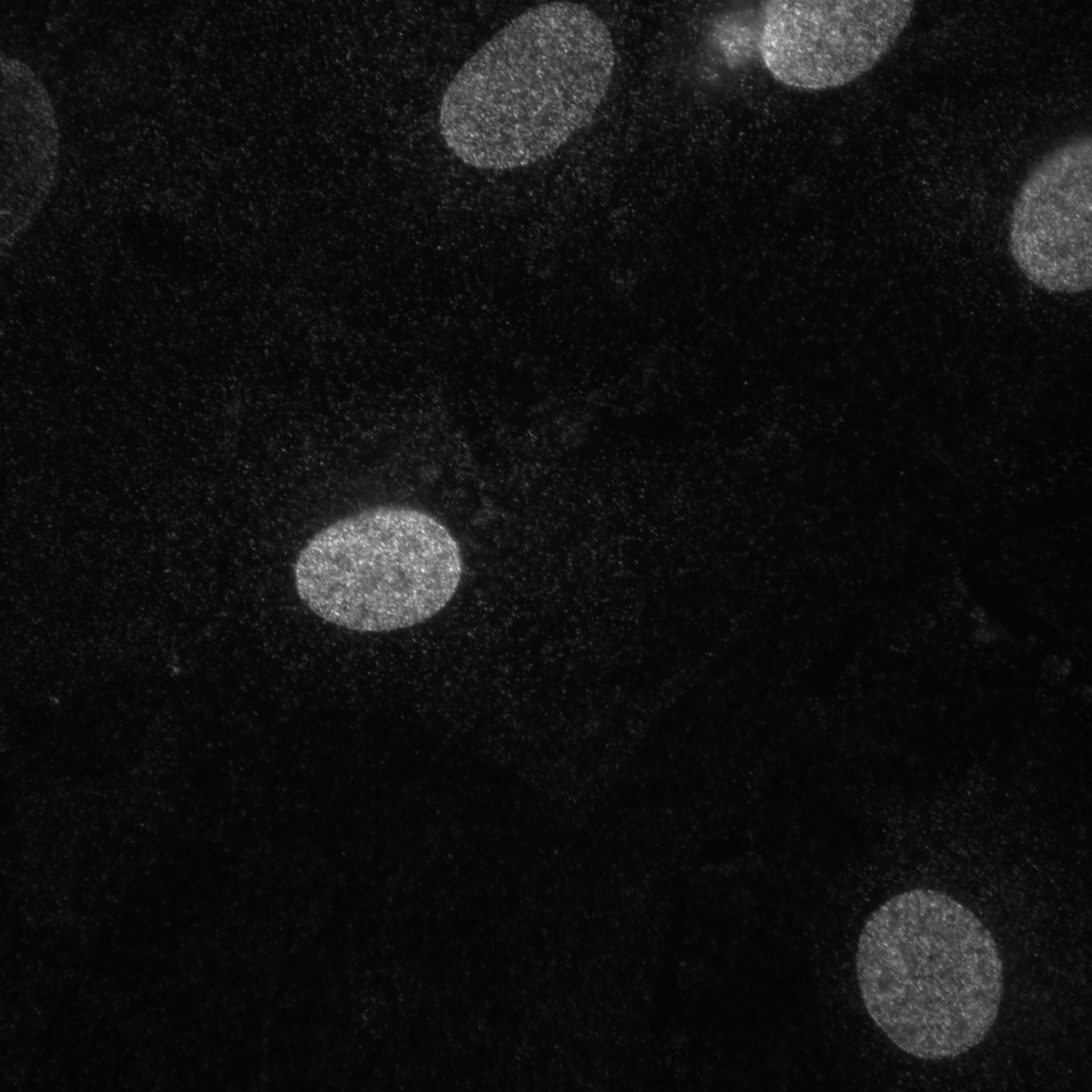

Supplement: Supplementary file 13 — Figures EV and Appendix Source Data [file 44318_2024_348_MOESM13_ESM.zip › SD figure EV and Appendix/Appendix Figure 1F/Brwd1/480.jpg]

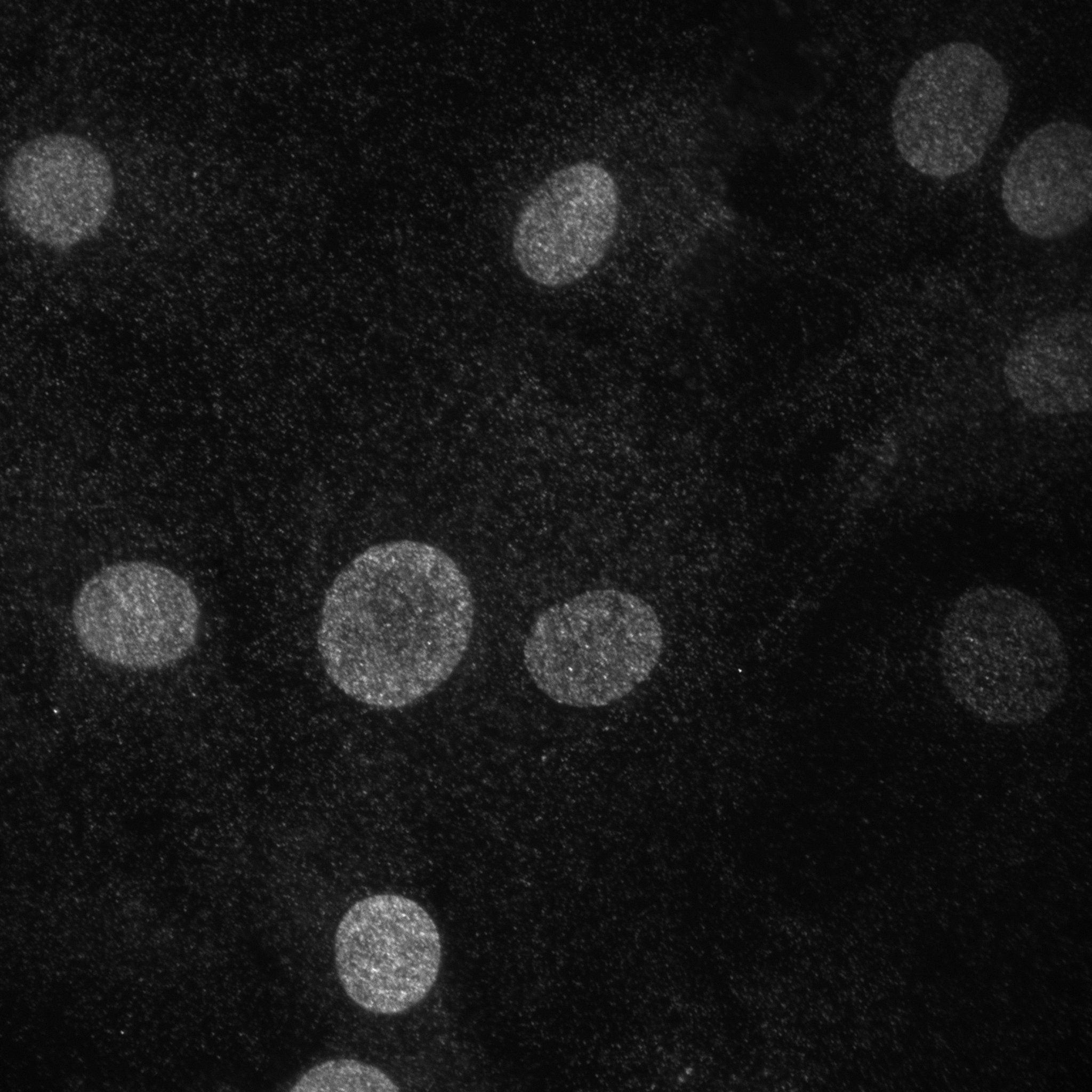

Supplement: Supplementary file 13 — Figures EV and Appendix Source Data [file 44318_2024_348_MOESM13_ESM.zip › SD figure EV and Appendix/Appendix Figure 1F/Brwd1/480-2.jpg]

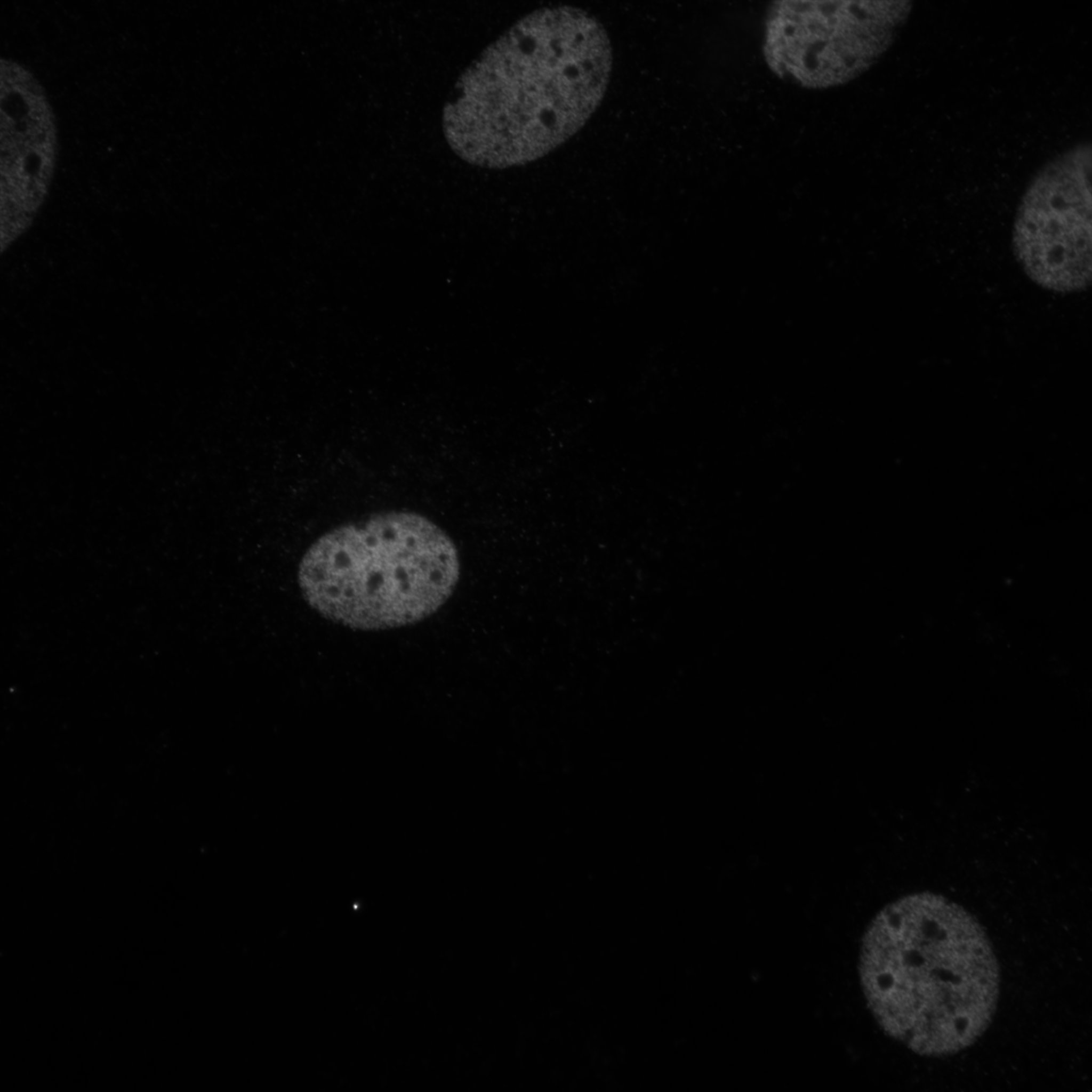

Supplement: Supplementary file 13 — Figures EV and Appendix Source Data [file 44318_2024_348_MOESM13_ESM.zip › SD figure EV and Appendix/Appendix Figure 1F/Brwd1/560.jpg]
