# Supplementary figures and images for: Cohesin positions the epigenetic reader Phf2 within the genome (part 2 of 3)
Source: EMBO J. 2025 Jan 2;44(3):736–66. doi: 10.1038/s44318-024-00348-2 (PMC11790891; doi:10.1038/s44318-024-00348-2)

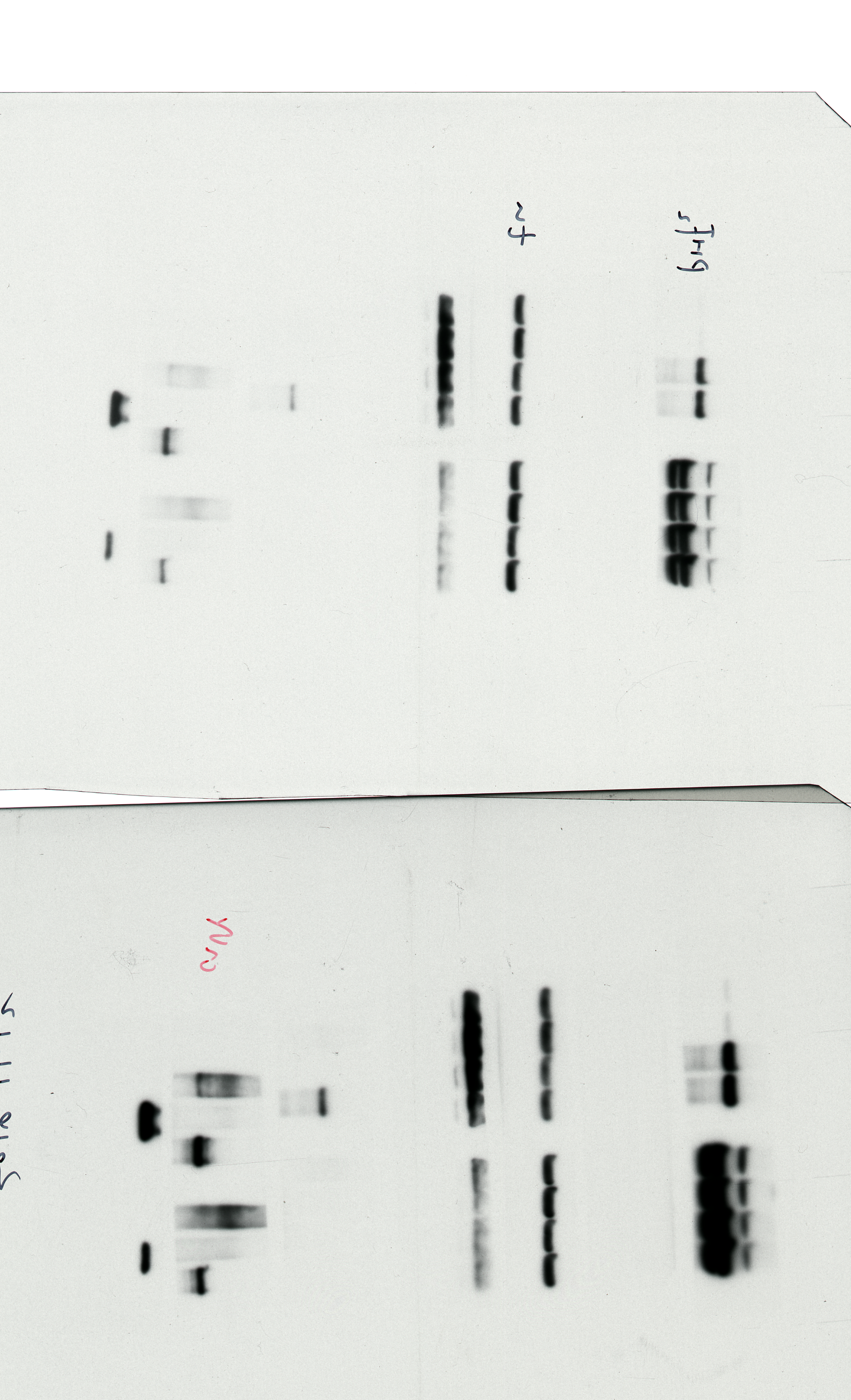

Supplement: Supplementary file 13 — Figures EV and Appendix Source Data [file 44318_2024_348_MOESM13_ESM.zip › SD figure EV and Appendix/Appendix Figure 1B/Untitled-1.tif]

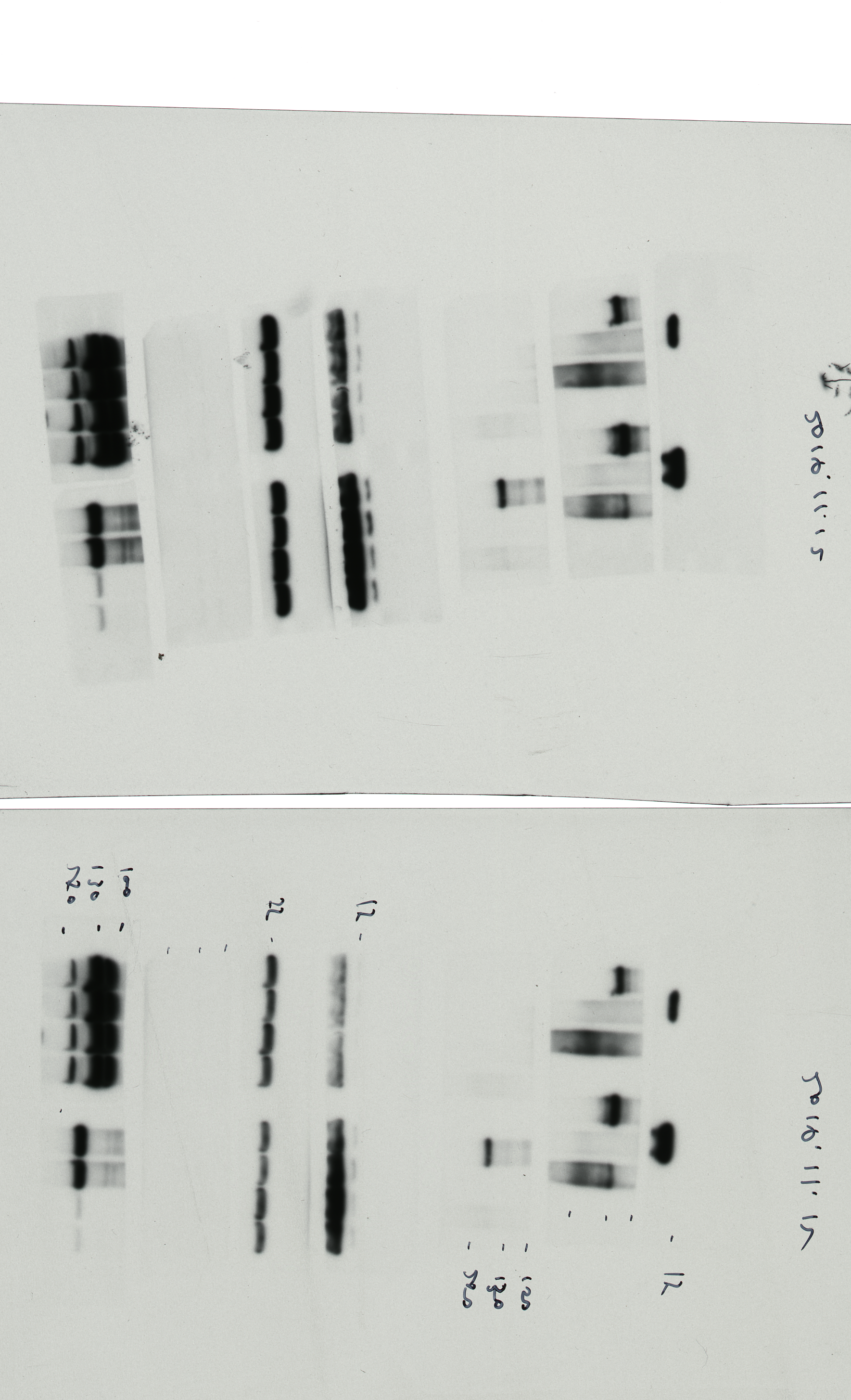

Supplement: Supplementary file 13 — Figures EV and Appendix Source Data [file 44318_2024_348_MOESM13_ESM.zip › SD figure EV and Appendix/Appendix Figure 1B/Untitled-2.tif]

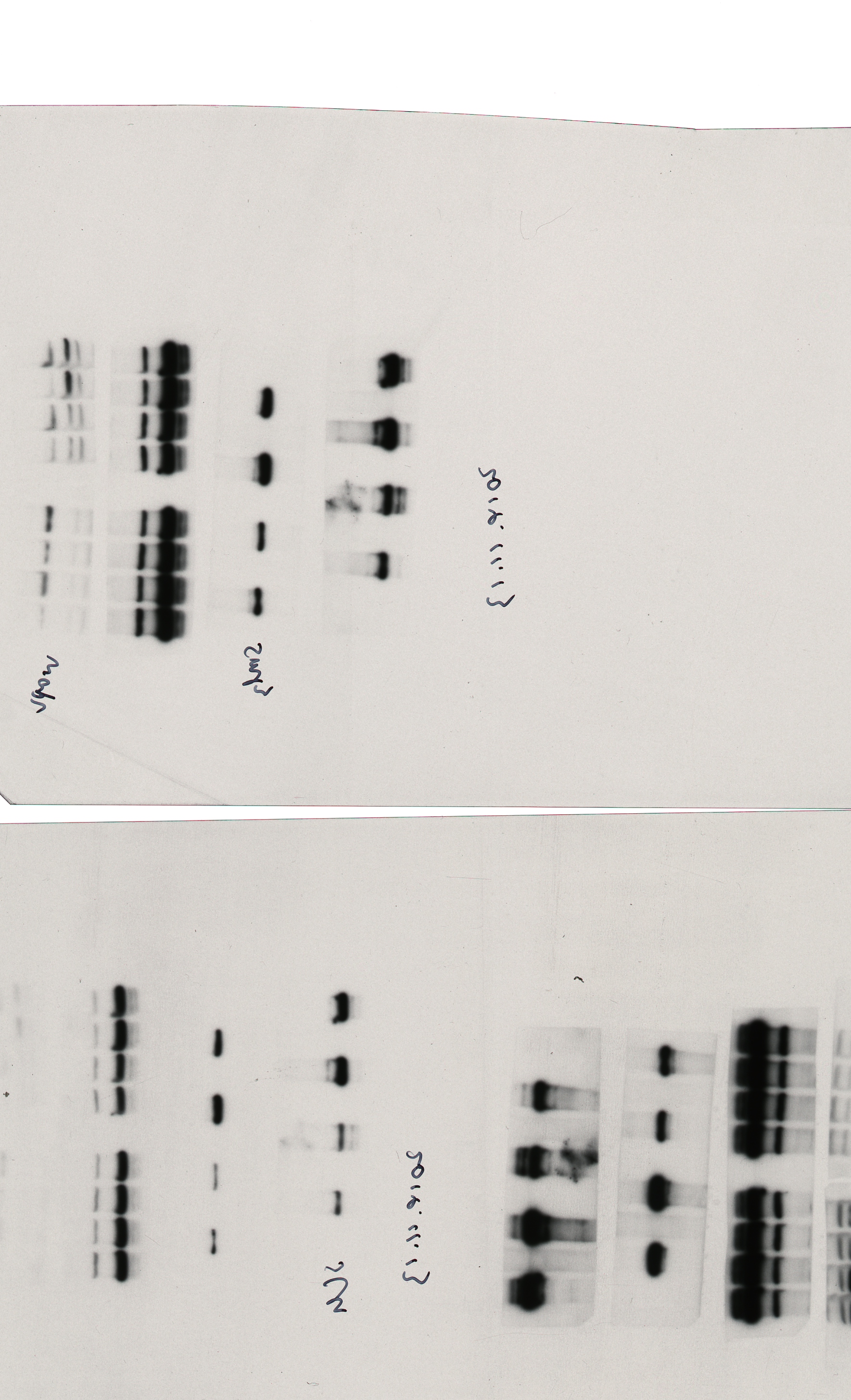

Supplement: Supplementary file 13 — Figures EV and Appendix Source Data [file 44318_2024_348_MOESM13_ESM.zip › SD figure EV and Appendix/Appendix Figure 1B/Untitled-3.tif]

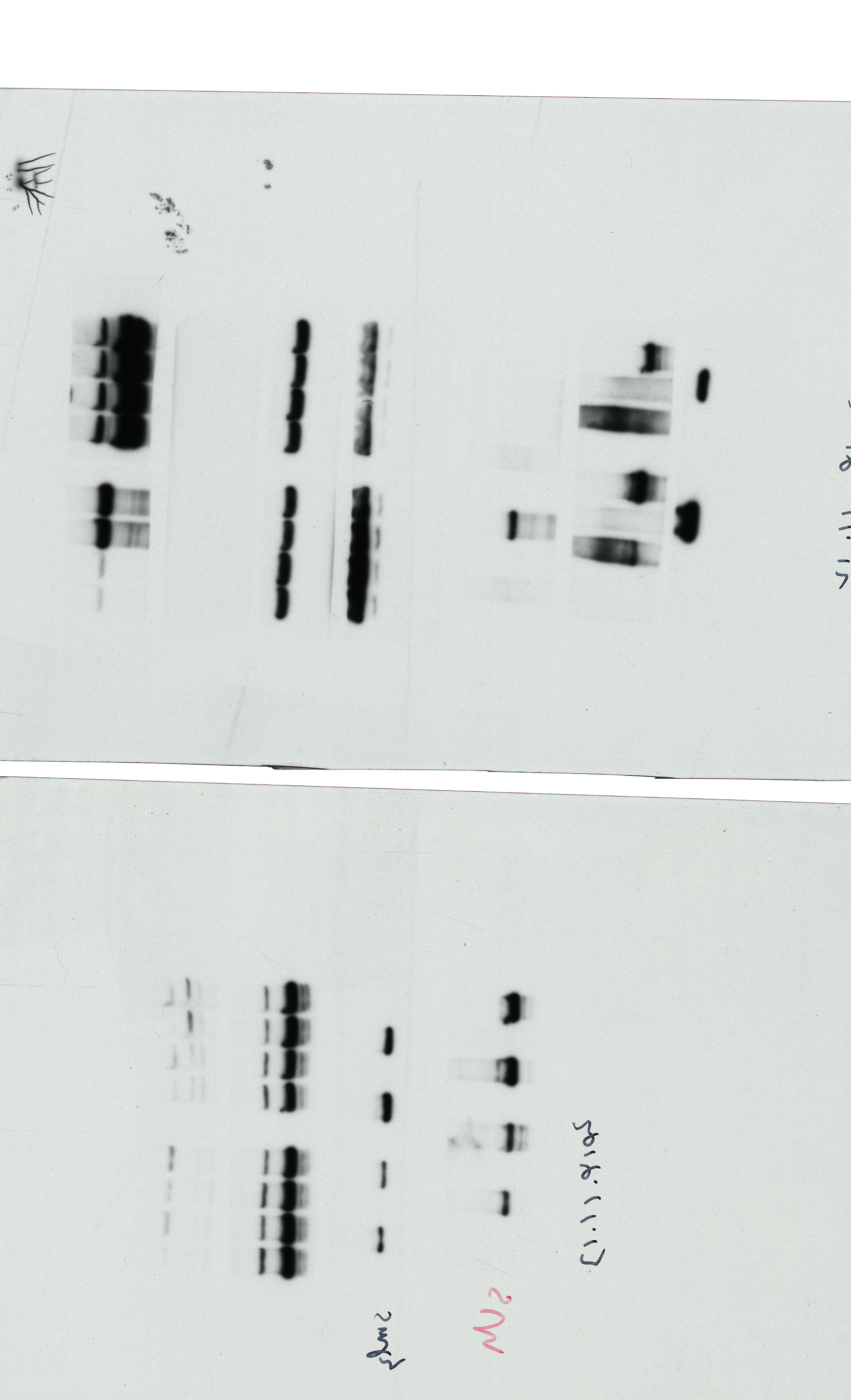

Supplement: Supplementary file 13 — Figures EV and Appendix Source Data [file 44318_2024_348_MOESM13_ESM.zip › SD figure EV and Appendix/Appendix Figure 1B/Untitled-5.tif]

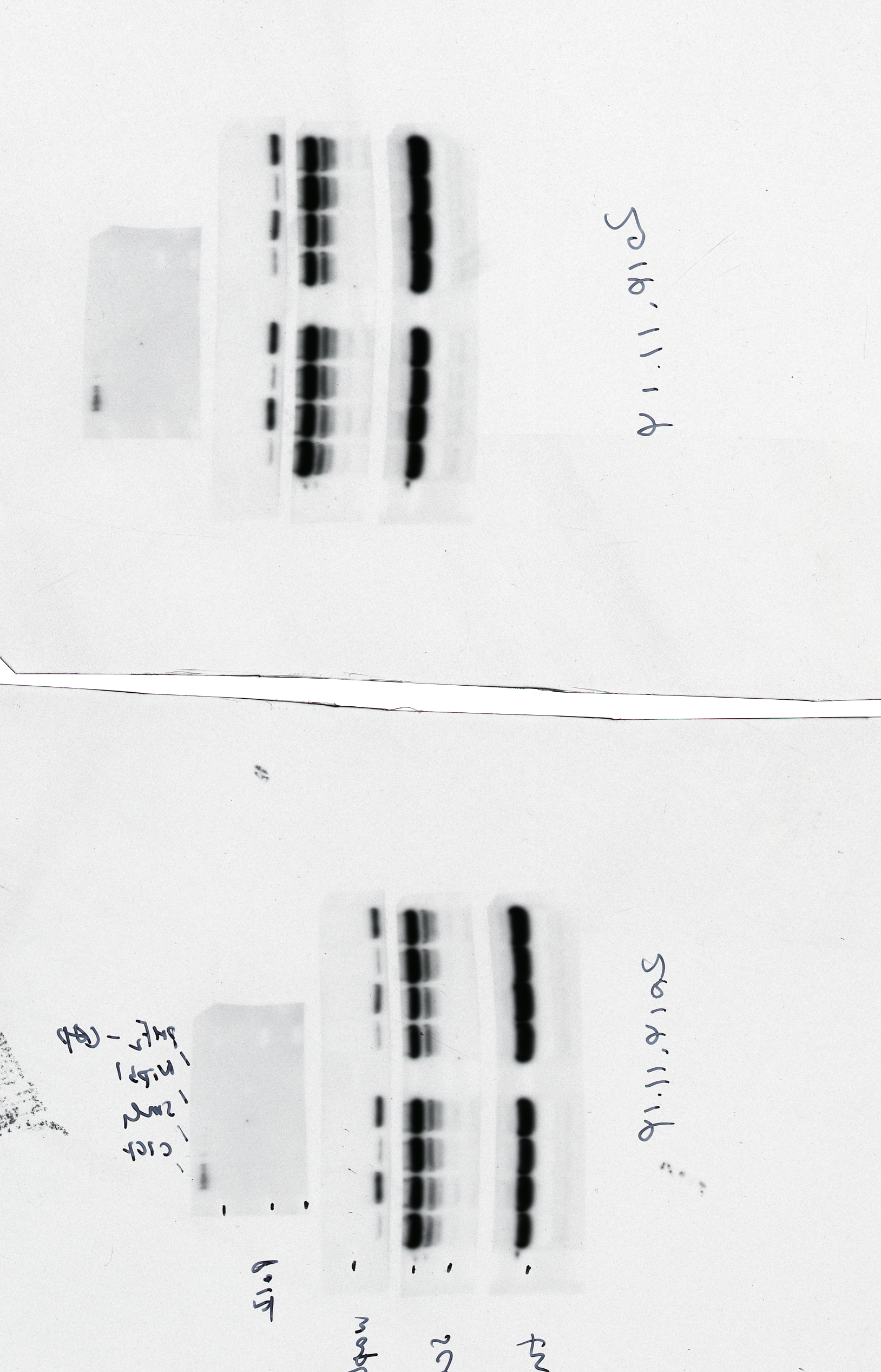

Supplement: Supplementary file 13 — Figures EV and Appendix Source Data [file 44318_2024_348_MOESM13_ESM.zip › SD figure EV and Appendix/Appendix Figure 4AB/Untitled-11.tif]

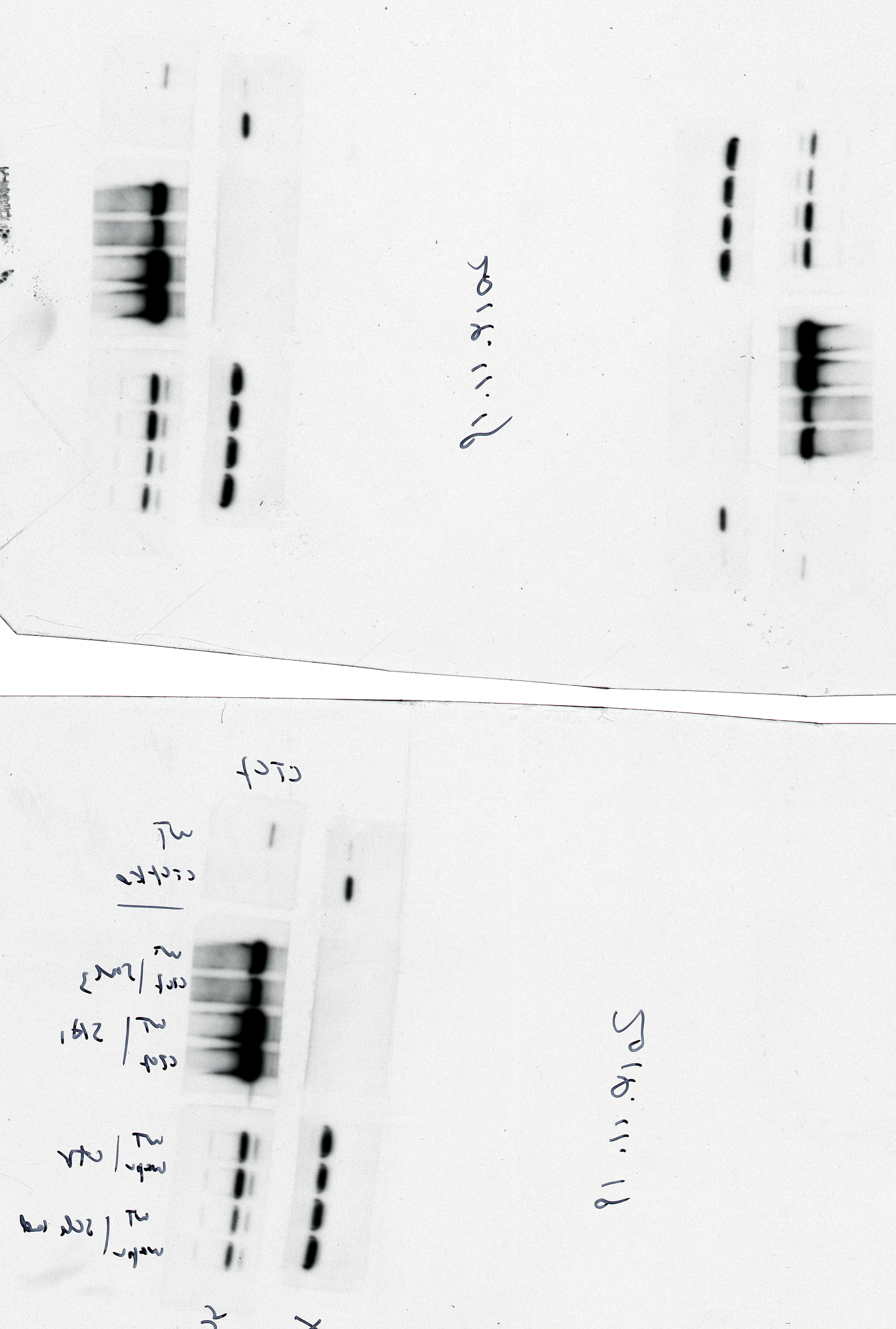

Supplement: Supplementary file 13 — Figures EV and Appendix Source Data [file 44318_2024_348_MOESM13_ESM.zip › SD figure EV and Appendix/Appendix Figure 4AB/Untitled-22.tif]

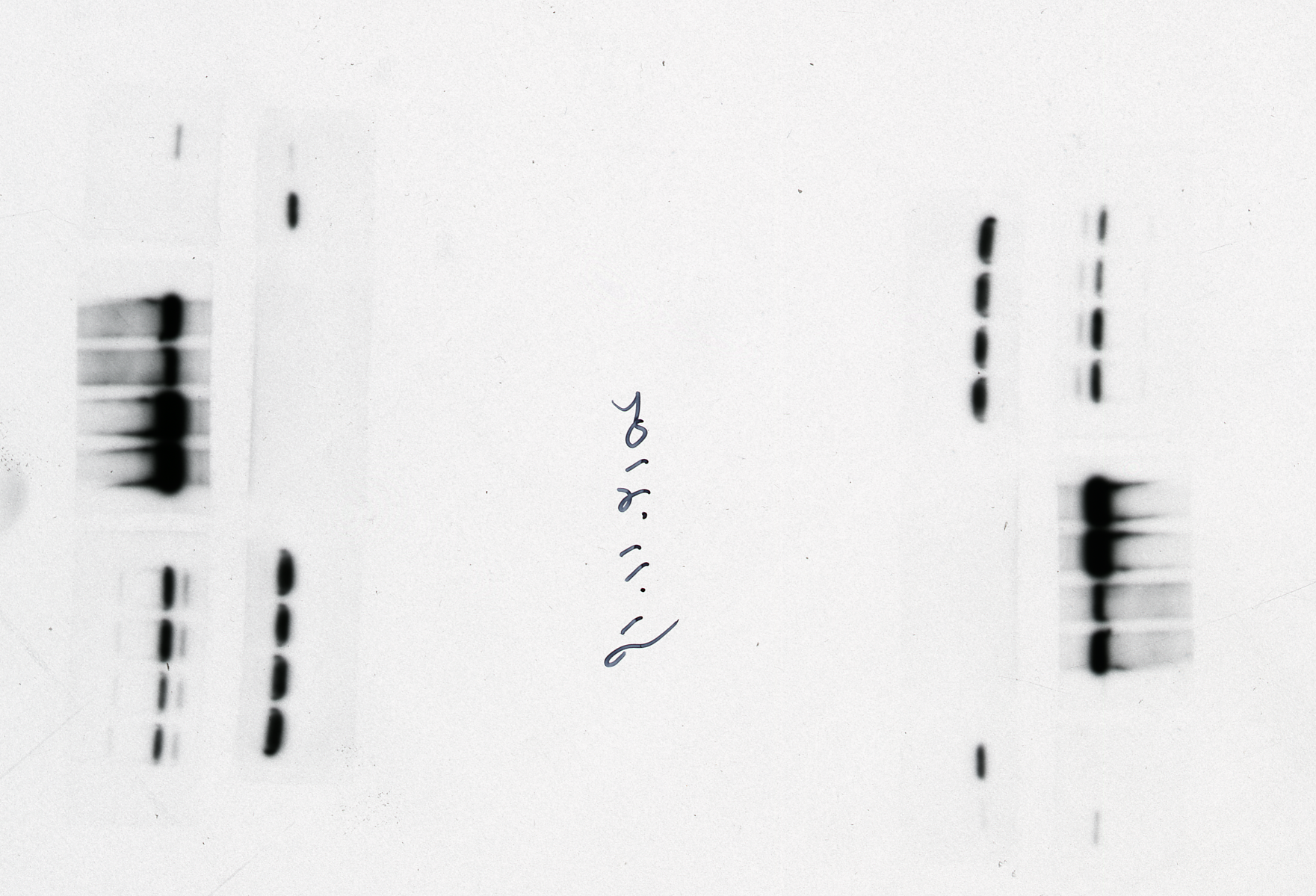

Supplement: Supplementary file 13 — Figures EV and Appendix Source Data [file 44318_2024_348_MOESM13_ESM.zip › SD figure EV and Appendix/Appendix Figure 4AB/Untitled-33.tif]

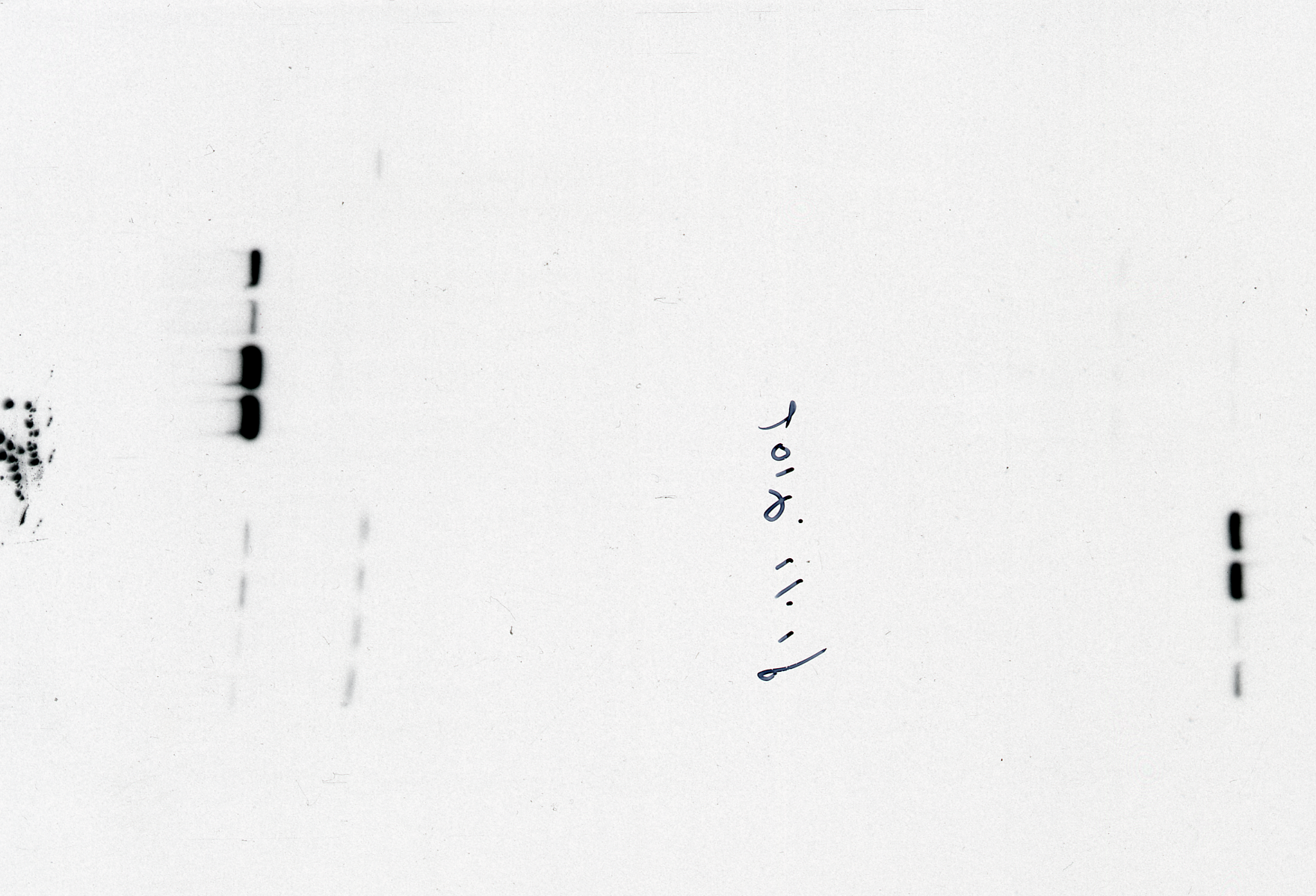

Supplement: Supplementary file 13 — Figures EV and Appendix Source Data [file 44318_2024_348_MOESM13_ESM.zip › SD figure EV and Appendix/Appendix Figure 4AB/Untitled-44.tif]

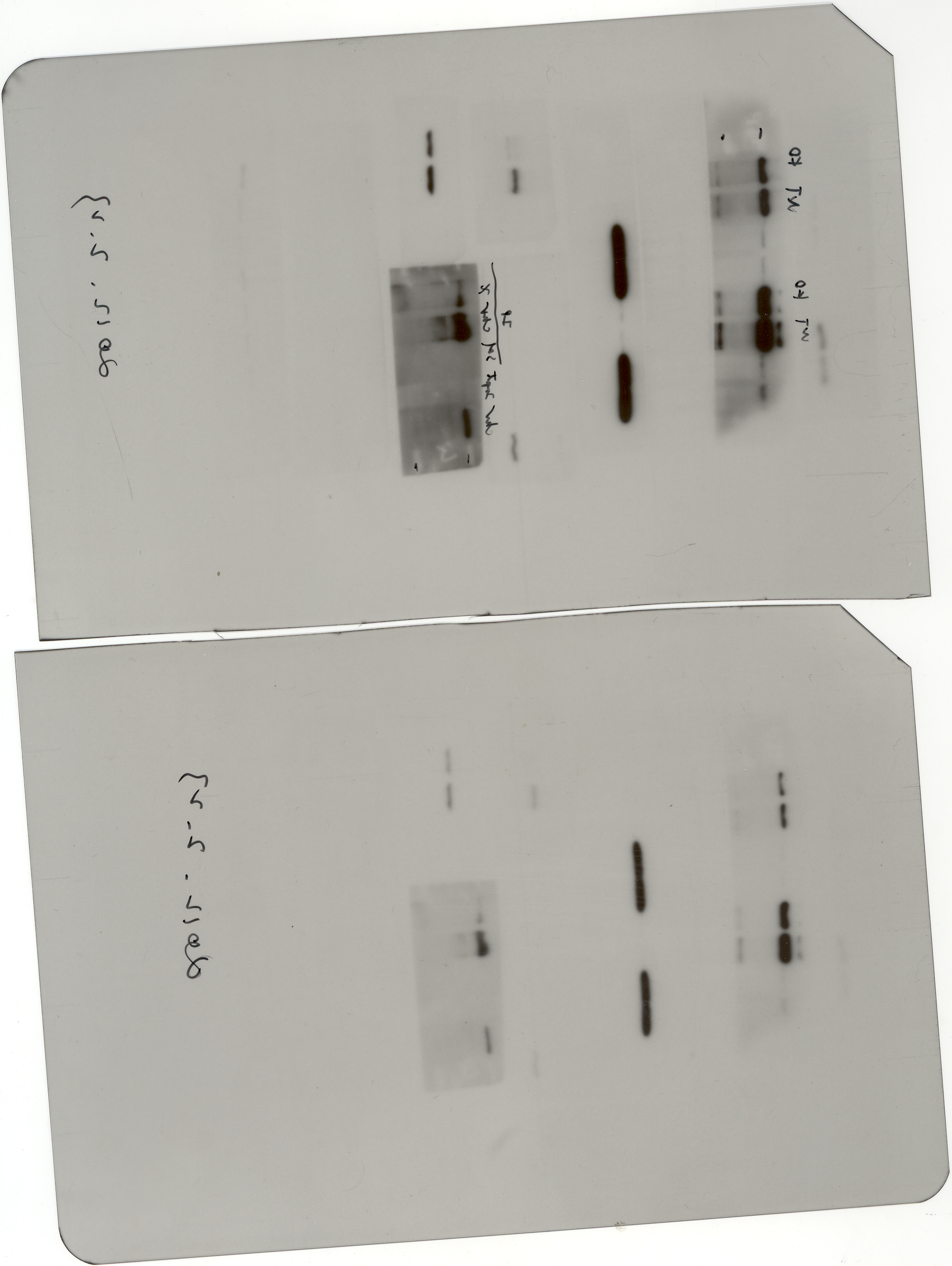

Supplement: Supplementary file 13 — Figures EV and Appendix Source Data [file 44318_2024_348_MOESM13_ESM.zip › SD figure EV and Appendix/Appendix Figure 2A/Untitled-1.tif]

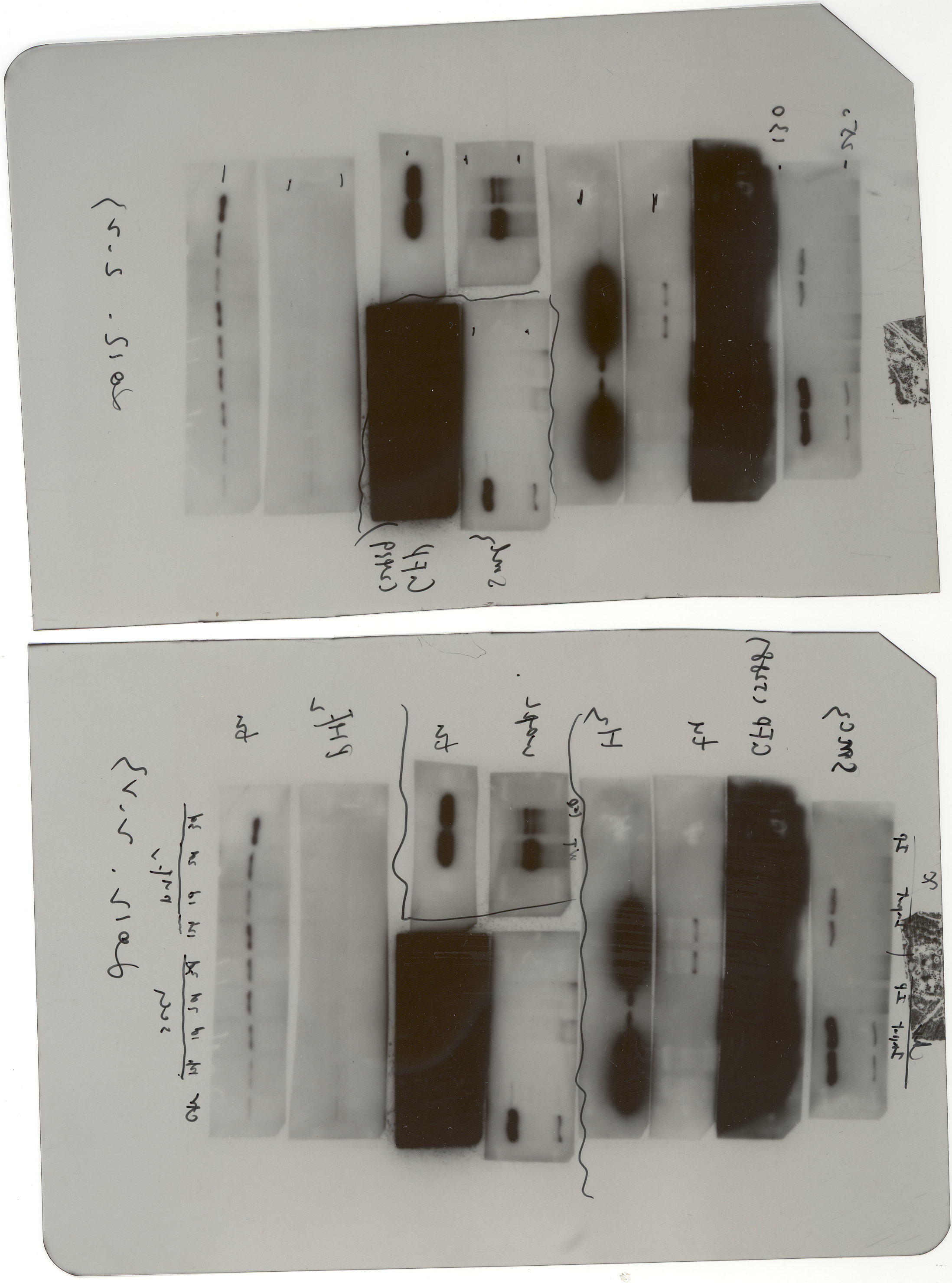

Supplement: Supplementary file 13 — Figures EV and Appendix Source Data [file 44318_2024_348_MOESM13_ESM.zip › SD figure EV and Appendix/Appendix Figure 2A/Untitled-2.tif]

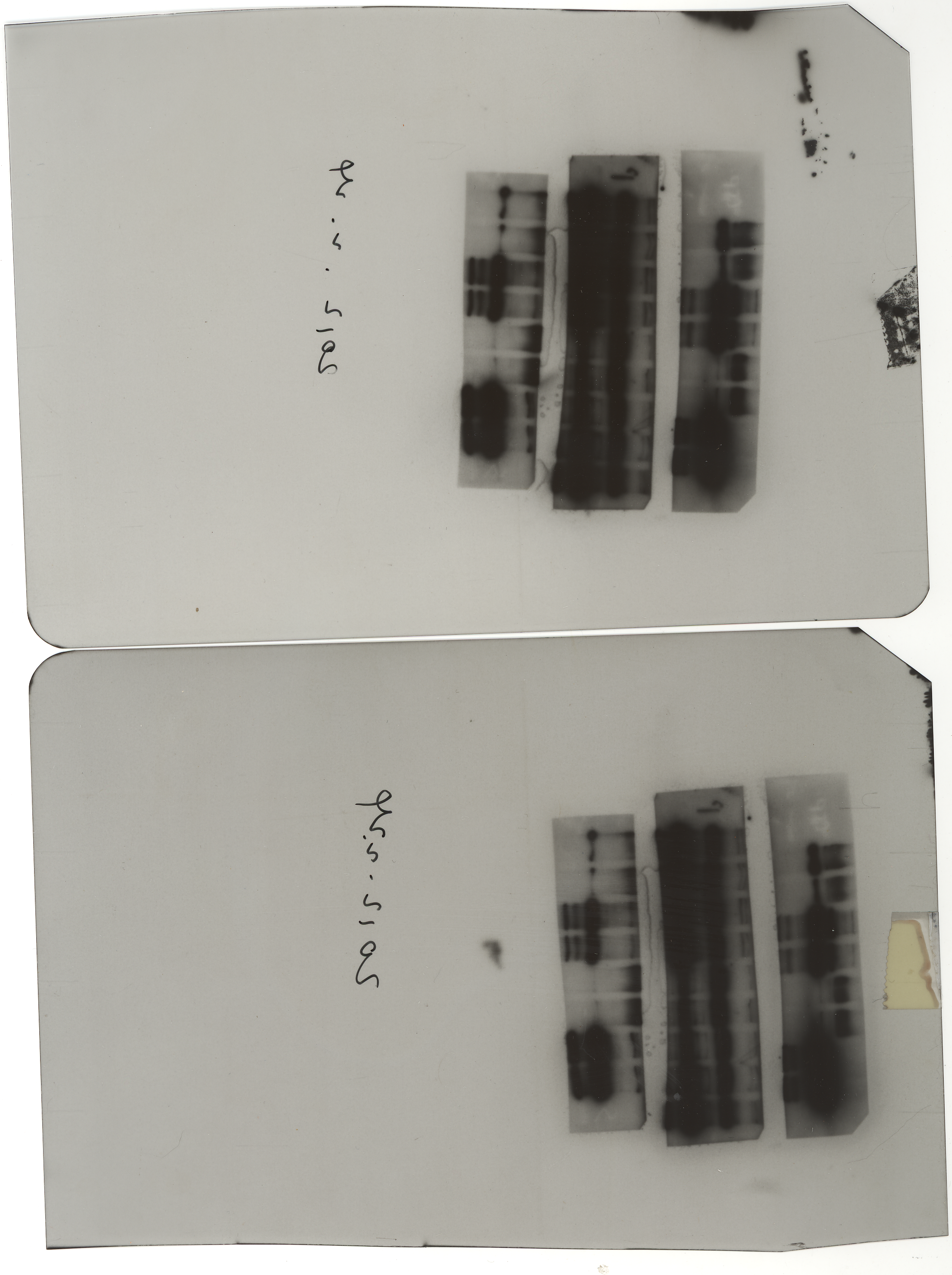

Supplement: Supplementary file 13 — Figures EV and Appendix Source Data [file 44318_2024_348_MOESM13_ESM.zip › SD figure EV and Appendix/Appendix Figure 2A/Untitled-3.tif]

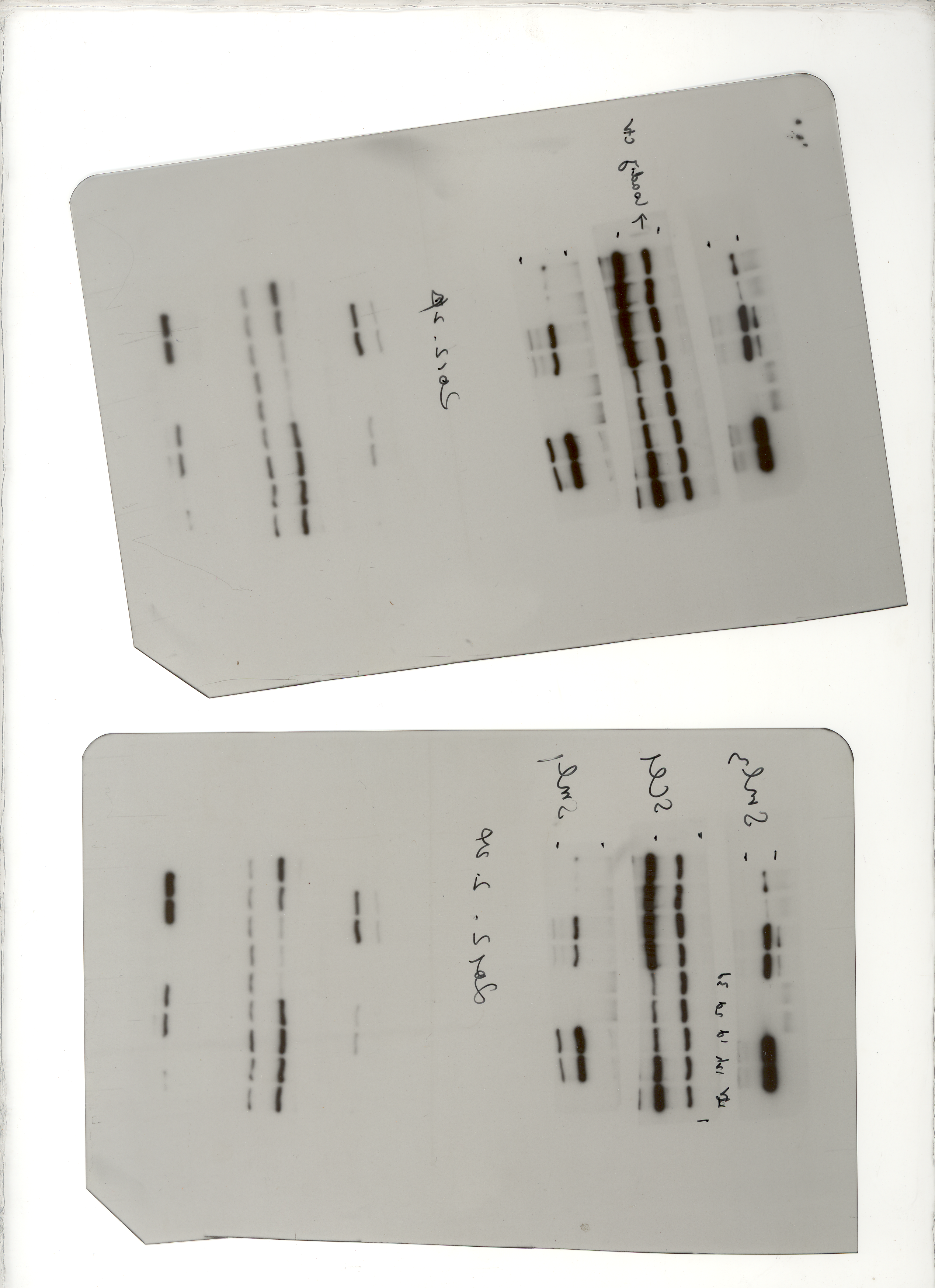

Supplement: Supplementary file 13 — Figures EV and Appendix Source Data [file 44318_2024_348_MOESM13_ESM.zip › SD figure EV and Appendix/Appendix Figure 2A/Untitled-4.tif]

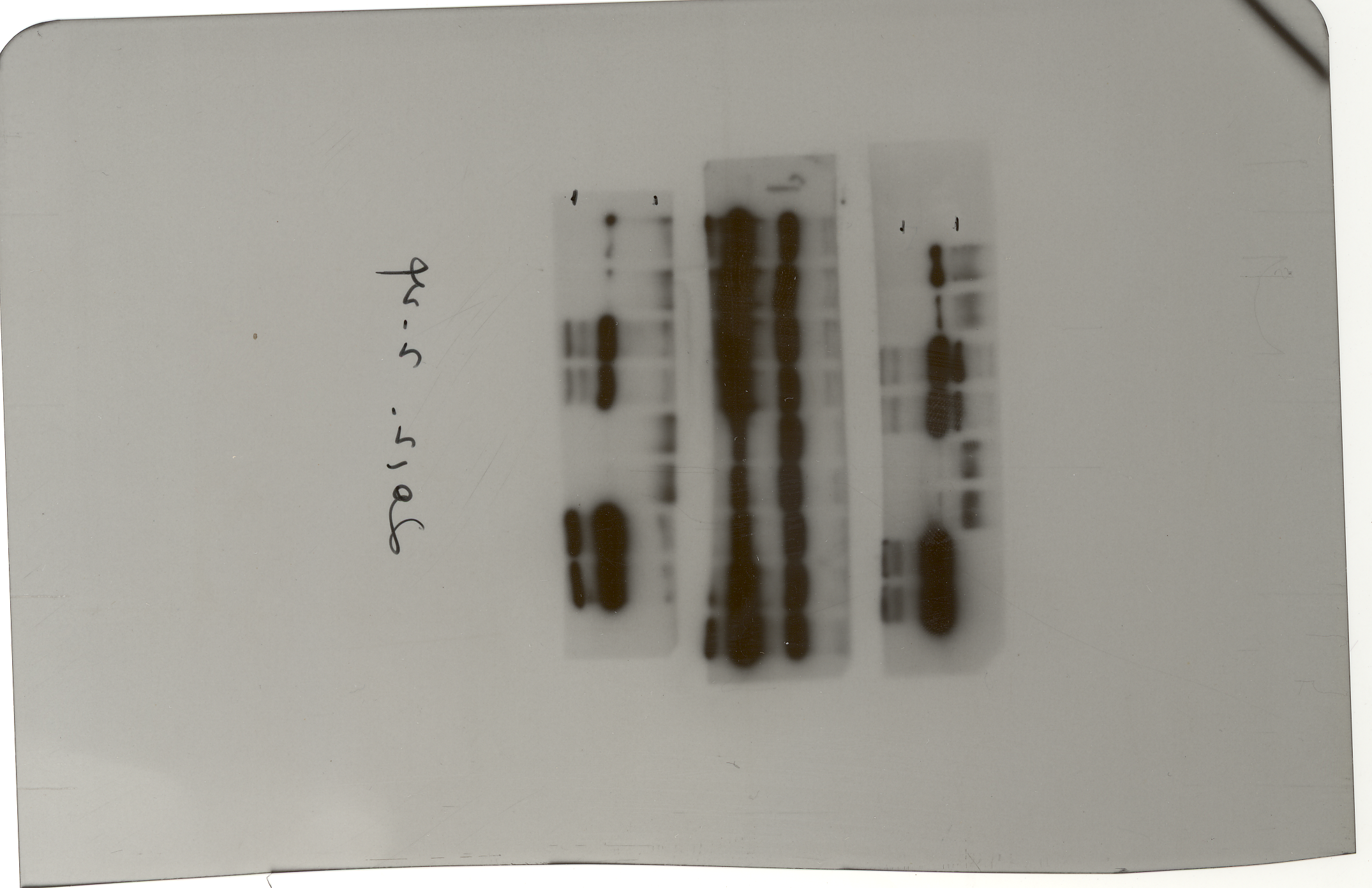

Supplement: Supplementary file 13 — Figures EV and Appendix Source Data [file 44318_2024_348_MOESM13_ESM.zip › SD figure EV and Appendix/Appendix Figure 2A/Untitled-5.tif]

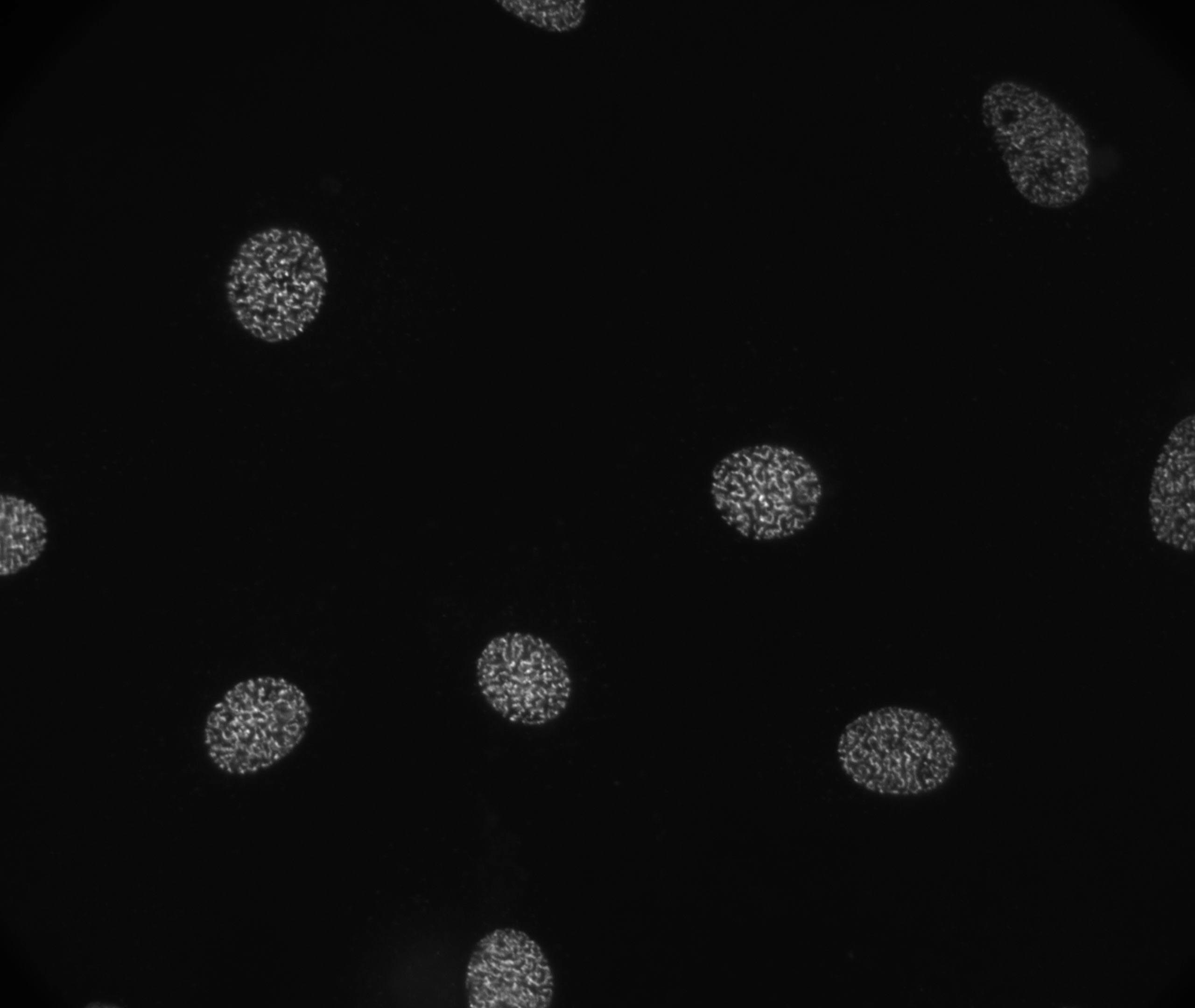

Supplement: Supplementary file 13 — Figures EV and Appendix Source Data [file 44318_2024_348_MOESM13_ESM.zip › SD figure EV and Appendix/EV3F/H3K4me2/560-2.jpg]

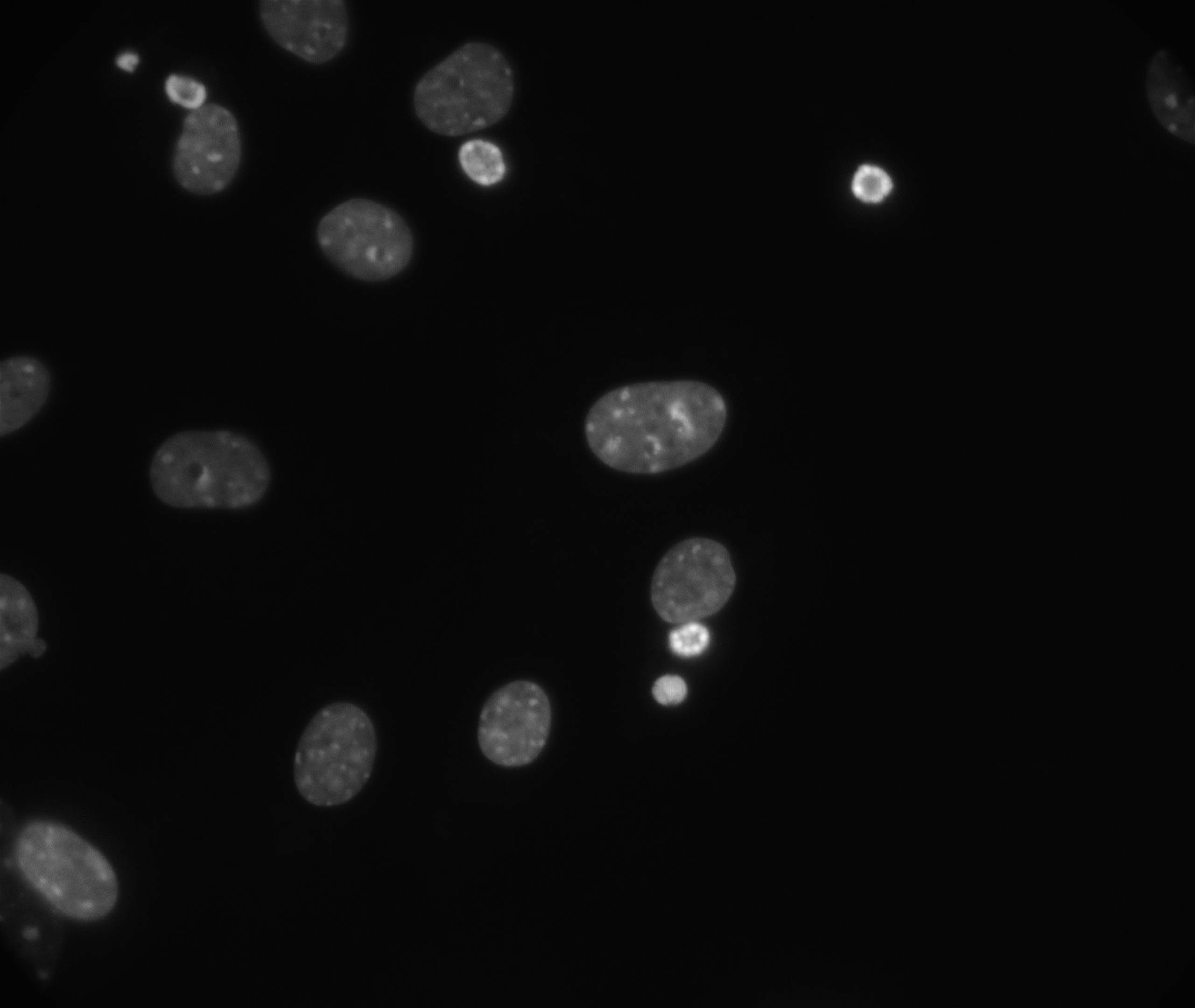

Supplement: Supplementary file 13 — Figures EV and Appendix Source Data [file 44318_2024_348_MOESM13_ESM.zip › SD figure EV and Appendix/EV3F/H3K4me2/360.jpg]

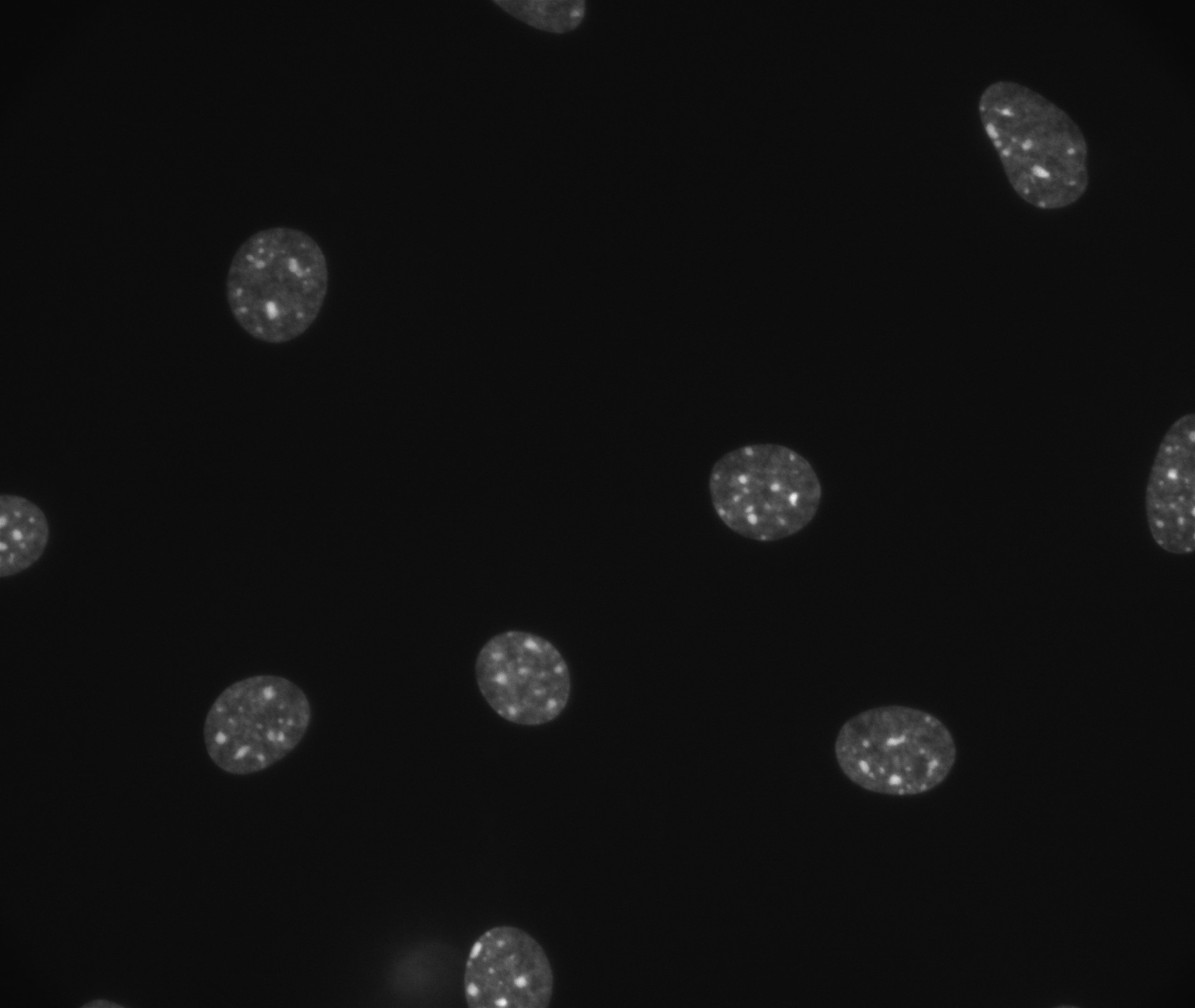

Supplement: Supplementary file 13 — Figures EV and Appendix Source Data [file 44318_2024_348_MOESM13_ESM.zip › SD figure EV and Appendix/EV3F/H3K4me2/360-2.jpg]

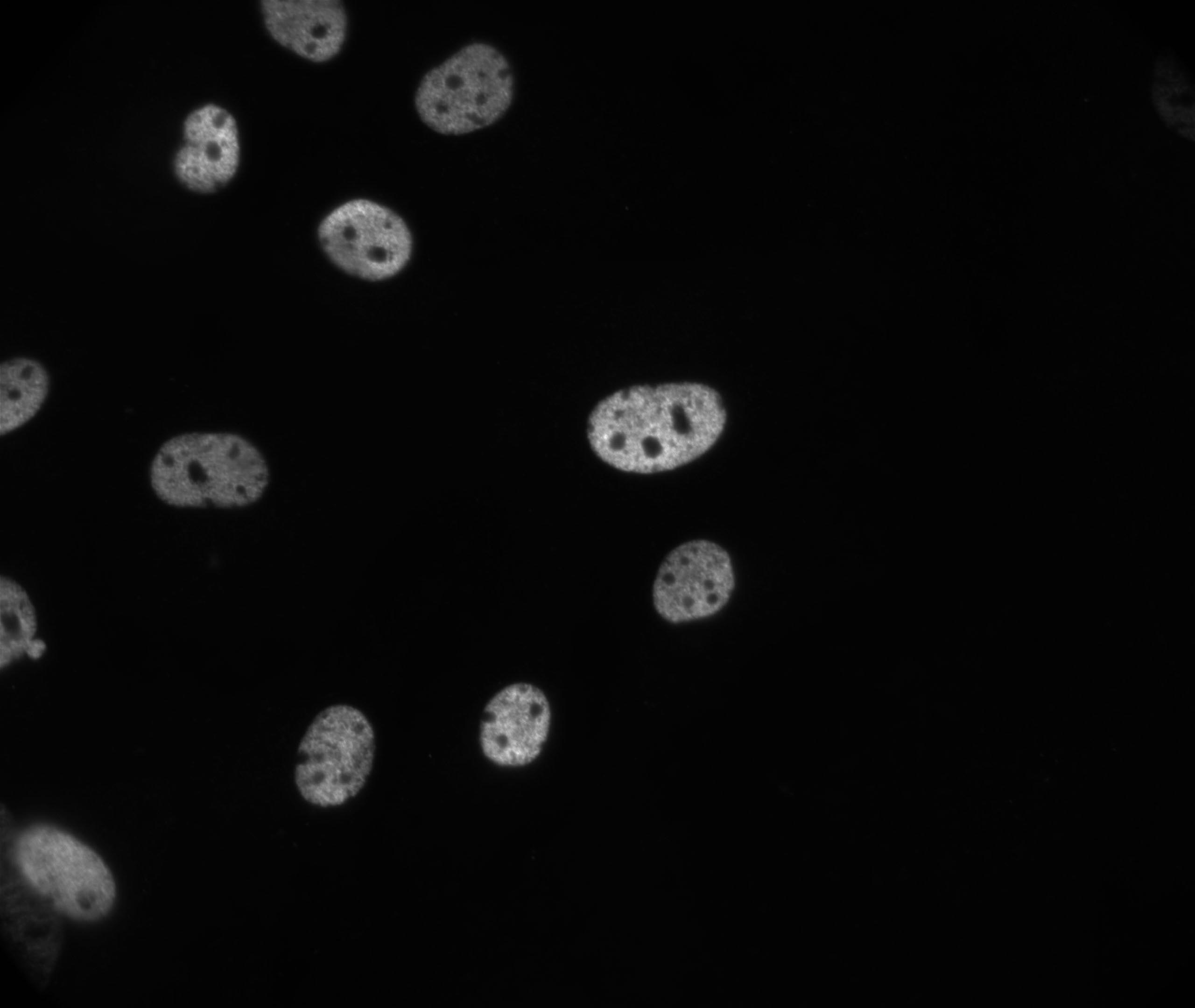

Supplement: Supplementary file 13 — Figures EV and Appendix Source Data [file 44318_2024_348_MOESM13_ESM.zip › SD figure EV and Appendix/EV3F/H3K4me2/480.jpg]

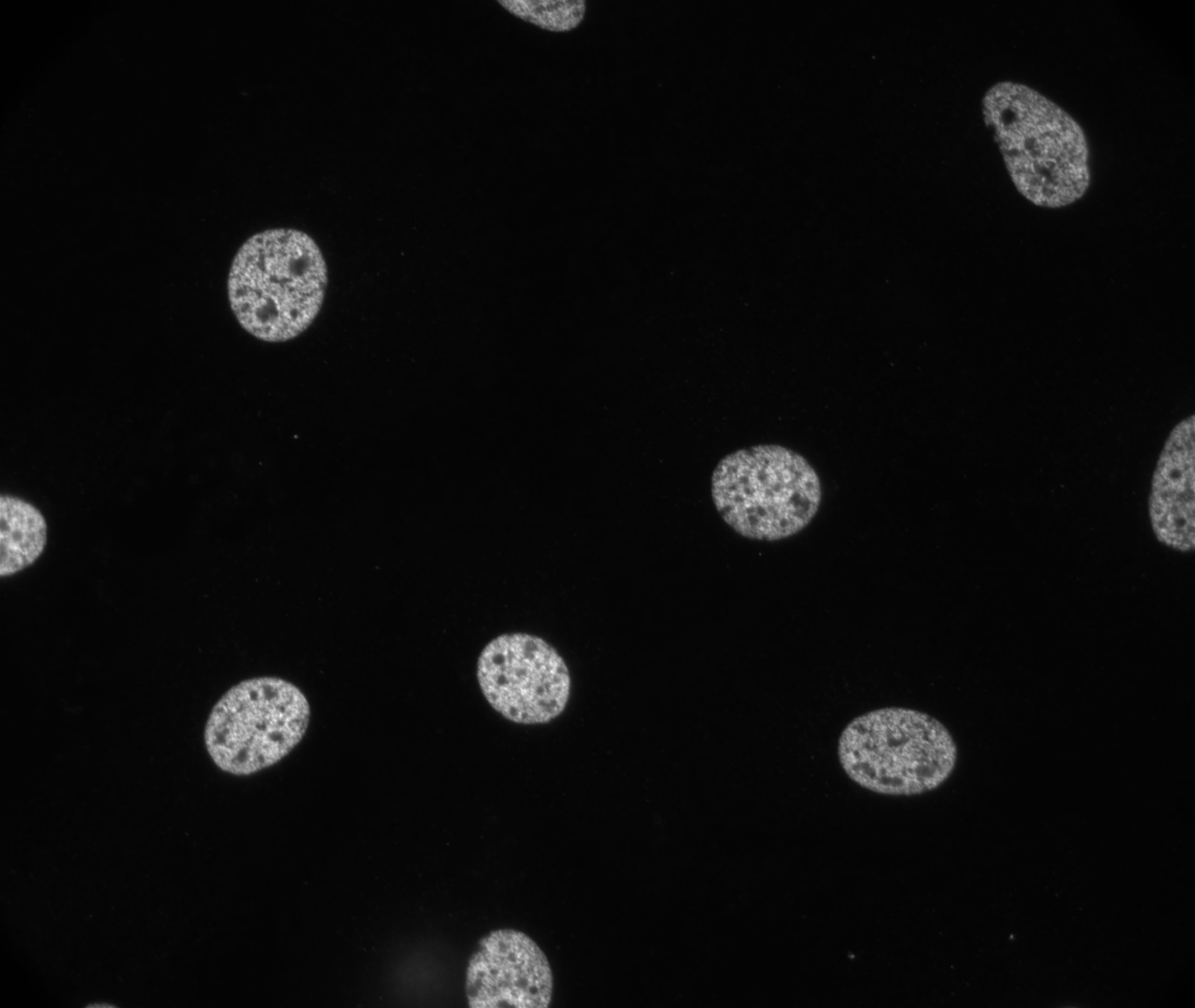

Supplement: Supplementary file 13 — Figures EV and Appendix Source Data [file 44318_2024_348_MOESM13_ESM.zip › SD figure EV and Appendix/EV3F/H3K4me2/480-2.jpg]

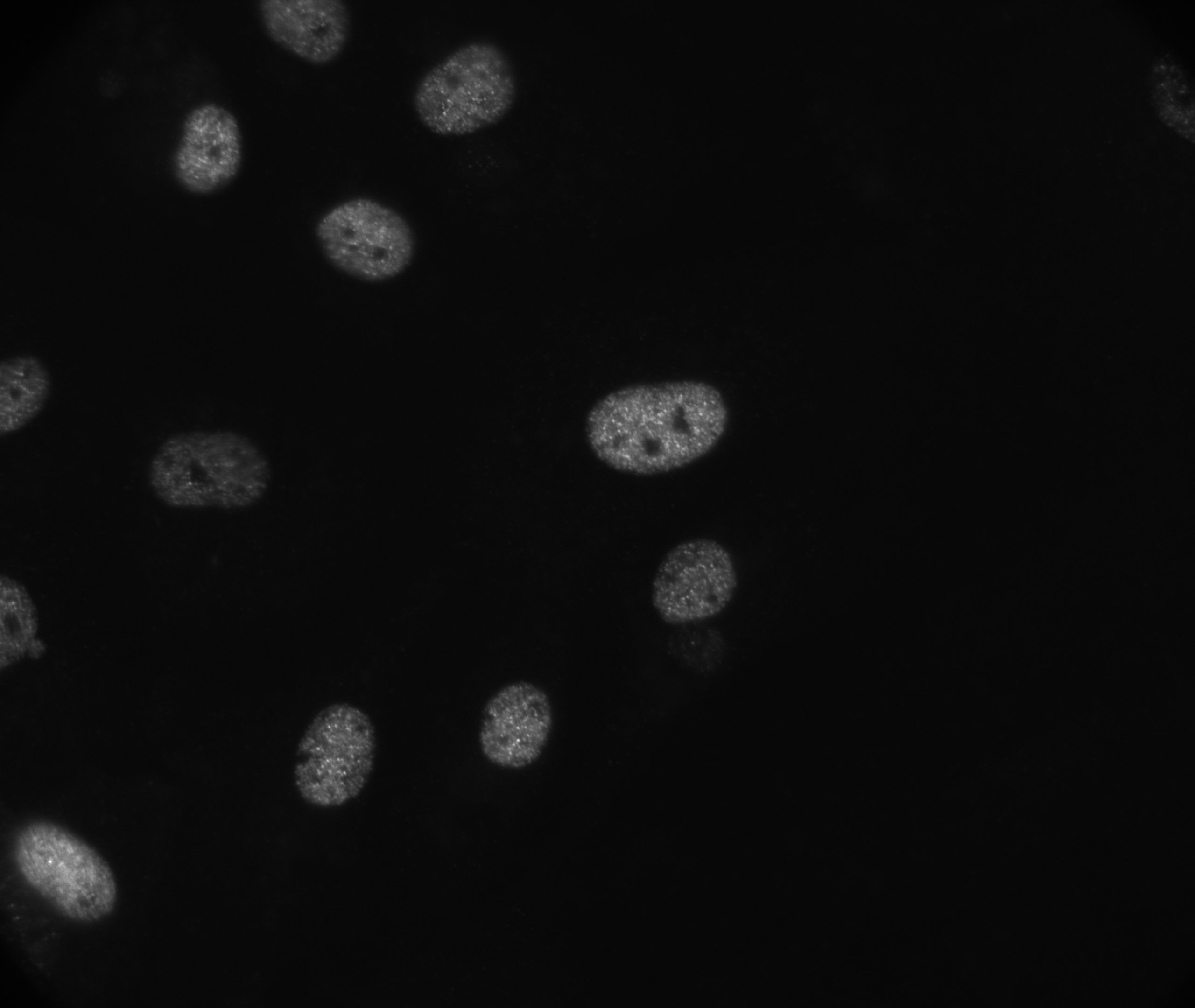

Supplement: Supplementary file 13 — Figures EV and Appendix Source Data [file 44318_2024_348_MOESM13_ESM.zip › SD figure EV and Appendix/EV3F/H3K4me2/560.jpg]

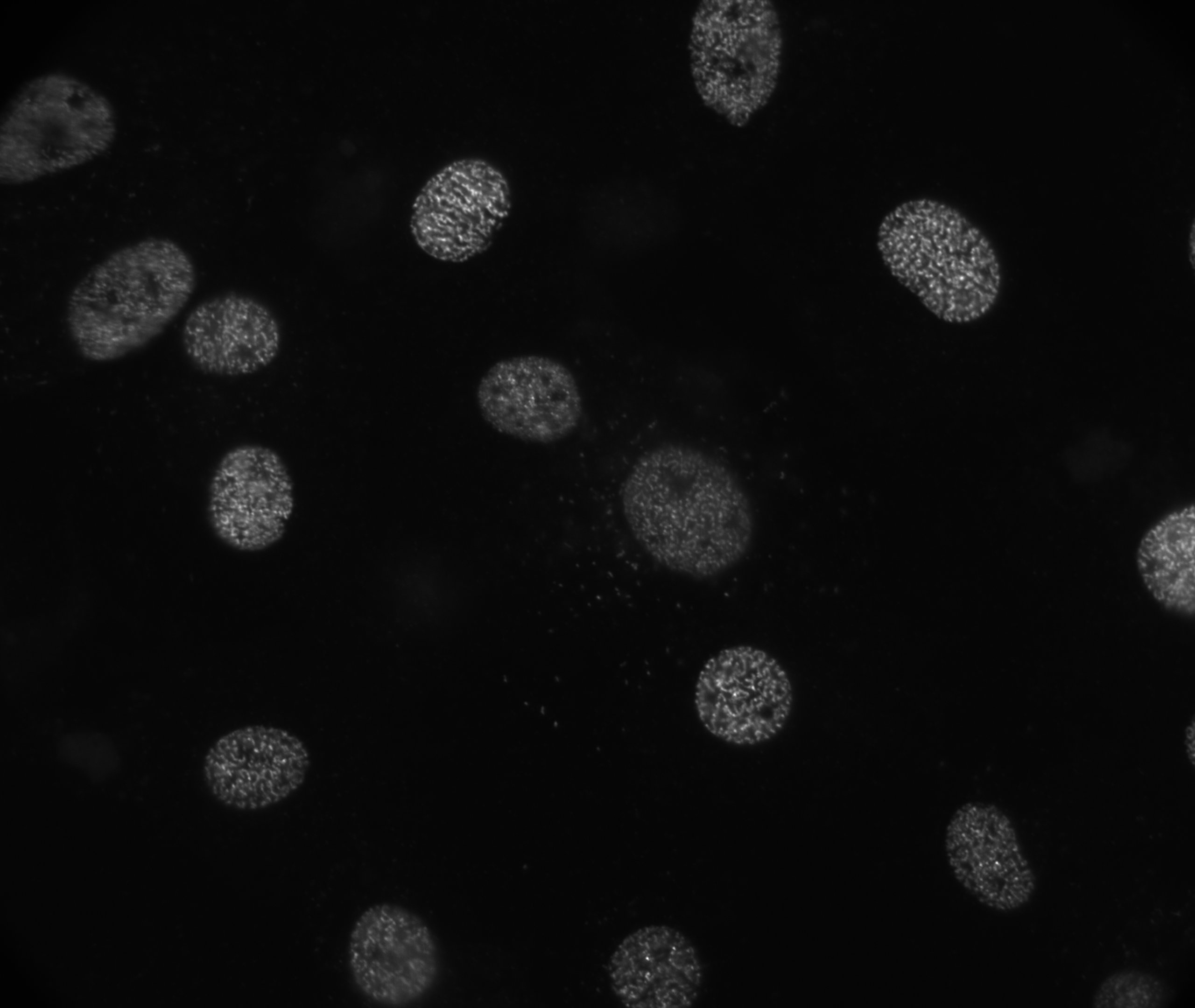

Supplement: Supplementary file 13 — Figures EV and Appendix Source Data [file 44318_2024_348_MOESM13_ESM.zip › SD figure EV and Appendix/EV3F/H3K4me3/560-2.jpg]

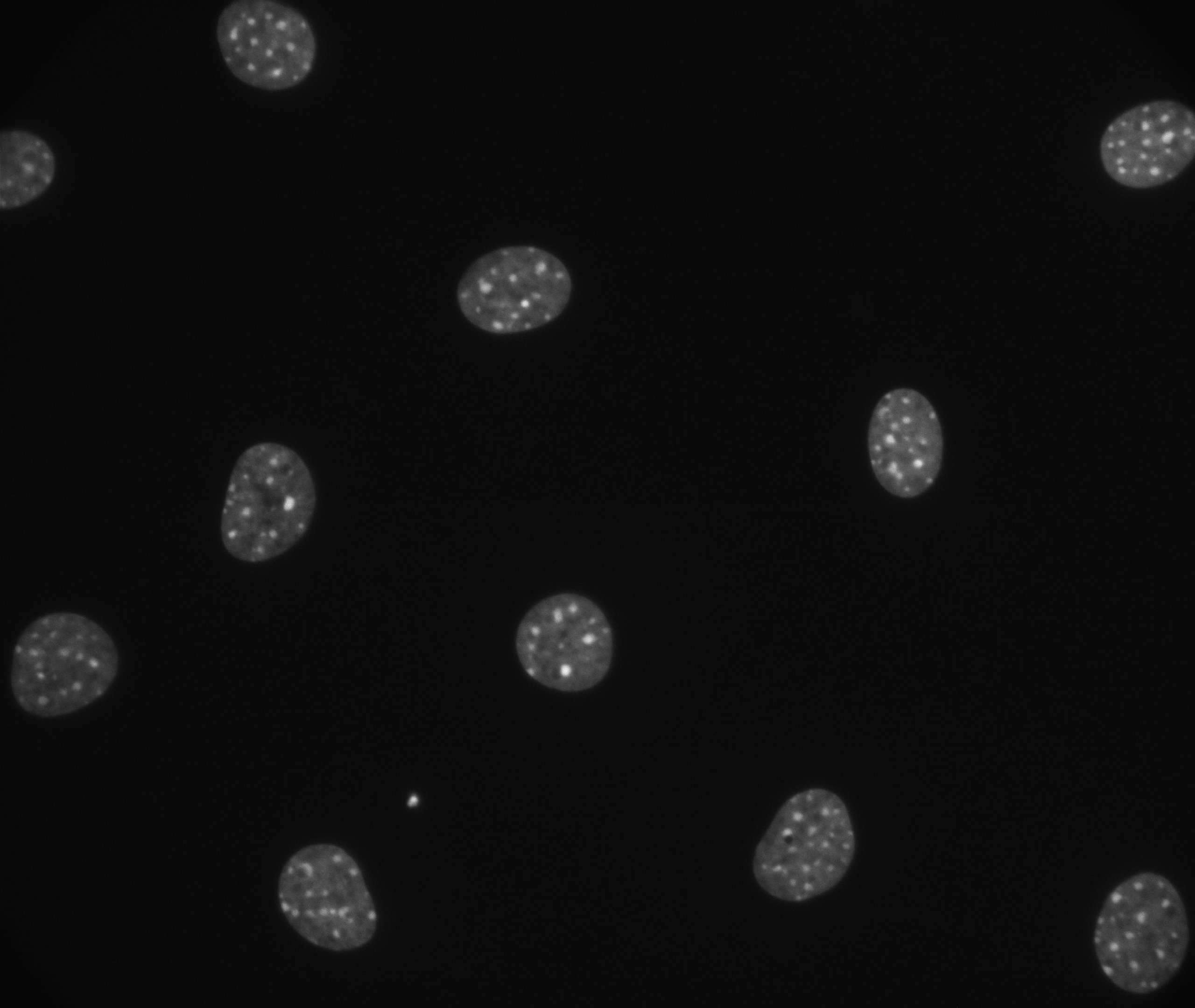

Supplement: Supplementary file 13 — Figures EV and Appendix Source Data [file 44318_2024_348_MOESM13_ESM.zip › SD figure EV and Appendix/EV3F/H3K4me3/360.jpg]

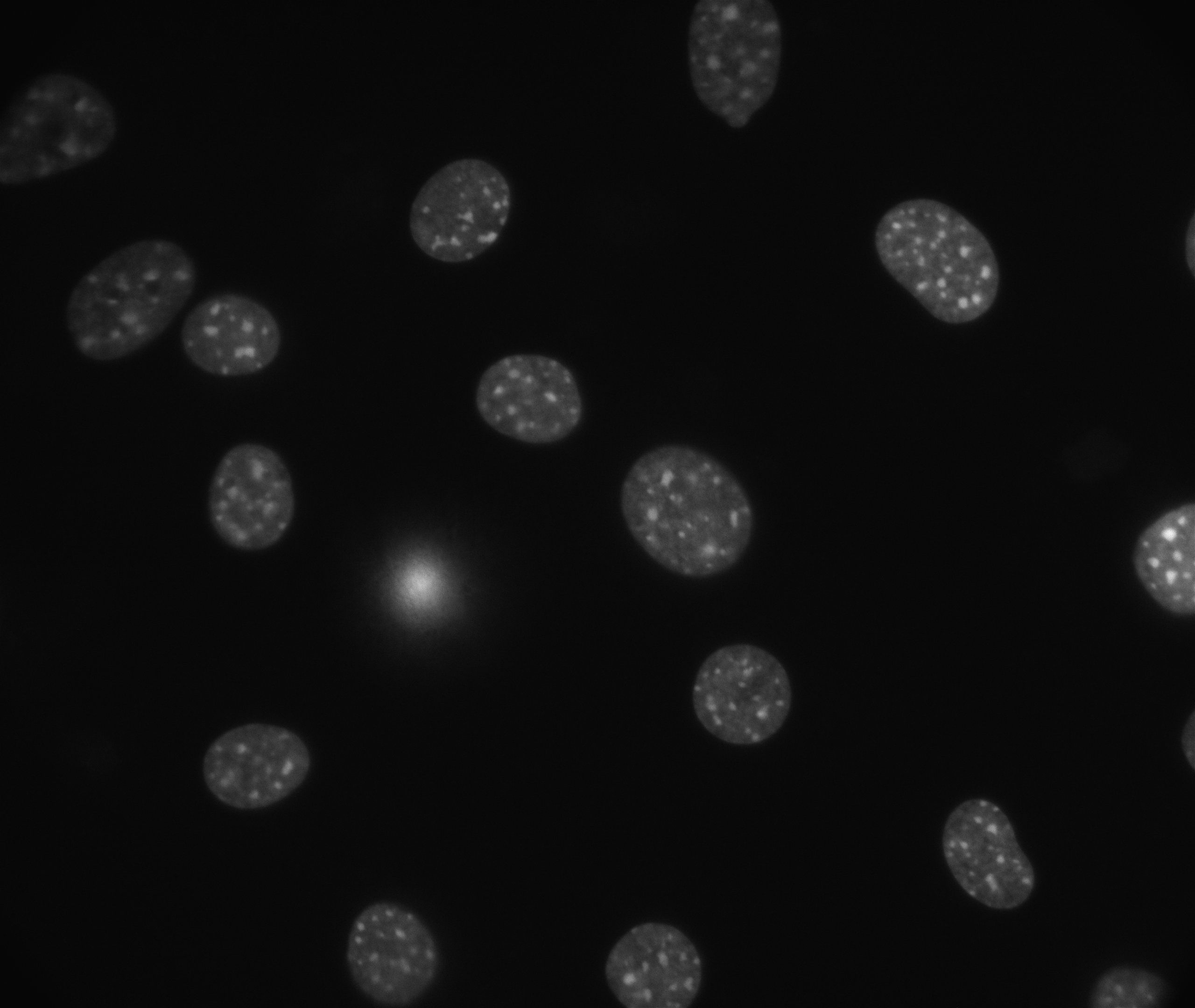

Supplement: Supplementary file 13 — Figures EV and Appendix Source Data [file 44318_2024_348_MOESM13_ESM.zip › SD figure EV and Appendix/EV3F/H3K4me3/360-2.jpg]

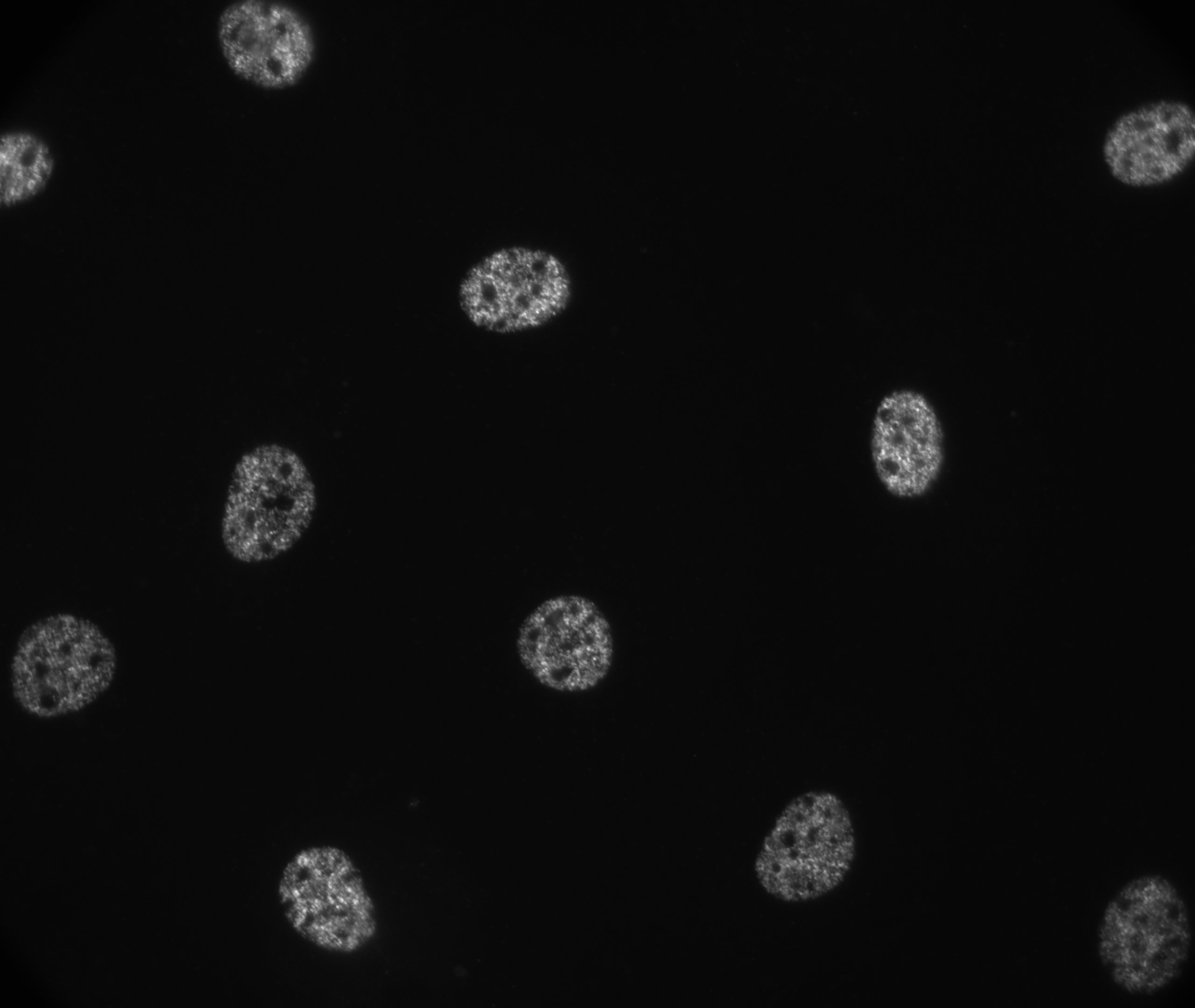

Supplement: Supplementary file 13 — Figures EV and Appendix Source Data [file 44318_2024_348_MOESM13_ESM.zip › SD figure EV and Appendix/EV3F/H3K4me3/480.jpg]

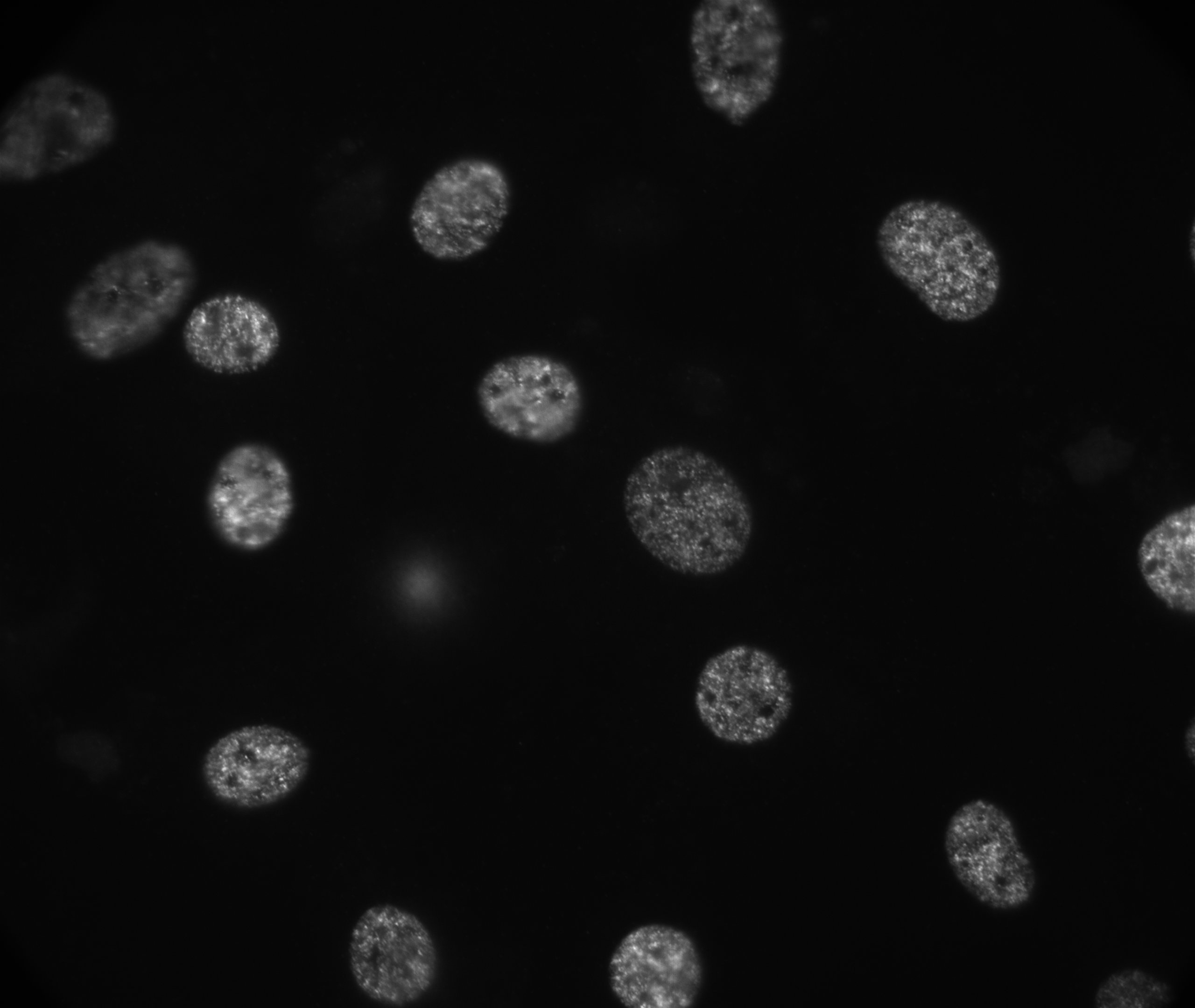

Supplement: Supplementary file 13 — Figures EV and Appendix Source Data [file 44318_2024_348_MOESM13_ESM.zip › SD figure EV and Appendix/EV3F/H3K4me3/480-2.jpg]

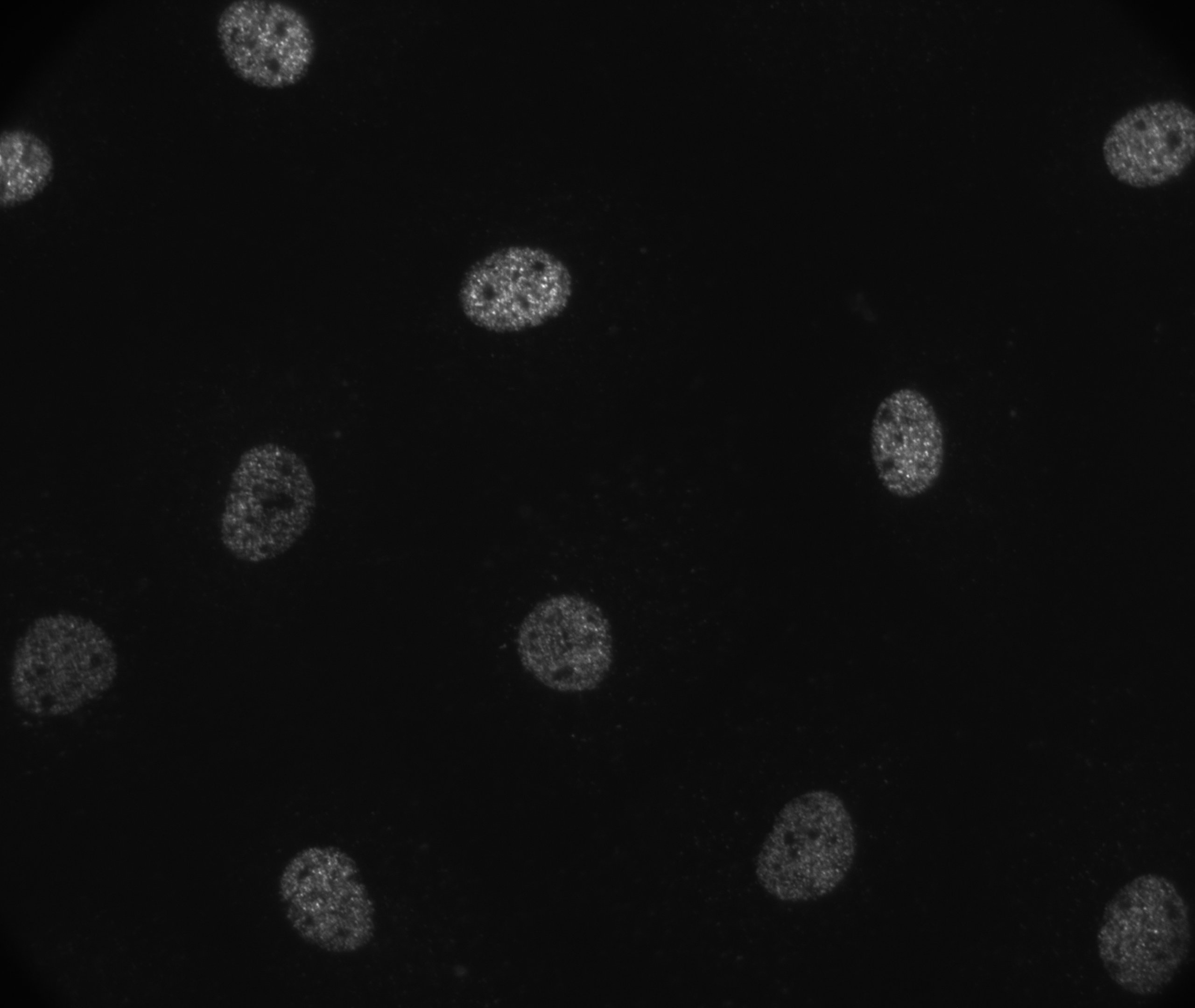

Supplement: Supplementary file 13 — Figures EV and Appendix Source Data [file 44318_2024_348_MOESM13_ESM.zip › SD figure EV and Appendix/EV3F/H3K4me3/560.jpg]

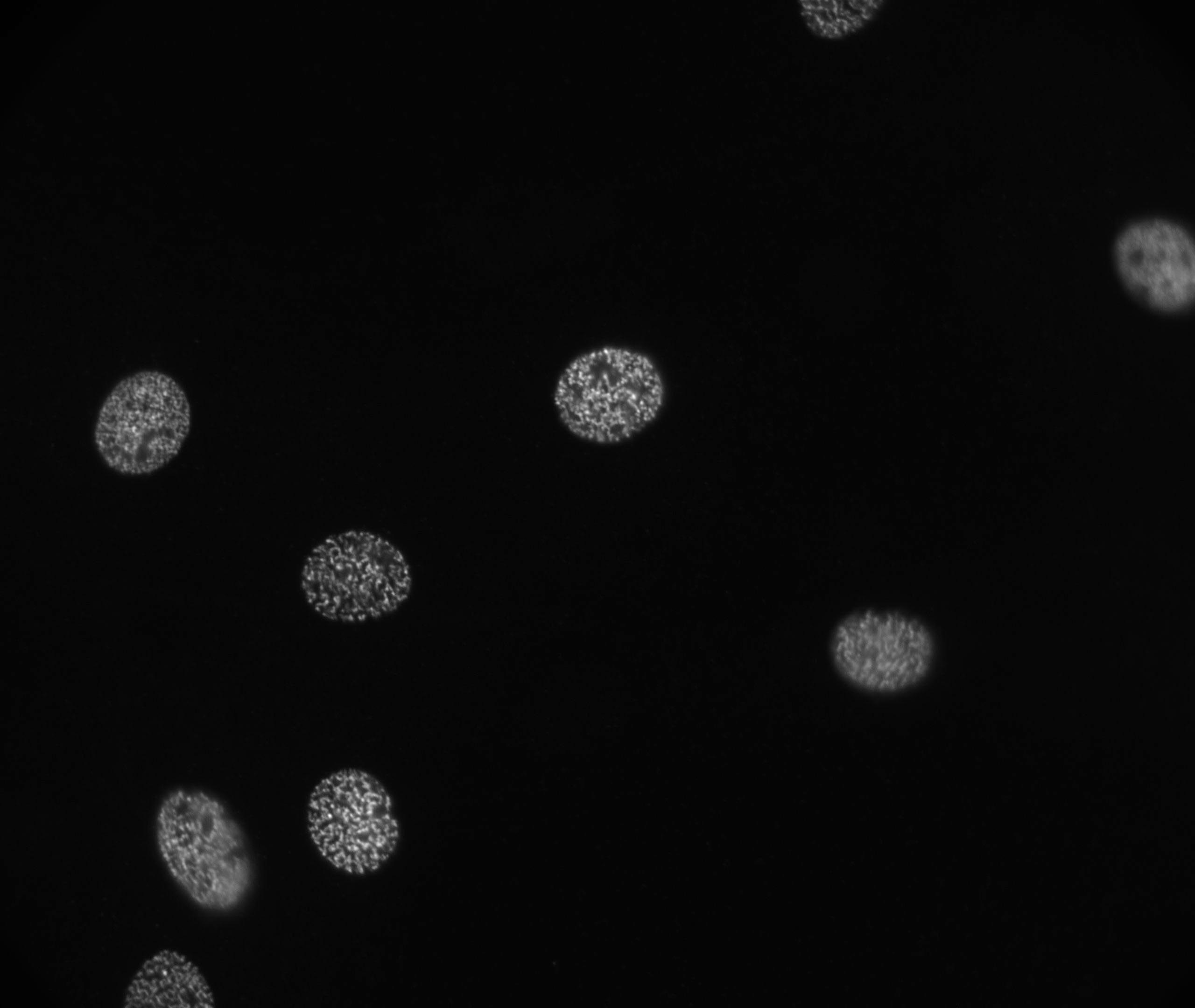

Supplement: Supplementary file 13 — Figures EV and Appendix Source Data [file 44318_2024_348_MOESM13_ESM.zip › SD figure EV and Appendix/EV3F/H3K9me1/560-2.jpg]

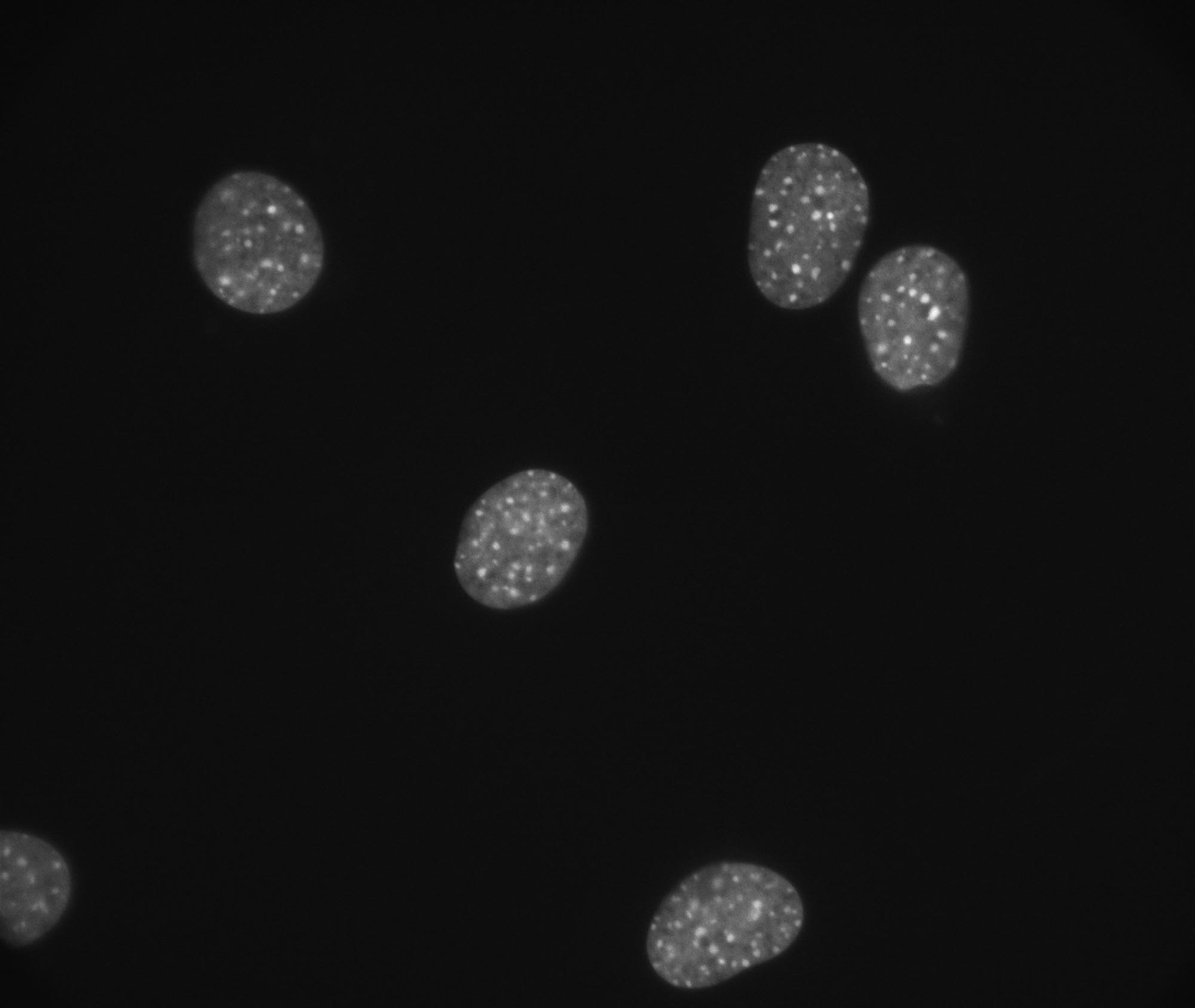

Supplement: Supplementary file 13 — Figures EV and Appendix Source Data [file 44318_2024_348_MOESM13_ESM.zip › SD figure EV and Appendix/EV3F/H3K9me1/360.jpg]

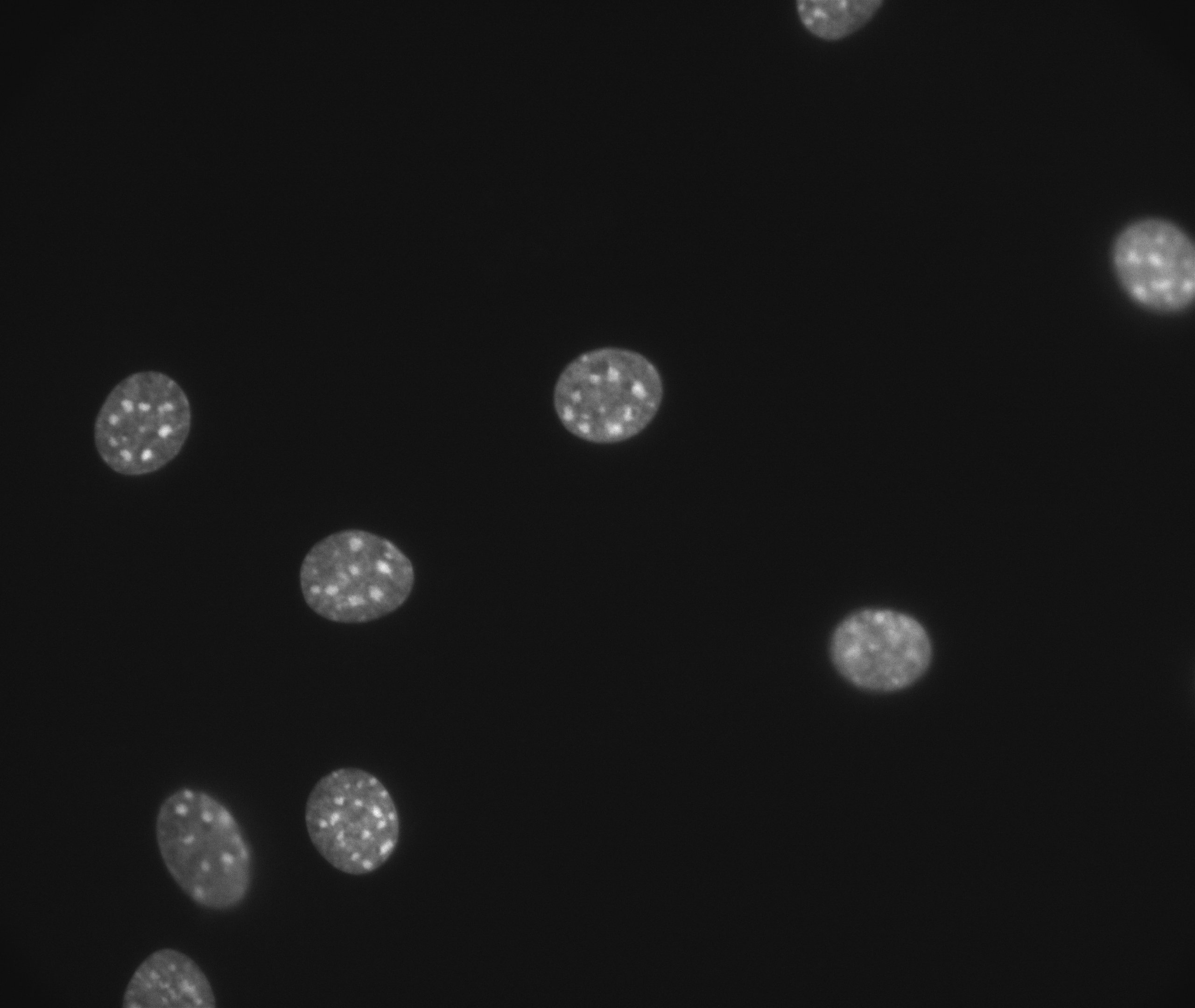

Supplement: Supplementary file 13 — Figures EV and Appendix Source Data [file 44318_2024_348_MOESM13_ESM.zip › SD figure EV and Appendix/EV3F/H3K9me1/360-2.jpg]

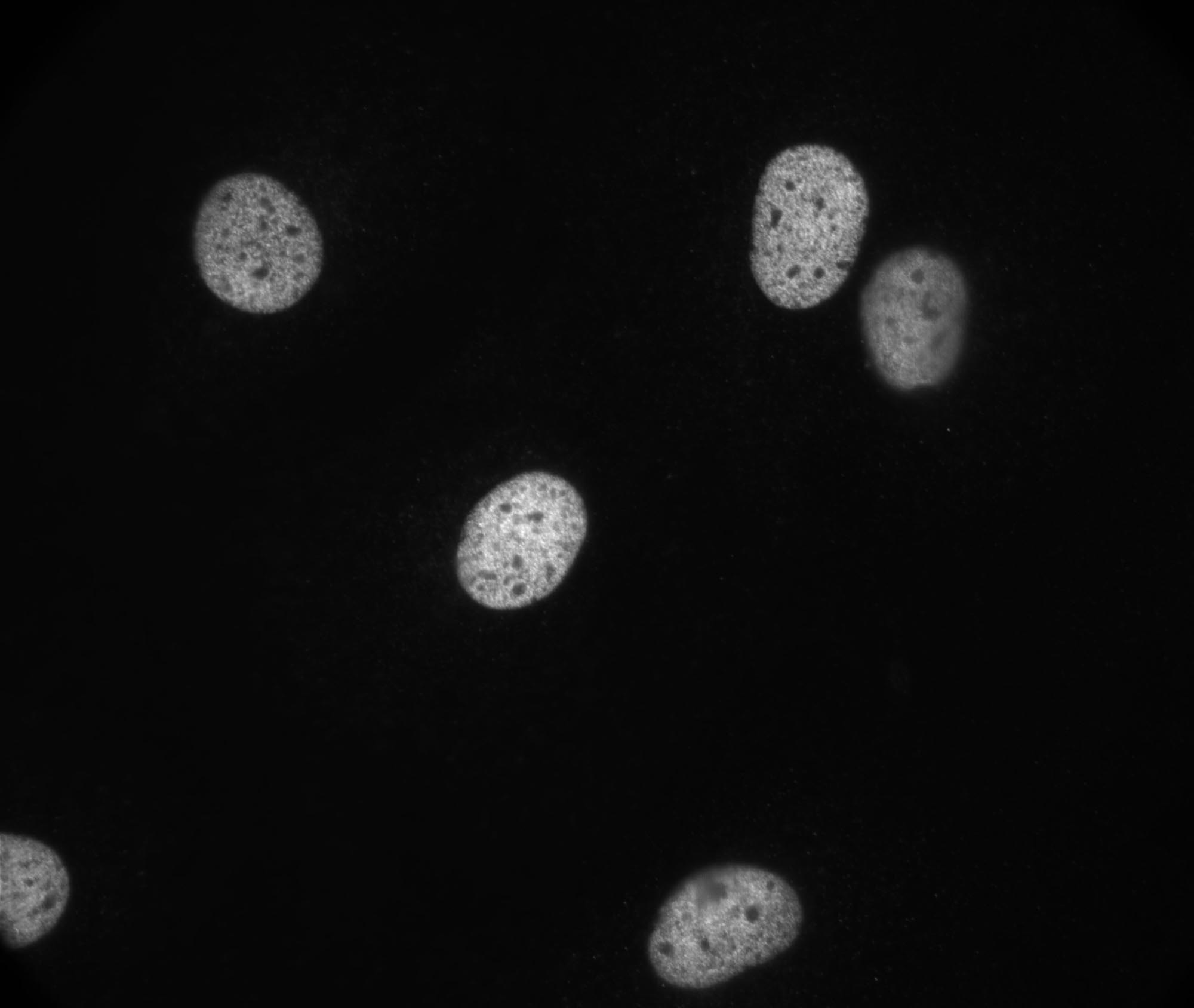

Supplement: Supplementary file 13 — Figures EV and Appendix Source Data [file 44318_2024_348_MOESM13_ESM.zip › SD figure EV and Appendix/EV3F/H3K9me1/480.jpg]

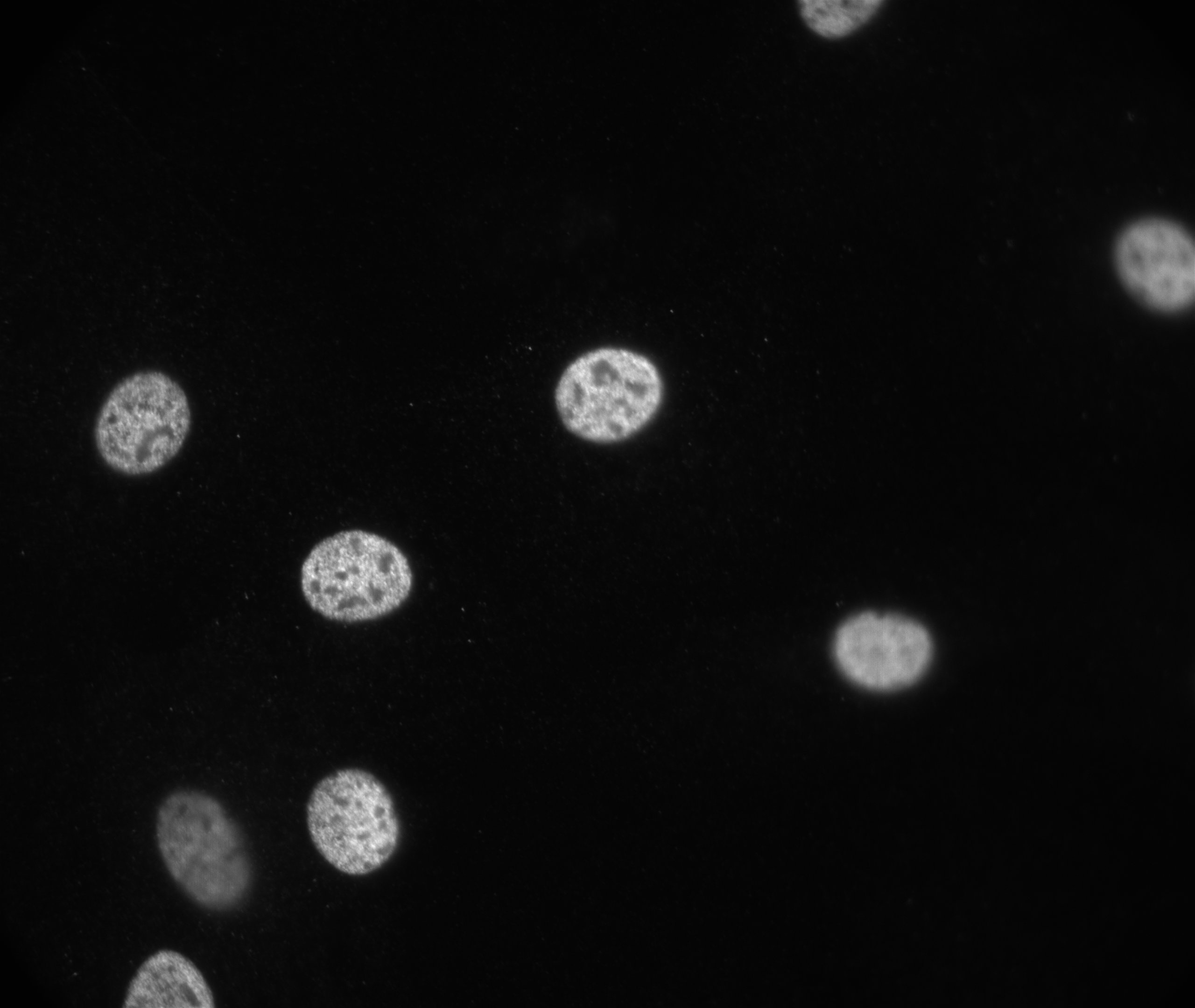

Supplement: Supplementary file 13 — Figures EV and Appendix Source Data [file 44318_2024_348_MOESM13_ESM.zip › SD figure EV and Appendix/EV3F/H3K9me1/480-2.jpg]

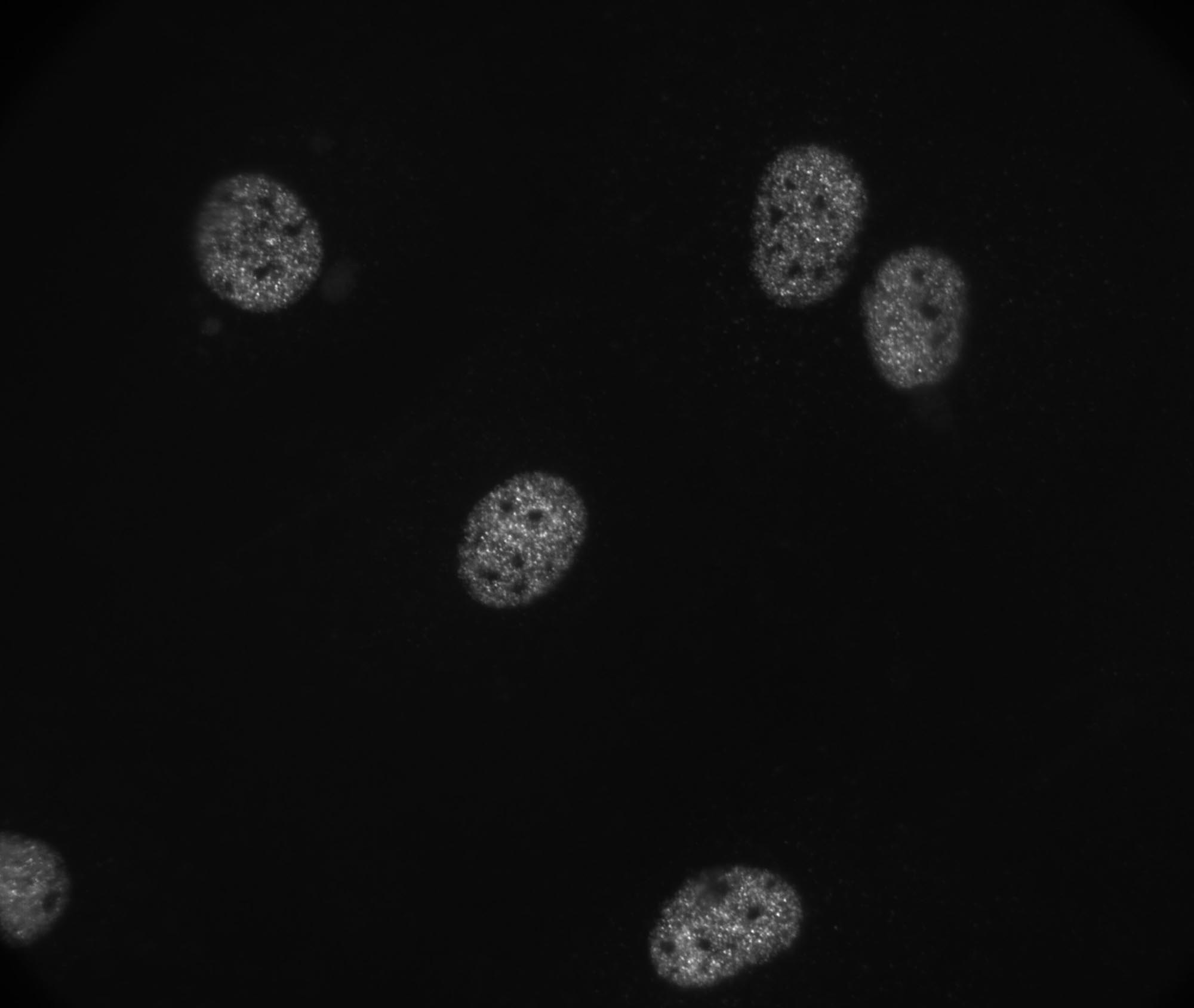

Supplement: Supplementary file 13 — Figures EV and Appendix Source Data [file 44318_2024_348_MOESM13_ESM.zip › SD figure EV and Appendix/EV3F/H3K9me1/560.jpg]

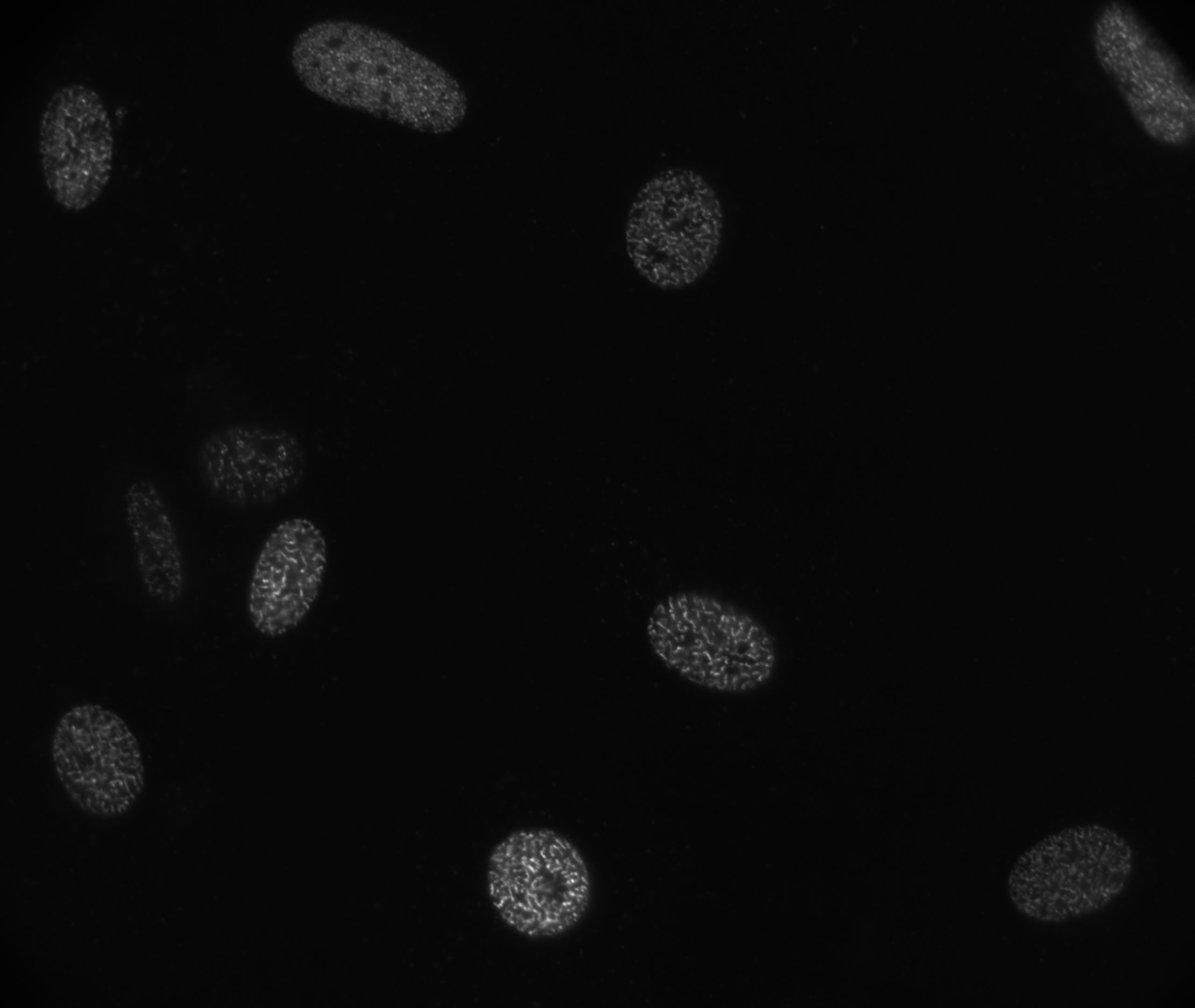

Supplement: Supplementary file 13 — Figures EV and Appendix Source Data [file 44318_2024_348_MOESM13_ESM.zip › SD figure EV and Appendix/EV3F/H3K9me2/560-2.jpg]

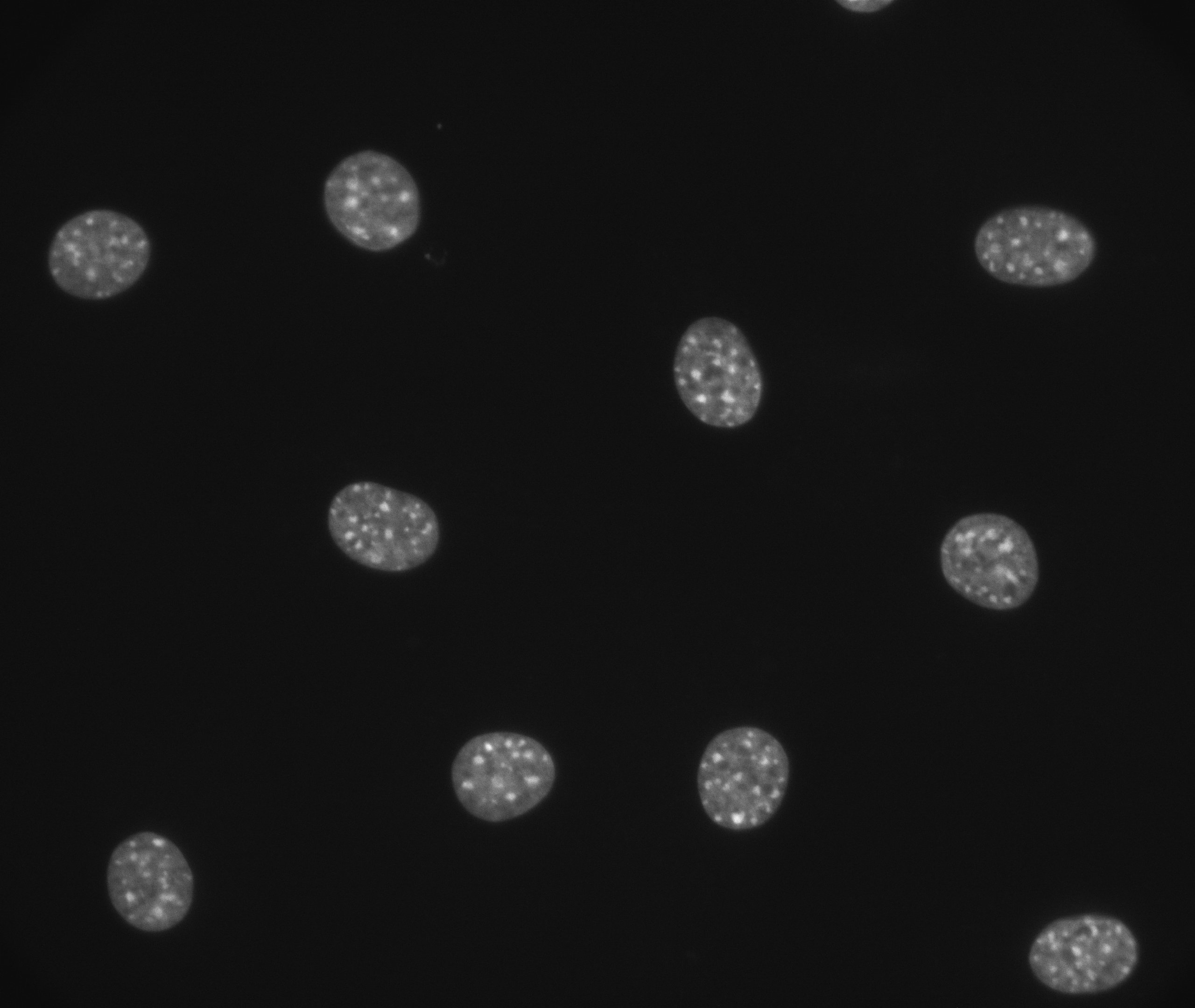

Supplement: Supplementary file 13 — Figures EV and Appendix Source Data [file 44318_2024_348_MOESM13_ESM.zip › SD figure EV and Appendix/EV3F/H3K9me2/360.jpg]

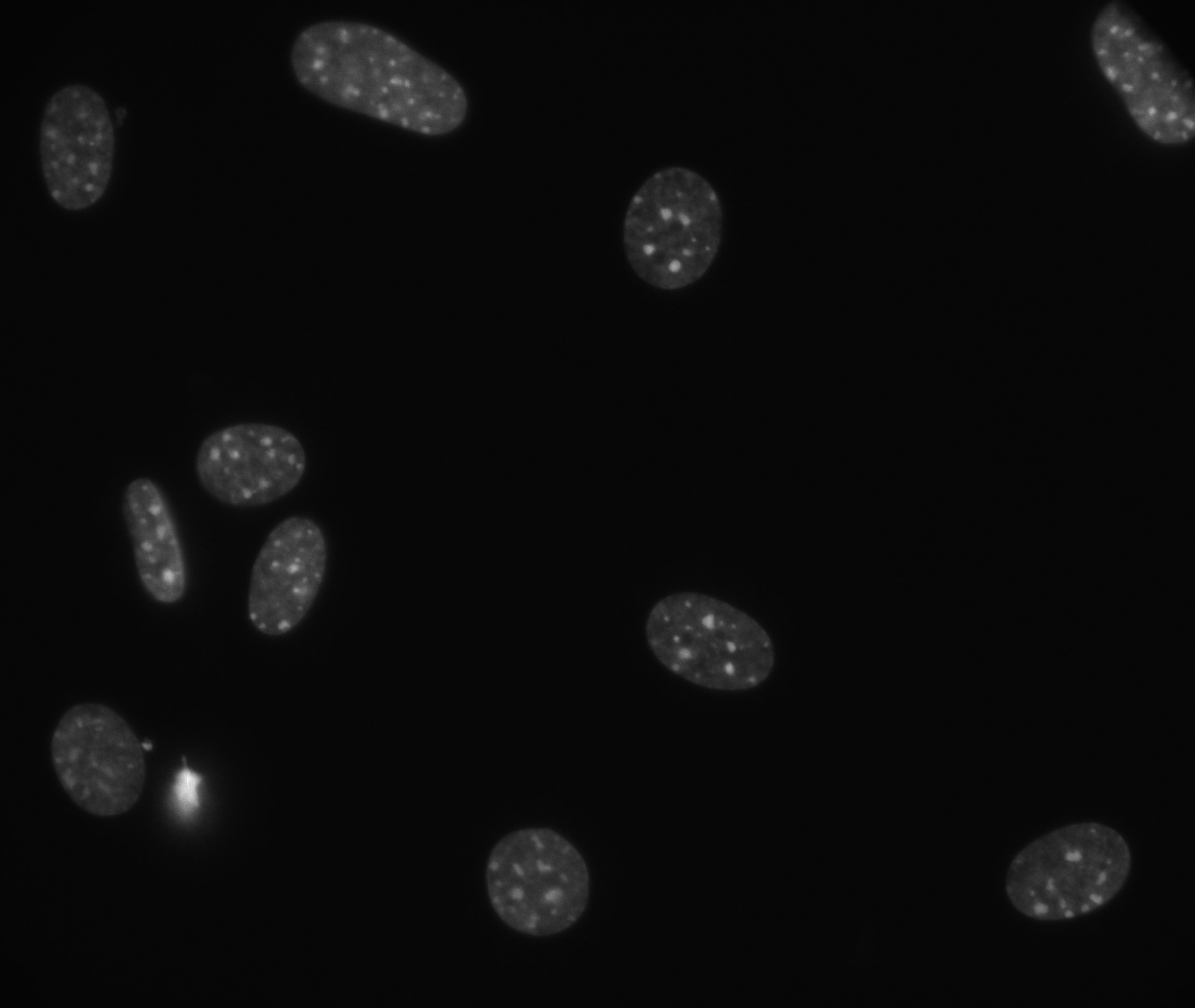

Supplement: Supplementary file 13 — Figures EV and Appendix Source Data [file 44318_2024_348_MOESM13_ESM.zip › SD figure EV and Appendix/EV3F/H3K9me2/360-2.jpg]

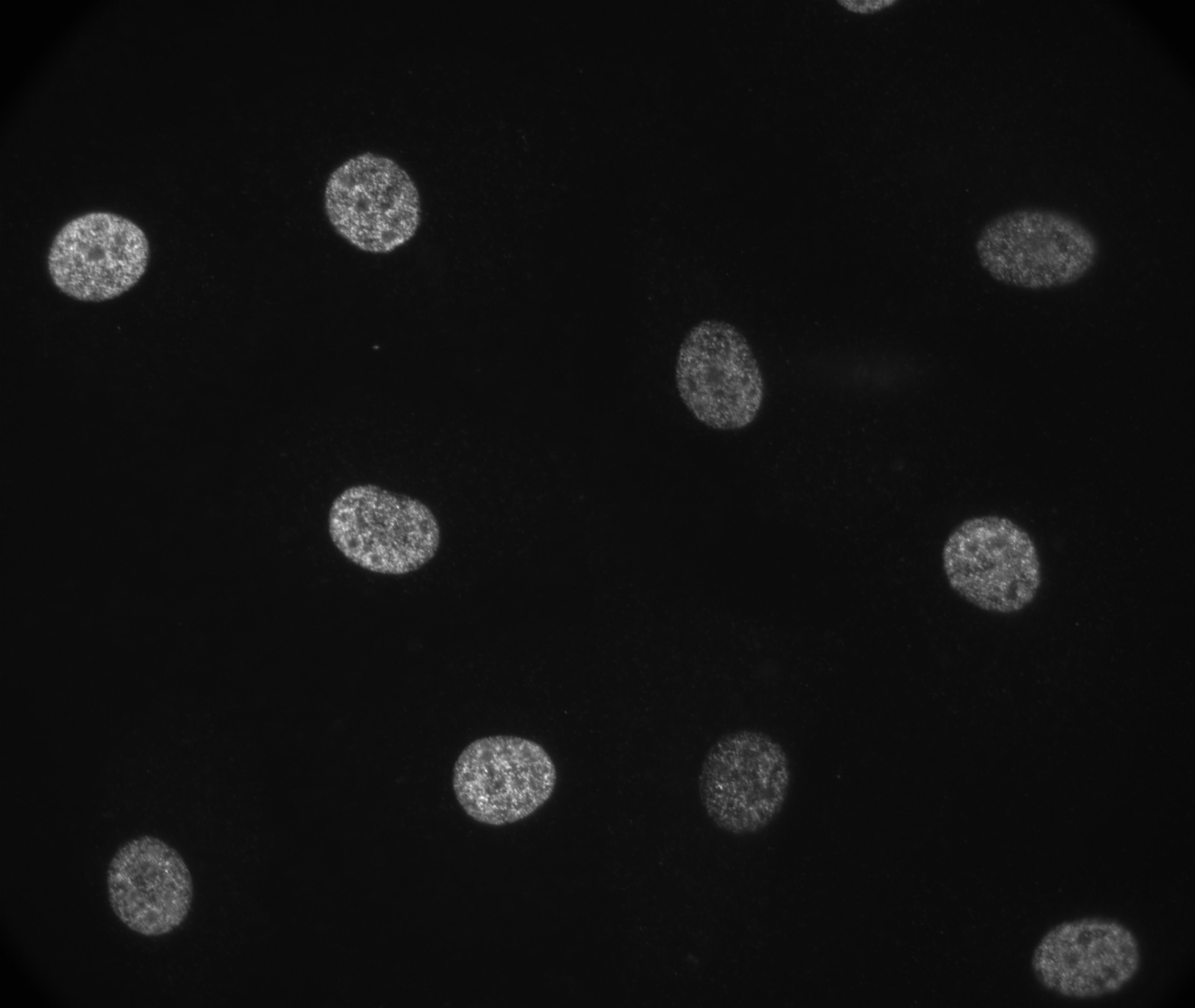

Supplement: Supplementary file 13 — Figures EV and Appendix Source Data [file 44318_2024_348_MOESM13_ESM.zip › SD figure EV and Appendix/EV3F/H3K9me2/480.jpg]

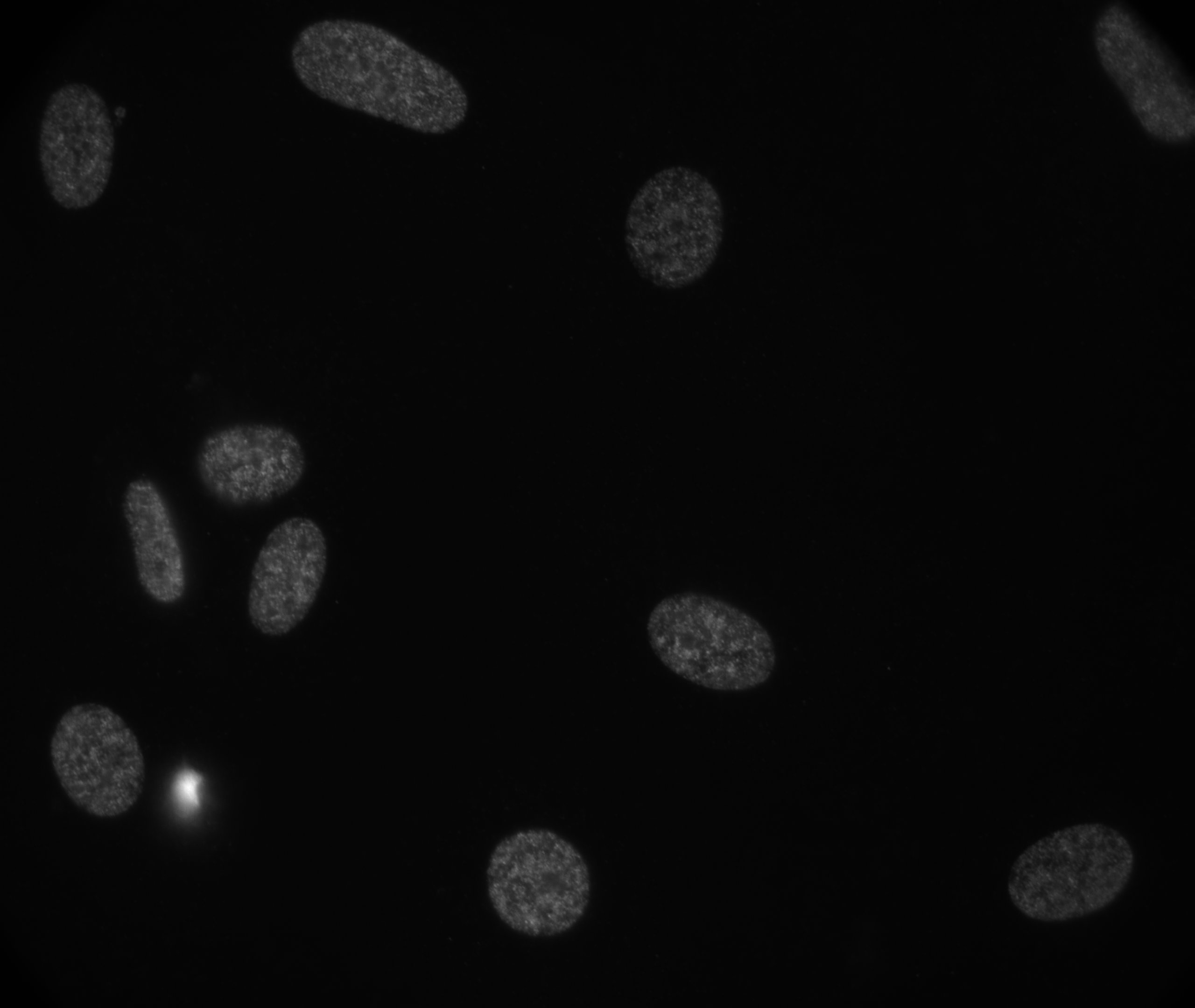

Supplement: Supplementary file 13 — Figures EV and Appendix Source Data [file 44318_2024_348_MOESM13_ESM.zip › SD figure EV and Appendix/EV3F/H3K9me2/480-2.jpg]

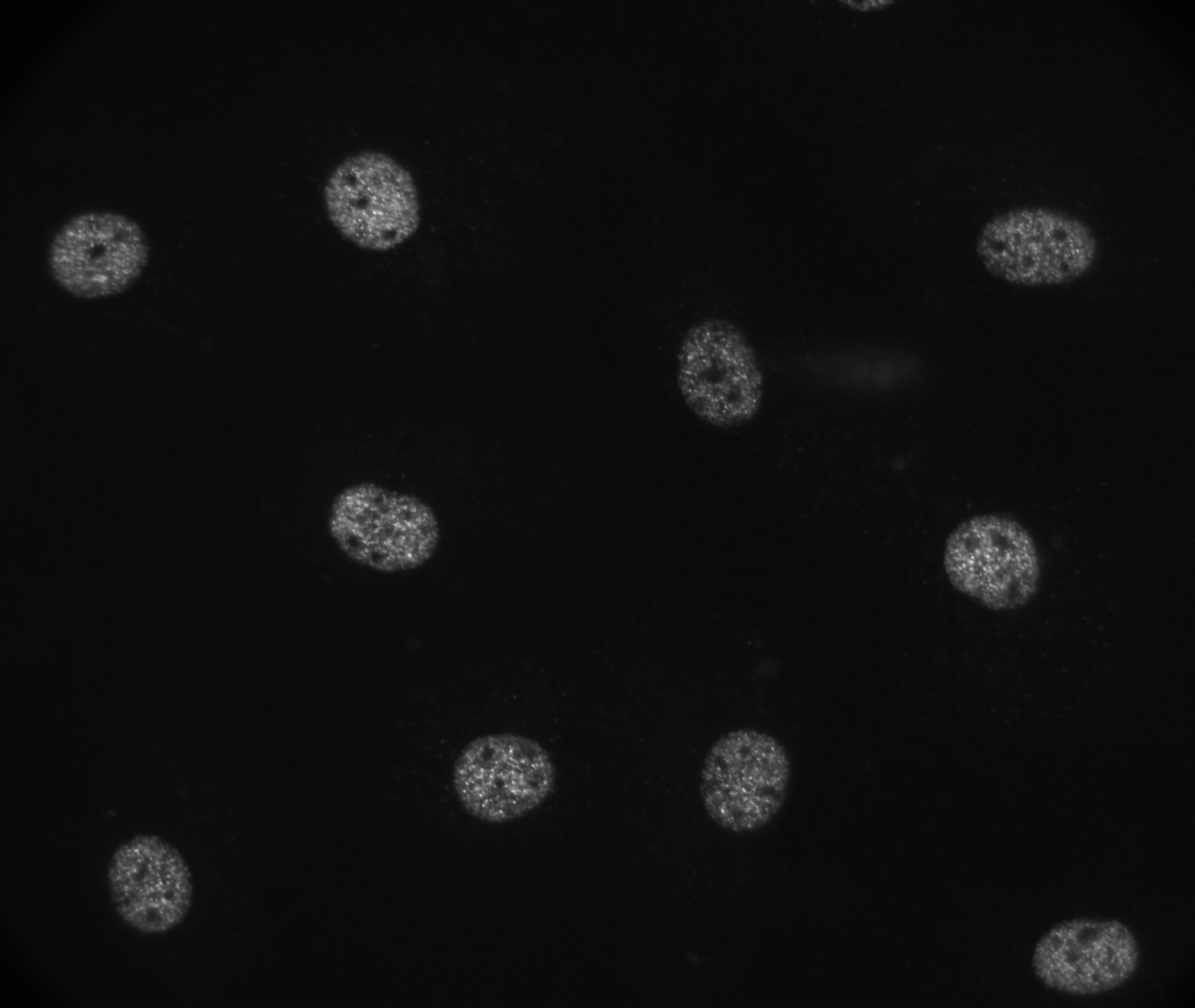

Supplement: Supplementary file 13 — Figures EV and Appendix Source Data [file 44318_2024_348_MOESM13_ESM.zip › SD figure EV and Appendix/EV3F/H3K9me2/560.jpg]

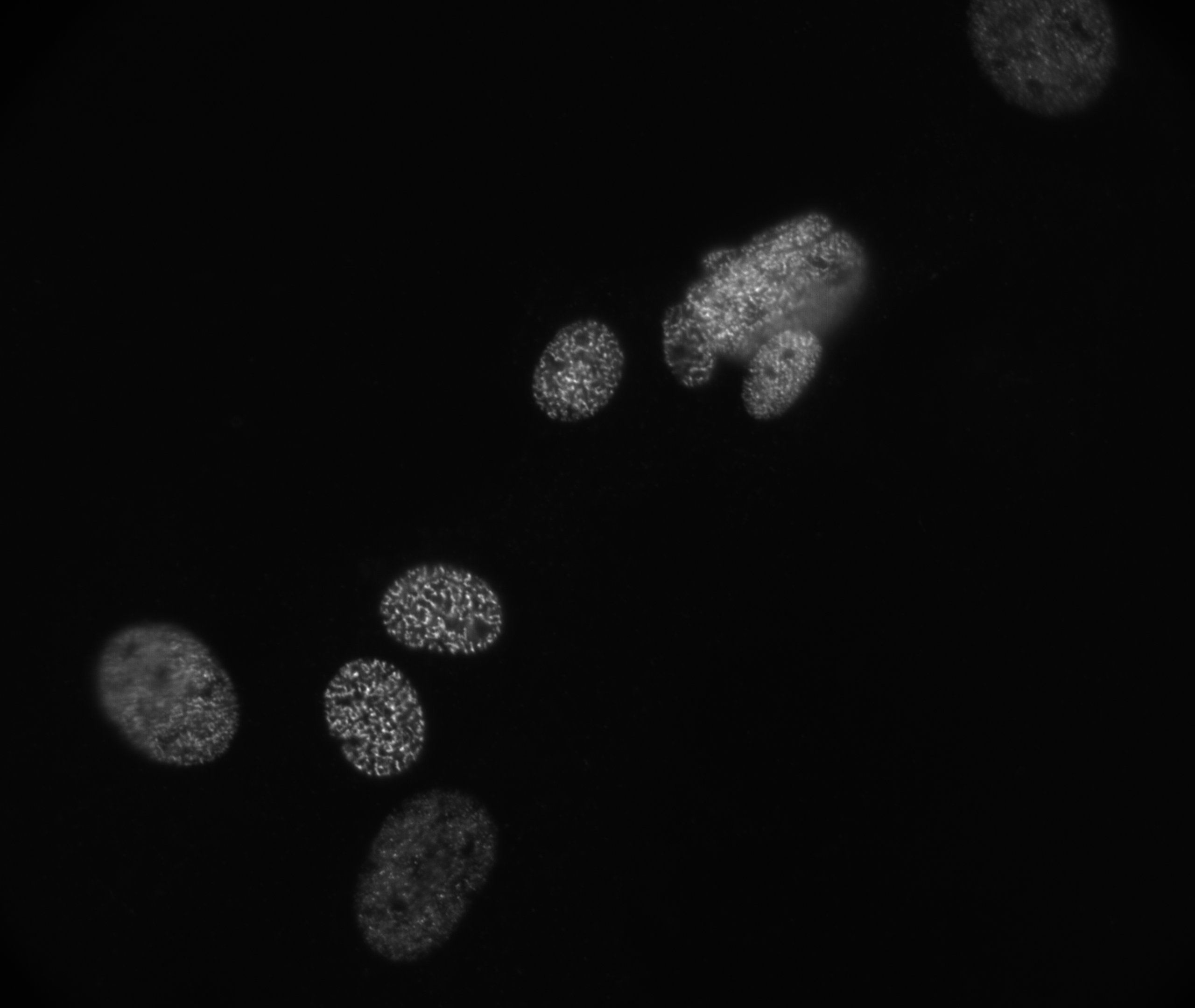

Supplement: Supplementary file 13 — Figures EV and Appendix Source Data [file 44318_2024_348_MOESM13_ESM.zip › SD figure EV and Appendix/EV3F/H3K9me3/560-2.jpg]

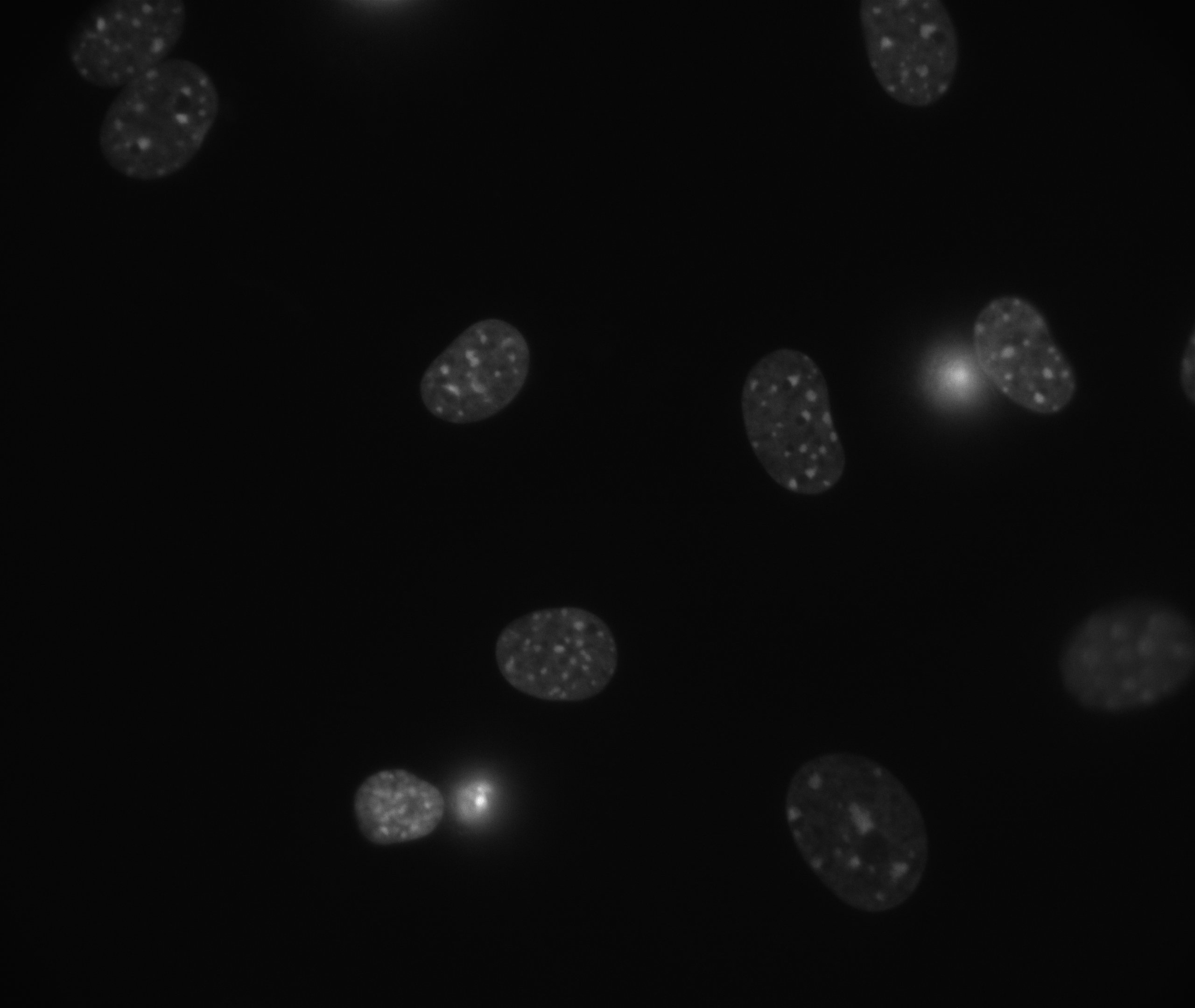

Supplement: Supplementary file 13 — Figures EV and Appendix Source Data [file 44318_2024_348_MOESM13_ESM.zip › SD figure EV and Appendix/EV3F/H3K9me3/360.jpg]

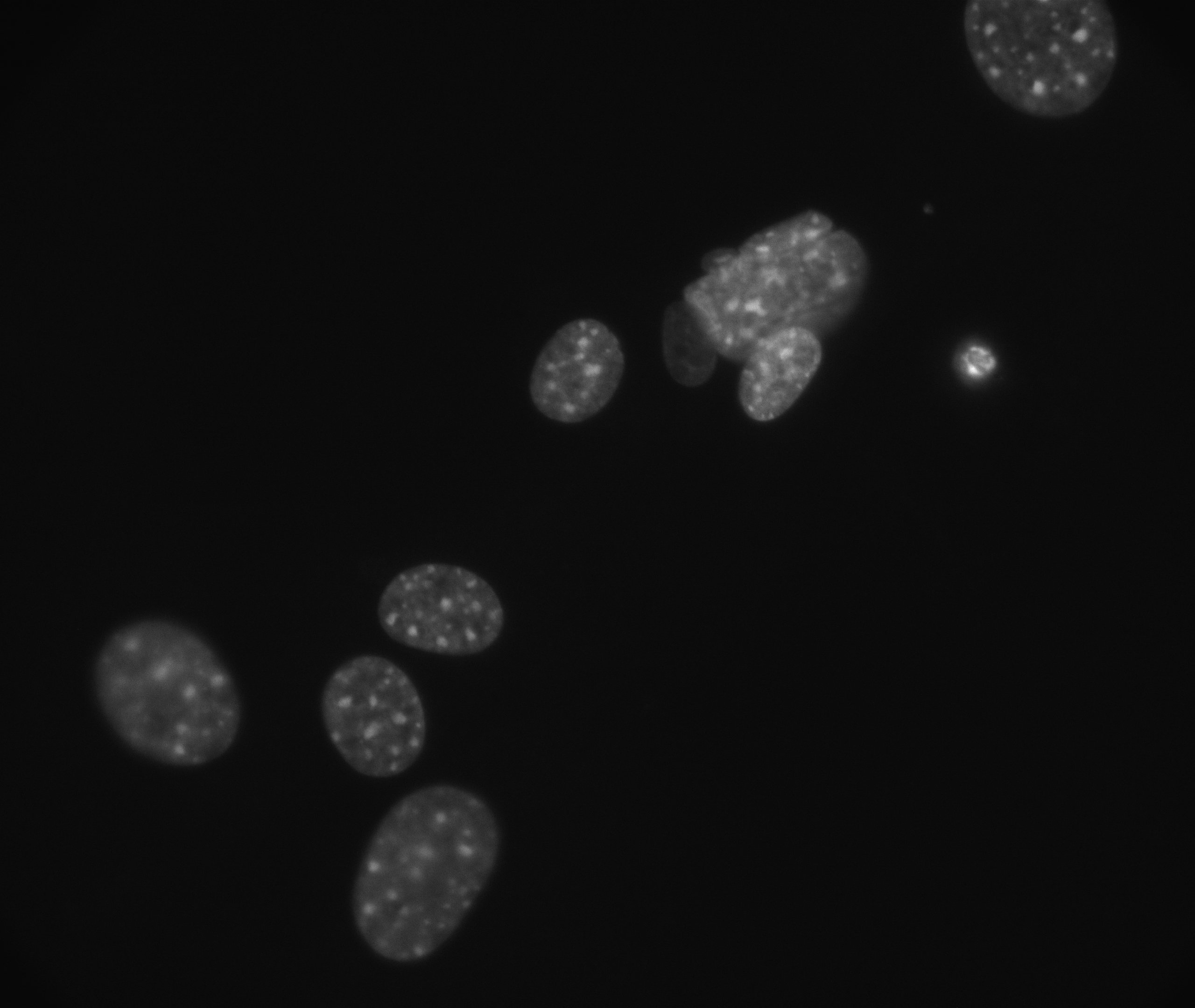

Supplement: Supplementary file 13 — Figures EV and Appendix Source Data [file 44318_2024_348_MOESM13_ESM.zip › SD figure EV and Appendix/EV3F/H3K9me3/360-2.jpg]

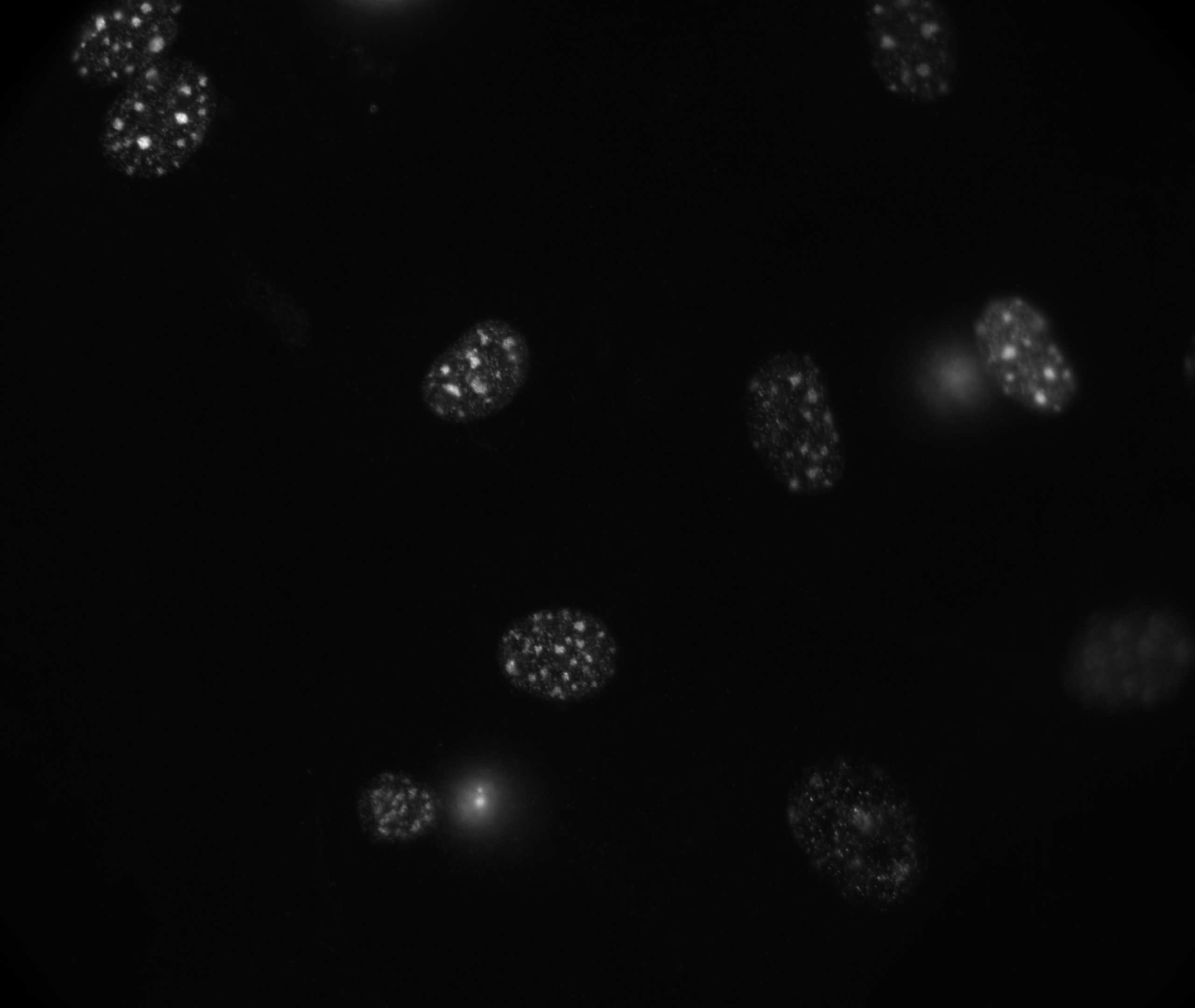

Supplement: Supplementary file 13 — Figures EV and Appendix Source Data [file 44318_2024_348_MOESM13_ESM.zip › SD figure EV and Appendix/EV3F/H3K9me3/480.jpg]

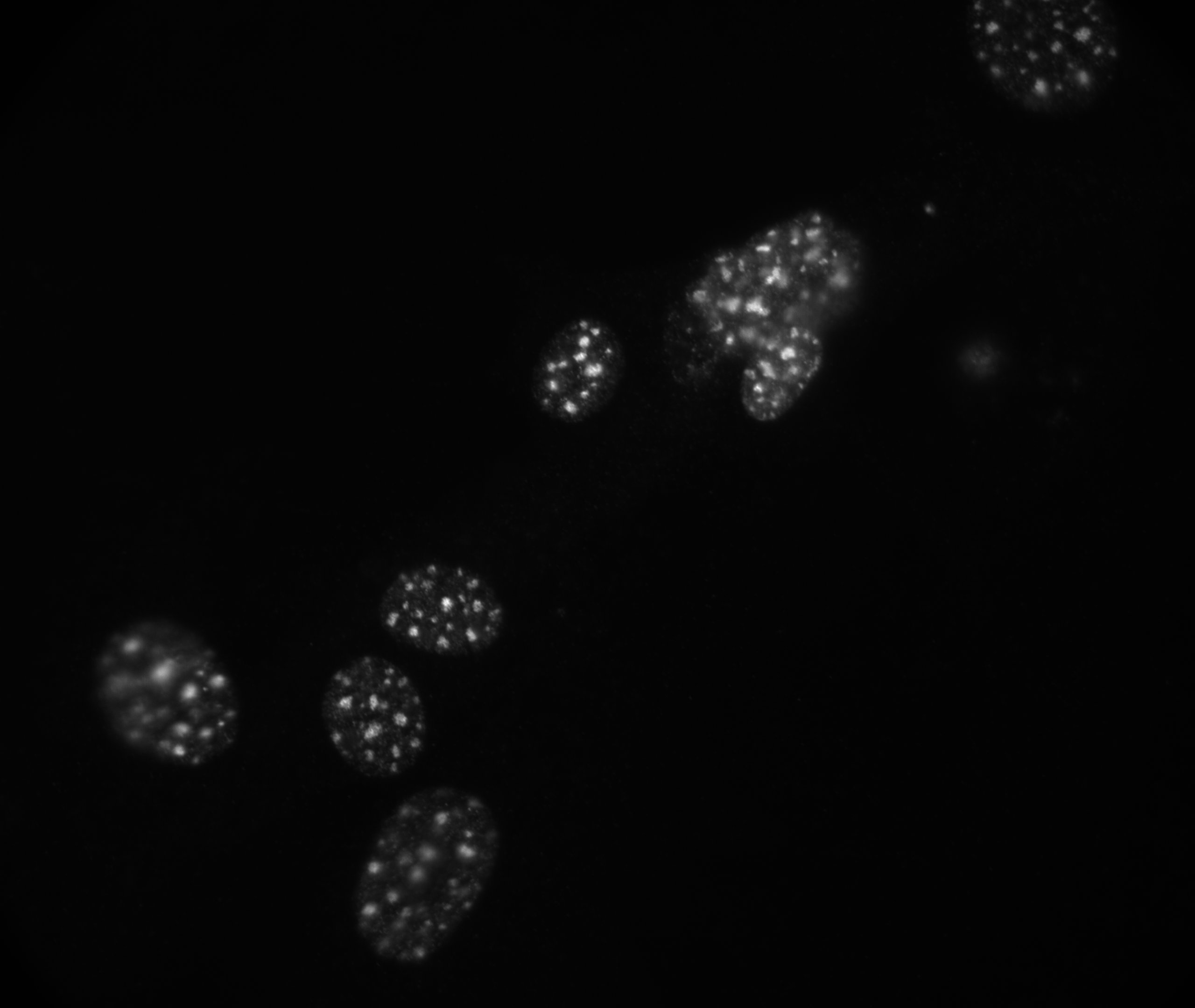

Supplement: Supplementary file 13 — Figures EV and Appendix Source Data [file 44318_2024_348_MOESM13_ESM.zip › SD figure EV and Appendix/EV3F/H3K9me3/480-2.jpg]

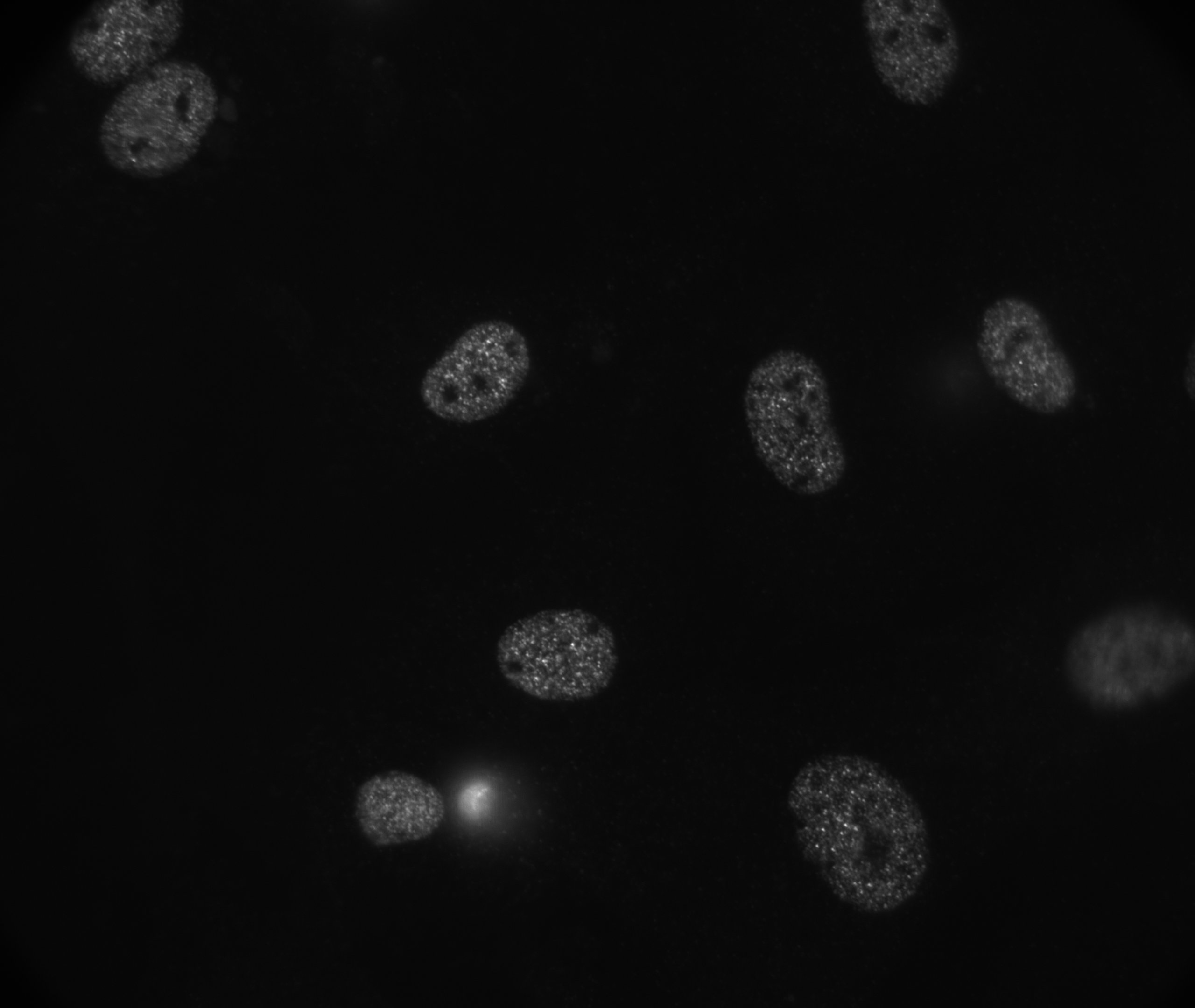

Supplement: Supplementary file 13 — Figures EV and Appendix Source Data [file 44318_2024_348_MOESM13_ESM.zip › SD figure EV and Appendix/EV3F/H3K9me3/560.jpg]

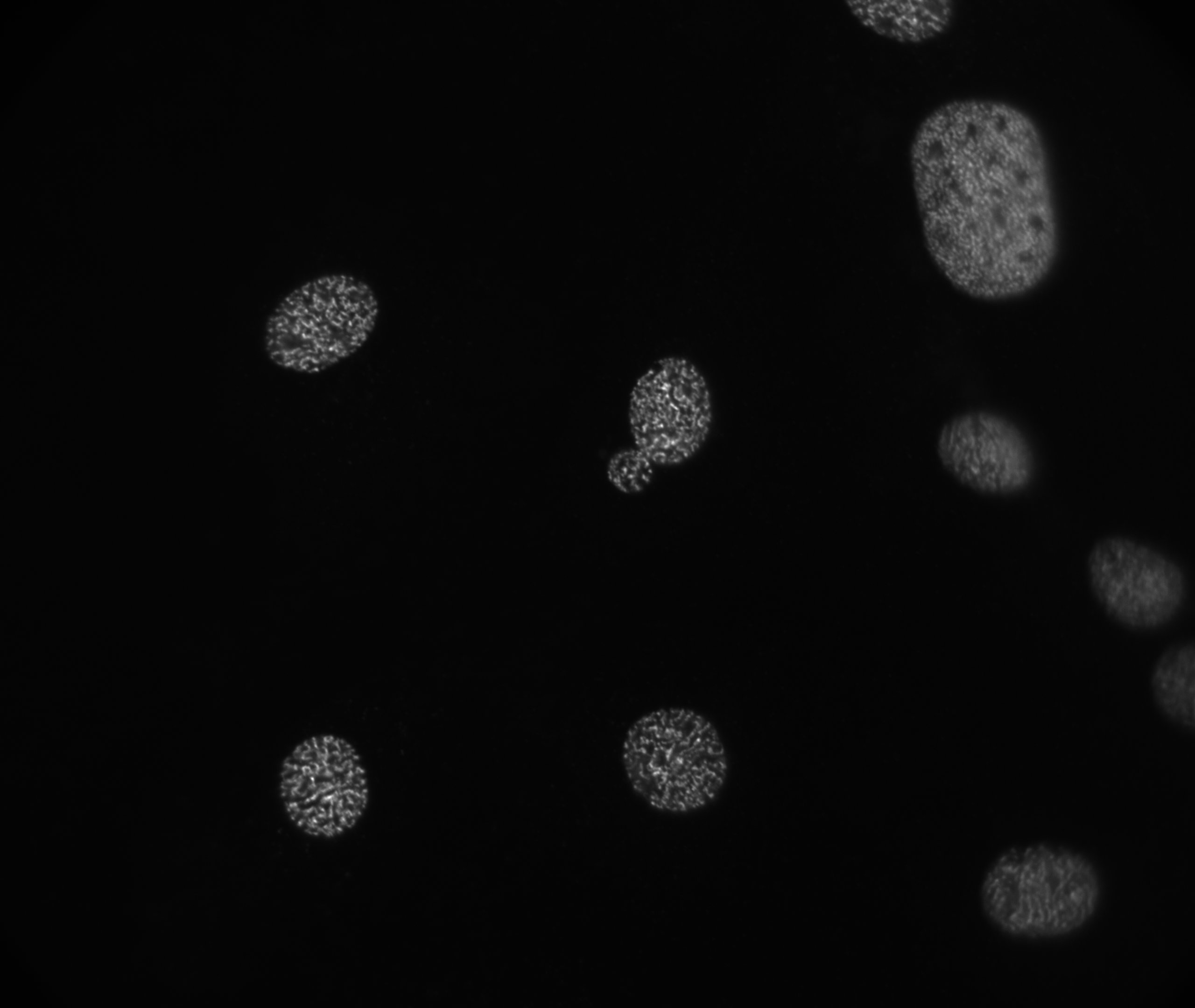

Supplement: Supplementary file 13 — Figures EV and Appendix Source Data [file 44318_2024_348_MOESM13_ESM.zip › SD figure EV and Appendix/EV3F/H4K20me3/560-2.jpg]

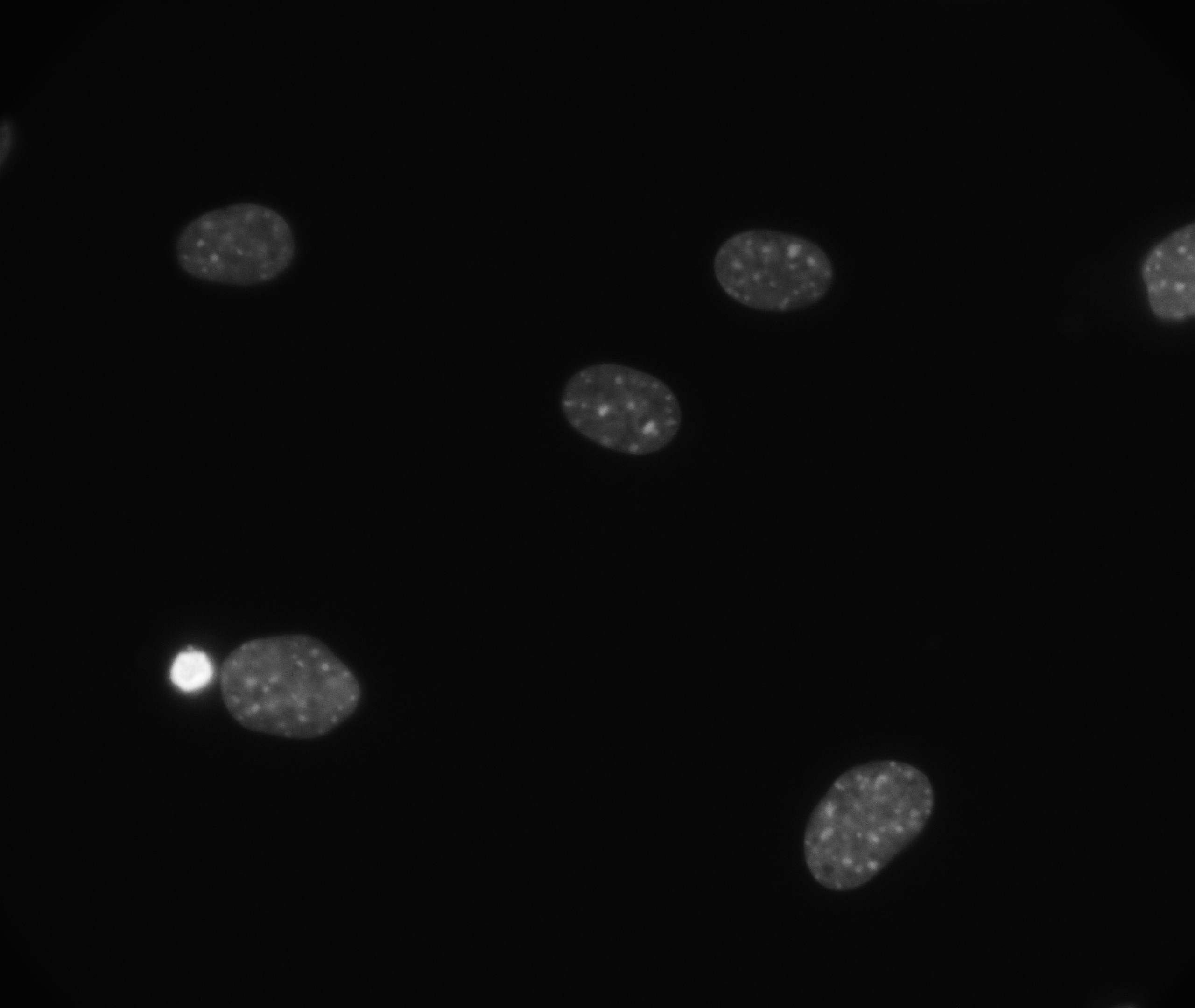

Supplement: Supplementary file 13 — Figures EV and Appendix Source Data [file 44318_2024_348_MOESM13_ESM.zip › SD figure EV and Appendix/EV3F/H4K20me3/360.jpg]

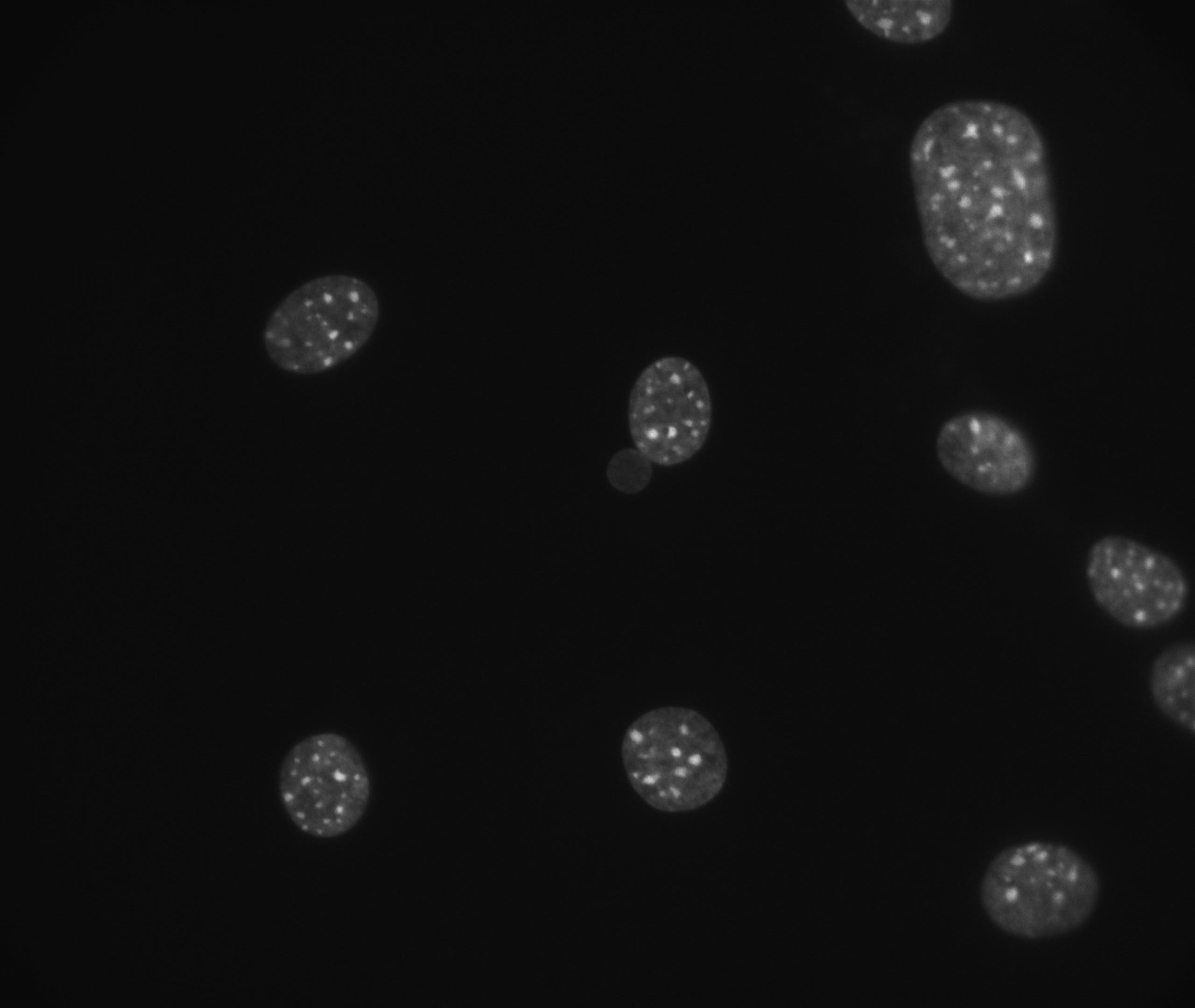

Supplement: Supplementary file 13 — Figures EV and Appendix Source Data [file 44318_2024_348_MOESM13_ESM.zip › SD figure EV and Appendix/EV3F/H4K20me3/360-2.jpg]

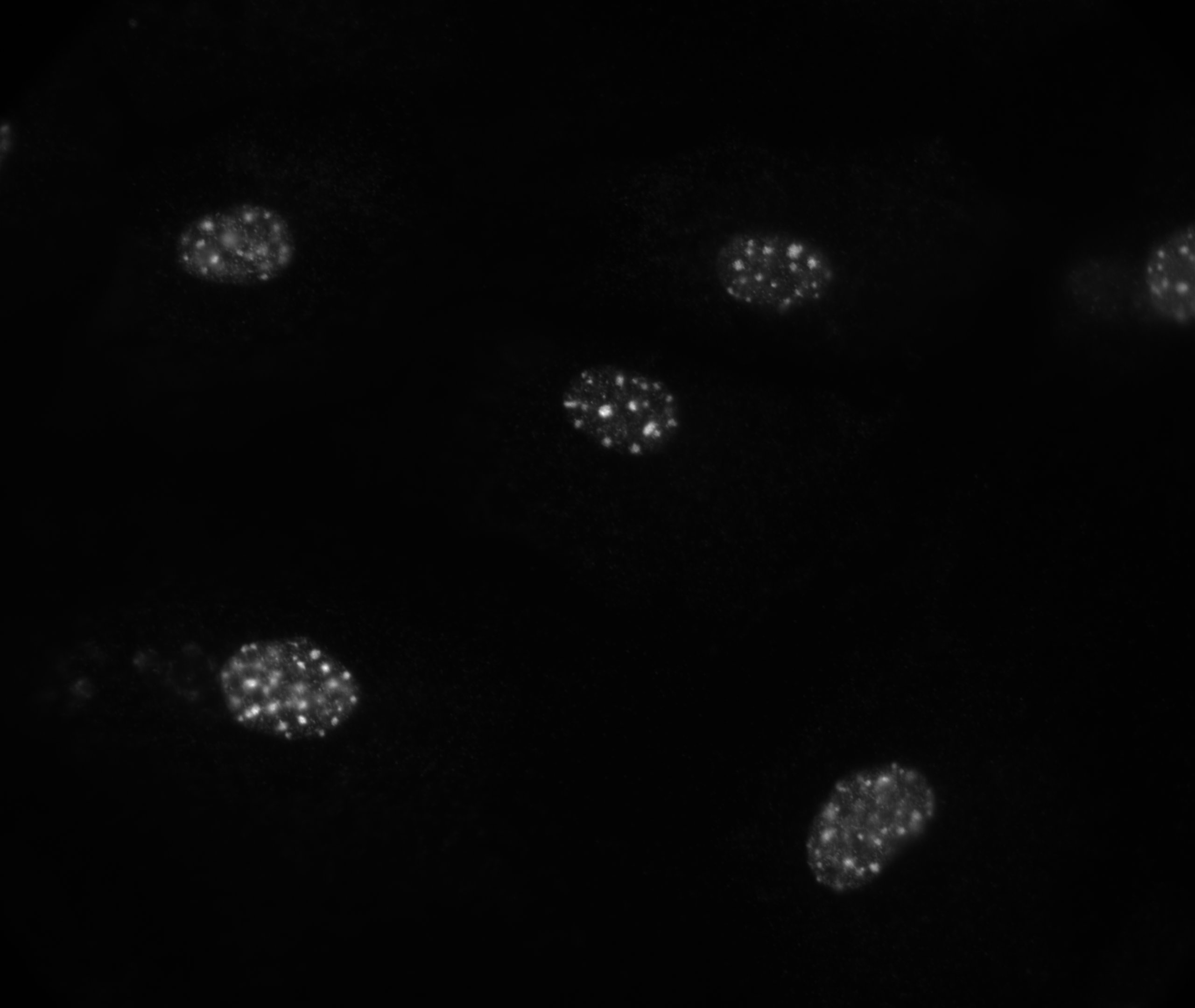

Supplement: Supplementary file 13 — Figures EV and Appendix Source Data [file 44318_2024_348_MOESM13_ESM.zip › SD figure EV and Appendix/EV3F/H4K20me3/480.jpg]

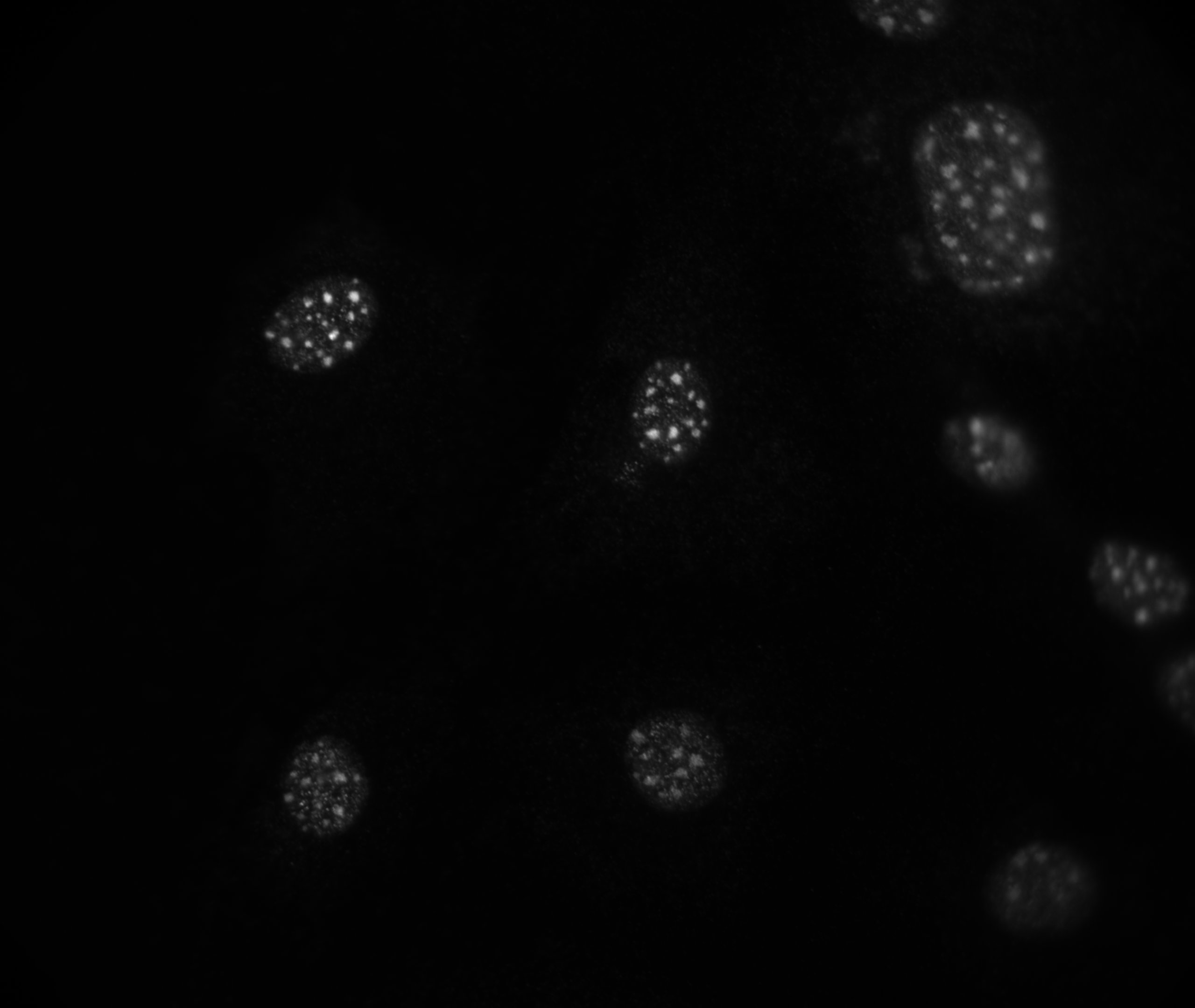

Supplement: Supplementary file 13 — Figures EV and Appendix Source Data [file 44318_2024_348_MOESM13_ESM.zip › SD figure EV and Appendix/EV3F/H4K20me3/480-2.jpg]

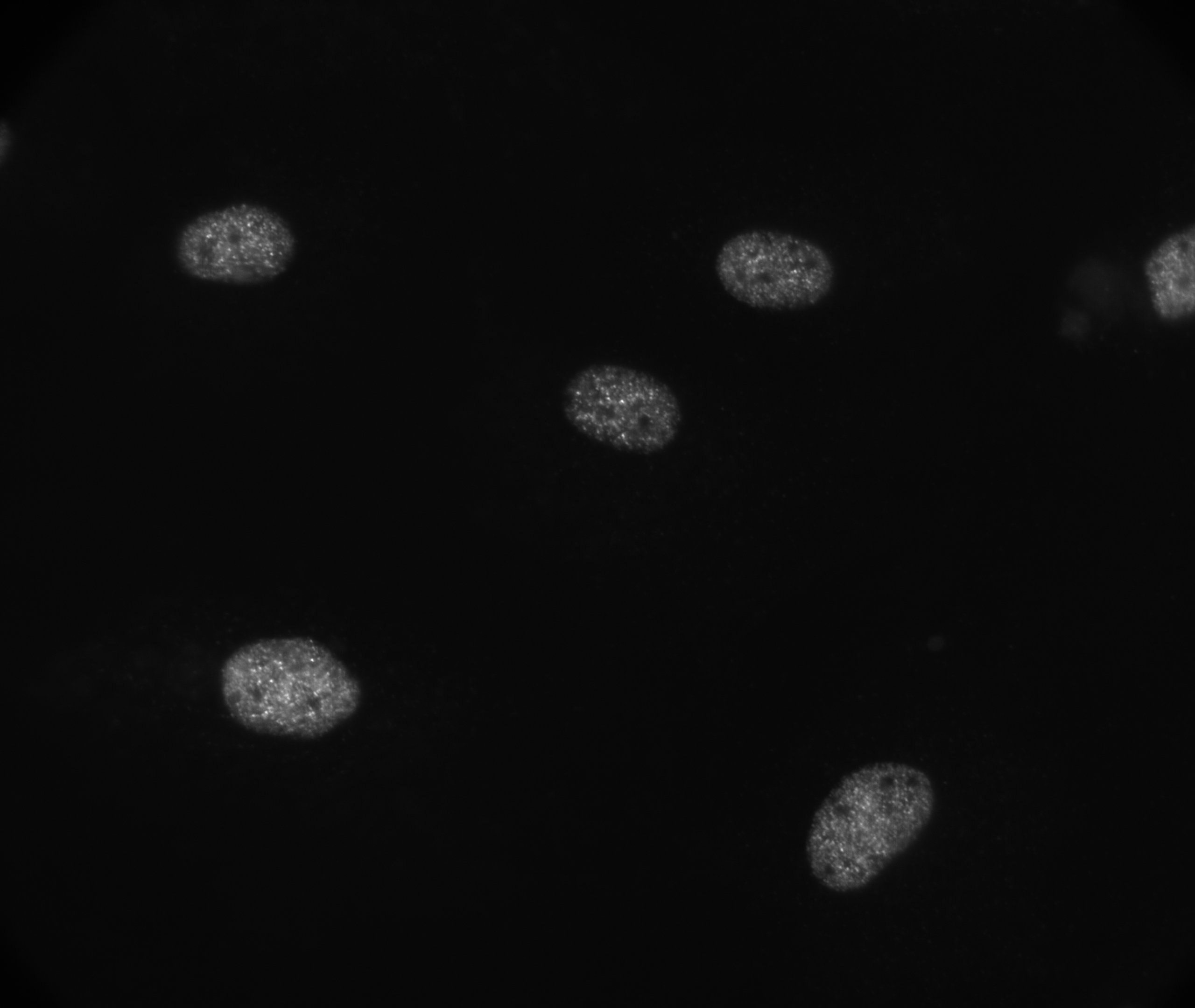

Supplement: Supplementary file 13 — Figures EV and Appendix Source Data [file 44318_2024_348_MOESM13_ESM.zip › SD figure EV and Appendix/EV3F/H4K20me3/560.jpg]

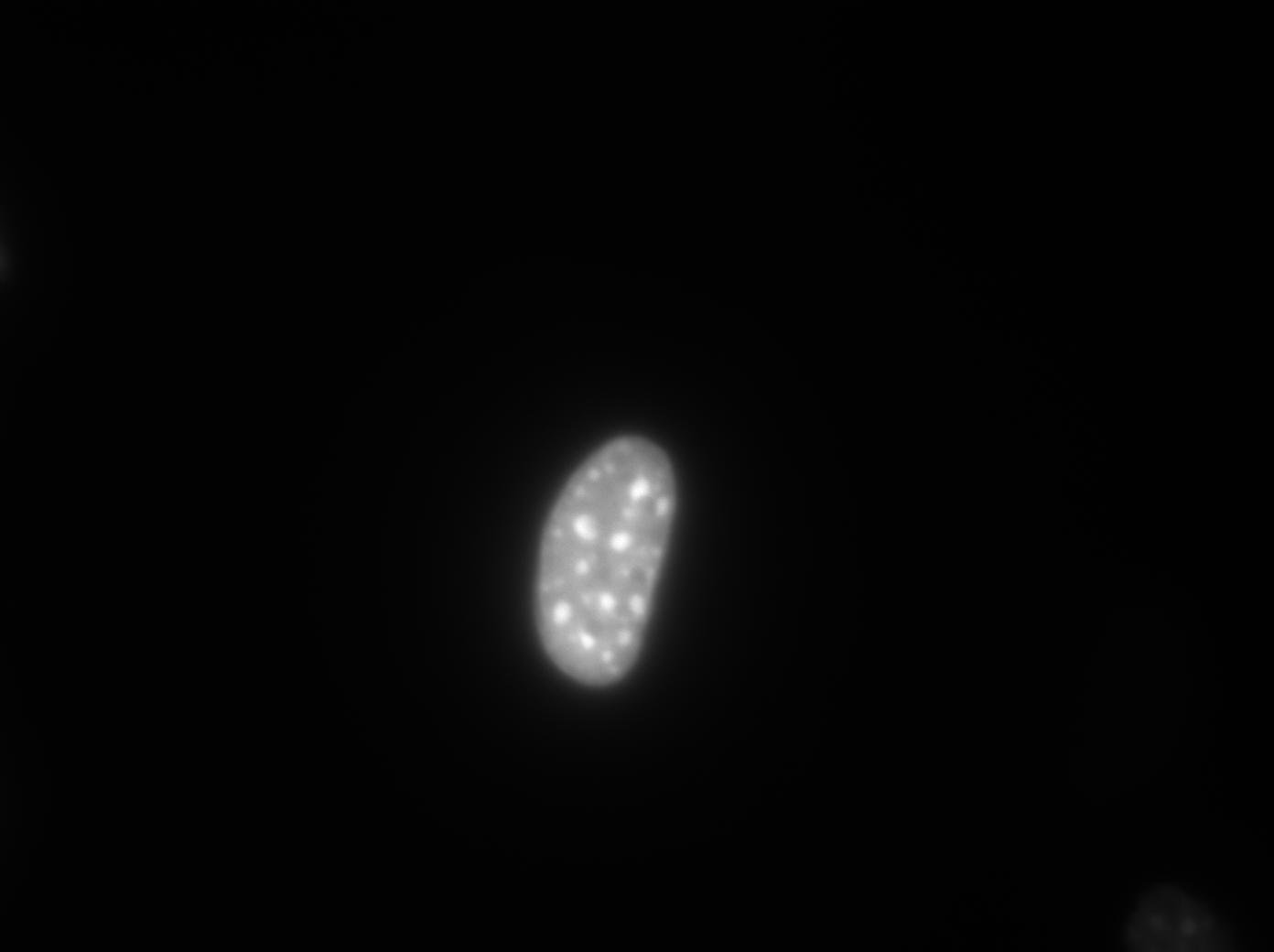

Supplement: Supplementary file 13 — Figures EV and Appendix Source Data [file 44318_2024_348_MOESM13_ESM.zip › SD figure EV and Appendix/Appendix Figure 1H/Phf8 Ab/Acquired-4.jpg]

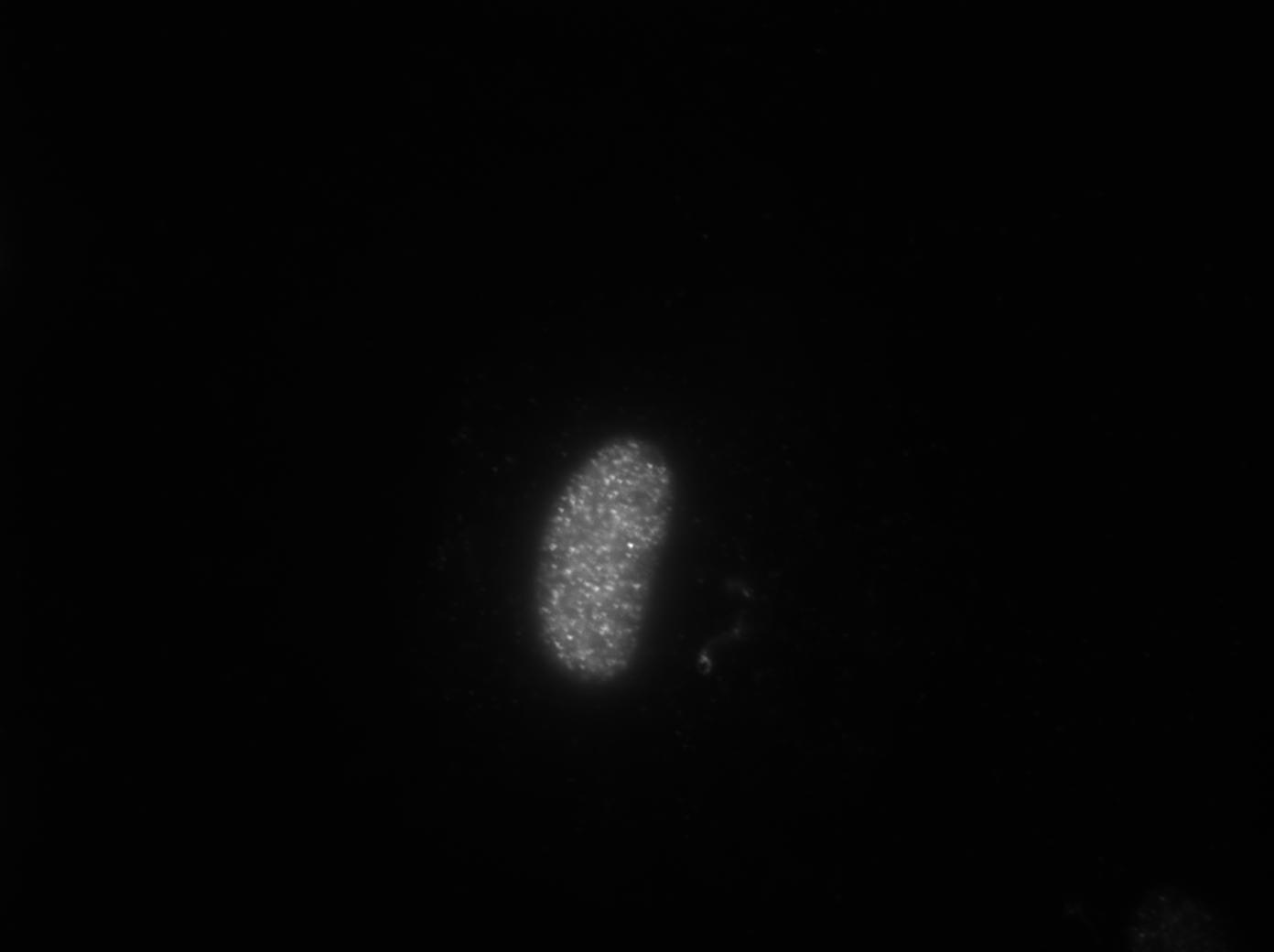

Supplement: Supplementary file 13 — Figures EV and Appendix Source Data [file 44318_2024_348_MOESM13_ESM.zip › SD figure EV and Appendix/Appendix Figure 1H/Phf8 Ab/Acquired-5.jpg]

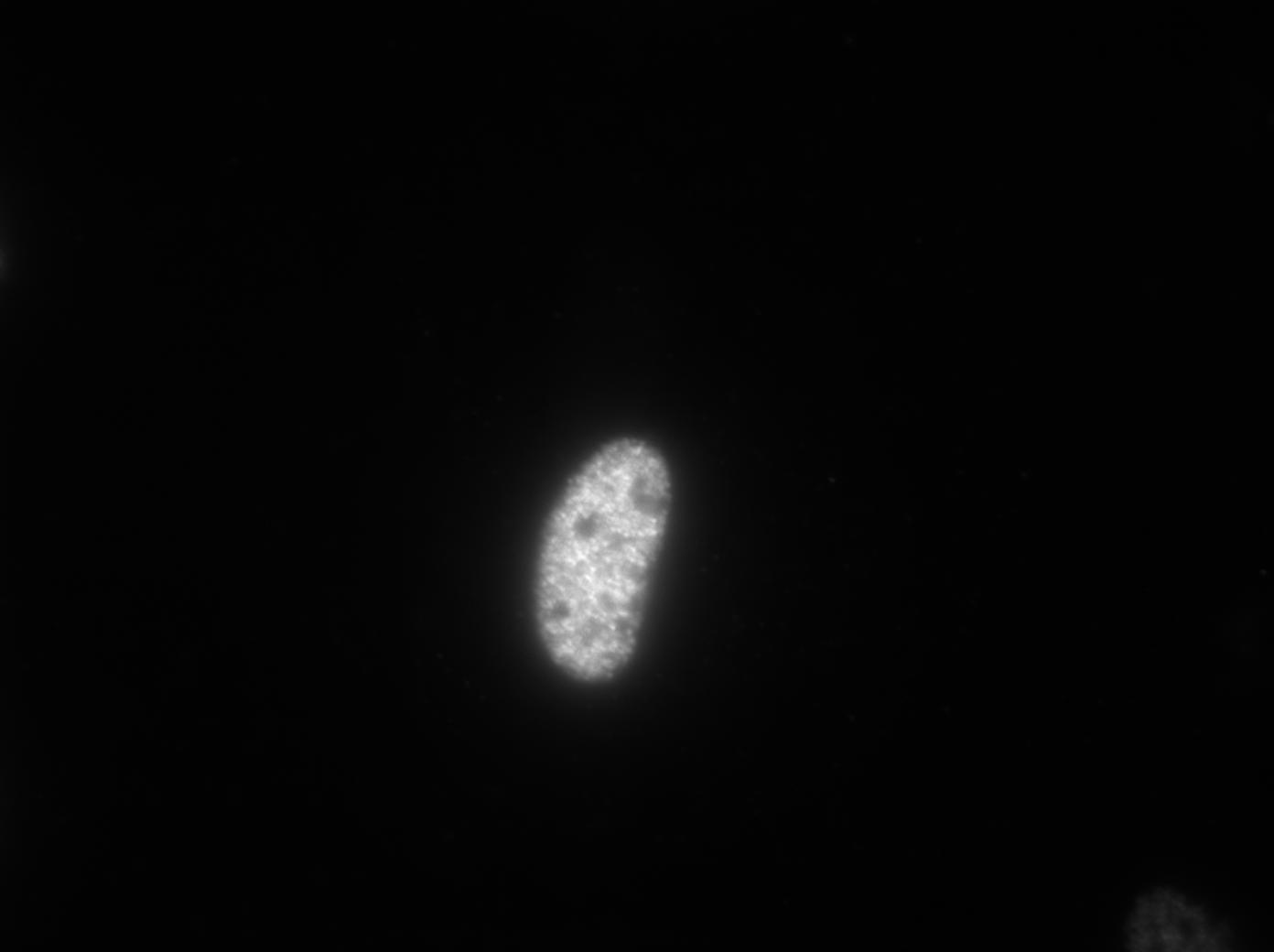

Supplement: Supplementary file 13 — Figures EV and Appendix Source Data [file 44318_2024_348_MOESM13_ESM.zip › SD figure EV and Appendix/Appendix Figure 1H/Phf8 Ab/Acquired-6.jpg]

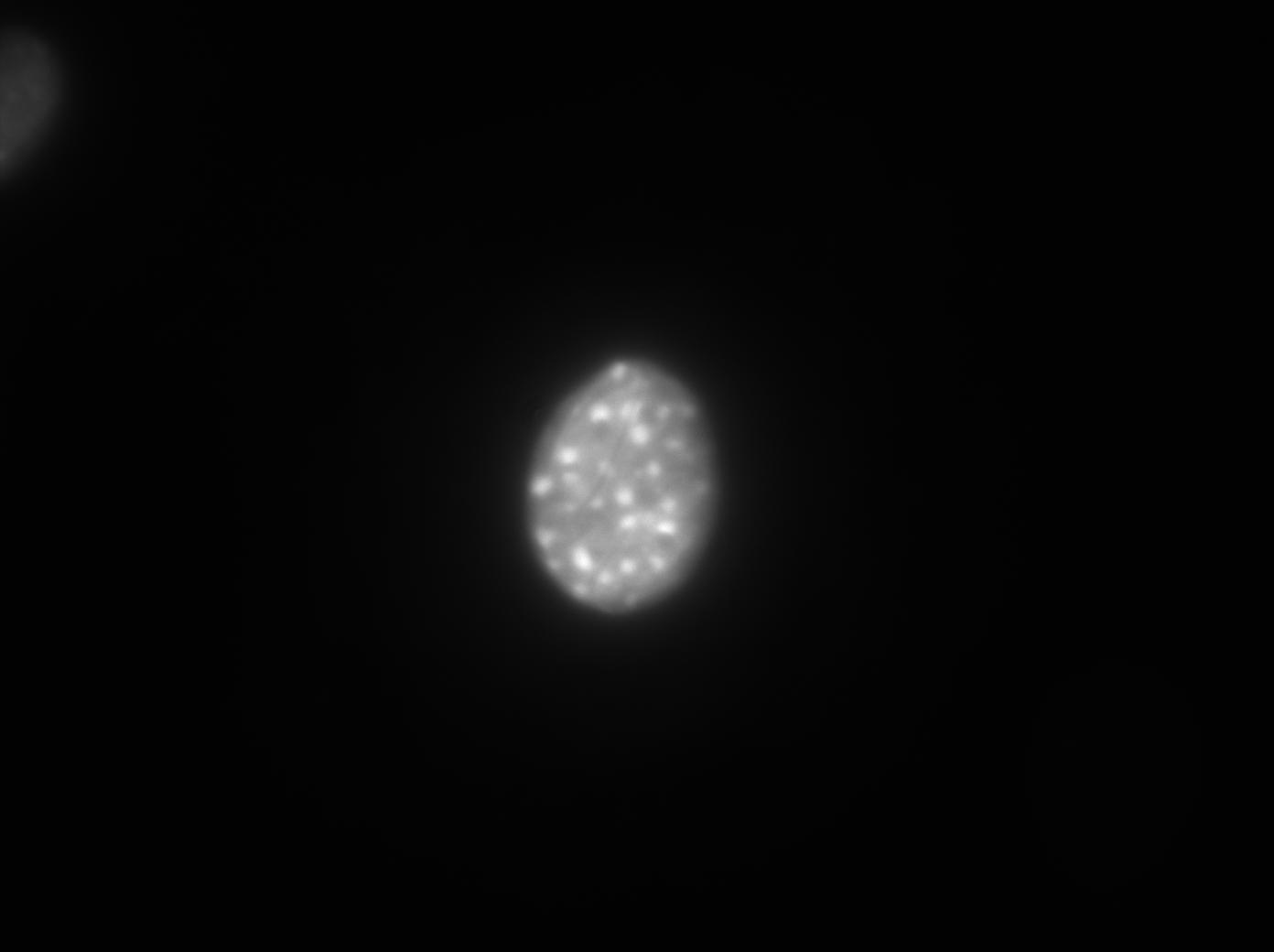

Supplement: Supplementary file 13 — Figures EV and Appendix Source Data [file 44318_2024_348_MOESM13_ESM.zip › SD figure EV and Appendix/Appendix Figure 1H/Phf8 Ab/Acquired-111.jpg]

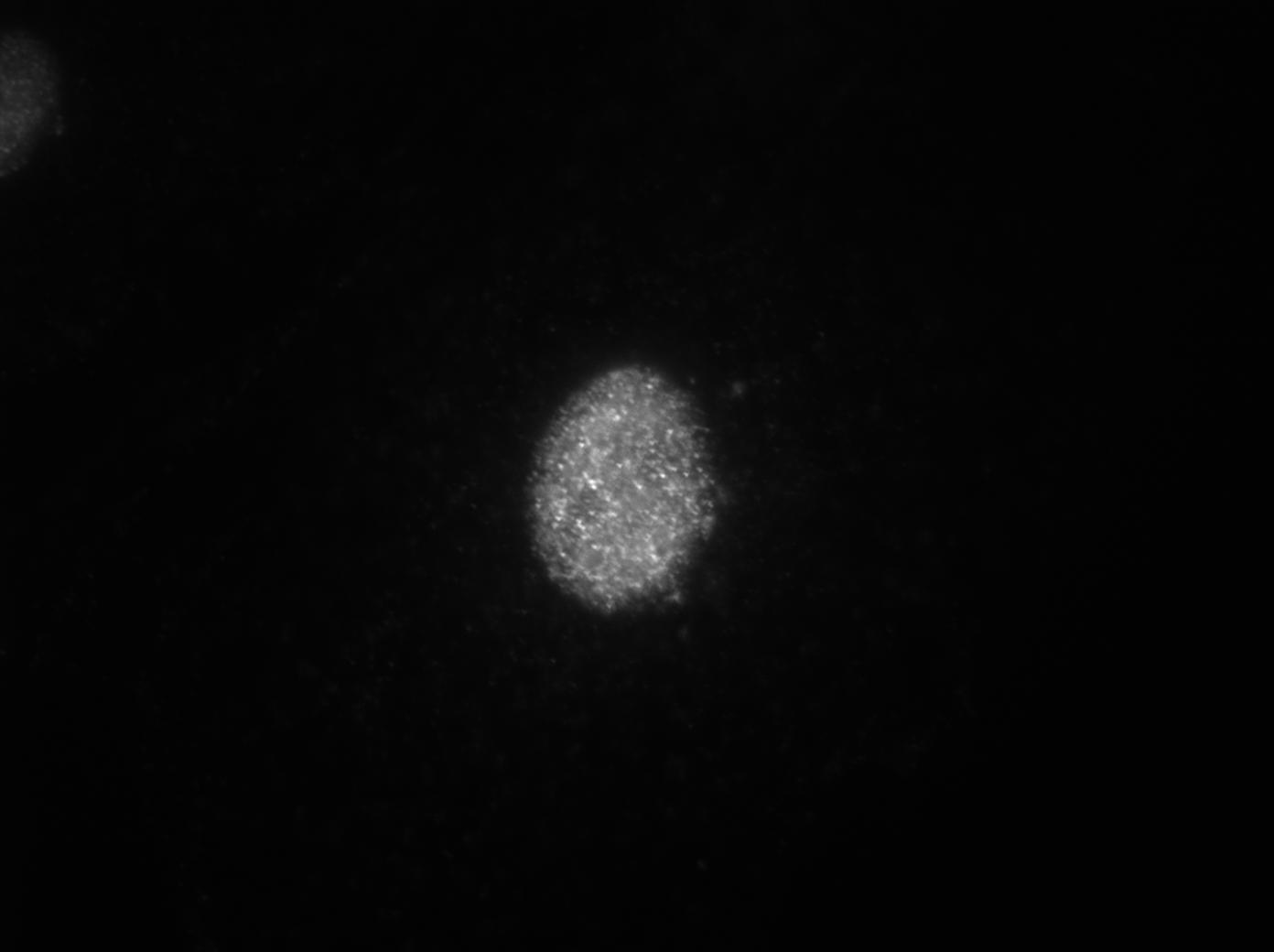

Supplement: Supplementary file 13 — Figures EV and Appendix Source Data [file 44318_2024_348_MOESM13_ESM.zip › SD figure EV and Appendix/Appendix Figure 1H/Phf8 Ab/Acquired-222.jpg]

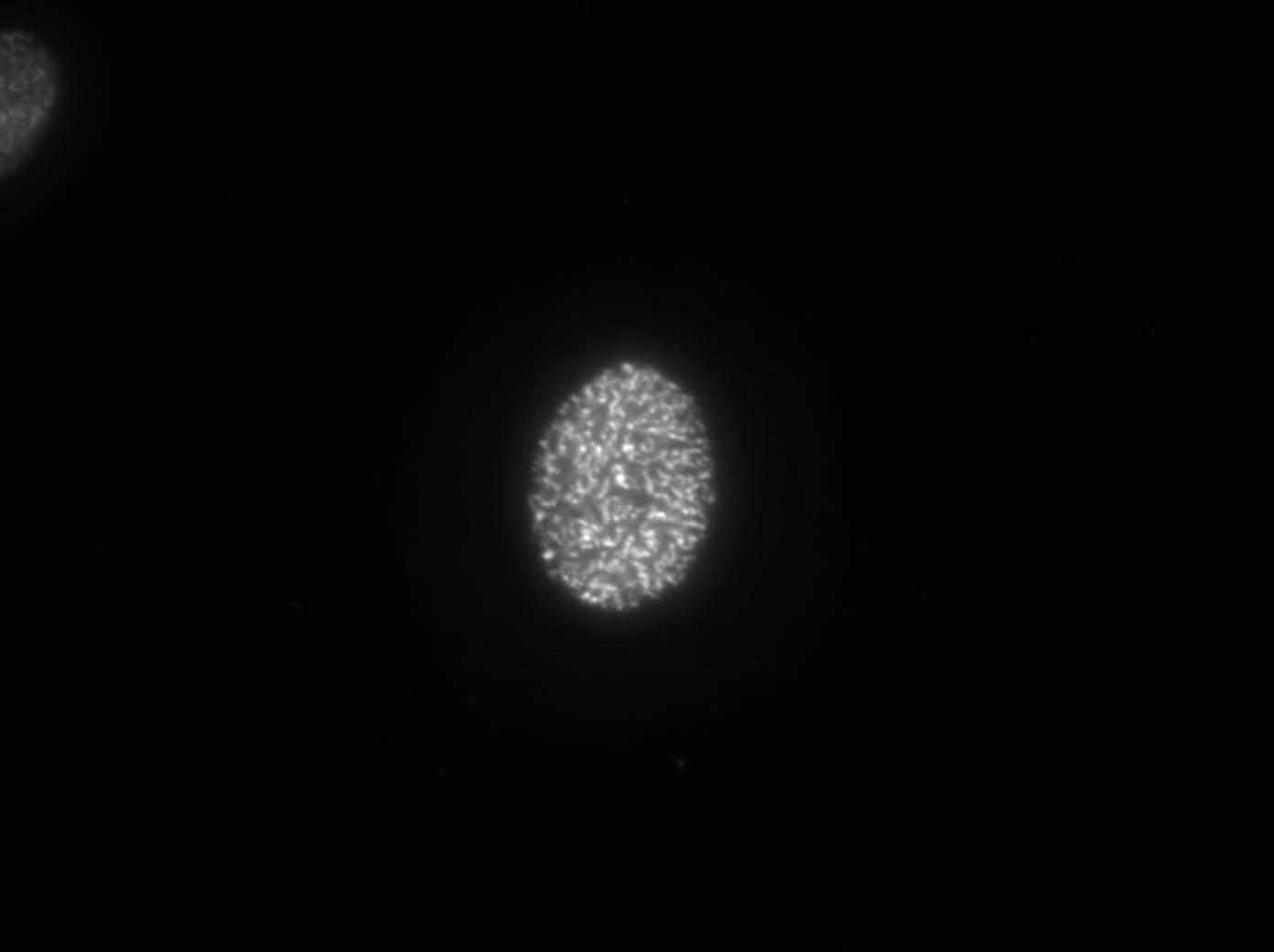

Supplement: Supplementary file 13 — Figures EV and Appendix Source Data [file 44318_2024_348_MOESM13_ESM.zip › SD figure EV and Appendix/Appendix Figure 1H/Phf8 Ab/Acquired-333.jpg]

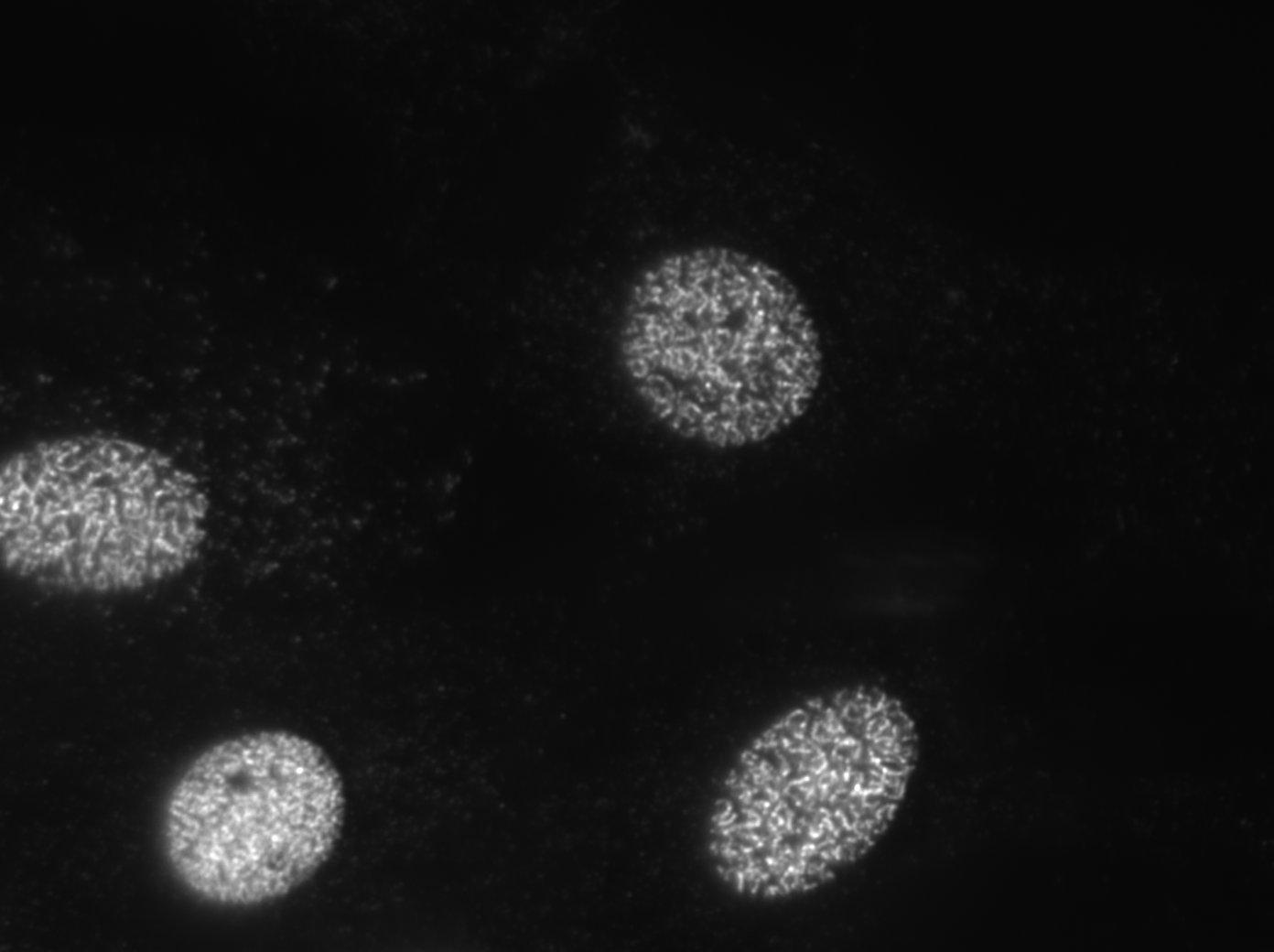

Supplement: Supplementary file 13 — Figures EV and Appendix Source Data [file 44318_2024_348_MOESM13_ESM.zip › SD figure EV and Appendix/Appendix Figure 1H/Phf8-GFP/560-22.jpg]

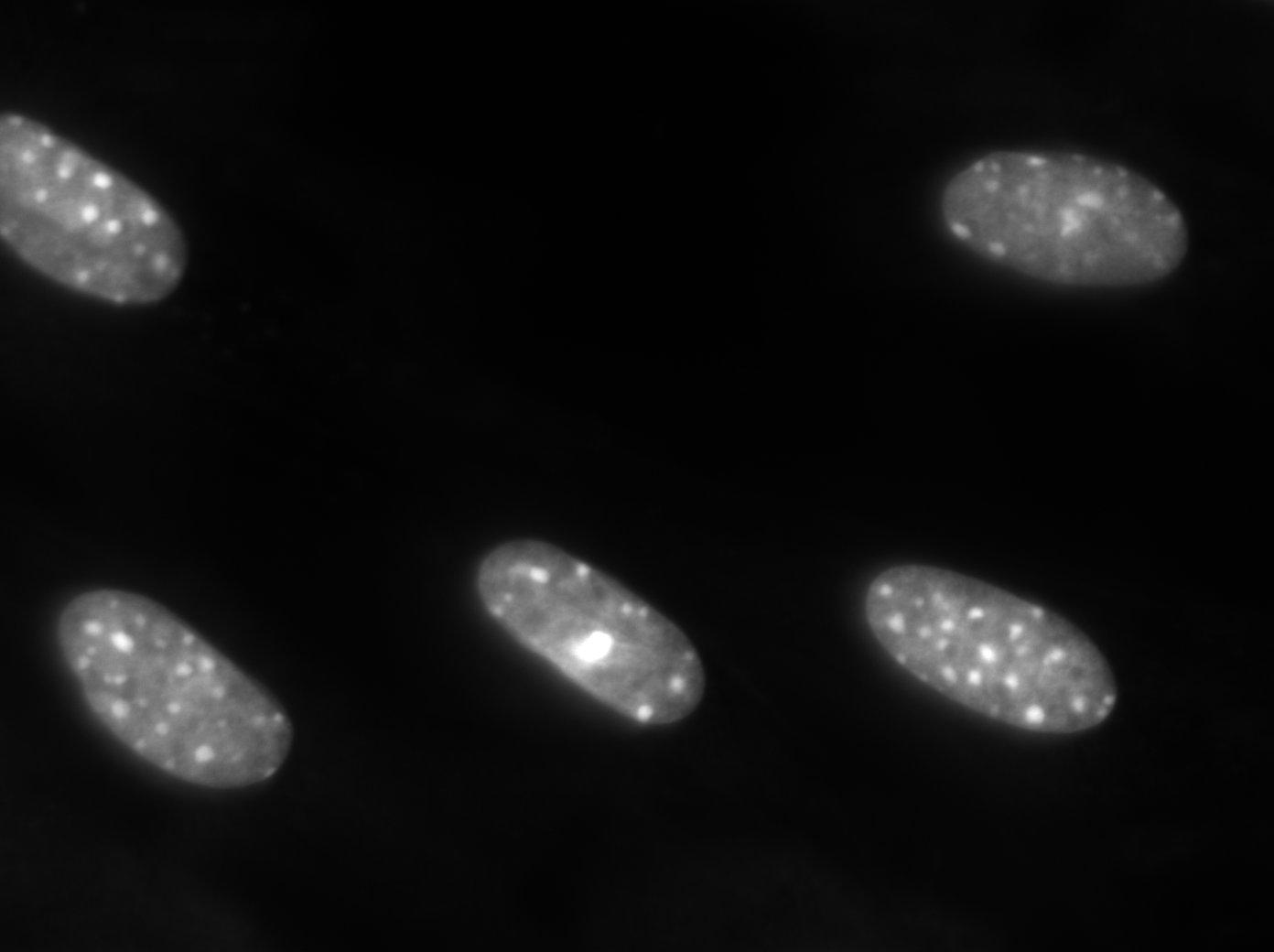

Supplement: Supplementary file 13 — Figures EV and Appendix Source Data [file 44318_2024_348_MOESM13_ESM.zip › SD figure EV and Appendix/Appendix Figure 1H/Phf8-GFP/360-11.jpg]

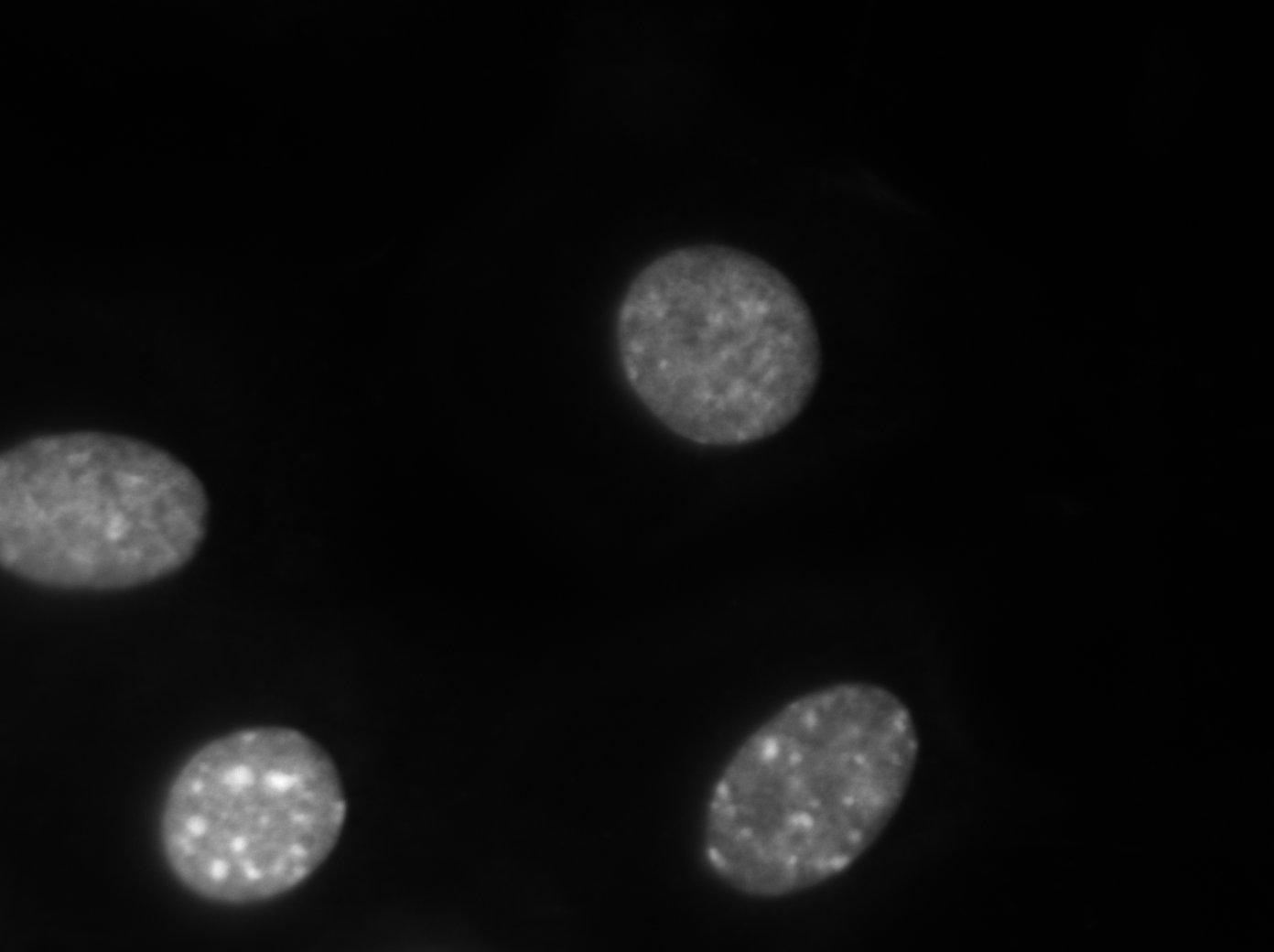

Supplement: Supplementary file 13 — Figures EV and Appendix Source Data [file 44318_2024_348_MOESM13_ESM.zip › SD figure EV and Appendix/Appendix Figure 1H/Phf8-GFP/360-22.jpg]

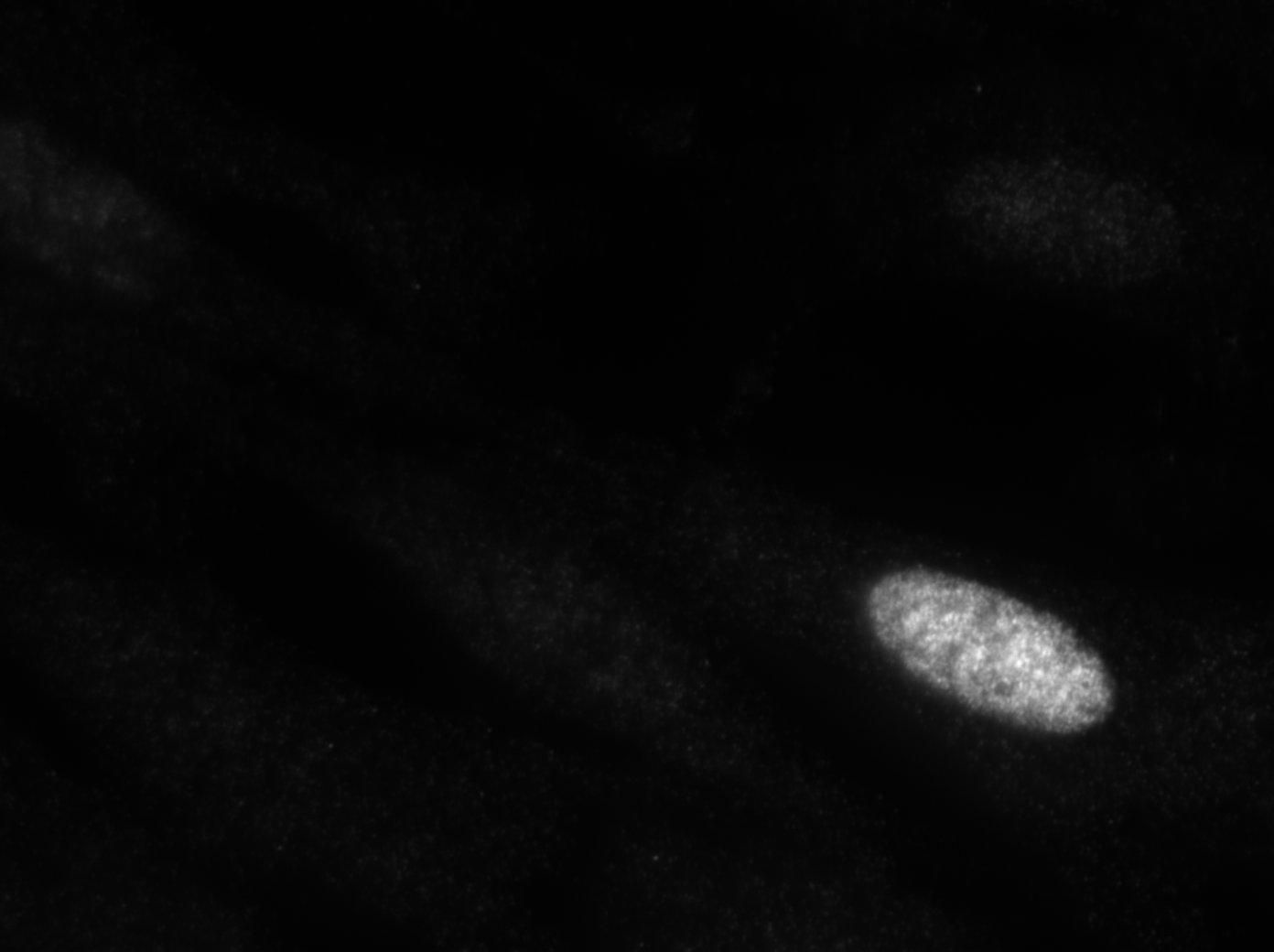

Supplement: Supplementary file 13 — Figures EV and Appendix Source Data [file 44318_2024_348_MOESM13_ESM.zip › SD figure EV and Appendix/Appendix Figure 1H/Phf8-GFP/480-11.jpg]

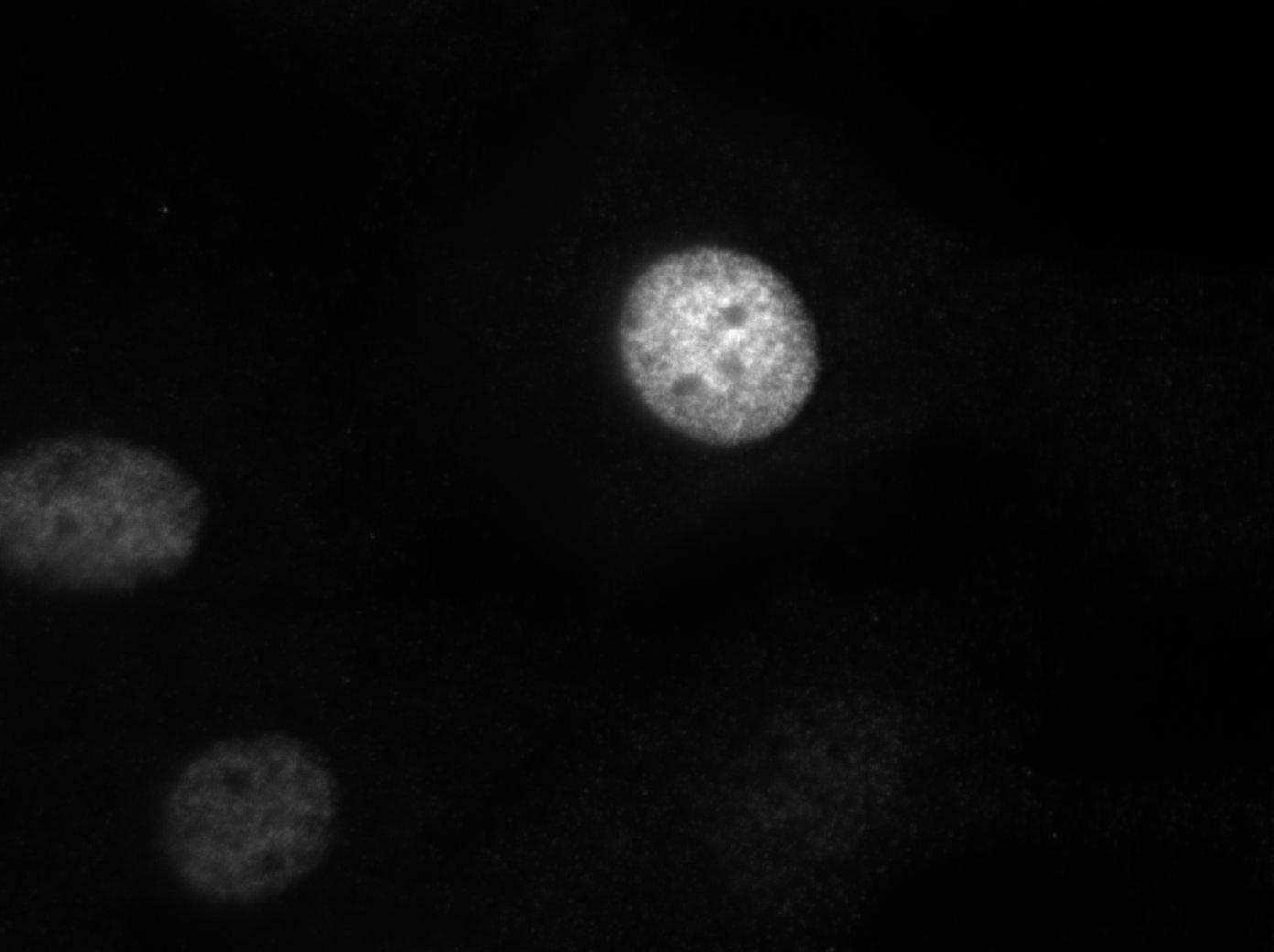

Supplement: Supplementary file 13 — Figures EV and Appendix Source Data [file 44318_2024_348_MOESM13_ESM.zip › SD figure EV and Appendix/Appendix Figure 1H/Phf8-GFP/480-22.jpg]

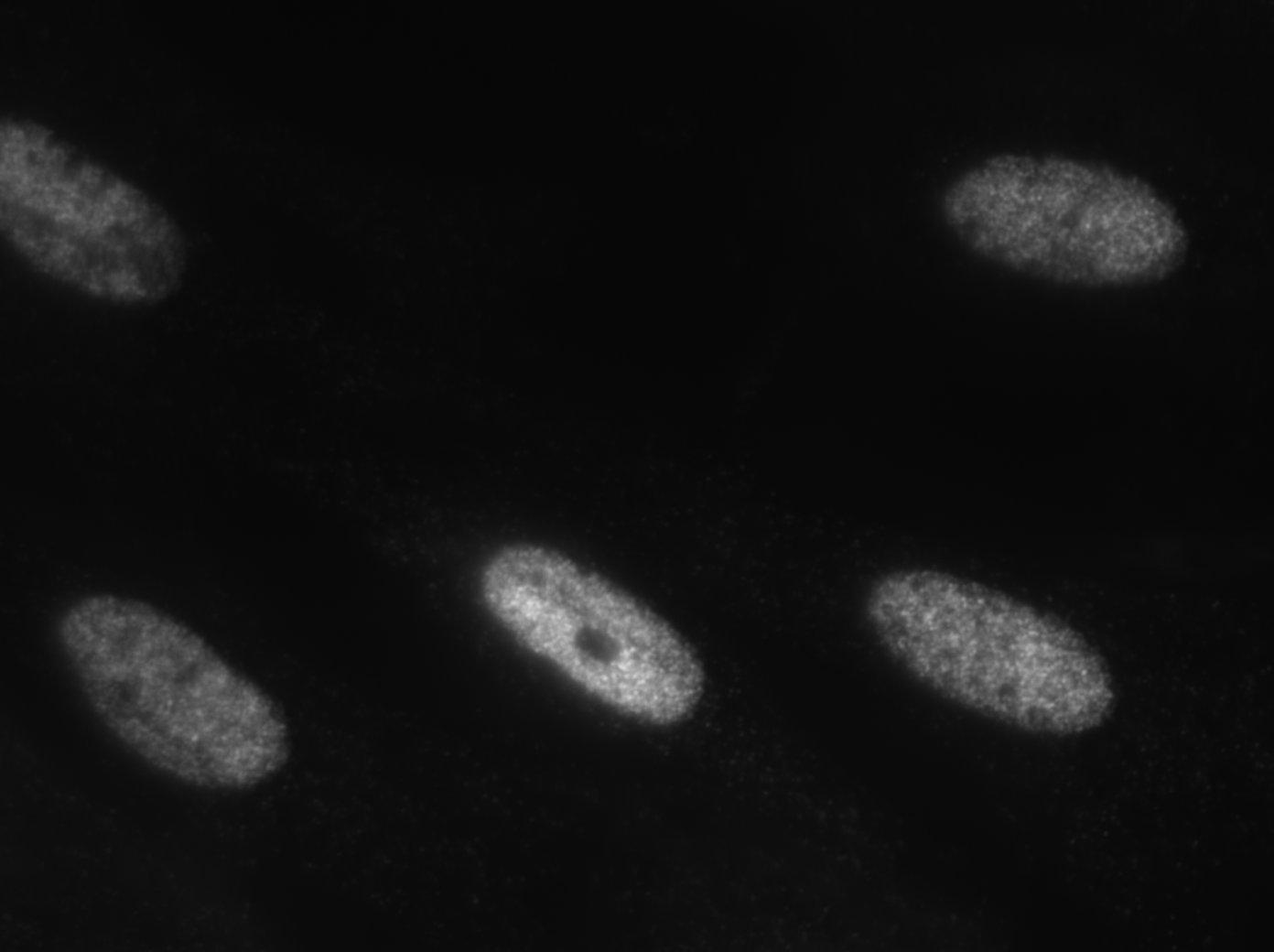

Supplement: Supplementary file 13 — Figures EV and Appendix Source Data [file 44318_2024_348_MOESM13_ESM.zip › SD figure EV and Appendix/Appendix Figure 1H/Phf8-GFP/560-11.jpg]

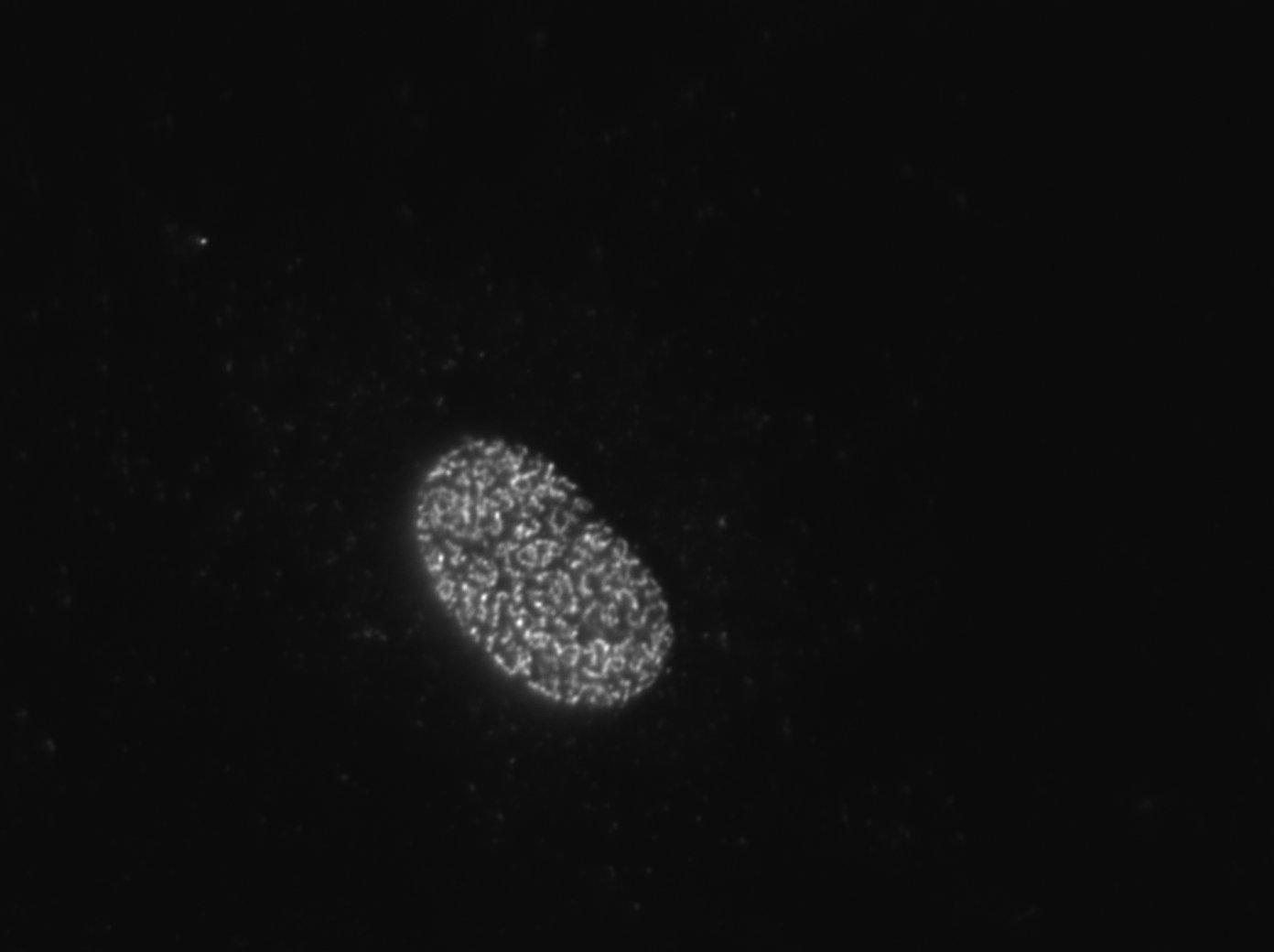

Supplement: Supplementary file 13 — Figures EV and Appendix Source Data [file 44318_2024_348_MOESM13_ESM.zip › SD figure EV and Appendix/Appendix Figure 1G/Baz1b/560-3.jpg]

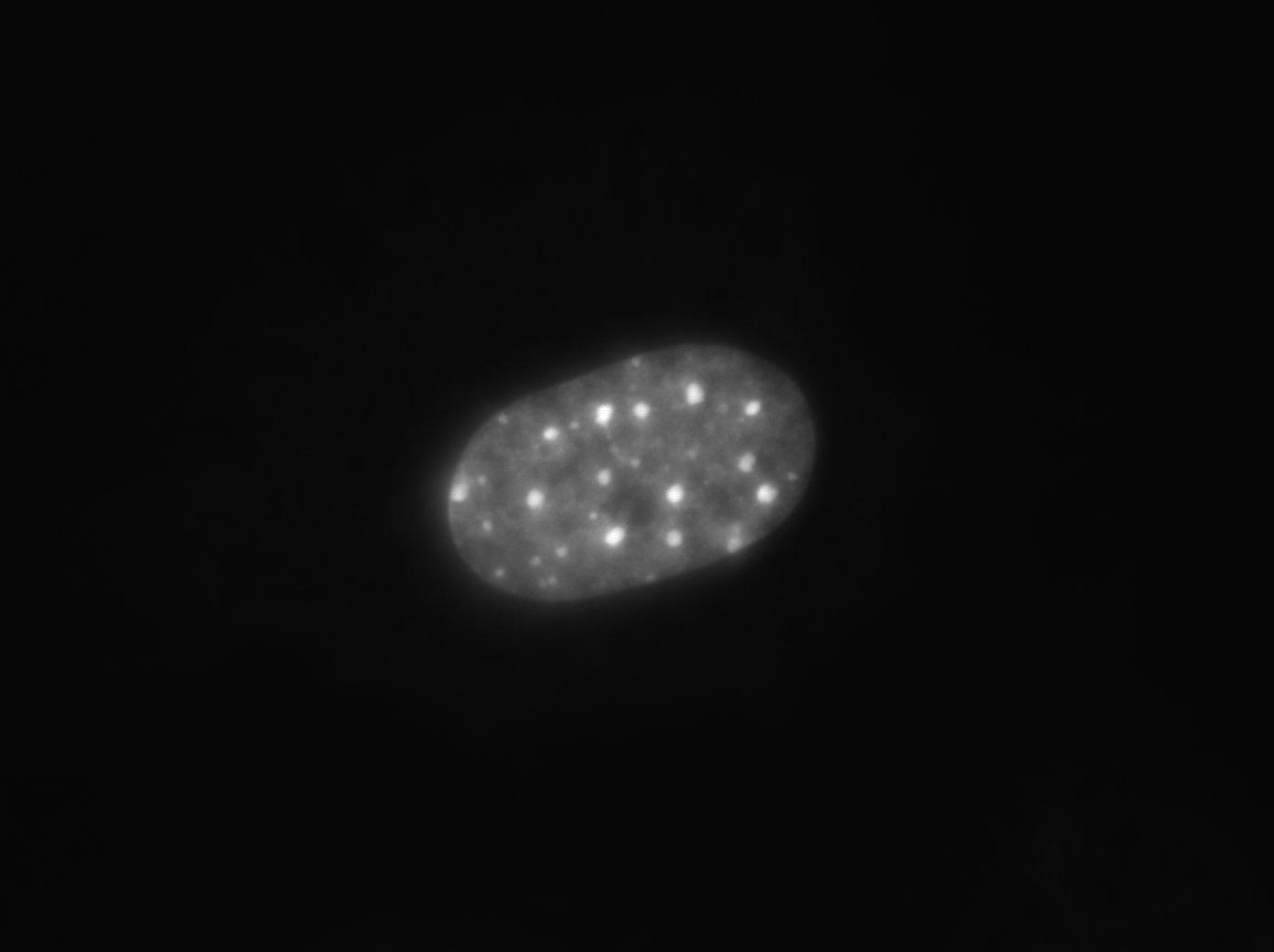

Supplement: Supplementary file 13 — Figures EV and Appendix Source Data [file 44318_2024_348_MOESM13_ESM.zip › SD figure EV and Appendix/Appendix Figure 1G/Baz1b/360-2.jpg]

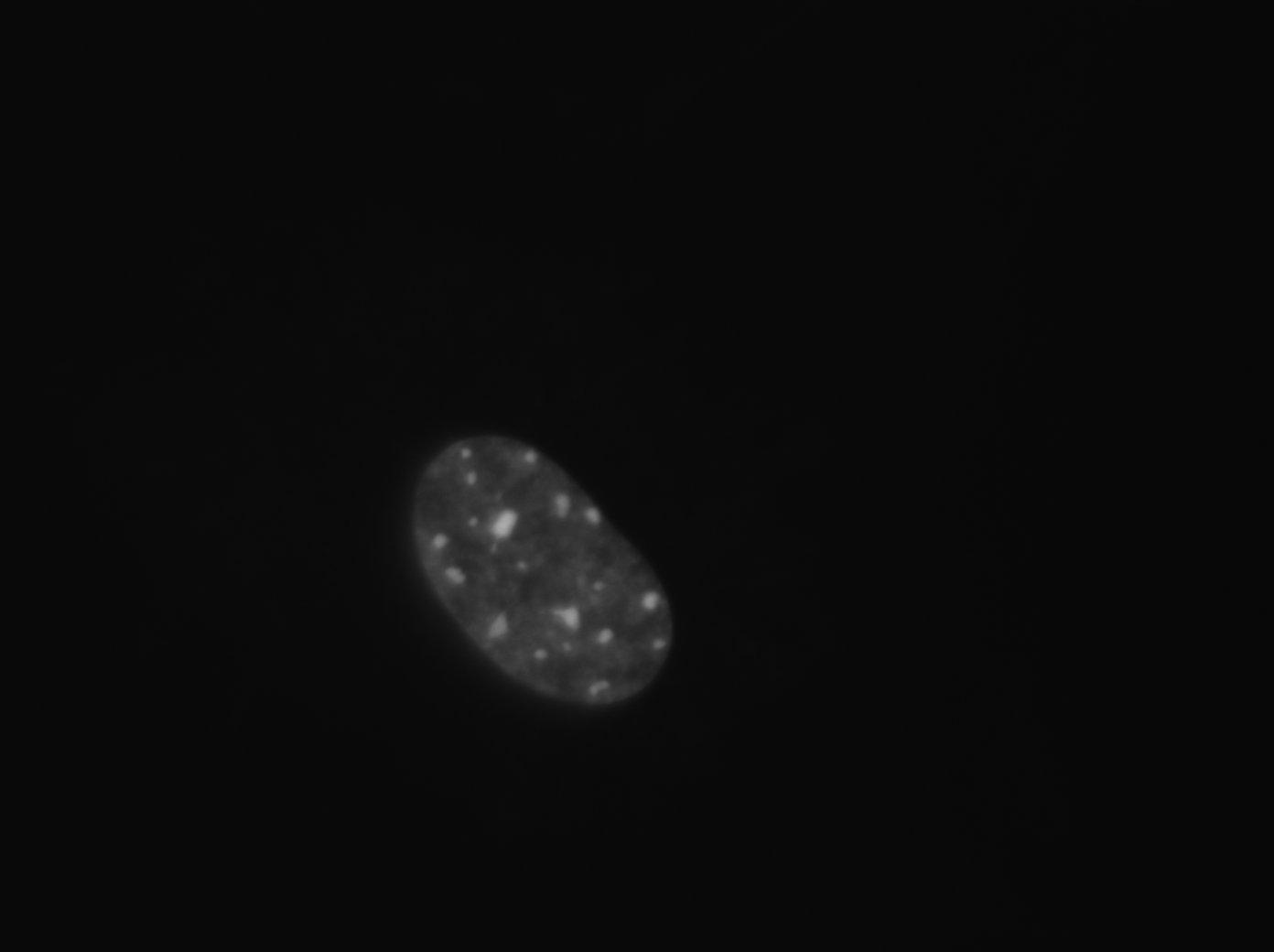

Supplement: Supplementary file 13 — Figures EV and Appendix Source Data [file 44318_2024_348_MOESM13_ESM.zip › SD figure EV and Appendix/Appendix Figure 1G/Baz1b/360-3.jpg]

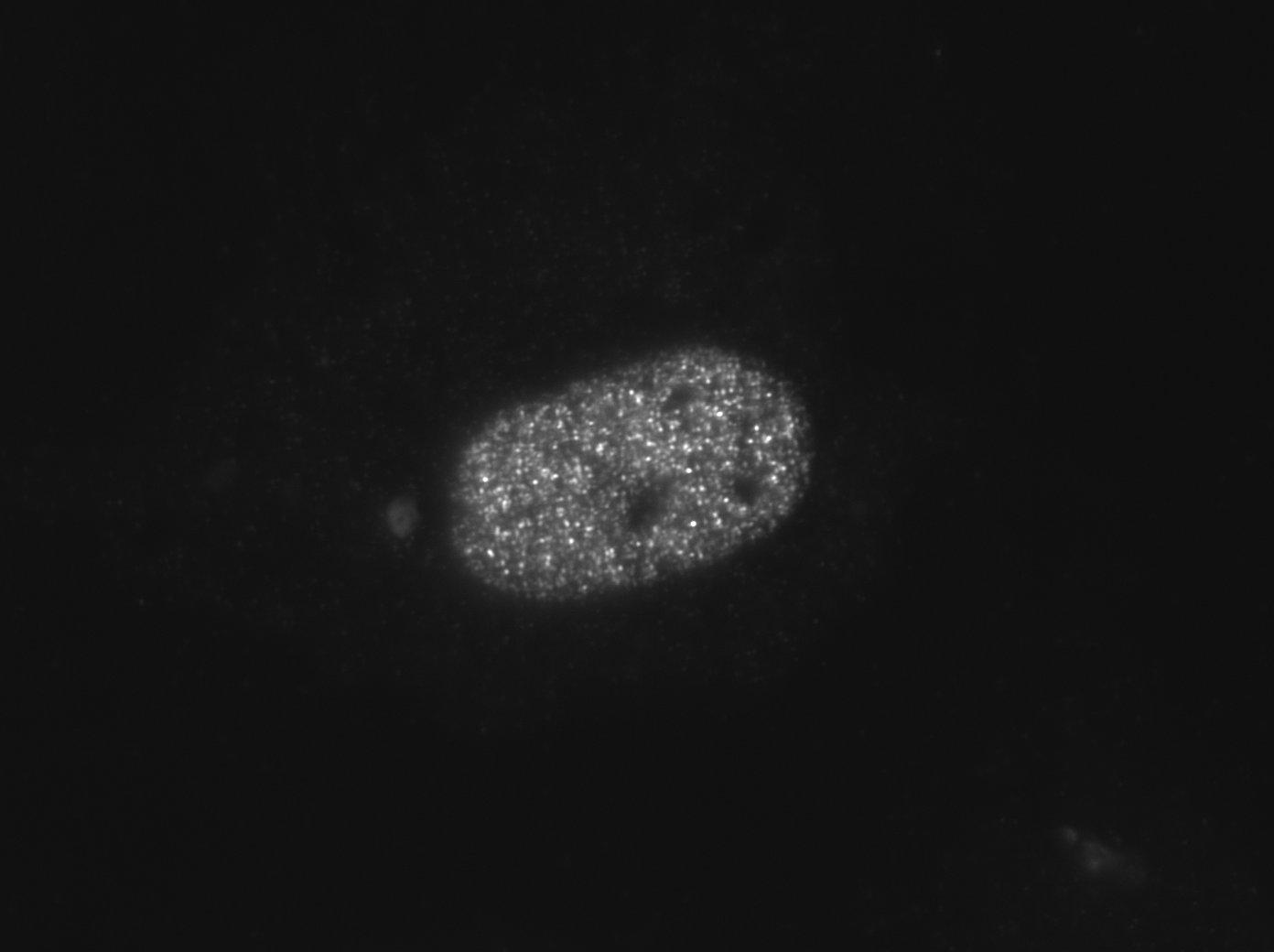

Supplement: Supplementary file 13 — Figures EV and Appendix Source Data [file 44318_2024_348_MOESM13_ESM.zip › SD figure EV and Appendix/Appendix Figure 1G/Baz1b/480-2.jpg]

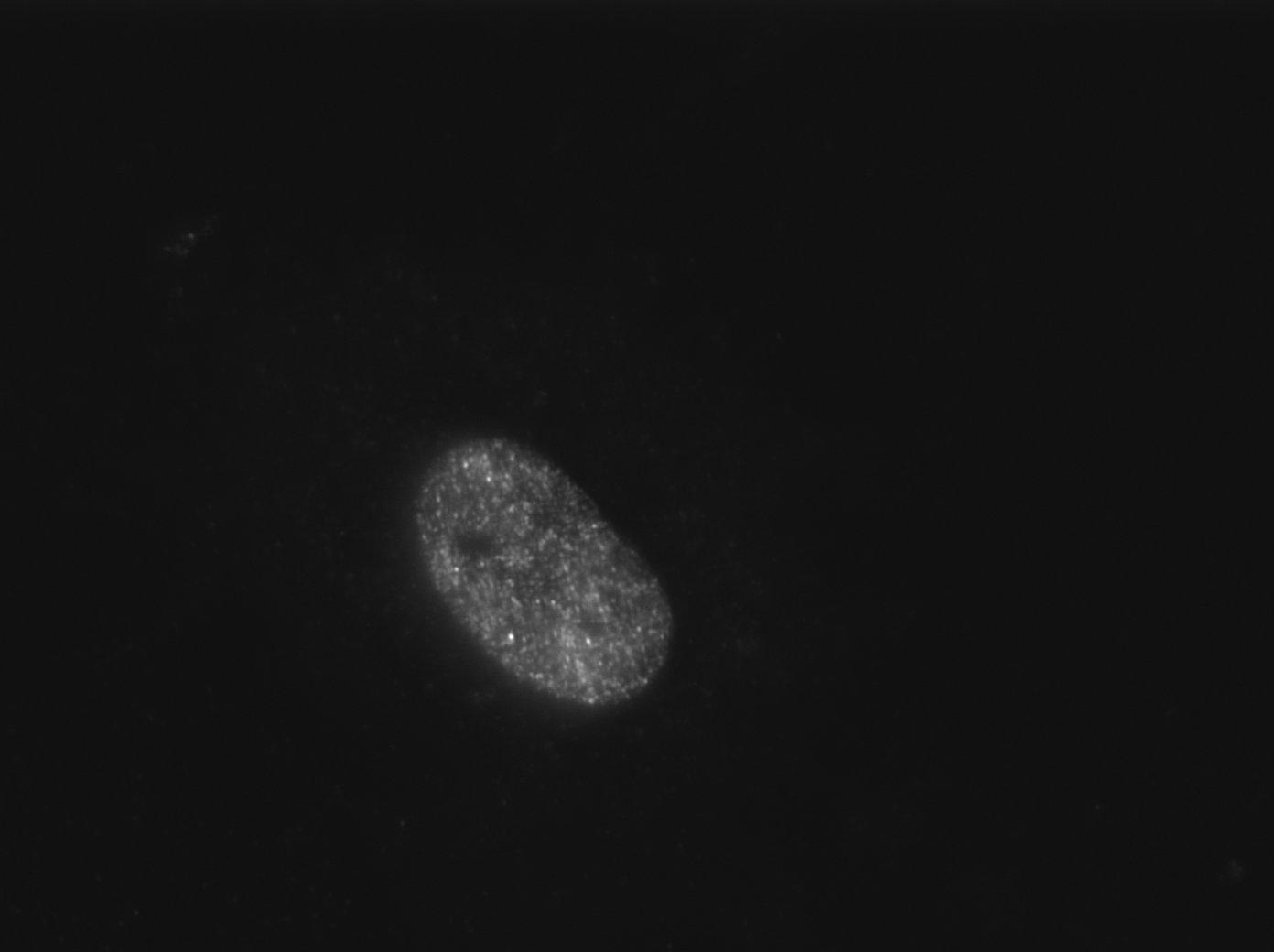

Supplement: Supplementary file 13 — Figures EV and Appendix Source Data [file 44318_2024_348_MOESM13_ESM.zip › SD figure EV and Appendix/Appendix Figure 1G/Baz1b/480-3.jpg]

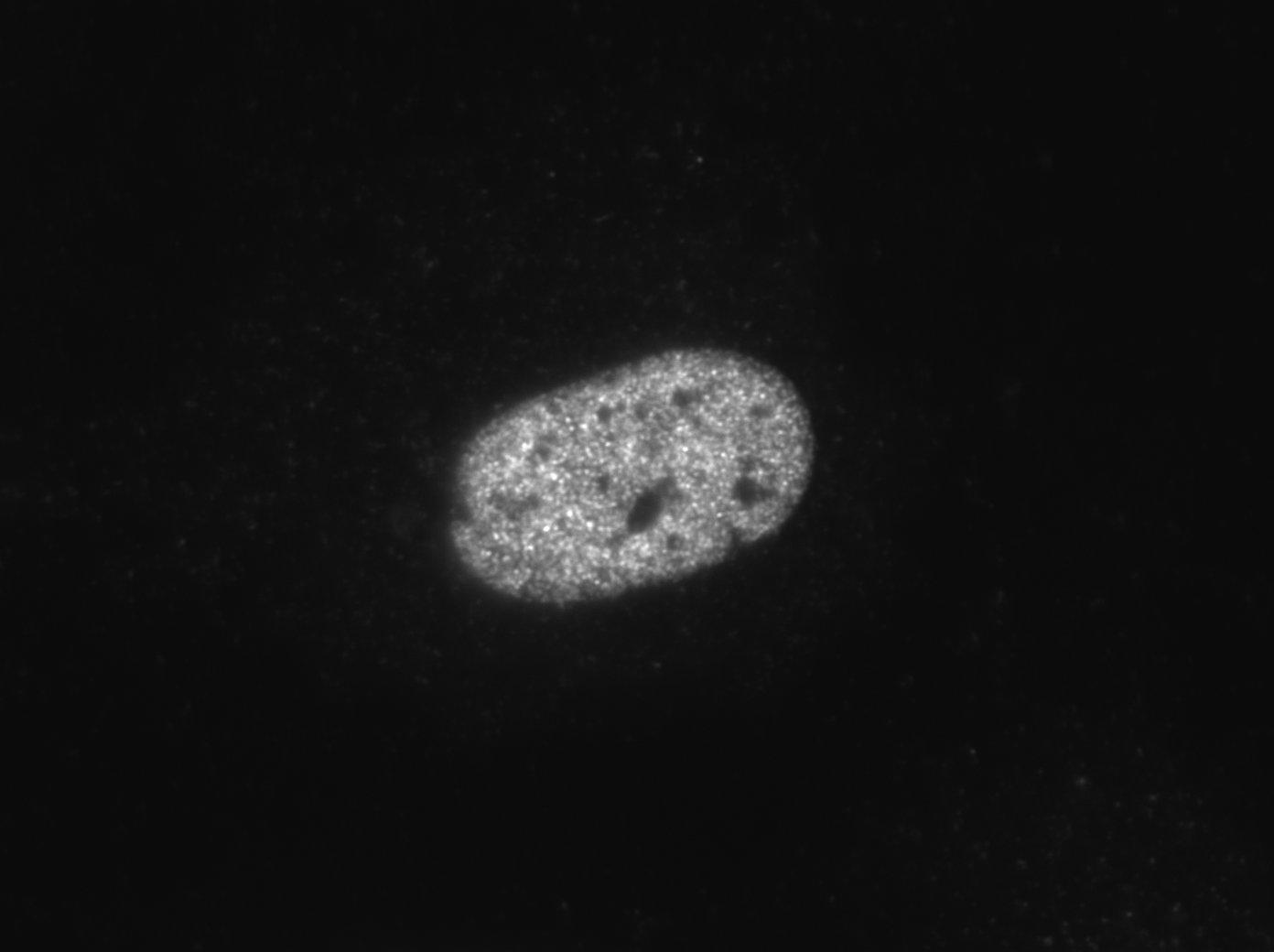

Supplement: Supplementary file 13 — Figures EV and Appendix Source Data [file 44318_2024_348_MOESM13_ESM.zip › SD figure EV and Appendix/Appendix Figure 1G/Baz1b/560-2.jpg]

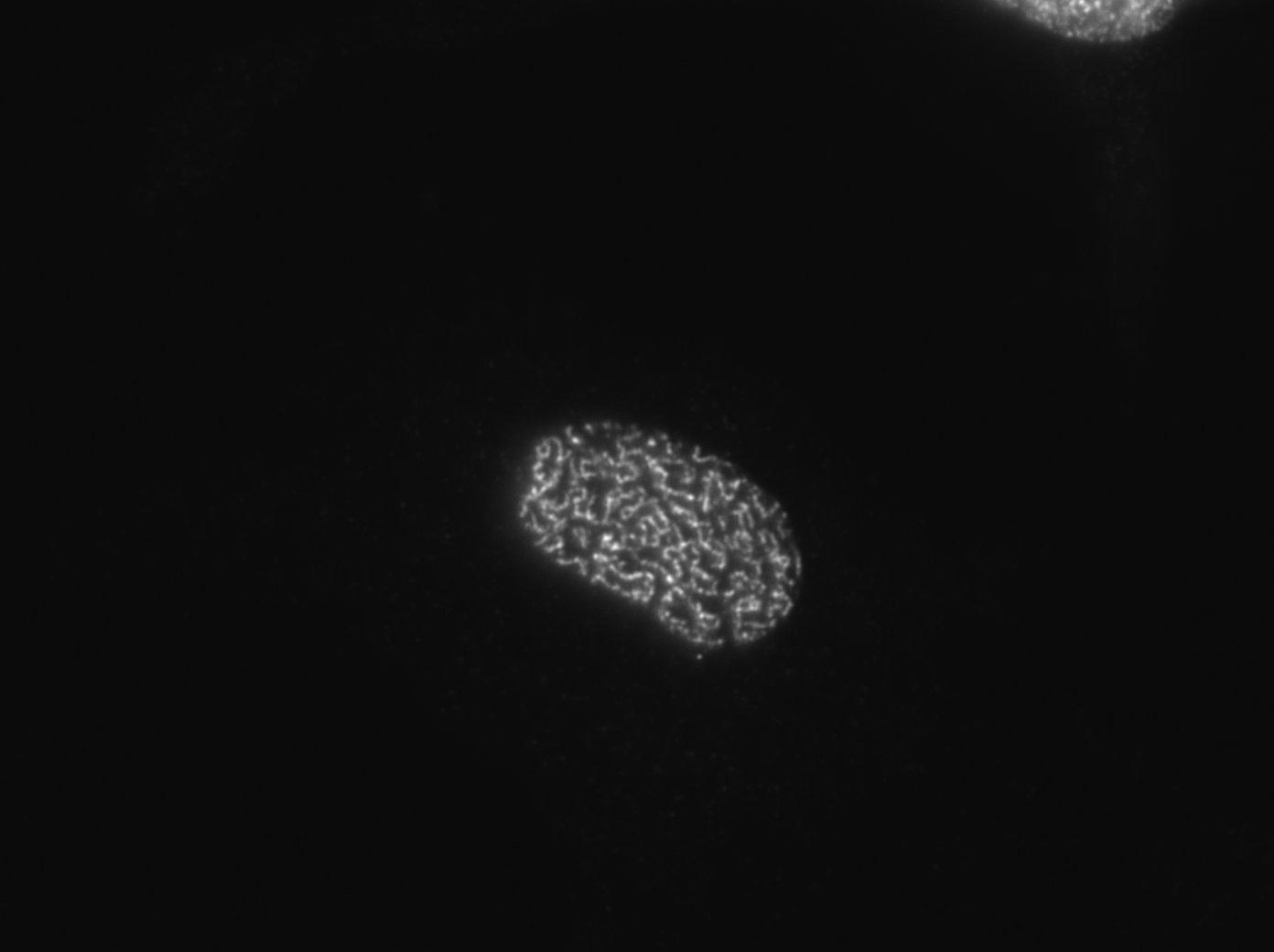

Supplement: Supplementary file 13 — Figures EV and Appendix Source Data [file 44318_2024_348_MOESM13_ESM.zip › SD figure EV and Appendix/Appendix Figure 1G/Chd4/560-3.jpg]

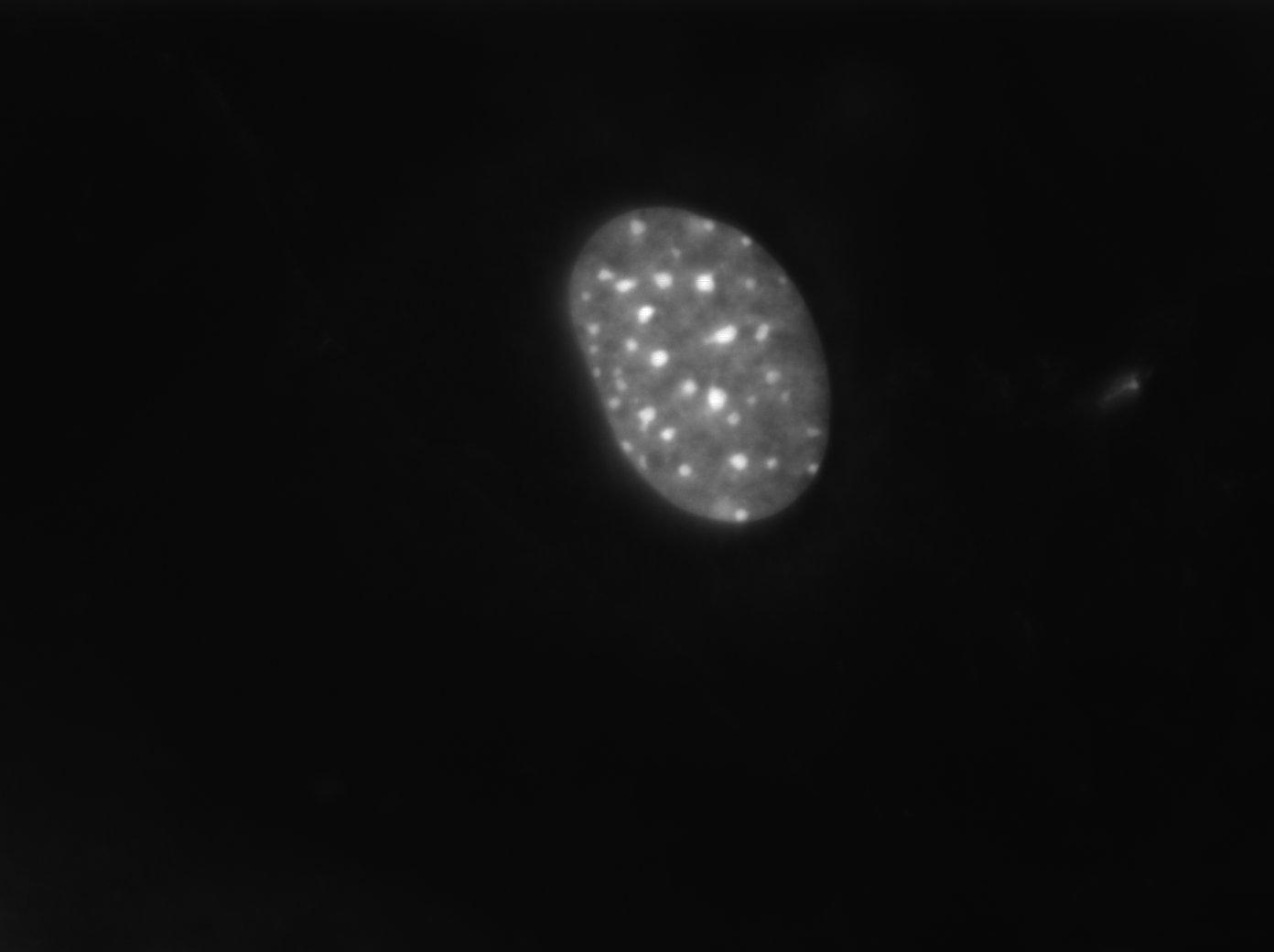

Supplement: Supplementary file 13 — Figures EV and Appendix Source Data [file 44318_2024_348_MOESM13_ESM.zip › SD figure EV and Appendix/Appendix Figure 1G/Chd4/360-2.jpg]

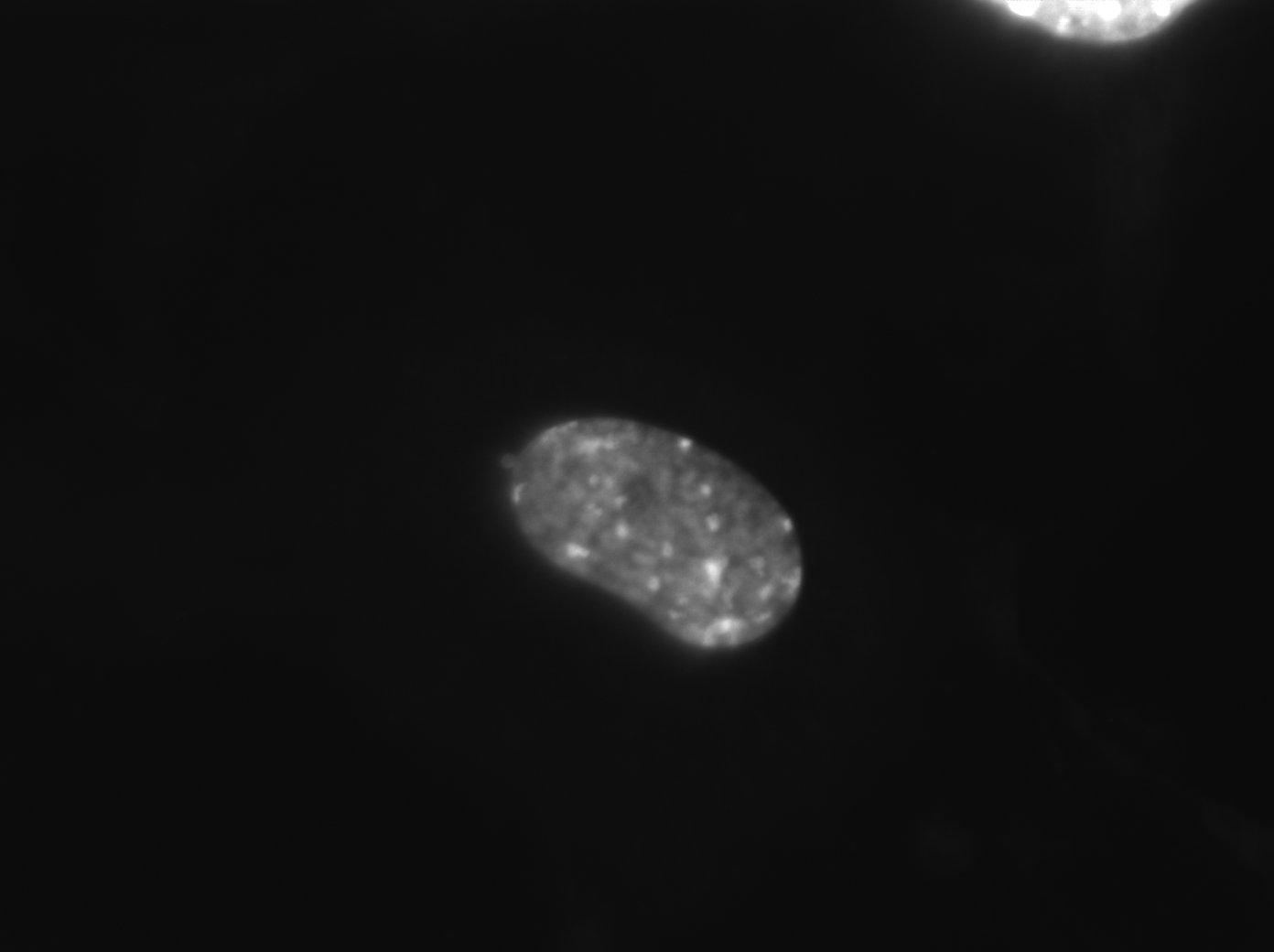

Supplement: Supplementary file 13 — Figures EV and Appendix Source Data [file 44318_2024_348_MOESM13_ESM.zip › SD figure EV and Appendix/Appendix Figure 1G/Chd4/360-3.jpg]

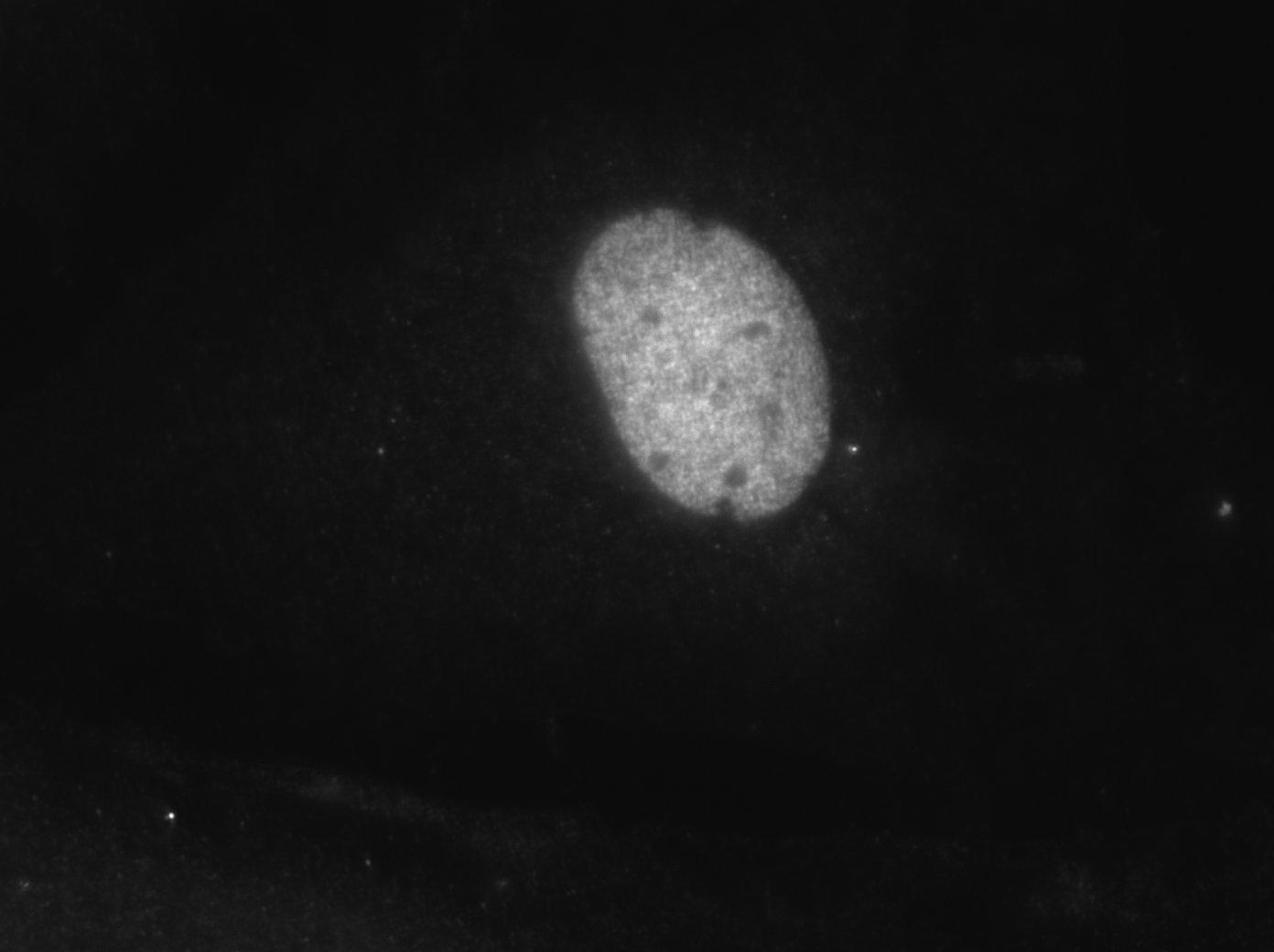

Supplement: Supplementary file 13 — Figures EV and Appendix Source Data [file 44318_2024_348_MOESM13_ESM.zip › SD figure EV and Appendix/Appendix Figure 1G/Chd4/480-2.jpg]

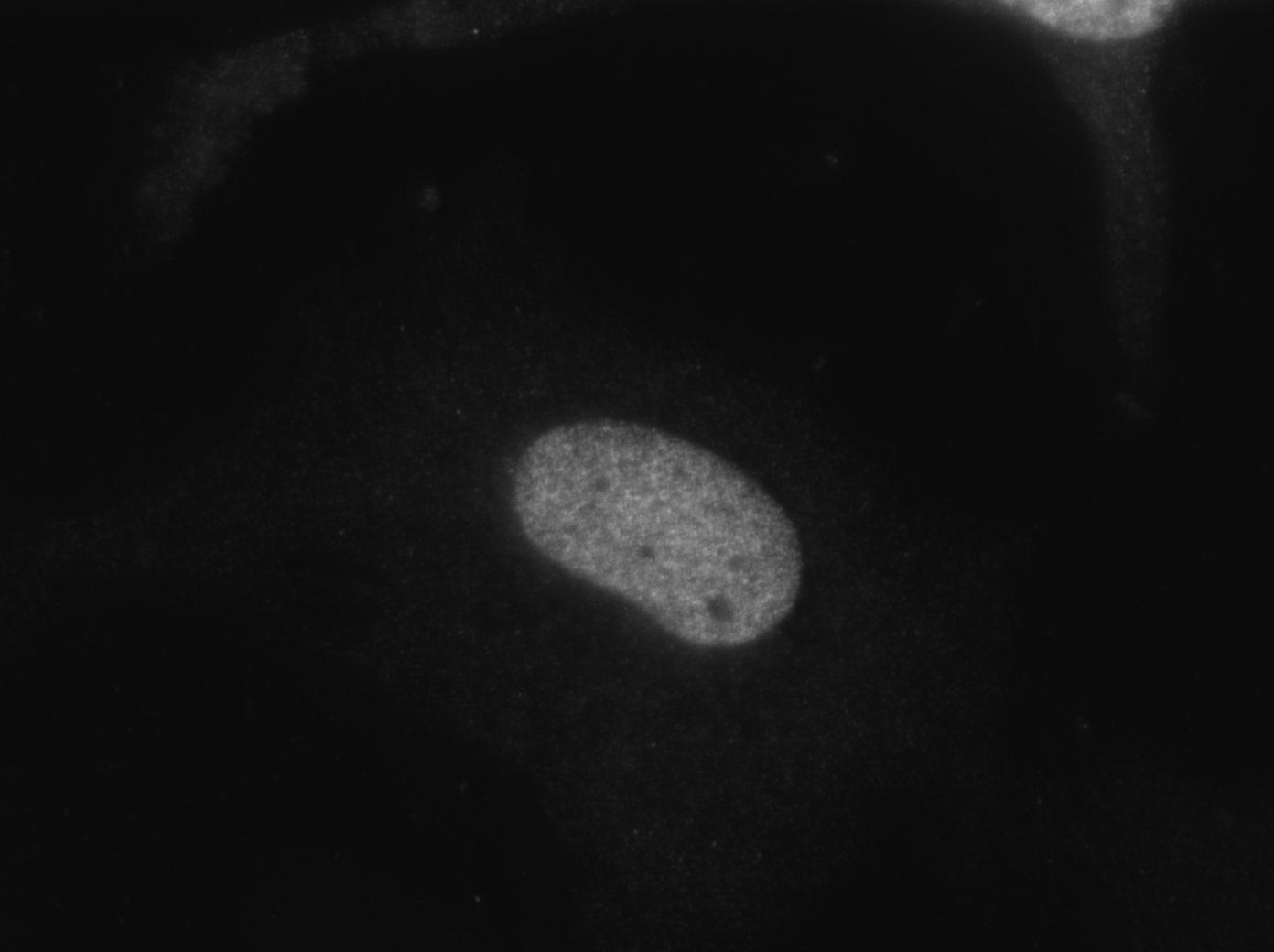

Supplement: Supplementary file 13 — Figures EV and Appendix Source Data [file 44318_2024_348_MOESM13_ESM.zip › SD figure EV and Appendix/Appendix Figure 1G/Chd4/480-3.jpg]

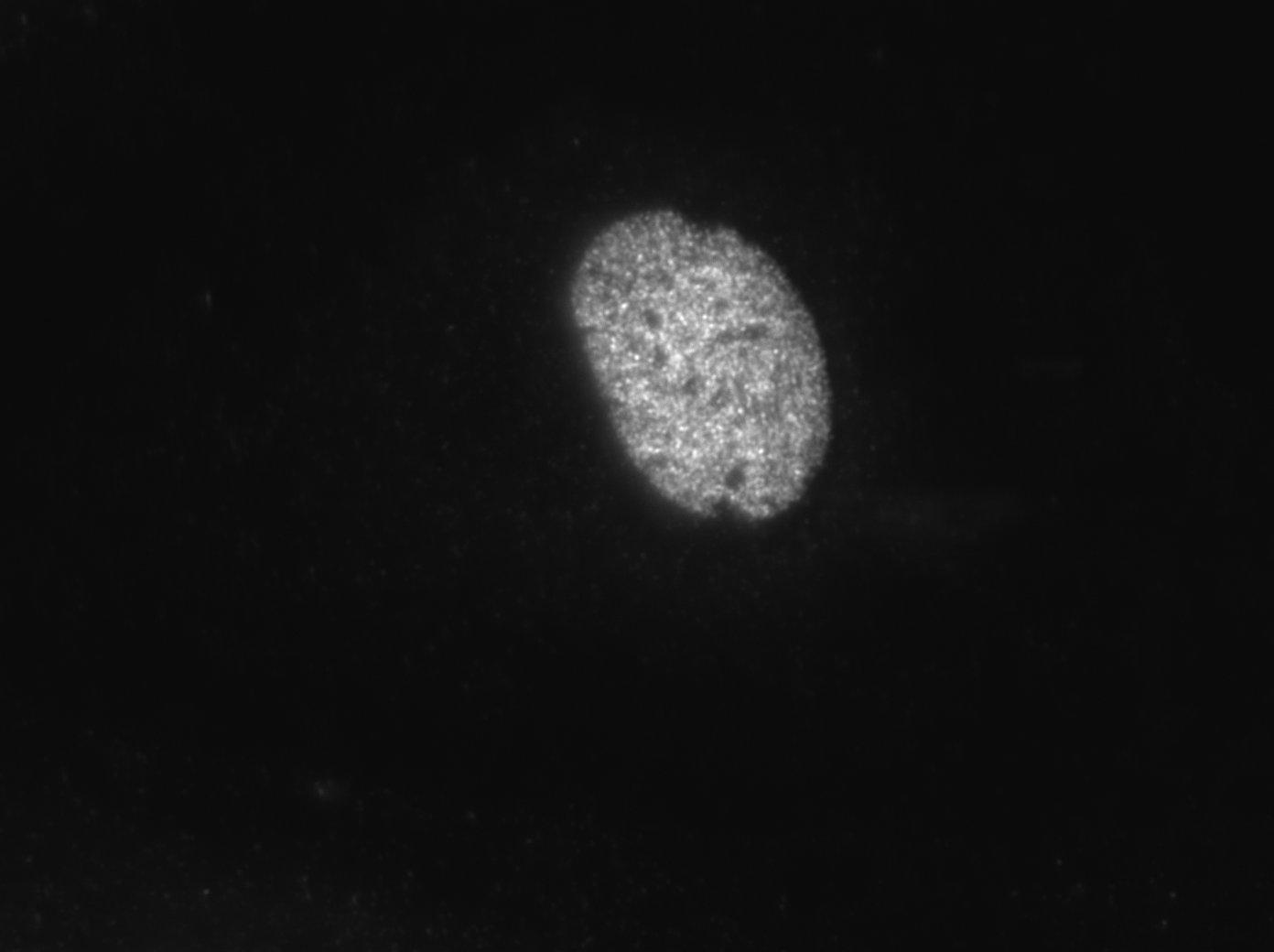

Supplement: Supplementary file 13 — Figures EV and Appendix Source Data [file 44318_2024_348_MOESM13_ESM.zip › SD figure EV and Appendix/Appendix Figure 1G/Chd4/560-2.jpg]

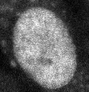

Supplement: Supplementary file 13 — Figures EV and Appendix Source Data [file 44318_2024_348_MOESM13_ESM.zip › SD figure EV and Appendix/Appendix Figure 4C/control/SUM_Registered-1.tif]

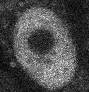

Supplement: Supplementary file 13 — Figures EV and Appendix Source Data [file 44318_2024_348_MOESM13_ESM.zip › SD figure EV and Appendix/Appendix Figure 4C/control/SUM_Registered-2.tif]

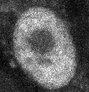

Supplement: Supplementary file 13 — Figures EV and Appendix Source Data [file 44318_2024_348_MOESM13_ESM.zip › SD figure EV and Appendix/Appendix Figure 4C/control/SUM_Registered-3.tif]

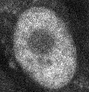

Supplement: Supplementary file 13 — Figures EV and Appendix Source Data [file 44318_2024_348_MOESM13_ESM.zip › SD figure EV and Appendix/Appendix Figure 4C/control/SUM_Registered-4.tif]

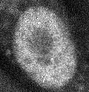

Supplement: Supplementary file 13 — Figures EV and Appendix Source Data [file 44318_2024_348_MOESM13_ESM.zip › SD figure EV and Appendix/Appendix Figure 4C/control/SUM_Registered-5.tif]

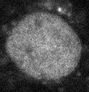

Supplement: Supplementary file 13 — Figures EV and Appendix Source Data [file 44318_2024_348_MOESM13_ESM.zip › SD figure EV and Appendix/Appendix Figure 4C/phf2 kd/SUM_Registered-1.tif]

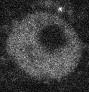

Supplement: Supplementary file 13 — Figures EV and Appendix Source Data [file 44318_2024_348_MOESM13_ESM.zip › SD figure EV and Appendix/Appendix Figure 4C/phf2 kd/SUM_Registered-2.tif]

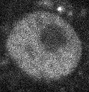

Supplement: Supplementary file 13 — Figures EV and Appendix Source Data [file 44318_2024_348_MOESM13_ESM.zip › SD figure EV and Appendix/Appendix Figure 4C/phf2 kd/SUM_Registered-3.tif]

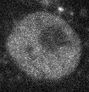

Supplement: Supplementary file 13 — Figures EV and Appendix Source Data [file 44318_2024_348_MOESM13_ESM.zip › SD figure EV and Appendix/Appendix Figure 4C/phf2 kd/SUM_Registered-4.tif]

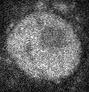

Supplement: Supplementary file 13 — Figures EV and Appendix Source Data [file 44318_2024_348_MOESM13_ESM.zip › SD figure EV and Appendix/Appendix Figure 4C/phf2 kd/SUM_Registered-5.tif]

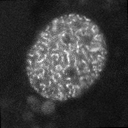

Supplement: Supplementary file 13 — Figures EV and Appendix Source Data [file 44318_2024_348_MOESM13_ESM.zip › SD figure EV and Appendix/Appendix Figure 4C/wapl ko/SUM_Registered.tif]

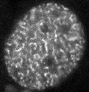

Supplement: Supplementary file 13 — Figures EV and Appendix Source Data [file 44318_2024_348_MOESM13_ESM.zip › SD figure EV and Appendix/Appendix Figure 4C/wapl ko/SUM_Registered-1.tif]

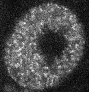

Supplement: Supplementary file 13 — Figures EV and Appendix Source Data [file 44318_2024_348_MOESM13_ESM.zip › SD figure EV and Appendix/Appendix Figure 4C/wapl ko/SUM_Registered-2.tif]

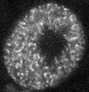

Supplement: Supplementary file 13 — Figures EV and Appendix Source Data [file 44318_2024_348_MOESM13_ESM.zip › SD figure EV and Appendix/Appendix Figure 4C/wapl ko/SUM_Registered-3.tif]

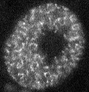

Supplement: Supplementary file 13 — Figures EV and Appendix Source Data [file 44318_2024_348_MOESM13_ESM.zip › SD figure EV and Appendix/Appendix Figure 4C/wapl ko/SUM_Registered-4.tif]

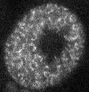

Supplement: Supplementary file 13 — Figures EV and Appendix Source Data [file 44318_2024_348_MOESM13_ESM.zip › SD figure EV and Appendix/Appendix Figure 4C/wapl ko/SUM_Registered-5.tif]

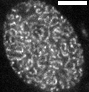

Supplement: Supplementary file 13 — Figures EV and Appendix Source Data [file 44318_2024_348_MOESM13_ESM.zip › SD figure EV and Appendix/Appendix Figure 4C/wapl ko, phf2 kd/SUM_Registered-1.tif]

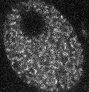

Supplement: Supplementary file 13 — Figures EV and Appendix Source Data [file 44318_2024_348_MOESM13_ESM.zip › SD figure EV and Appendix/Appendix Figure 4C/wapl ko, phf2 kd/SUM_Registered-2.tif]

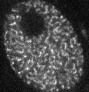

Supplement: Supplementary file 13 — Figures EV and Appendix Source Data [file 44318_2024_348_MOESM13_ESM.zip › SD figure EV and Appendix/Appendix Figure 4C/wapl ko, phf2 kd/SUM_Registered-3.tif]

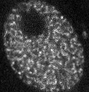

Supplement: Supplementary file 13 — Figures EV and Appendix Source Data [file 44318_2024_348_MOESM13_ESM.zip › SD figure EV and Appendix/Appendix Figure 4C/wapl ko, phf2 kd/SUM_Registered-4.tif]

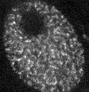

Supplement: Supplementary file 13 — Figures EV and Appendix Source Data [file 44318_2024_348_MOESM13_ESM.zip › SD figure EV and Appendix/Appendix Figure 4C/wapl ko, phf2 kd/SUM_Registered-5.tif]

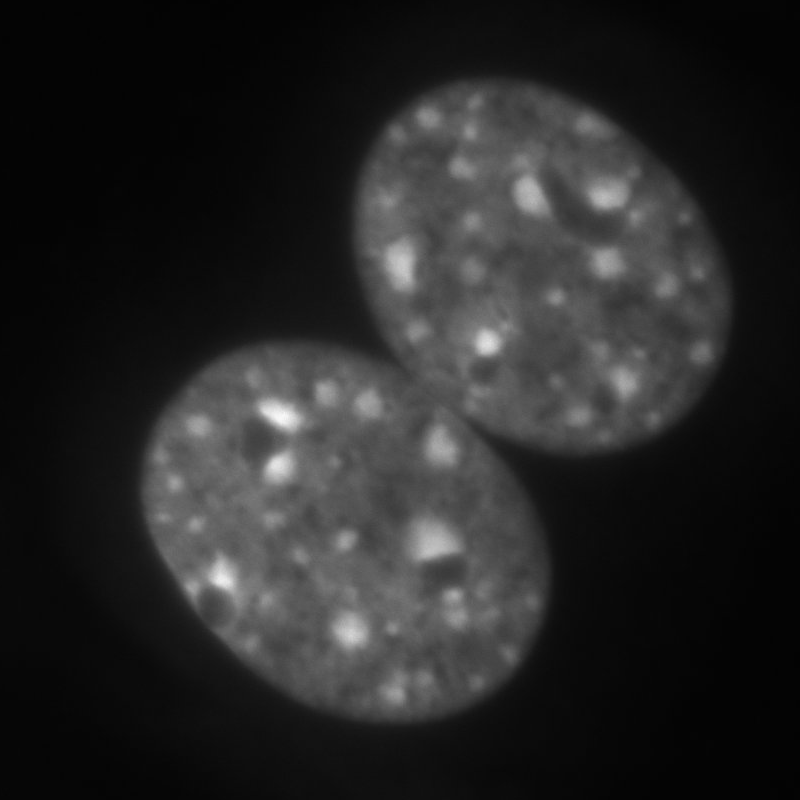

Supplement: Supplementary file 13 — Figures EV and Appendix Source Data [file 44318_2024_348_MOESM13_ESM.zip › SD figure EV and Appendix/Appendix Figure 1F/top2b/Untitled-1.tif]

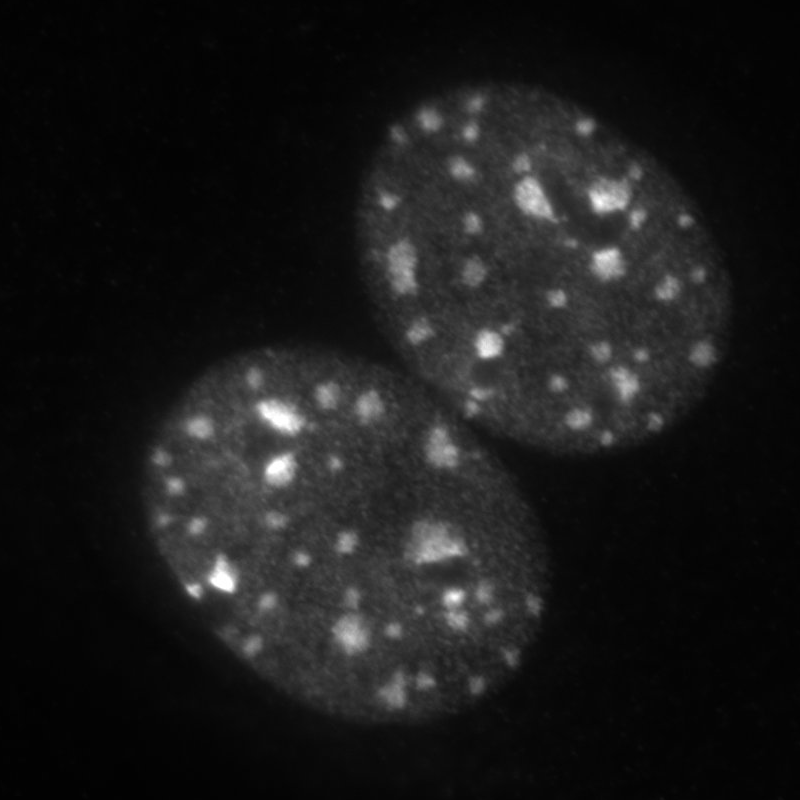

Supplement: Supplementary file 13 — Figures EV and Appendix Source Data [file 44318_2024_348_MOESM13_ESM.zip › SD figure EV and Appendix/Appendix Figure 1F/top2b/Untitled-2.tif]

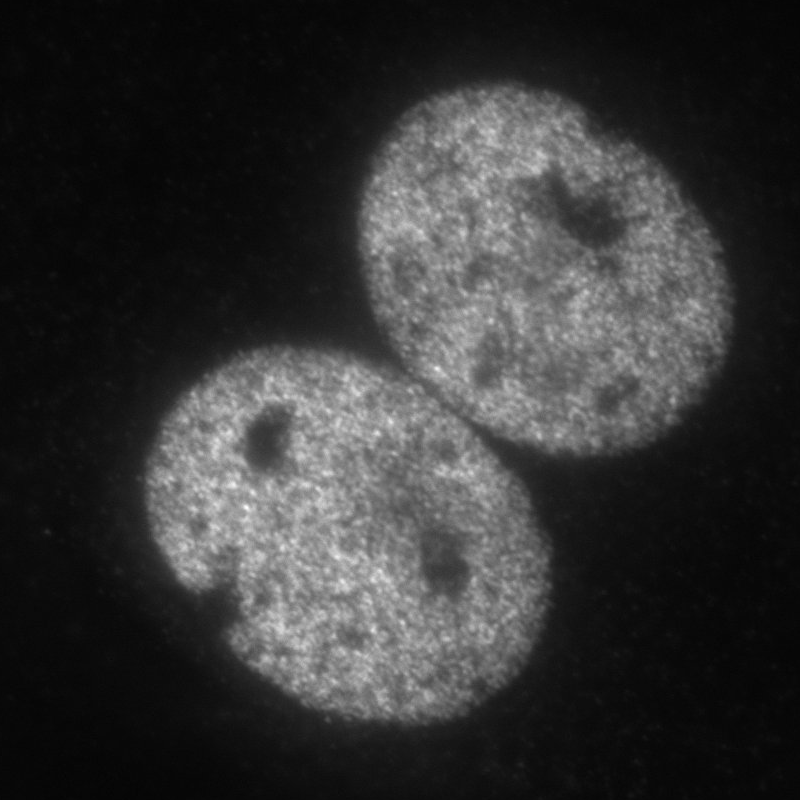

Supplement: Supplementary file 13 — Figures EV and Appendix Source Data [file 44318_2024_348_MOESM13_ESM.zip › SD figure EV and Appendix/Appendix Figure 1F/top2b/Untitled-3.tif]

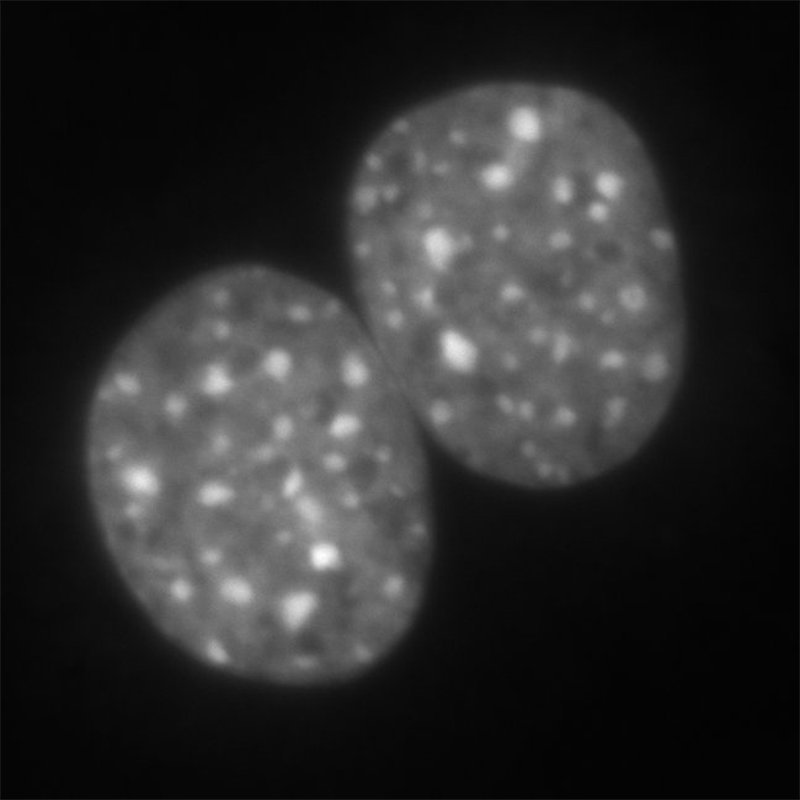

Supplement: Supplementary file 13 — Figures EV and Appendix Source Data [file 44318_2024_348_MOESM13_ESM.zip › SD figure EV and Appendix/Appendix Figure 1F/top2b/Untitled-4.tif]

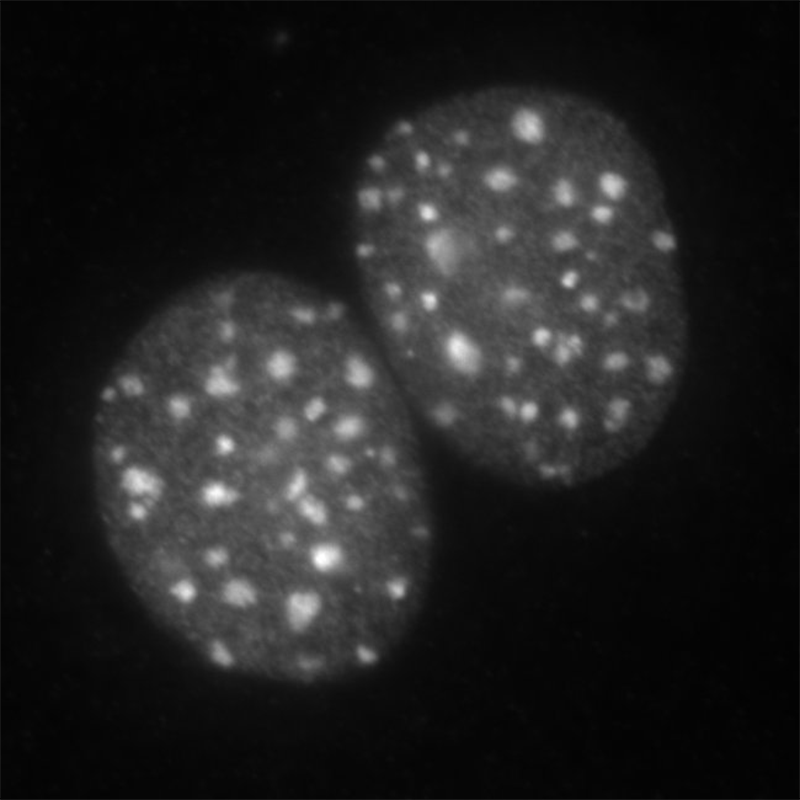

Supplement: Supplementary file 13 — Figures EV and Appendix Source Data [file 44318_2024_348_MOESM13_ESM.zip › SD figure EV and Appendix/Appendix Figure 1F/top2b/Untitled-5.tif]

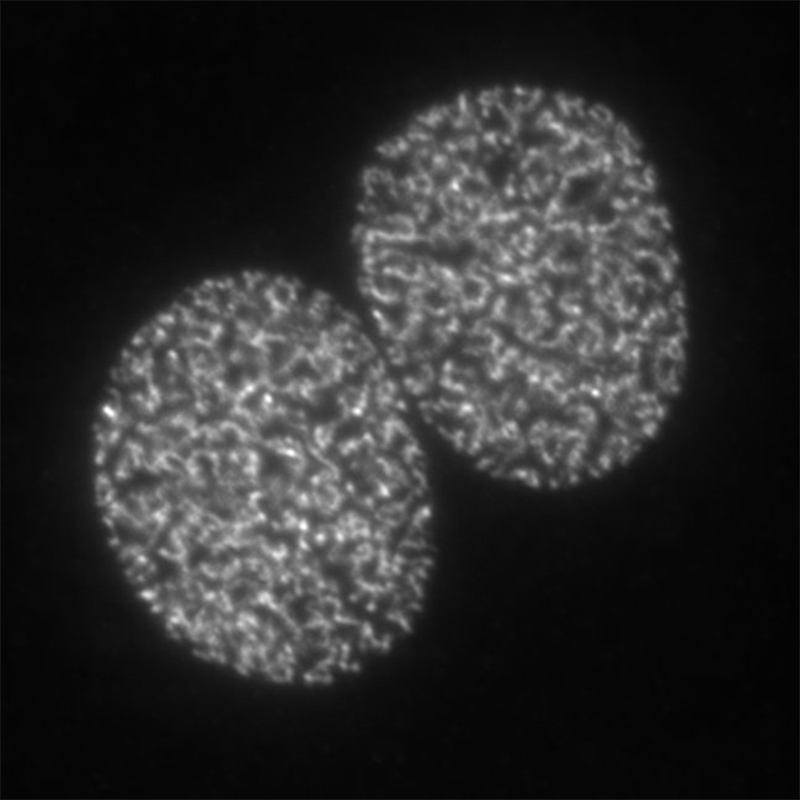

Supplement: Supplementary file 13 — Figures EV and Appendix Source Data [file 44318_2024_348_MOESM13_ESM.zip › SD figure EV and Appendix/Appendix Figure 1F/top2b/Untitled-6.tif]
