# Supplementary material for: Cohesin positions the epigenetic reader Phf2 within the genome
Source: EMBO J. 2025 Jan 2;44(3):736–66. doi: 10.1038/s44318-024-00348-2 (PMC11790891; doi:10.1038/s44318-024-00348-2)
Supplement: Supplementary file 14 — Expanded View Figures [file 44318_2024_348_MOESM14_ESM.pdf]

Expanded View Figures

Figure EV1. Phf2 partially colocalizes with CTCF genome wide.

(**A**) Immunoblot analysis of Phf2 in *Phf2* KO MEFs. MEFs with floxed alleles of Phf2 with or without ERCre (*Phf2* F/F, ERCre/+ or *Phf2* F/F, no ERCre) were treated with 4-OHT for the indicated days and whole-cell extracts were analyzed using the indicated antibodies. (**B**) Venn diagram illustrating the overlap between ChIP-seq peaks of CTCF and Phf2 in WT MEFs. (**C**) Pile-up heat maps and summary plots of ChIP-seq signals for Phf2 (in WT and *Phf2* KO), H3K4me3, Smc3, CTCF, and PolII Ser5-P at overlap groups indicated in (**B**). The Zoom-in panels show the indicated sub-groups of ChIP-seq signals at different color scales. (**D**) Summary plots of ChIP-seq signals obtained for Phf2 and Smc3 at oriented CTCF sites. The binding at the CTCF sequence on the + and the – strand was assessed in WT (left) and *Wapl* KO MEFs (right). Source data are available online for this figure.

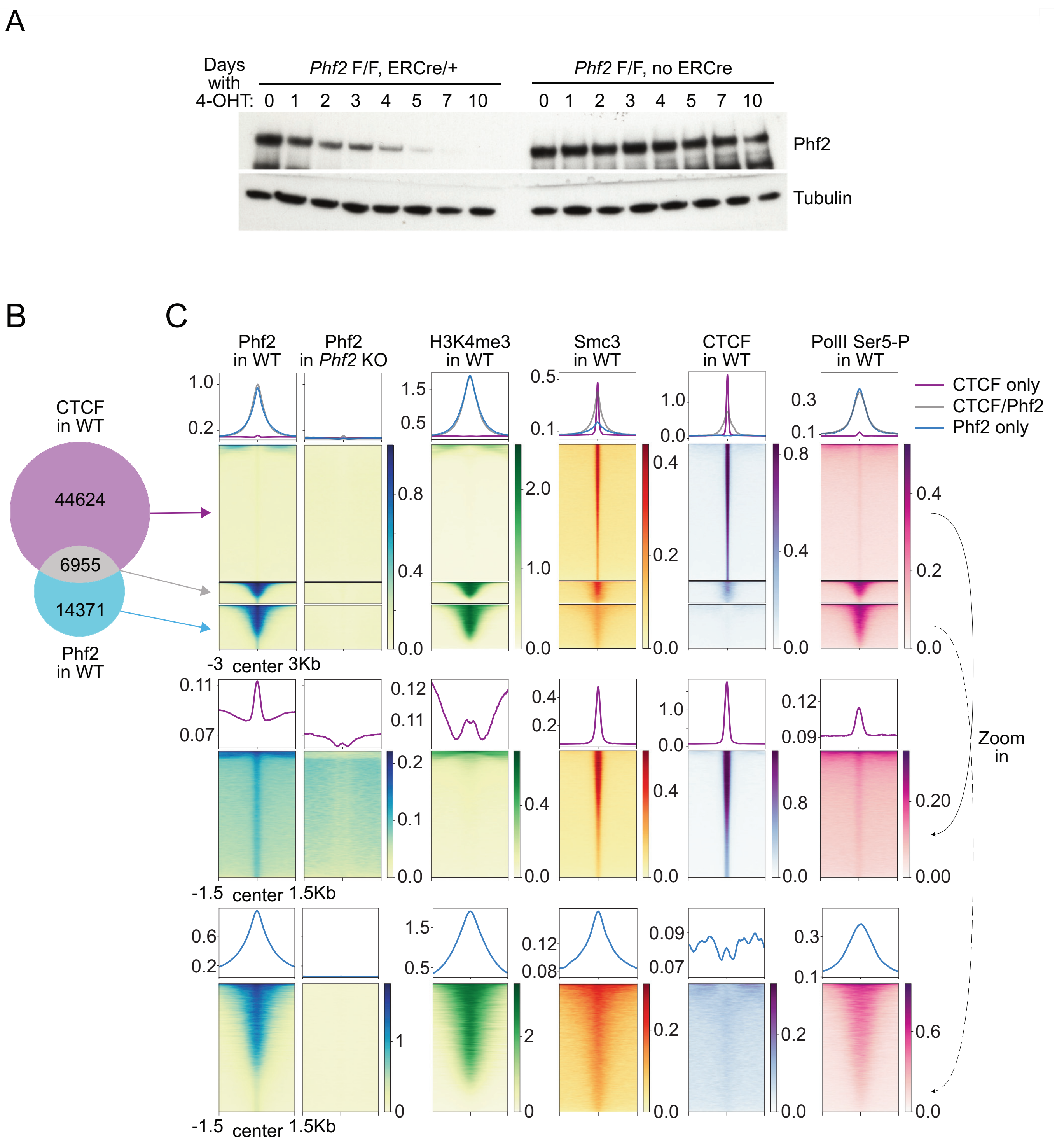
A
Days with 4-OHT: 0 1 2 3 4 5 7 10 0 1 2 3 4 5 7 10
Phf2 F/F, ERCre/+
Phf2 F/F, no ERCre
Phf2
Tubulin
B
CTCF in WT
44624
6955
14371
Phf2 in WT
C
Phf2 in WT
Phf2 in Phf2 KO
H3K4me3 in WT
Smc3 in WT
CTCF in WT
PolII Ser5-P in WT
CTCF only
CTCF/Phf2
Phf2 only
-3 center 3Kb
-1.5 center 1.5Kb
Zoom in
-1.5 center 1.5Kb
D
ChIP-seq signal in WT
CTCF -
CTCF +
Phf2
Smc3
ChIP-seq signal in Wapl KO
CTCF -
CTCF +
–1Kb
0
1Kb
–1Kb
0
1Kb

 © The Author(s)

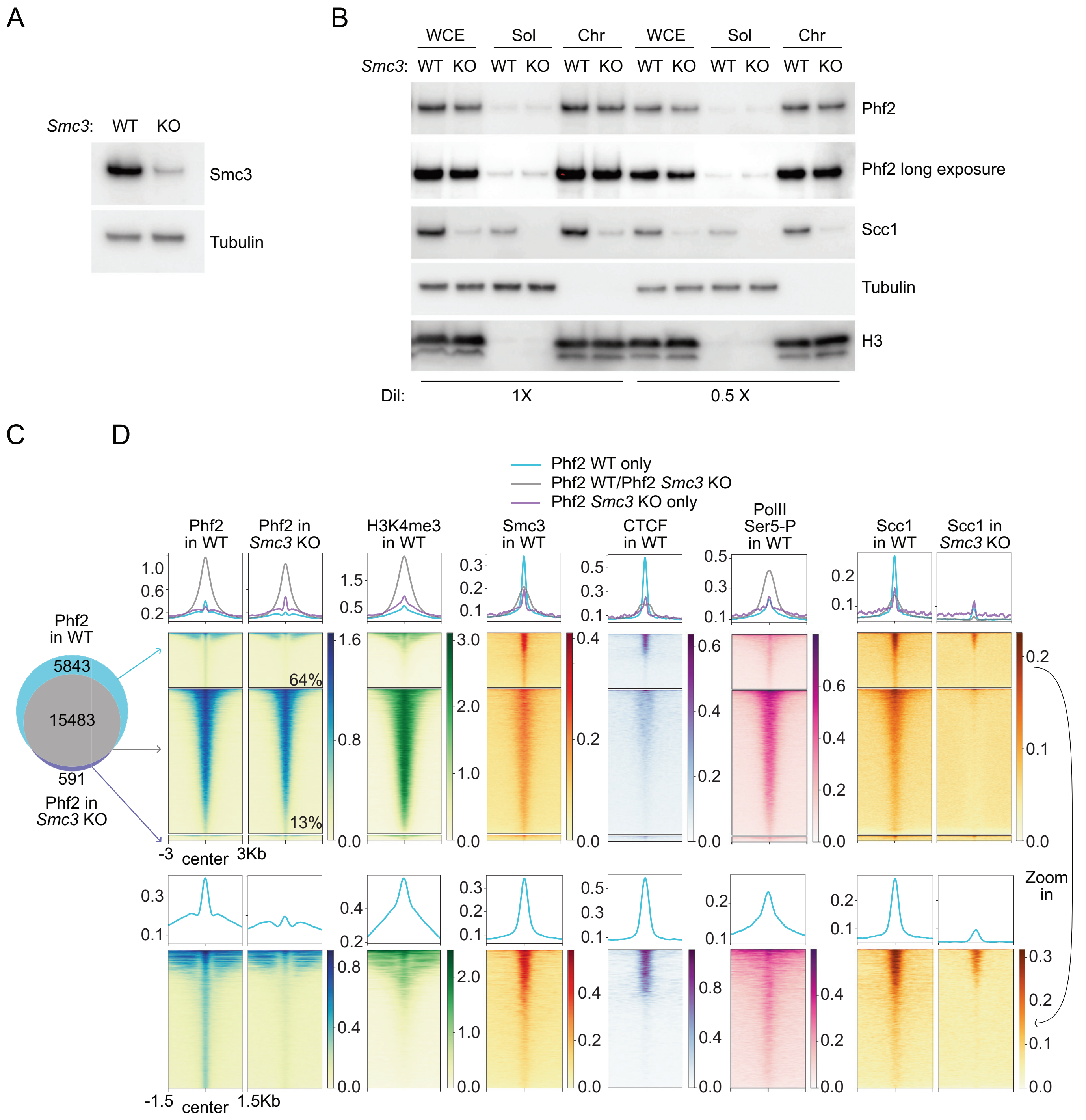

Figure EV2. Enrichment of Phf2 at sites lacking H3K4me3 is dependent on cohesin.

(**A**) Immunoblot analysis of whole-cell extracts from WT and *Smc3* KO MEFs using the indicated antibodies. (**B**) Immunoblot analysis of whole-cell extract (WCE), soluble (Sol), and chromatin-bound (Chr) fractions from WT and *Smc3* KO MEFs using the indicated antibodies. Two protein dilutions were assessed. Please note that a portion of this blot was used in Fig. 3A. (**C**) Venn diagram illustrating the overlap between ChIP-seq peaks of Phf2 in WT and *Smc3* KO MEFs. (**D**) Pile-up heat maps and summary plots of ChIP-seq signals for Phf2 (in WT and *Smc3* KO), H3K4me3, Smc3, CTCF, PolII Ser5-P, and Scc1 (in WT and *Smc3* KO; data from Busslinger et al, 2017) at overlap groups indicated in (**C**). Phf2 peaks present only in WT MEFs were reduced by 64% in read numbers upon Smc3 depletion, whereas Phf2 peaks that were present in WT and *Smc3* KO MEFs were reduced by only 13%. Zoom-in panels show the indicated sub-groups of ChIP-seq signals at different color scales. The panels from Fig. 3D are shown here again to facilitate a direct side-by-side comparison with the extended panels presented in this figure.

© The Author(s)

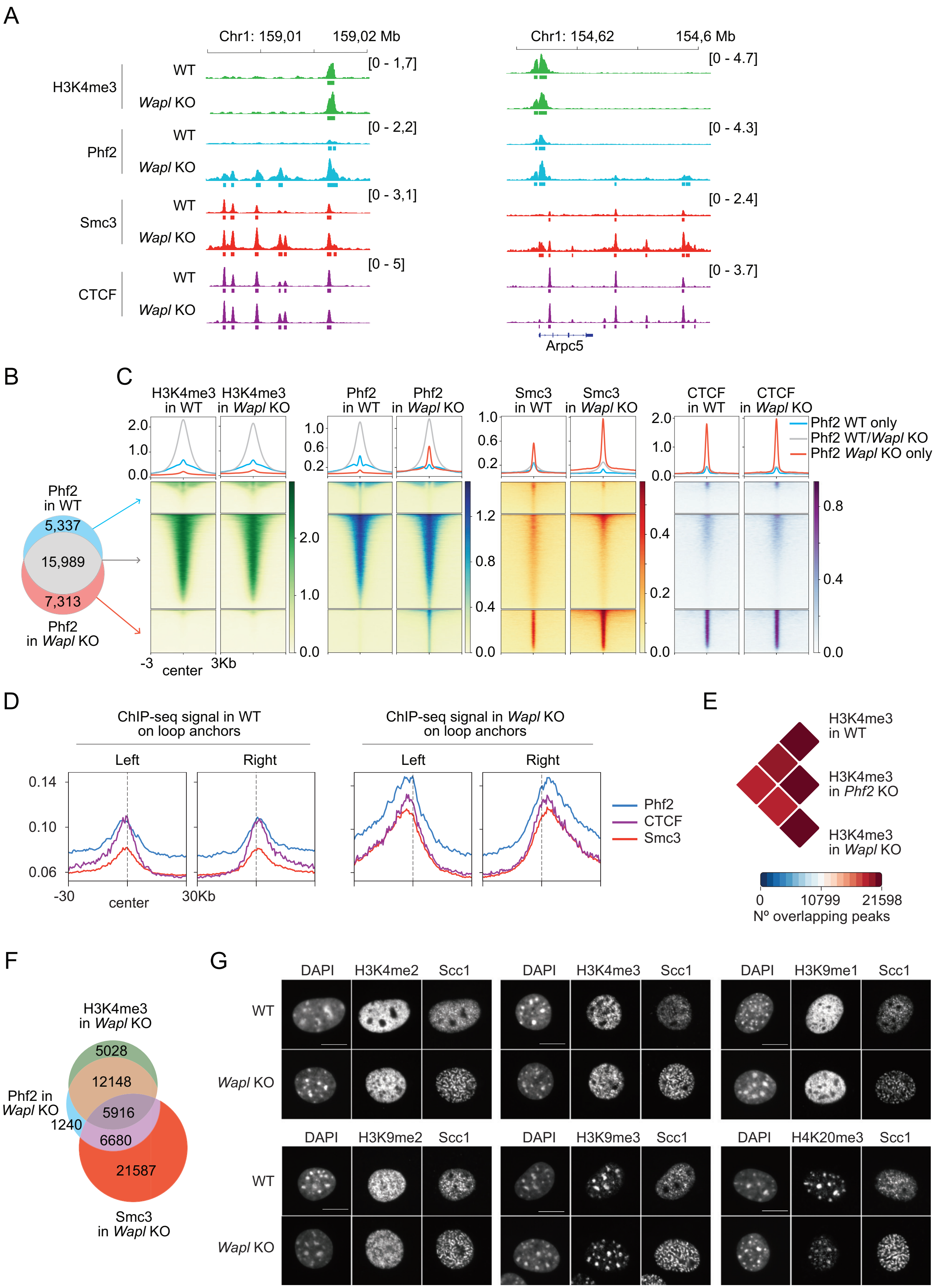
A
Chr1: 159,01
159,02 Mb
Chr1: 154,62
154,6 Mb
H3K4me3
WT
Wapl KO
[0 - 1,7]
[0 - 4.7]
Phf2
[0 - 2,2]
[0 - 4.3]
Smc3
[0 - 3,1]
[0 - 2.4]
CTCF
[0 - 5]
[0 - 3.7]
Arpc5
B
Phf2 in WT
5,337
15,989
7,313
Phf2 in Wapl KO
C
H3K4me3 in WT
H3K4me3 in Wapl KO
Phf2 in WT
Phf2 in Wapl KO
Smc3 in WT
Smc3 in Wapl KO
CTCF in WT
CTCF in Wapl KO
Phf2 WT only
Phf2 WT/Wapl KO
Phf2 Wapl KO only
-3
center
3Kb
D
ChIP-seq signal in WT on loop anchors
ChIP-seq signal in Wapl KO on loop anchors
Left
Right
0.14
0.10
0.06
-30
center
30Kb
Phf2
CTCF
Smc3
E
H3K4me3 in WT
H3K4me3 in Phf2 KO
H3K4me3 in Wapl KO
0
10799
21598
Nº overlapping peaks
F
H3K4me3 in Wapl KO
5028
12148
5916
Phf2 in Wapl KO
1240
6680
21587
Smc3 in Wapl KO
G
DAPI
H3K4me2
Scc1
H3K4me3
H3K9me1
H3K9me2
H3K9me3
H4K20me3
WT
Wapl KO

 © The Author(s)

Figure EV3. Wapl depletion re-positions Phf2 with cohesin in the genome.

(**A**) Binding of H3K4me3 (in WT and *Wapl* KO), Phf2 (in WT and *Wapl* KO), Smc3 (in WT and *Wapl* KO), and CTCF (in WT and *Wapl* KO) at two representative loci determined by ChIP-seq. The tracks from Fig. 5A are presented again in the left panel, now expanded to include all conditions. (**B**) Venn diagram showing the overlap between ChIP-seq peaks of Phf2 in WT and *Wapl* KO. (**C**) Pile-up heat maps and summary plots of ChIP-seq signals of H3K4me3 (in WT and *Wapl* KO), Phf2 (in WT and *Wapl* KO), Smc3 (in WT and *Wapl* KO), and CTCF (in WT and *Wapl* KO) at overlap groups indicated in (**B**). The panels from Fig. 5C are shown here again to facilitate a direct side-by-side comparison with the extended panels presented in this figure. (**D**) Summary plots of ChIP-seq signals obtained for Phf2, Smc3, and CTCF at loop anchors. The binding at left and right loop anchors was assessed in WT (left) and *Wapl* KO MEFs (right). (**E**) Triangular heatmap illustrating the overlap between ChIP-seq peaks of H3K4me3 in WT, *Phf2* KO, and *Wapl* KO. (**F**) Venn diagram showing the overlap between ChIP-seq peaks of Phf2, H3K4me3, and Smc3 in *Wapl* KO. (**G**) Fluorescence microscopy images for H3K4me2, H3K4me3, H3K9me1, H3K9me2, H3K9me3, H4K20me3, and Scc1 in WT and *Wapl* KO MEFs. Representative images of WT or *Wapl* KO MEFs stained with DAPI and antibodies to H3K4me2 (top-left), H3K4me3 (top-middle), H3K9me1 (top-right), H3K9me2 (bottom-left), H3K9me3 (bottom-middle), H4K20me3 (bottom-right) and Scc1. Scale bar, 10 μm. Source data are available online for this figure.

© The Author(s)

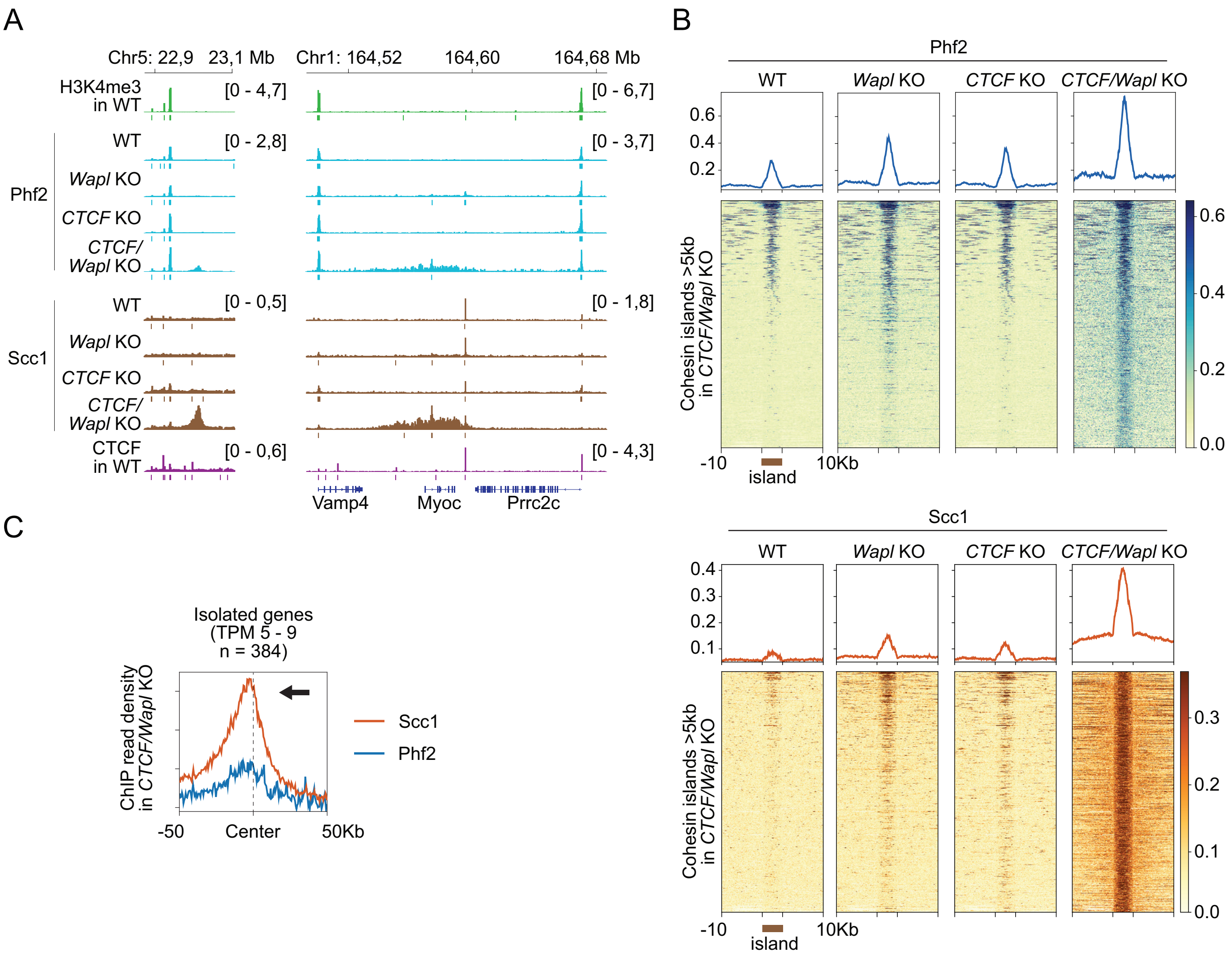

Figure EV4. Wapl and CTCF depletion re-positions Phf2 with cohesin in the genome.

(**A**) Binding of H3K4me3, Phf2 (in WT, *Wapl* KO, *CTCF* KO, and *CTCT/Wapl* KO MEFs), Scc1 (in WT, *Wapl* KO, *CTCF* KO, and *CTCT/Wapl* KO MEFs), and CTCF at two representative loci determined by ChIP-seq. The tracks from Fig. 5D are presented again in the right panel, now expanded to include all conditions. (**B**) Pile-up heat maps and summary plots of ChIP-seq signals obtained for Phf2 and Scc1 (in WT, *Wapl* KO, *CTCF* KO, and *CTCT/Wapl* KO MEFs) at cohesin islands found in *CTCF/Wapl* KO MEFs bigger than 5 kb (islands were stretched to equal size). The panels from Fig. 5E are shown here again to facilitate a direct side-by-side comparison with the extended panels presented in this figure. (**C**) Summary plots of ChIP-seq signals obtained for Scc1 and Phf2 in *CTCT/Wapl* KO MEFs for cohesin islands where transcription came only from one side, as measured by RNA-seq in Busslinger et al, 2017.

 © The Author(s)

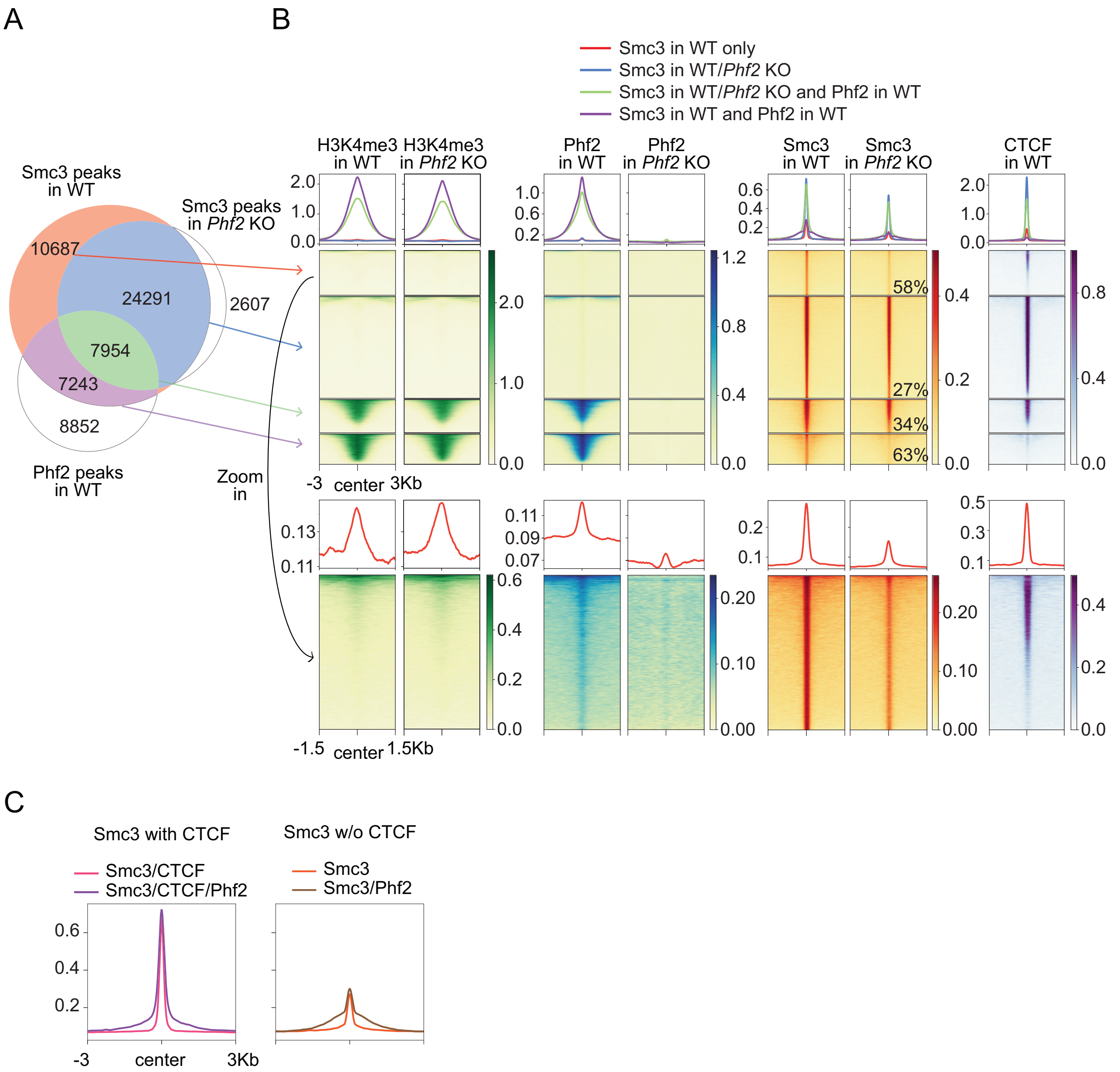

Figure EV5. Phf2 localizes cohesin to TSSs in the absence of CTCF.

(**A**) Venn diagram showing the overlap between ChIP-seq peaks obtained for Smc3 (in WT and *Phf2* KO), and Phf2 in WT. (**B**) Pile-up summary plots (top) and heat maps (bottom) of ChIP-seq signals obtained for H3K4me3 (in WT and *Phf2* KO), Phf2 (in WT and *Phf2* KO), Smc3 (in WT and *Phf2* KO), and CTCF at overlap groups indicated in (**A**). Numbers indicate the reduction in ChIP-seq signal of Smc3 peaks in *Phf2* KO MEFs at overlap groups indicated in (**A**). The Zoom-in panels show the indicated sub-groups of ChIP-seq signals at different color scales. The panels from Fig. 8C are shown here again to facilitate a direct side-by-side comparison with the extended panels presented in this figure. (**C**) Summary plots of ChIP-seq signals obtained for Smc3 at Smc3 peaks colocalizing with CTCF (left), or without CTCF (right). Peaks were further divided into colocalizing with Phf2 peaks or not.

© The Author(s)
